# Supplementary material for: Diagnostic pathways for breast cancer in 10 International Cancer Benchmarking Partnership (ICBP) jurisdictions: an international comparative cohort study based on questionnaire and registry data
Source: BMJ Open. 2022 Dec 14;12(12):e059669. doi: 10.1136/bmjopen-2021-059669 (PMC9756230; doi:10.1136/bmjopen-2021-059669)
Supplement: Supplementary data [file bmjopen-2021-059669supp001.pdf]

BMJ Publishing Group Limited (BMJ) disclaims all liability and responsibility arising from any re[illegible]
placed on this supplemental material which has been supplied by the author(s)

ICBP International Cancer Benchmarking Partnership

Additional File 1. Patient Questionnaire

International Cancer Benchmarking Partnership Module 4

Patient questionnaire

Breast Cancer

Thank you very much for taking the time to fill in this questionnaire – it should take about 20 minutes to complete. We are sending the questionnaire to a large sample of people who we understand have had a diagnosis of breast cancer. If this has been sent to you in error and you do not have cancer, please do not continue and return the documents in the prepaid envelope.

Our aim is to gain a better understanding of the process by which people have their cancer diagnosed. We would also like to find out more about the symptoms they experience (if any), and the pathway they follow from start of symptoms to treatment of their cancer. This will help in identifying ways in which cancers can be diagnosed quickly and effectively. Thank you once again for your time.

Sample

This information is confidential and will not be passed to anyone involved in your treatment.

Name: ____________________

Date of Birth: ____________________

Address: ____________________

Copyright property of the International Benchmarking Partnership (ICBP) and Module 4 collaborators.
Usage must be authorised

ICBP
International Cancer Benchmarking Partnership

Consent form

Please read the consent form and sign your name and date **BELOW**.

If you require any clarification, please do not hesitate to ring the study team members. Their contact details are found on the information sheet.

Please be reassured that your responses are completely confidential and will not be passed to anyone involved in your treatment. For the purposes of the study it is important that you agree to consent to all the statements listed below.

- I confirm that I have read the attached information sheet and I understand why the research is being done.
- I am willing for the team to request information from my GP and hospital doctors which is relevant to the audit as described in the information sheet.
- I give permission for my details (name, address) to be given to the cancer registry (NHS Information Centre for Health and Social Care) for follow up.
- I agree for the information I have provided and any other relevant information from my medical records to be stored as described in the information sheet under the custodianship of University College London.
- I consent to sharing of coded data which contains no personal identifiers between researchers, some of whom are located outside the European Union.
- I consent for use of my data if I become mentally incapacitated during the course of the project.

I agree to all the statements listed and consent to participate in the study.

Name (Please print)

Signature: Date:

If we have any questions, may we phone you for clarification? (Please tick) ☐ **Yes** ☐ **No**

If **Yes**, please provide your telephone number:

Sample

Copyright property of the International Benchmarking Partnership (ICBP) and Module 4 collaborators. Usage must be authorised
27 May 2013 Version 4

BMJ Publishing Group Limited (BMJ) disclaims all liability and responsibility arising from any re
placed on this supplemental material which has been supplied by the author(s)

1. **Please can you confirm the details of your GP/GP practice (name, practice address – as best as you can remember): We appreciate that you may have more than one GP involved in your care – in which case, we are interested in the GP you would say provides the majority of your care, particularly relating to the cancer you've had diagnosed.**

Name of doctor ______________________

Name of practice ______________________

Address ______________________

____

____

Postcode ______________________

Town ______________________

Sample

Copyright property of the International Benchmarking Partnership (ICBP) and Module 4 collaborators.
Usage must be authorised

ICBP
International Cancer Benchmarking Partnership

2. Which of the following best describes the events which led to your diagnosis of cancer? (please tick only **ONE** answer)

	✓
I had symptoms/I noticed a bodily change and went to see a doctor (e.g. GP)	
I had symptoms/I noticed a bodily change and went/was taken to Accident and Emergency (A&E)	
I had seen a doctor/GP with symptoms, but went/was taken to Accident and Emergency (A&E) when things worsened	
I was being investigated by my doctor(s) for another problem during which time the cancer was discovered	
I had a cancer screening test (mammogram) as part of a breast screening programme (e.g. the NHS Breast Screening Programme in England – NHSBSP)	
Other (please describe):	

Sample

What date did you have this screening test? If you cannot remember the exact date, you can fill in the month and the year.

Day (optional), month, year

D	D	M	M	Y	Y	Y	Y

Copyright property of the International Benchmarking Partnership (ICBP) and Module 4 collaborators. Usage must be authorised

BMJ Publishing Group Limited (BMJ) disclaims all liability and responsibility arising from any re
placed on this supplemental material which has been supplied by the author(s)

3. The following health concerns or symptoms are commonly experienced with breast cancer.

Changes in the appearance of the breast, eg:<br>■ the nipple<br>■ the skin of the breast<br>■ the shape of the breast<br>■ the size of the breast
Nipple discharge including bleeding
Lump, swelling or thickening in breast or armpit
Fatigue
Unexplained weight loss
Loss of appetite

Sample

Please write down ALL health concern(s) or symptom(s) you may have had before contacting a doctor or taking part in screening. It does not matter if they are not included in the list above:

Please write your health concern(s) or symptom(s) in the boxes below:
1)
2)
3)
4)
5)
6)

	✓
This is not applicable to me (e.g. I did not have any symptoms), please tick	

Copyright property of the International Benchmarking Partnership (ICBP) and Module 4 collaborators. Usage must be authorised

ICBP
International Cancer Benchmarking Partnership

4. Please write down your best estimate of the date you noticed the first of these health concern(s) or symptom(s). If you cannot remember the exact date, you can fill in the month and the year.

Day (optional), month, year

D	D	M	M	Y	Y	Y	Y

	✓
This is not applicable to me (e.g. I had no symptoms), please tick	

5. Approximately how long did you have health concern(s) or symptom(s) before contacting a doctor? (Please think of the first visit to the doctor, not re-visits after that). Please tick only **ONE** answer.

	✓
Less than 1 week	
1-2 weeks	
3-4 weeks	
5-7 weeks	
2-5 months	
6-12 months	
More than 12 months	

Sample

	✓
This is not applicable to me (e.g. I had no symptoms), please tick	

Copyright property of the International Benchmarking Partnership (ICBP) and Module 4 collaborators.
Usage must be authorised

6a. Once you contacted a practice about your health concern(s) or symptom(s), how long did it take to get an appointment with a doctor? (Please think of the first visit to the doctor, to discuss your health concern(s) or symptom(s)). Please tick only **ONE** answer.

	✓
Same day/next day	
Within 1 week	
1-2 weeks	
3-4 weeks	
Longer	
If there was no waiting time (e.g. you went/were taken to A&E), please tick this box	
This is not applicable to me (e.g. I had no symptoms), please tick	

Sample

6b. What was the date you first saw your doctor about your health concern(s) or symptom(s)? If you cannot remember the exact date, you can fill in the month and the year.

Day (optional), month, year

D	D	M	M	Y	Y	Y	Y

	✓
This is not applicable to me (e.g. I had no symptoms), please tick	

Copyright property of the International Benchmarking Partnership (ICBP) and Module 4 collaborators. Usage must be authorised

ICBP International Cancer Benchmarking Partnership

7. How many times did you visit the following for the investigation of your symptoms before your cancer was diagnosed?

	Please write down the number of visits
GP	
Hospital	
Consultant/specialist outside of a hospital	

	✓
This is not applicable to me (e.g. I had no symptoms)	

8a. After your doctor referred you to a specialist, how long did it take you to get an appointment? Please tick only **ONE** answer.

	✓
Less than 1 week	
1-2 weeks	
3-4 weeks	
5-7 weeks	
2-5 months	
6-12 months	
More than 12 months	

	✓
This is not applicable to me (eg my doctor did not refer me), please tick	

Sample

Copyright property of the International Benchmarking Partnership (ICBP) and Module 4 collaborators. Usage must be authorised

8b. What was the date of your first appointment with a doctor, involved in investigating and/or treating your cancer, to whom you were referred?
If you cannot remember the exact date, you can fill in the month and the year.

Day (optional), month, year

D	D	M	M	Y	Y	Y	Y

This is not applicable to me (e.g. my doctor did not refer me), please tick	✓

9. What was the date you were told you had cancer? If you cannot remember the exact date, you can fill in the month and the year.

Day (optional), month, year

D	D	M	M	Y	Y	Y	Y

Sample

Copyright property of the International Benchmarking Partnership (ICBP) and Module 4 collaborators.
Usage must be authorised

ICBP
International Cancer Benchmarking Partnership

10. Have you had any of the following treatments for your cancer yet? If so, please can you estimate the date this treatment started? Please tick **ALL** that apply. If you cannot remember the exact date, you can fill in the month and the year.

	**Type of treatment**		**Date of treatment** (give first date if you had more than one)
a.	Surgery	☐ **Yes** ☐ **No**	Day (optional), month, year D D M M Y Y Y Y
b.	Chemotherapy	☐ **Yes** ☐ **No**	Day (optional), month, year D D M M Y Y Y Y
c.	Radiotherapy	☐ **Yes** ☐ **No**	Day (optional), month, year D D M M Y Y Y Y
d.	Other Please specify:	☐ **Yes** ☐ **No**	Day (optional), month, year D D M M Y Y Y Y
e.	Treatment not started yet	☐ **Yes**	

Sample

Copyright property of the International Benchmarking Partnership (ICBP) and Module 4 collaborators. Usage must be authorised

BMJ Publishing Group Limited (BMJ) disclaims all liability and responsibility arising from any re placed on this supplemental material which has been supplied by the author(s)

11. Who is the consultant doctor who has taken responsibility for diagnosing and or/treating your cancer?

Name of consultant:
Hospital name:
Hospital department:

Please can you answer some more general questions about your health?
It will help us in interpreting your responses to this questionnaire to know about your general health and other health problems you may have had in the past.

Sample

12. Looking back to the 2 years before you were diagnosed with cancer, would you say your general health was (Please tick only **ONE** answer.):

Very good	
Good	
Fair	
Poor	
Very poor	

13. Have you been treated before for any of the conditions below?
Please tick 'yes' or 'no' for each condition:

Heart disease	☐ **Yes** ☐ **No**
Stroke	☐ **Yes** ☐ **No**
Lung disease	☐ **Yes** ☐ **No**
Diabetes	☐ **Yes** ☐ **No**

Copyright property of the International Benchmarking Partnership (ICBP) and Module 4 collaborators.
Usage must be authorised

ICBP
International Cancer Benchmarking Partnership

Finally, a little more information about you. The information you provide below will help us to analyse the results of the survey in more detail.

14. Which of these best describes your ethnic group? (please tick one box, as appropriate). If you are descended from more than one ethnic or racial group, please tick the group you consider you belong to, or tick 'any other ethnic group'.

	✓		✓		✓		✓
White		Chinese		Black - Caribbean		Black - African	
Black - other		Indian		Pakistani		Bangladeshi	
Any other ethnic group, please specify:							

Sample

15. What is the main language spoken in your home? Please tick

	✓
English	
Other, please specify:	

16. What is the highest level of education you have achieved?
Please tick only **ONE** answer.

	✓
Finished school at or before the age of fifteen	
Completed GCSEs, O-levels or equivalent	
Completed A Levels or equivalent	
Completed further education but not a degree	
Completed a Bachelor's degree / Masters degree / PhD	
Other, please specify:	

Copyright property of the International Benchmarking Partnership (ICBP) and Module 4 collaborators.
Usage must be authorised

17. Have you ever smoked cigarettes, including hand-rolled ones, pipes or cigars?

☐ Yes ☐ No

18. Are you a current smoker, smoking either cigarettes, including hand-rolled ones, pipes or cigars?

☐ Yes ☐ No

19. If you are a current smoker or have smoked in the past, how many cigarettes, including hand-rolled ones, pipes or cigars on average do you smoke/have you smoked per day?

Number per day:	

Sample

Copyright property of the International Benchmarking Partnership (ICBP) and Module 4 collaborators.
Usage must be authorised

ICBP
International Cancer
Benchmarking Partnership

20. Further comments

Please add anything else that you would like to tell us about your cancer diagnosis or treatment.

Sample

Thank you very much for taking the time to complete this questionnaire.

Copyright property of the International Benchmarking Partnership (ICBP) and Module 4 collaborators.
Usage must be authorised

ICBP International Cancer Benchmarking Partnership

Additional File 2. Primary Care Audit

International Cancer Benchmarking Partnership Module 4

Primary Care Audit

Breast Cancer

Thank you very much for agreeing to fill in this questionnaire. As part of an international study examining differences in cancer survival, we are sending the questionnaire to health care providers of a sample of consented patients with cancer.

Our aim is to gain a better understanding of the process by which people have their cancer diagnosed – the symptoms they experience, and the pathway they follow from onset of symptoms to treatment of their cancer. This will help in identifying ways in which cancers can be diagnosed and treated quickly and effectively. Thank you once again for your time.

Sample

Please can you refer to your patient's notes in completing the questionnaire as this will help in obtaining accurate data on time points.

If you would prefer to return this questionnaire without the patient details, please tear off along the dotted line

ID-number: Jurisdiction-ID + Patient-ID:

Copyright property of the International Benchmarking Partnership (ICBP) and Module 4 collaborators. Usage must be authorised

BMJ Publishing Group Limited (BMJ) disclaims all liability and responsibility arising from any re
placed on this supplemental material which has been supplied by the author(s)

ICBP
International Cancer
Benchmarking Partnership

Sample

Patient information

ID-number: Jurisdiction-ID + Patient-ID:

Full name:

Address:

Postcode:

Date of birth: D D M M Y Y Y Y

Copyright property of the International Benchmarking Partnership (ICBP) and Module 4 collaborators.
Usage must be authorised

2 / May 2013 Version 4

BMJ Publishing Group Limited (BMJ) disclaims all liability and responsibility arising from any re
placed on this supplemental material which has been supplied by the author(s)

1. Duration of symptoms

Please estimate how long your patient had symptom(s), attributable to breast cancer, before attending your practice (or other health service).
We appreciate that identifying a 'date of first symptom' is not always straightforward – particularly when there are multiple and/or chronic symptoms. Nevertheless, we hope you can provide a 'best estimate'.

**Estimate of symptom duration** (please tick **one**):	✓	**What were the symptoms?** Please describe:
Less than 1 week		
1 to 4 weeks		
5 to 7 weeks		
2-5 months		
6-12 months		
More than 12 months		
Not possible to estimate		
No symptoms (e.g. screen detected cancers)		

Sample

Copyright property of the International Benchmarking Partnership (ICBP) and Module 4 collaborators. Usage must be authorised

ICBP
International Cancer Benchmarking Partnership

2. Pathway of presentation

2.1 Through what route did the patient first present? Please tick **ONE**.

	✓	
Your patient first presented to primary care (either in-hours or out-of-hours)		Please can you provide your best approximation of the date of this primary care visit<br>D D M M Y Y Y Y
Your patient presented straight to A&E (with or without your involvement)		
Your patient first presented to primary care, but then at a later date presented to A&E as an emergency (with or without your involvement)		Please can you provide your best approximation of the date of this primary care visit<br>D D M M Y Y Y Y
Your patient's breast cancer was diagnosed through an organised screening programme (e.g. not as a result of investigation of symptoms)		
Other – please describe:		

Sample

Copyright property of the International Benchmarking Partnership (ICBP) and Module 4 collaborators. Usage must be authorised

3. Date you ordered any tests/investigations in response to symptom(s).

We are interested in any kind of tests/investigations (e.g. imaging etc) that you may have ordered. Please only consider the tests/investigations that you ordered yourself. Please tick **ALL** that apply and put in the date that the test/investigation was ordered:

	✓	
Mammogram		D D M M Y Y Y Y
Breast ultrasound		D D M M Y Y Y Y
Breast biopsy		D D M M Y Y Y Y
Other (please specify):		D D M M Y Y Y Y

Sample

4. Date of referral to specialist medical services

At what date did you **first** refer the patient to hospital or another specialist transferring the responsibility for on-going investigation/treatment to other medical services?

D	D	M	M	Y	Y	Y	Y

Copyright property of the International Benchmarking Partnership (ICBP) and Module 4 collaborators. Usage must be authorised

ICBP
International Cancer Benchmarking Partnership

5. Nature of this referral

5.1 Do you know the date that the patient was seen for this referral?

- [ ] **Yes**, please provide the date: | D | D | M | M | Y | Y | Y | Y |
- [ ] **No**

5.2 If you did make a referral to specialist services, which of the following best describes the nature/characteristics of this referral? Please tick **one**.

	✓
Emergency admission: a referral to A&E (or equivalent) for immediate admission	
An urgent referral for assessment of cancer symptoms/signs/test results (Note this will be within 2 weeks for England/Wales)	
A less urgent referral in which cancer is raised as a possibility (Note this will be greater than 2 weeks for England/Wales)	
A more general referral for investigation and assessment without cancer mentioned	
No referral was made	
Other – please describe:	

Sample

5.3 Would you say this patient's diagnostic pathway was conducted predominantly in the public or private system? Please tick **one**.

	✓
Public healthcare system	
Private healthcare system	

Copyright property of the International Benchmarking Partnership (ICBP) and Module 4 collaborators. Usage must be authorised

6. Date of breast cancer diagnosis

This can be decided in different ways. Please provide whichever of the following dates you have to hand. Please tick **all** that apply.

	✓	
Date of histological confirmation [ideal]		D D M M Y Y Y Y
Date results of investigation (histological or other) confirming cancer received		D D M M Y Y Y Y
Date patient was told		D D M M Y Y Y Y
Date biopsy undertaken		D D M M Y Y Y Y
Date patient was first admitted to hospital because of the malignancy		D D M M Y Y Y Y
Other (please specify):		D D M M Y Y Y Y

Sample

Copyright property of the International Benchmarking Partnership (ICBP) and Module 4 collaborators.
Usage must be authorised

ICBP
International Cancer Benchmarking Partnership

7. Additional information

Finally, we are interested to know what other conditions your patient has, and the severity/impact of these conditions

Have you and/or any of your partners treated this patient (or has the patient been to hospital) for any of the following conditions? Please tick **ALL** that apply:

Cardiovascular disease	☐ Yes	☐ No
Stroke	☐ Yes	☐ No
Lung disease	☐ Yes	☐ No
Diabetes	☐ Yes	☐ No

Are there any other comments you would like to make about this patient?

Sample

Name (and title):

Signature:

Date:

Thank you very much for taking the time to complete this questionnaire.

Copyright property of the International Benchmarking Partnership (ICBP) and Module 4 collaborators. Usage must be authorised

ICBP
International Cancer Benchmarking Partnership

Additional File 3. Specialist Care Audit

International Cancer Benchmarking Partnership Module 4

Specialist Care Audit

Breast Cancer

Thank you very much for agreeing to fill in this questionnaire – it should take about 10 minutes to complete. As part of an international study examining differences in cancer survival, we are sending the questionnaire to health care providers of a sample of patients with cancer.

Our aim is to gain a better understanding of the process by which people have their cancer diagnosed – the symptoms they experience, and the pathway they follow from onset of symptoms to treatment of their cancer. We hope you can help us with information on this patient's cancer journey once they were referred to specialist cancer services. This will help in identifying ways in which cancers can be diagnosed and treated quickly and effectively.

Sample

Thank you once again for your time

Please can you refer to your patient's notes in completing the questionnaire, as this will help in obtaining accurate data on time points.

If you would prefer to return this questionnaire without the patient details, please tear off along the dotted line.

Your patient ______________________________

is participating in the study.

Copyright property of the International Benchmarking Partnership (ICBP) and Module 4 collaborators.
Usage must be authorised

ICBP
International Cancer
Benchmarking Partnership

Sample

Patient information

ID-number: Jurisdiction-ID + Patient-ID:

Full name:

Address:

Postcode:

Date of birth: D D M M Y Y Y Y

Copyright property of the International Benchmarking Partnership (ICBP) and Module 4 collaborators.
Usage must be authorised

2 / May 2013 Version 3

1. **Date patient first attended hospital/specialist services related to their cancer diagnosis.** We appreciate this date can at times be difficult to identify, particularly when there have been multiple visits in the lead up to a definitive diagnosis. Put another way, it's the date that the hospital/specialist service assumed responsibility for on-going investigation/treatment for your patient.

Day (optional), month, year

D	D	M	M	Y	Y	Y	Y

2. **How was the patient referred to the hospital/specialist services related to their cancer diagnosis?** Please tick.

Was it through a:

	✓		✓
GP referral		Screening	
Referral from general surgery clinic		Medical specialist/ Consultant referral	
Other referral – please specify:			

Sample

3. **Where did this first contact/appointment happen?** Please tick.
Which of the following best describes where this first contact/appointment took place?

	✓		✓
Emergency department ('A&E')		Medical outpatient department, please specify which department	
Oncology general outpatient department		Surgical outpatient department, please specify which department	
Other – please specify:			

Copyright property of the International Benchmarking Partnership (ICBP) and Module 4 collaborators. Usage must be authorised

ICBP
International Cancer
Benchmarking Partnership

4. Date of diagnosis

This can be decided in different ways.
Please tick and complete as many of the following dates as possible.

	✓	
Date of histological confirmation (ideal)		Day (optional), month, year D D M M Y Y Y Y
Date results of investigation confirming cancer received		Day (optional), month, year D D M M Y Y Y Y
Date patient was told		Day (optional), month, year D D M M Y Y Y Y
Date of biopsy		Day (optional), month, year D D M M Y Y Y Y
Date patient was first admitted to hospital because of the malignancy		Day (optional), month, year D D M M Y Y Y Y
Date of MDT confirmation of diagnosis		Day (optional), month, year D D M M Y Y Y Y
Other (please specify):		Day (optional), month, year D D M M Y Y Y Y

Sample

Copyright property of the International Benchmarking Partnership (ICBP) and Module 4 collaborators.
Usage must be authorised

5. Date treatment for the cancer commenced

Based on your records, when would you say that any treatment specifically targeting the patient's cancer started?

Day (optional), month, year

D	D	M	M	Y	Y	Y	Y

6. Additional information

Please can you provide any further information on the patient's cancer:

**TNM**, please tick as appropriate:	
0	
I	
IIA	
IIB	
IIC	
IIIA	
IIIB	
IIIC	
IV	
Not able to stage	

Sample

Copyright property of the International Benchmarking Partnership (ICBP) and Module 4 collaborators.
Usage must be authorised

6.1 Histological subtype:

	✓
Invasive Ductal	
Invasive Lobular	
Invasive Tubular	
DCIS	
Other (please specify):	

Sample

Copyright property of the International Benchmarking Partnership (ICBP) and Module 4 collaborators.
Usage must be authorised

BMJ Publishing Group Limited (BMJ) disclaims all liability and responsibility arising from any re
placed on this supplemental material which has been supplied by the author(s)

Further comments

Sample

Copyright property of the International Benchmarking Partnership (ICBP) and Module 4 collaborators.
Usage must be authorised

ICBP
International Cancer
Benchmarking Partnership

Name (and title):

Signature:

Date:

Sample

Are you a ... (please tick below):

Surgeon	
Medical Oncologist	
Clinical Oncologist	
Clinical Nurse Specialist	
Other (please specify):	

Thank you very much for taking the time to complete this questionnaire.

Copyright property of the International Benchmarking Partnership (ICBP) and Module 4 collaborators.
Usage must be authorised

Additional File 4. *ICBP M4 Rules for missing, incomplete, multiple response and out of range data*

1. Oversampling<br>To handle oversampling in Ontario, include only the first 430 consecutive breast cancer patients
2. Language/Participation in study/Presence of cancer<br>Exclude patients who checked “No, I don’t understand the language” or “I don’t want to participate in this study” or “I don’t have cancer”.
3. Survey responders<br>a) Exclude Patient/PCP/Specialist survey from the analysis, if it was not written by Patient/PCP/Specialist (example: a medical oncologist completed a PCP survey);<br>b) In the case of duplicates, include only the first survey (example: 2 specialists completed surveys for the same patient).
4. Age<br>a) Exclude patients with unknown age;<br>b) Exclude patients younger 40 years;<br>c) Use registry data, if Age is reported by both patient and registry.
5. No cancer or Previous cancer in the same organ<br>a) Exclude patents with no cancer based on registry data;<br>b) Exclude patients with previous cancer in the same organ based on data from registry or free-text for Presentation in the patient survey.
6. Date of consent<br>Exclude patients with date of consent, which is unknown, before 01.01.2013 or in the future.
7. Multiple responses to Dates<br>If multiple responses were given to the dates (of first symptom; screening; first presentation to primary care; referral; diagnosis; treatment start), then use the earliest date.
8. Order of Dates<br>The dates must be in the following order –<br>a) First symptom; first presentation to Primary Care; referral; diagnosis; treatment start.<br>b) Screening; diagnosis; treatment start.<br>If not, check for mistakes.
9. Date of first symptom<br>Date of first symptom is defined as date of first symptom from patient data.
10. Date of first presentation<br>Date of first presentation to Primary Care is defined as (in the order of declining priority):<br>a) date of first presentation to Primary Care from PCP data;<br>b) date of first presentation to Primary Care and A&E from PCP data;<br>c) date of first presentation to Primary Care from patient data.
11. Date of referral<br>Date of referral is defined as date of referral from PCP data.
12. Date of screening<br>Date of screening is defined as (in the order of declining priority):<br>a) date of screening from registry;<br>b) date of screening from patient data.
13. Date of diagnosis<br>*Definition*<br>a) If Registry reports both date of histological confirmation and date of confirming investigation, then use date of histological confirmation.<br>b) Date of diagnosis (based on patient data, PCP data, specialist data, registry data) is defined as (in the order of declining priority):<br>- date of diagnosis from registry;<br>- date of histological confirmation (from specialist data, PCP data);<br>- date of biopsy (from specialist data, PCP data);<br>- date of confirming investigation (from specialist data, PCP data);<br>- date of first hospital admission (from specialist data, PCP data);<br>- date of MDT confirmation (from specialist data);<br>- date patient was told (from specialist data, PCP data);<br>- other date of diagnosis (from specialist data, PCP data, patient data);<br>Choose a Date from a lower level of hierarchy, if the Date from a higher level is after the Date of consent or more than 9 months (=271 days) before the Date of consent.<br><br>*Exclusion criteria*<br>a) Unknown date of diagnosis;

b) Date of diagnosis is after the date of consent;
c) Date of diagnosis is more than 9 months before the Date of consent.

14. Date of treatment start
    a) Date of treatment start from patient data is defined as the earliest of the treatment dates for Surgery, Chemo, Radio and Other;
    b) Date of treatment start (based on registry data, specialist data, patient data) is defined as (in the order of declining priority):
        - date of treatment start from registry data,
        - date of treatment start from specialist data,
        - date of treatment start from patient data,
        - anticipated date of treatment from patient data.

15. Imputation of missing day in the date
    Imputation rules for missing day (given month and year are known):
    a) Set missing day to ‘16’;
    b) Consider adjacent dates in a backwards order (from “Treatment” to “First symptom”). For each pair of such adjacent dates: If dates are not in a logical order (e.g. “Treatment” is before “Diagnosis”), but month and year are the same in both dates, and the day was imputed to ‘16’ in one of the dates:
    - Recode the day imputed earlier to ‘16’ to the day from the adjacent date.

16. Considering time
    If patient gave multiple answers to the “How long did you have symptoms before contacting a doctor?” question, then use the option with the shortest time interval.

17. Delay arranging appointment
    If patient gave multiple answers to the “How long did it take to get an appointment with PCP?” question, then use the option with the shortest time interval.

18. Duration of symptoms
    If PCP gave multiple answers to the “Duration of symptoms” question, then use the option with the shortest time interval.

19. Definition of Presentation
    A. Define Presentation within a Data Source (Patient, PCP)
    1. Review the free-text for Presentation (Patient, PCP) and re-code, if possible.
    2. If PCP reports ‘VisitPCP and AE’ or ‘VisitPCP’ as Presentation and no symptoms, then check Patient’s records. If Patient reports ‘Screening’ and no symptoms, then re-code Presentation for this case as ‘Screening’.
    3. If PCP reports ‘Screening’ as Presentation and at least one symptom (or “Duration of Symptoms”, rule 18), then re-code Presentation to ‘Other non-screen-detected’-option.
    4. If PCP reports ‘Other’ as Presentation and at least one symptom (or “Duration of Symptoms”, rule 18), then re-code Presentation to ‘Other non-screen-detected’-option.
    5. If Patient reports ‘Screening’ as Presentation and at least one symptom (or date of first symptom), then re-code Presentation to ‘Other non-screen-detected’- option.
    6. If Patient reports ‘Other’ as Presentation and at least one symptom (or date of first symptom or “Considering time” (rule 16) or “Delay arranging appointment” (rule 17), then re-code Presentation to ‘Other non-screen-detected’-option.
    7. In the case of multiple Presentation responses (Patient, PCP sources) - use a single option (in the order of declining priority):
        a) ‘VisitPCP and AE’,
        b) ‘VisitPCP´, ‘AE´ (if both ‘VisitPCP´ and ‘AE´ are given, then re-code as ‘VisitPCP and AE’),
        c) ‘Other non-screen-detected’,
        d) ‘Screening’,
        e) ‘Investigation for another problem’ ,
        f) ’Other”

    B. Define Presentation from Alternative Data
    If Presentation hasn’t been reported in either of data sources, then define it as (in the order of declining priority):
    1. ‘Other non-screen-detected ‘, if PCP reports at least one symptom (or “Duration of symptoms”, rule 18);
    2. ‘Other non-screen-detected ‘, if Patient reports at least one symptom (or date of first symptom);
    3. ‘Other non-screen-detected ‘, if Patient reports “Considering time” (rule 16) or “Delay arranging appointment” (rule 17) and no screening date;
    4. ‘Screening’, if Patient reports screening date and no symptoms and no date of first symptom;

    C. Define Presentation from Data Source Hierarchy

1. In Wales/England/Scotland (patients of 48-72 years old), Manitoba (patients 48-76 years old), N Ireland, Sweden and Denmark: if Registry reports 'Screening' – use Presentation-data from Registry data.
2. In Wales/England/Scotland (patients of 48-72 years old), Manitoba (patients 48-76 years old), N Ireland, Sweden and Denmark: if Registry reports ' No Screening'– use Presentation-data from (in the order of declining priority):
   a. PCP data (not available in Sweden);
   b. Patient data;

If PCP (or Patient, in the case of PCP data is not available) reports 'Screening', then code Presentation as 'Other non-screen-detected'.

If Presentation from PCP and Patient datasets is missing, then code Presentation as 'Other non-screen-detected'.

3. In Wales/England/Scotland (patients of 48-72 years old), Manitoba (patients 48-76 years old), N Ireland, Sweden and Denmark: if screening status from Registry is missing – use Presentation-data from (in the order of declining priority):
   a. PCP data (not available in Sweden);
   b. Patient data;

4. In Wales/England/Scotland (patients of <48 or >72 years old), Manitoba (patients of <48 or >76 years old), Norway and Ontario – use Presentation-data from (in the order of declining priority):
   a. PCP data;
   b. Patient data.

5. In Victoria: if Registry reports 'Screening organised by government' – use Presentation-data from Registry data.

6. In Victoria: if Registry reports 'No Screening organised by government' – use Presentation-data from (in the order of declining priority):
   a. PCP data;
   b. Patient data.

20. Patient interval
The Patient interval for non-screen-detected patients is defined as (in the order of declining priority):
a) "Date of first presentation to Primary Care" (rule 10) minus "Date of first symptom" (rule 9);
b) If the interval in (a) is unknown or negative: Calculate the interval as the middle of "Considering time" (rule 16) plus the middle of "Delay arranging appointment" (rule 17);
c) If the interval in (a) is unknown or negative and the interval in (b) is unknown: Calculate the interval as the middle of "Duration of symptoms interval" (rule 18).

21. Primary Care interval
The Primary Care interval for non-screen-detected is defined as "Date of referral" (rule 11) minus "Date of first presentation to Primary Care" (rule 10).

22. Diagnostic interval
a) The Diagnostic interval for non-screen-detected is defined as "Date of diagnosis" (rule 13) minus "Date of first presentation to Primary Care" (rule 10);
b) The Diagnostic interval for screen-detected patients is defined as "Date of diagnosis" (rule 13) minus "Date of screening" (rule 12).

23. Treatment interval
The Treatment interval is defined as "Date of treatment start" (rule 14) minus "Date of diagnosis" (rule 13).

24. Total interval
a) The Total interval for non-screen-detected patients is defined as "Date of treatment start" (rule 14) minus "Date of first symptom" (rule 9);
b) The Total interval for screen-detected patients is defined as "Date of treatment start" (rule 14) minus "Date of screening" (rule 12).

25. Range of Time intervals
The time intervals (Patient, Primary Care, Diagnosis, Treatment, Total) must be in range 0-1 year.

If > 1 year: set the interval to 365 days
If negative: set the interval to 0.

For each jurisdiction calculate the number of imputations due to:

a. unknown day in a date (given known month and year);<br>b. very large(>1 year) interval;<br>c. negative interval.
26. Number of visits<br>If patient gave multiple answers to the "Number of visits" questions, then use the option with a fewer number of visits.
27. Specialist waiting time interval<br>If patient gave multiple answers to the ""How long did it take to get an appointment with specialist?" question, then use the option with the shortest time interval.
28. Type of treatment<br>If patient ticked both "Yes" and "No" as answers to the "Type of treatment (Surgery, Chemotherapy, Radiotherapy)" questions, then choose "Yes" answer.
29. Health state<br>If patient gave multiple answers to the "Health state" question, then use the option with a better health condition.
30. Comorbidity<br>a) If patient ticked both "Yes" and "No" as answers to the "Comorbidity (Heart disease, Stroke, Lung disease, Diabetes)" questions, then choose "Yes" answer;<br>b) If both patient and PCP report "Comorbidity", then use the PCP Data.
31. Ethnicity<br>a) If patient didn't report "Ethnicity", then use the information from (in the order of declining priority):<br>- "Ethnicity_Other_Details";<br>- "Other main language spoken at home";<br>- "The main language spoken at home" (only for Victoria);<br>- "The main language spoken at home is the chief one for this jurisdiction"="Yes" given "Main language spoken at home is other than the main one for this jurisdiction"="No";<br>b) Consider Ethnicity as unknown, if answers to the "Ethnicity" question are multiple and belong to different categories ('white', 'Asian', 'black', 'other').
32. Education<br>If patient gave multiple answers to the "Education" question, then use the option with a higher level of education.
33. Smoking Current<br>a) If patient ticked both "Yes" and "No" as answers to the "Smoking Current" question, then use "Yes" answer;<br>b) If patient hasn't ticked neither "Yes" nor "No, then consider this case as Unknown.
34. Smoking Number<br>If patient reports "SmokingNumber" as text, then re-code using following rules:<br>a) Where there is a number smoked /day – accept number;<br>b) Where a range has been given – take the upper value;<br>c) Where patient has put 10+ or 20+ - capture this as 11 or 21;<br>d) Where number of cigarettes smoked in the past and currently being smoked are provided - average the numbers;<br>e) Non entries code as "." ;<br>f) Non-smokers (eg, "nil", "N/A") are coded as "0".
35. Smoked ever<br>a) If patient ticked both "Yes" and "No" as answers to the "Smoking ever" question, then use "Yes" answer;<br>b) If patient hasn't ticked neither "Yes" nor "No": consider it as "Yes", if patient is a current smoker ("Smoking_Current="Yes"") or has specified a number of cigarettes ("SmokingNumber">0). Otherwise consider this case as Unknown.<br>c) If patient has ticked "No": recode it to "Yes", if patient is a current smoker "Smoking_Current="Yes").
36. Nature of referral<br>a) Review free-text for " Nature of referral" (PCP Data) and re-code, if possible;<br>b) In the case of multiple responses, use a single option as (in the order of declining priority):<br>- "Referral for immediate admission";<br>- "Urgent referral";<br>- "Less urgent referral";<br>- "General referral" ;<br>- "No referral";<br>- "Other".
37. Refer Public or Private<br>a) If PCP ticked both "Public" and "Private" as answers to the "Refer Public or Private" question, then use "Private" answer;<br>b) If PCP hasn't ticked neither "Public" nor "Private", then consider this case as Unknown.

38. <u>Type of referral</u><br>If specialist gave multiple responses to the "How was the patient referred…" question, then use a single option (in the order of declining priority):<br>- "Screening";<br>- "Respiratory clinic";<br>- "General surgery clinic";<br>- "General gynaecology";<br>- "Specialist/consultant";<br>- "PCP";<br>- "Other".
39. <u>First Attendance Place</u><br>If specialist gave multiple responses to the "First Attendance Place" question, then consider this case as Unknown.
40. <u>Stage-TNM</u><br>a) If specialist gave multiple responses to the "Stage_TNM" question, then use the highest category;<br>b) If registry gave multiple responses to the "Stage_TNM", then use a single option (in the order of declining priority):<br>- stage at time of diagnosis<br>- stage at surgery<br>- stage at oncology<br>c) If "Stage_TNM" is reported by both the specialist and registry, then use the registry data.

Additional File 5. *Comparisons of the participants and the study base of the eligible breast cancer women*

	Eligible women		Participating women		p-value
	n	(%)	n	(%)	
Women, total	19,419	(81.3%)	4,458	(18.7%)	
**Age**					
Under 40	83	(0.4%)	11	(0.2%)	<0.001*
40-49	2,874	(14.8%)	676	(15.2%)	
50-59	4,756	(24.5%)	1,222	(27.4%)	
60-69	5,436	(28%)	1,434	(32.2%)	
70-79	3,696	(19%)	791	(17.7%)	
80-89	2,092	(10.8%)	297	(6.7%)	
90+	383	(2.0%)	27	(0.6%)	
Missing	99	(0.5%)	0	(0.0%)	
**Deceased**					
3 months	176	(18.6%)	2	(5.1%)	<0.05
6 months	213	(22.5%)	5	(12.8%)	
9 months	236	(25.0%)	12	(30.8%)	
12 months	320	(33.9%)	20	(51.3%)	
**Tumour stage**					
I	7,220	(42.9%)	1,807	(49.0%)	<0.001*
II	6,231	(37.0%)	1,311	(35.6%)	
III	2,028	(12.0%)	341	(9.3%)	
IV	653	(3.9%)	87	(2.4%)	
missing	702	(4.2%)	139	(3.8%)	

* both with and without missing

Additional file 6. *The differences in interval lengths (days) between Wales, the reference, and the other nine jurisdictions.*

		**Wales**	**England**	**Scotland**	**N Ireland**	**Denmark**	**Ontario**	**Norway**	**Manitoba**	**Victoria**	**Sweden**
**Patient Interval (Symptomatic)**	Number	111	201	198	219	213	170	206	172	189	101
	Median	11 (ref.)	-3(-9,3)	-3 (-8,3)	-1 (-8,6)	**-7** (-12,-2)	7 (-1,16)	1 (-7,9)	4 (-2,11)	-4 (-9,2)	**17** (11,22)
	75th percentile	34 (ref.)	-7 (-21,7)	-4 (-12,3)	-2 (-8,5)	**-10** (-16,-4)	**19** (5,33)	**16** (7,25)	12 (-1,25)	-6 (-16,4)	**22** (8,36)
	90th percentile	73 (ref.)	21 (-19,61)	11 (-45,66)	30 (-13,73)	-14 (-64,35)	50 (-9,110)	**74** (31,117)	9 (-35,54)	33 (-13,80)	14 (-31,60)
**Diagnostic interval (Symptomatic)**	Number	111	200	197	216	207	166	203	184	191	101
	Median	29 (ref.)	**-18** (-22,-15)	**-10** (-14,-6)	**-16** (-19,-13)	**-22** (-25,-18)	**-6** (-9,-2)	**-10** (-15,-5)	-1 (-5,4)	**-18** (-22,-14)	**-17** (-23,-12)
	75th percentile	54 (ref.)	**-39** (-45,-33)	**-24** (-29,-19)	**-37** (-42,-33)	**-34** (-40,-28)	-3 (-13,6)	**-21** (-36,-6)	**-16** (-23,-8)	**-34** (-52,-15)	**-33** (-39,-27)
	90th percentile	92 (ref.)	**-68** (-88,-48)	**-58** (-91,-25)	**-62** (-80,-45)	**-55** (-75,-35)	**73** (35,112)	**-35** (-62,-8)	**-30** (-46,-15)	**-63** (-78,-47)	**-58** (-81,-35)
**Diagnostic interval (Screen-detected)**	Number	113	153	159	86	131	184	126	125	152	158
	Median	25 (ref.)	-11 (-22,1)	-7 (-18,5)	-7 (-19,4)	-1 (-17,15)	0 (-12,12)	1 (-11,13)	-2 (-15,10)	-5 (-17,6)	-12 (-24,0)
	75th percentile	45 (ref.)	**-24** (-36,-13)	**-17** (-28,-6)	**-20** (-31,-8)	-9 (-21,2)	-3 (-15,8)	-1 (-15,13)	-4 (-15,7)	**-14** (-25,-3)	**-21** (-34,-7)
	90th percentile	58 (ref.)	**-23** (-30,-16)	**-16** (-22,-11)	**-19** (-26,-12)	**-10** (-14,-6)	9 (-6,24)	8 (-3,20)	**55** (38,71)	**-17** (-26,-8)	**-17** (-20,-13)
**Diagnostic interval (All)**	Number	224	353	356	302	338	350	329	309	343	259
	Median	29 (ref.)	**-14** (-16,-12)	**-8** (-11,-6)	**-13** (-15,-11)	**-14** (-16,-11)	-2 (-5,1)	**-6** (-9,-2)	-1 (-5,2)	**-12** (-15,-10)	**-14** (-17,-11)
	75th percentile	49 (ref.)	**-30** (-34,-25)	**-18** (-23,-14)	**-29** (-33,-25)	**-20** (-25,-16)	-5 (-15,4)	**-11** (-20,-2)	**-8** (-12,-3)	**-22** (-26,-19)	**-25** (-32,-17)
	90th percentile	70 (ref.)	**-35** (-39,-31)	**-23** (-30,-16)	**-26** (-34,-19)	**-21** (-40,-2)	**20** (9,31)	-1 (-16,14)	**22** (7,38)	**-27** (-40,-13)	**-29** (-36,-21)
**Treatment interval (Symptomatic)**	Number	115	203	202	241	230	201	216	198	196	113
	Median	24 (ref.)	**6** (1,11)	1 (-3,5)	-3 (-7,1)	-3 (-7,1)	**12** (8,16)	-5 (-11,1)	**15** (11,19)	**-10** (-14,-6)	-2 (-6,2)
	75th percentile	33 (ref.)	**8** (4,12)	4 (-1,9)	-3 (-8,1)	-4 (-9,0)	**16** (11,21)	**-6** (-9,-2)	**22** (18,26)	**-9** (-16,-2)	-4 (-13,5)
	90th percentile	45 (ref.)	10 (-1,20)	11 (-4,26)	-4 (-19,11)	-1 (-14,12)	**20** (10,31)	-11 (-23,2)	**28** (10,46)	-11 (-26,4)	-3 (-15,10)
**Treatment interval (Screen-detected)**	Number	115	155	158	86	134	195	120	155	170	165
	Median	27 (ref.)	**5** (1,8)	**8** (4,11)	0 (-5,5)	**-6** (-10,-1)	**9** (7,12)	**-12** (-15,-10)	**12** (9,16)	-5 (-12,1)	**-3** (-6,-1)
	75th percentile	36 (ref.)	4 (-2,10)	10 (-3,22)	0 (-6,7)	-7 (-16,3)	**11** (3,20)	**-15** (-27,-4)	**15** (7,23)	-7 (-16,2)	-6 (-17,5)
	90th percentile	49 (ref.)	5 (-8,18)	**15** (4,25)	-9 (-20,3)	**-14** (-26,-2)	9 (-4,22)	**-24** (-35,-13)	12 (-1,24)	**-14** (-27,-1)	-11 (-21,0)
**Treatment interval (All)**	Number	231	359	361	330	368	400	341	353	366	278
	Median	25 (ref.)	5 (0,11)	4 (0,8)	-4 (-8,1)	**-5** (-9,-1)	**10** (6,14)	**-10** (-14,-6)	**14** (9,18)	**-10** (-14,-6)	-3 (-7,0)
	75th percentile	35 (ref.)	**6** (2,10)	**6** (1,10)	-4 (-8,0)	**-6** (-10,-2)	**13** (10,17)	**-11** (-16,-6)	**20** (16,23)	**-9** (-13,-5)	**-6** (-10,-2)
	90th percentile	46 (ref.)	**8** (2,14)	12 (-1,26)	**-6** (-10,-2)	**-6** (-9,-2)	**17** (11,23)	**-14** (-17,-12)	**23** (16,29)	**-11** (-14,-9)	**-7** (-10,-4)
**Total interval (Symptomatic)**	Number	104	190	186	205	189	173	184	155	177	98
	Median	70 (ref.)	**-14** (-24,-3)	**-12** (-23,-2)	**-20** (-31,-8)	**-25** (-38,-11)	**28** (15,42)	-15 (-34,5)	**22** (12,31)	**-28** (39,-17)	-1 (-11,10)

	75th percentile	96 (ref.)	**-16** (-27,-5)	-5 (-18,9)	**-18** (-33,-3)	**-25** (-39,-12)	**69** (31,107)	**27** (8,46)	**30** (8,53)	-13 (-27,0)	3 (-30,35)
	90th percentile	218 (ref.)	**-71** (-102,-41)	**-60** (-85,-34)	**-57** (-66,-48)	**-41** (-66,-16)	**79** (66,93)	8 (-15,30)	**-17** (-34,-1)	-21 (-67,26)	**-39** (-67,-10)
**Total interval (All)**	Number	217	343	337	291	320	354	298	277	327	253
	Median	60 (ref.)	**-9** (-14,-3)	-4 (-11,2)	**-13** (-18,-8)	**-15** (-26,-4)	**16** (6,26)	**-13** (-21,-6)	**17** (9,24)	**-18** (-24,-12)	**-17** (-25,-10)
	75th percentile	81 (ref.)	**-12** (-20,-4)	1 (-9,10)	**-13** (-20,-6)	**-14** (-20,-9)	**32** (24,41)	-4 (-12,4)	**35** (21,48)	**-19** (-25,-12)	**-17** (-26,-7)
	90th percentile	123 (ref.)	-13 (-34,9)	3 (-12,18)	-5 (-22,11)	-8 (-38,22)	**73** (33,114)	**32** (2,62)	**47** (13,82)	-12 (-37,13)	-24 (-51,4)

The differences for the median, 75th and 90th percentiles are calculated as marginal effects after quantile regression by setting the continuous covariate age to its mean value and comorbidity to the mode. Significant results are shown in bold. The actual number of days are included for Wales are shown in Table 5. Note that analyses of primary care intervals are presented in Table 6.

Additional File 7. *Agreement between the ICBP M4 Presentation-rule (without using registry data) and registry information to define if a breast cancer case was screen detected, in Wales, England, Scotland, N Ireland, Denmark, Manitoba and Sweden*

Jurisdiction	Number of cases	Agreement on screening-status between ICBP M4 and Registry %	Kappa	(95%CI)
Wales	227	96	0.91	(0.86-0.97)
England	341	95	0.89	(0.84-0.93)
Scotland	360	97	0.94	(0.90-0.97)
N Ireland	331	91	0.77	(0.69-0.84)
Denmark	342	91	0.81	(0.74-0.87)
Manitoba	364	84	0.68	(0.60-0.76)
Sweden	284	87	0.75	(0.67-0.82)

Additional File 8. ICBP M4 ethics, working and academic reference groups

Section 1 - Summary of the (ethical) approvals obtained in the ten jurisdictions for local data collection and data transfer to University College London (United Kingdom) and Aarhus University (Denmark).

	Date of Ethics Approval	Approvals obtained	Reference
Victoria	4 September 2012	Cancer Council Victoria Human Research Ethics Committee	HREC 1125
Manitoba	7 March 2013<br><br>15 April 2013	Health Research Ethics Board, University of Manitoba<br><br>Research Resource Ethics Committee, CancerCare Manitoba	HS15227 (H2012:105)<br><br>RRIC#28-2012
Ontario	7 November 2013<br><br>28 January 2014	University of Toronto Research Ethics Board	27881
Denmark	6 August 2013<br><br>19 June 2013	The Danish Data Protection Agency<br><br>According to Danish law and the Central Denmark Region Committees on Health Research Ethics, approval by the National Committee on Health Research Ethics was not required as no biomedical intervention was performed.	2013-41-2030<br><br>1-10-72-20-13
Sweden	23 October 2013	Ethics Review Board, Uppsala	2013/306
Norway	04 April 2013	Regional committees for medical and health research ethics	2013/136/REK nord
Wales	16 November 2012	NRES Committee East Midlands – Derby 2,<br><br>local R&D for each health board	11/EM/0420
Scotland	16 November 2012	NRES Committee East Midlands – Derby 2,<br><br>R&D for each health board,<br><br>Privacy Advisory Committee,<br><br>CHI Advisory Group	11/EM/0420
N Ireland	1 June 2012	ORECNI Ethical approval, local governance for each health Trust	12/NI/0053
England	16 November 2012	NRES Committee East Midlands – Derby 2<br><br>R&D for each Clinical Research Network	11/EM/0420

Section 2 – ICBP Module 4 Working Group

Alina Zalounina Falborg, Statistician, Research Unit for General Practice, Department of Public Health, Aarhus University, Bartholins Allé 2, 8000 Aarhus C, Denmark

Andriana Barisic, Research Associate, Department of Prevention and Cancer Control, Cancer Care Ontario, 620 University Avenue, Toronto, Ontario, M5G 2L7, Canada

Anna Gavin, Director, Northern Ireland Cancer Registry, Centre for Public Health, Queen's University Belfast, Mulhouse Building, Mulhouse Road, Belfast, BT12 6DP, United Kingdom

Anne Kari Knudsen, Administrative leader, Department of Cancer Research and Molecular Medicine, Norwegian University of Science and Technology, 7489 Trondheim, Norway

Breann Hawryluk, Project Planning Coordinator, Department of Patient Navigation, Cancer Care Manitoba, 675 McDermot Street, Winnipeg, Manitoba, Canada

Chantelle Anandan, Research Fellow, Centre for Population Health Sciences, University of Edinburgh, Doorway 1, Medical Quad Teviot Place, Edinburgh, EH8 9DX, United Kingdom

Conan Donnelly, Research Fellow, Centre for Public Health, Queen's University Belfast, Mulhouse Building, Mulhouse Road, Belfast, BT12 6DP, United Kingdom

David H Brewster, Scottish Cancer Registry, Information Services Division, NHS National Services Scotland, Gyle Square, 1 South Gyle Crescent, Edinburgh, EH12 9EB, United Kingdom

David Weller, James Mackenzie Professor of General Practice, Centre for Population Health Sciences, University of Edinburgh, Doorway 1, Medical Quad Teviot Place, Edinburgh, EH8 9DX, United Kingdom

Donna Turner, Epidemiologist/Provincial Director, Population Oncology, Cancer Care Manitoba, 675 McDermot Street, Winnipeg, Manitoba, Canada

Elizabeth Harland, Project Coordinator, Department of Epidemiology and Cancer Registry, CancerCare Manitoba, 675 McDermot Street, Winnipeg, Manitoba

Eva Grunfeld, Director, Knowledge Translation Research Network Health Services Research Program, Ontario Institute for Cancer Research; Professor and Vice Chair Research, Department of Family and Community Medicine, University of Toronto, 500 University Avenue, Toronto, Ontario, M5G 1V7, Canada

Evangelia Ourania Fourkala, Research Associate, Gynaecological Cancer Research Centre, Women's Cancer, Institute for Women's Health, University College London, United Kingdom

Henry Jensen, Research fellow, Research Unit for General Practice, Department of Public Health, Aarhus University, Bartholins Allé 2, 8000 Aarhus C, Denmark

Jackie Boylan, Research Fellow, Centre for Public Health, Queen's University Belfast, Mulhouse Building, Mulhouse Road, Belfast, BT12 6DP, United Kingdom

Jacqueline Kelly, Tumour Verification Officer, Northern Ireland Cancer Registry, Centre for Public Health, Queen’s University Belfast, Mulhouse Building, Mulhouse Road, Belfast, BT12 6DP, United Kingdom

Kerry Moore, Research Fellow, Centre for Public Health, Queen's University Belfast, Mulhouse Building, Mulhouse Road, Belfast, BT12 6DP, United Kingdom

Maria Rejmyr Davis, Head, Southern Sweden Regional Cancer Center, Medicon Village, Scheelevägen 8, building 404, 223 81 Lund, Sweden

Martin Malmberg, MD PhD, Senior Consultant, Department of Oncology, Lund University Hospital, SE-221 85 Lund, Sweden

Mats Lambe, Professor of Medical Epidemiology, Regional Cancer Center Uppsala and Department of Medical Epidemiology and Biostatics, Karolinska Institutet, SE-171 77 Stockholm, Sweden

Oliver Bucher, Epidemiologist, Department of Epidemiology and Cancer Registry, CancerCare Manitoba, 675 McDermot Street, Winnipeg, Manitoba

Peter Vedsted, Professor, Research Unit for General Practice, Department of Public Health, Aarhus University, Bartholins Allé 2, 8000 Aarhus C, Denmark

Rebecca Bergin, Senior Research Officer/ PhD Candidate, Centre for Behavioural Research in Cancer, 615 St Kilda Rd, Melbourne, Victoria, 3004, Australia

Rebecca-Jane Law, Research Project Support Officer, North Wales Centre for Primary Care Research, Bangor University, Gwenfro Units 4-8, Wrexham Technology Park, Wrexham, LL13 7YP, United Kingdom

Richard D Neal, Professor of Primary Care Oncology, Academic Unit of Primary Care, Leeds Institute of Health Sciences, University of Leeds, Leeds LS2 9NL, United Kingdom

Sigrun Saur Almberg, Researcher, Department of Cancer Research and Molecular Medicine, Faculty of Medicine, Norwegian University of Science and Technology (NTNU), N-7491 Trondheim, Norway

Therese Kearney, Research fellow, Northern Ireland Cancer Registry, Centre for Public Health, Queen's University Belfast, Mulhouse Building, Mulhouse Road, Belfast, BT12 6DP, United Kingdom

Tindie Kalsi, Project Manager, Gynaecological Cancer Research Centre, Women's Cancer, Institute for Women's Health, University College London, United Kingdom

Victoria Cairnduff, Statistician, Northern Ireland Cancer Registry, Centre for Public Health, Queen's University Belfast, Mulhouse Building, Mulhouse Road, Belfast, BT12 6DP, United Kingdom

Victoria Hammersley, Researcher, Centre for Population Health Sciences, University of Edinburgh, Doorway 1, Medical Quad Teviot Place, Edinburgh, EH8 9DX, United Kingdom

Victoria White, Deputy Director, Centre for Behavioral Research in Cancer, Cancer Council Victoria, 615 St Kilda Road, Melbourne, Victoria, 3004, Australia

Usha Menon, Professor of Gynaecological Oncology and Head, Gynaecological Cancer Research Centre, Women's Cancer, Institute for Women's Health, University College London, United Kingdom

Yulan Lin, Postdoc, Department of Cancer Research and Molecular Medicine, Faculty of Medicine, Norwegian University of Science and Technology (NTNU), N-7491 Trondheim, Norway

Section 3 – ICBP Module 4 Academic Reference Group:

Prof Jan Willem Coebergh, Professor of Cancer Surveillance, Department of Public Health, Erasmus Universiteit Rotterdam, Rotterdam, the Netherlands

Jon Emery, Professor of Primary Care Cancer Research, University of Melbourne and Clinical Professor of General Practice, University of Western Australia, Australia

Dr Stefan Bergström, Senior consultant oncologist, Department of Oncology, Gävle, Sweden

Dr Monique E van Leerdam, Erasmus MC University Medical Centre, the Netherlands

Prof Marie-Louise Essink-Bot, Academic Medical Centre, Amsterdam University, the Netherlands

Prof Una MacLeod, Senior Lecturer in General Practice and Primary Care, Hull-York Medical School, United Kingdom
