# Supplementary material for: Diagnostic pathways for breast cancer in 10 International Cancer Benchmarking Partnership (ICBP) jurisdictions: an international comparative cohort study based on questionnaire and registry data
Source: BMJ Open. 2022 Dec 14;12(12):e059669. doi: 10.1136/bmjopen-2021-059669 (PMC9756230; doi:10.1136/bmjopen-2021-059669)
Supplement: Author's manuscript [file bmjopen-2021-059669.draft_revisions.pdf]

BMJ Open is committed to open peer review. As part of this commitment we make the peer review history of every article we publish publicly available.

When an article is published we post the peer reviewers' comments and the authors' responses online. We also post the versions of the paper that were used during peer review. These are the versions that the peer review comments apply to.

The versions of the paper that follow are the versions that were submitted during the peer review process. They are not the versions of record or the final published versions. They should not be cited or distributed as the published version of this manuscript.

BMJ Open is an open access journal and the full, final, typeset and author-corrected version of record of the manuscript is available on our site with no access controls, subscription charges or pay-per-view fees (http://bmjopen.bmj.com).

If you have any questions on BMJ Open's open peer review process please email info.bmjopen@bmj.com

Diagnostic pathways for breast cancer in ten International Cancer Benchmarking Partnership (ICBP) jurisdictions: An International comparative cohort study based on questionnaire and registry data

Journal:	*BMJ Open*
Manuscript ID	bmjopen-2021-059669
Article Type:	Original research
Date Submitted by the Author:	03-Dec-2021

Complete List of Authors:	Vedsted, Peter; Aarhus Universitet, Research Unit for General Practice<br>Weller, David; University of Edinburgh, General Practice<br>Zalounina Falborg, Alina; Research Unit for General Practice, Department of Public Health, Aarhus University<br>Jensen, Henry; Research Unit for General Practice, Department of Public Health, Aarhus University; Research Unite for General Practice, Department of Public Health, Aarhus university<br>Kalsi, Jatinderpal; University College London, Gynaecological Cancer Research Centre<br>Brewster, David; NHS National Services Scotland, Scottish Registry, Information Services Division<br>Lin, Yulan; NTNU Fakultet for ingeniorvitenskap og teknologi Trondheim, Department of Cancer Research and Molecular Medicine<br>Gavin, Anna; Queen's University Belfast, N. Ireland Cancer Registry<br>Barisic, Andriana; Cancer Care Ontario, Renal Network<br>Grunfeld, Eva; Ontario Institute for Cancer Research, Dept. of Family and Community Medicine<br>Lambe, Mats; Regional Cancer Centre of central Sweden, University Hospital<br>Malmberg, Martin; Lund University Hospital, 9. Department of Oncology<br>Turner, Donna; Cancer Care Manitoba, Population Oncology<br>Harland, Elizabeth; CancerCare Manitoba, Department of Epidemiology and Cancer Registry<br>Hawryluk, Breann; CancerCare Manitoba, Patient Navigation<br>Law, Rebecca-Jane; Bangor University, North Wales Centre for Primary Care Research<br>Neal, Richard; University of Leeds,<br>White, Victoria; Cancer Council Victoria, CBRC; Deakin University Faculty of Health,<br>Bergin, Rebecca; Cancer Council Victoria, Centre for Behavioral Research in Cancer<br>Harrison, Samantha; Cancer Research UK, Policy and Information<br>Menon, Usha; University College London, Women's Cancer<br>Working Group, ICBP; Cacncer Research UK, Policy and Information

Keywords:	Breast tumours < ONCOLOGY, Epidemiology < ONCOLOGY, Adult oncology < ONCOLOGY, International health services < HEALTH SERVICES ADMINISTRATION & MANAGEMENT, PUBLIC HEALTH

SCHOLARONE™
Manuscripts

I, the Submitting Author has the right to grant and does grant on behalf of all authors of the Work (as defined in the below author licence), an exclusive licence and/or a non-exclusive licence for contributions from authors who are: i) UK Crown employees; ii) where BMJ has agreed a CC-BY licence shall apply, and/or iii) in accordance with the terms applicable for US Federal Government officers or employees acting as part of their official duties; on a worldwide, perpetual, irrevocable, royalty-free basis to BMJ Publishing Group Ltd ("BMJ") its licensees and where the relevant Journal is co-owned by BMJ to the co-owners of the Journal, to publish the Work in this journal and any other BMJ products and to exploit all rights, as set out in our licence.

The Submitting Author accepts and understands that any supply made under these terms is made by BMJ to the Submitting Author unless you are acting as an employee on behalf of your employer or a postgraduate student of an affiliated institution which is paying any applicable article publishing charge ("APC") for Open Access articles. Where the Submitting Author wishes to make the Work available on an Open Access basis (and intends to pay the relevant APC), the terms of reuse of such Open Access shall be governed by a Creative Commons licence – details of these licences and which Creative Commons licence will apply to this Work are set out in our licence referred to above.

Other than as permitted in any relevant BMJ Author's Self Archiving Policies, I confirm this Work has not been accepted for publication elsewhere, is not being considered for publication elsewhere and does not duplicate material already published. I confirm all authors consent to publication of this Work and authorise the granting of this licence.

For peer review only

Diagnostic pathways for breast cancer in ten International Cancer Benchmarking Partnership (ICBP) jurisdictions: An International comparative cohort study based on questionnaire and registry data

Corresponding author:

Peter Vedsted

Research Unit for General Practice, Aarhus University

Bartholins Alle 2, 8000, Aarhus, Denmark

p.vedsted@ph.au.dk

1. **Peter Vedsted** *, Research Unit for General Practice, Department of Public Health, Aarhus University, Bartholins Allé 2, 8000 Aarhus C, Denmark, p.vedsted@ph.au.dk
2. **David Weller** *, Centre for Population Health Sciences, University of Edinburgh, Doorway 1, Medical Quad Teviot Place, Edinburgh, EH8 9DX, United Kingdom, david.weller@ed.ac.uk
3. **Alina Zalounina Falborg**, Research Unit for General Practice, Department of Public Health, Aarhus University, Bartholins Allé 2, 8000 Aarhus C, Denmark, az@ph.au.dk
4. **Henry Jensen**, Research Unit for General Practice, Department of Public Health, Aarhus University, Bartholins Allé 2, 8000 Aarhus C, Denmark, henry.jensen@feap.dk
5. **Jatinderpal Kalsi**, Gynaecological Cancer Research Centre, Women's Cancer, Institute for Women's Health, University College London, United Kingdom, j.k.kalsi@ucl.ac.uk
6. **David H Brewster**, Scottish Cancer Registry, Information Services Division, NHS National Services Scotland, Gyle Square, 1 South Gyle Crescent, Edinburgh, EH12 9EB, United Kingdom, David.Brewster@isd.csa.scot.nhs.uk
7. **Yulan Lin**, Department of Cancer Research and Molecular Medicine, Faculty of Medicine, Norwegian University of Science and Technology (NTNU), N-7491 Trondheim, Norway, yulan1984_lin@hotmail.com
8. **Anna Gavin**, Northern Ireland Cancer Registry, Centre for Public Health, Queen's University Belfast, Mulhouse Building, Mulhouse Road, Belfast, BT12 6DP, United Kingdom, A.Gavin@qub.ac.uk
9. Andriana Barisic, Department of Prevention and Cancer Control, Cancer Care Ontario, 620 University Avenue, Toronto, Ontario, M5G 2L7, Canada, Andriana.Barisic@renalnetwork.on.ca
10. **Eva Grunfeld**, Department of Family and Community Medicine, University of Toronto, 500 University Avenue, Toronto, Ontario, M5G 1V7, Canada, eva.grunfeld@utoronto.ca
11. **Mats Lambe**, Regional Cancer Center Uppsala and Department of Medical Epidemiology and Biostatics, Karolinska Institutet, SE-171 77 Stockholm, Sweden, mats.lambe@ki.se
12. **Martin Malmberg**, Department of Oncology, Lund University Hospital, SE-221 85 Lund, Sweden, Martin.Malmberg@skane.se
13. **Donna Turner**, Population Oncology, Cancer Care Manitoba, 675 McDermot Street, Winnipeg, Manitoba, Canada, donna.turner@cancercare.mb.ca
14. **Elizabeth Harland**, Department of Epidemiology and Cancer Registry, CancerCare Manitoba, 675 McDermot Street, Winnipeg, Manitoba, eharland@cancercare.mb.ca

For peer review only

15. **Breann Hawryluk**, Department of Patient Navigation, Cancer Care Manitoba, 675 McDermot Street, Winnipeg, Manitoba, Canada, Breann_haw@hotmail.com
16. **Rebecca-Jane Law**, North Wales Centre for Primary Care Research, Bangor University, Wrexham, LL13 7YP, United Kingdom, r.law@bangor.ac.uk
17. **Richard D Neal**, Academic Unit of Primary Care, Leeds Institute of Health Sciences, University of Leeds, Leeds LS2 9NL, United Kingdom, r.d.neal@leeds.ac.uk
18. **Victoria White,** Centre for Behavioral Research in Cancer, Cancer Council Victoria, 615 St Kilda Road, Melbourne, Victoria, 3004, Australia, vicki.white@cancervic.org.au
19. **Rebecca J Bergin,** Cancer Epidemiology Division at Cancer Council Victoria, 615 St Kilda Road, Melbourne, Victoria, 3004, Australia, Rebecca.Bergin@cancervic.org.au
20. **Samantha Harrison,** Policy, Information & Communications, Cancer Research UK, 2 Redman Place, London, E20 1JQ, UK, samantha.harrison@cancer.org.uk
21. **Usha Menon** *, MRC Clinical Trials Unit, Institute of Clinical Trials and Methodology, University College London, United Kingdom, u.menon@ucl.ac.uk
22. **ICBP Module 4 Working Group**

* equal contribution

Number of words: 3598
Number of tables: 6
Number of figures: 1
Number of Appendices: 7

For peer review only

For peer review only - http://bmjopen.bmj.com/site/about/guidelines.xhtml

Abstract

Objectives: Growing evidence suggests longer times between symptom onset to start of treatment affects breast cancer prognosis. The International Cancer Benchmarking Partnership Module 4 examines differences in breast cancer diagnostic pathways in 10 jurisdictions across Australia, Canada, Denmark, Norway, Sweden and the UK.

Design: Data was collated from 3,471 women aged >40 diagnosed first-time with breast cancer surveyed between 2013 and 2015, their primary care physician (PCP), cancer treatment specialists, and available registry data.

Results: Overall, 56% of women reported symptoms to primary care, and 66% first noticing lump/s or breast changes. PCPs reported 77% presented with symptom/s, of whom 81% were urgently referred with suspicion of cancer, (ranging 62-92%, Norway; Victoria). The median patient, primary care and diagnostic intervals (days) for symptomatic patients ranged: 3-29 (Denmark; Sweden), 0-20 (seven jurisdictions; Ontario), 8-29 (Denmark; Wales). The median treatment and total intervals (days) for all patients ranged: 15-39 (Norway, Victoria; Manitoba) and 4 -78 days (Sweden, Victoria; Ontario). The 10% longest waits ranged 101-209 days (Sweden; Ontario).

Conclusions: Large international differences in breast cancer diagnostic pathways exist, suggesting some jurisdictions examine strategies to optimise pathways and reduce time intervals. Targeted awareness interventions could also facilitate more timely diagnosis of breast cancer.

Keywords

Delayed diagnosis; Questionnaire; International comparisons, Early Detection of Cancer; Breast neoplasms; Health services, Health planning; Signs and Symptoms; Screening

For peer review only

Strengths and limitations of this study

- Internationally standardised survey methodology to explore and compare key intervals from symptom onset to treatment start.
- Comprehensive data pulled from state/provincial-level cancer registries and other sources to create as complete a record as possible of patient pathways to diagnosis and treatment
- Minimal recall bias through triangulation of different data sources and by ensuring that the patients received the questionnaire with a limited time window after the cancer diagnosis.
- Some jurisdictions were not able to recruit a sufficient number of patients to power this study
- Our cohort is not representative of all breast cancer patients as there was high self-selection of early stage breast cancer patients

Introduction

Breast cancer is the most common cancer among women in western countries. The incidence of female breast cancer is around 80 per 100,000 (standardised world population); by the age of 80, approximately 12% of women will have received a breast cancer diagnosis [1]. Although the prognosis of breast cancer has improved dramatically during recent decades [2], there are still important differences in disease-specific mortality and survival, both between and within countries [3,4]. For example, in 2010-14, the 5-year net survival varied from 85.6% in the UK, to 88.8% in Sweden, and 89.5% in Australia [2].

Differences in survival may relate to timely cancer diagnosis and access to optimal treatment and can also affect patient experience and healthcare costs [5-10]. Therefore, it becomes important to examine international differences in routes to diagnosis and treatment together with time intervals from first noticing symptoms until start of treatment.

Women differ in their help-seeking behaviours for breast cancer symptoms [11]. These behaviours might influence the time interval between first noticing symptoms until they present to healthcare [12]. Further, a

For peer review only - http://bmjopen.bmj.com/site/about/guidelines.xhtml

For peer review only

healthcare system's organisation might affect the way patients seek help, how healthcare professionals respond to these symptoms, and when and how they can refer for further investigations [13-16]. In some countries, breast cancer diagnosis is standardised or expedited with urgent referrals, where significant breast cancer specific 'red flag symptoms' are present – further, many countries have now implemented breast cancer screening programmes [17]. Therefore, routes to diagnosis and time intervals from first noticing symptoms to breast cancer diagnosis and treatment may vary between countries. This has consequences for patient outcomes and experience, as well as healthcare costs.

To date almost all breast cancer routes to diagnosis studies have been based on a single country [18]; international comparisons can shed light on factors which underpin observed differences. Accordingly, we undertook a study to explore pathways to diagnosis and treatment for female breast cancer in ten jurisdictions within six countries.

Methods

We undertook an international comparative cohort study based on questionnaire and registry data on newly diagnosed, first-time female breast cancer patients aged 40 or over.

Study context and management

Within the International Cancer Benchmarking Partnership (ICBP), Module 4 (ICBP M4) aims to explore differences in routes to diagnosis for four cancers; the methods used are described in detail elsewhere [19,20]. Briefly, the study was undertaken in ten jurisdictions in six countries: Australia (Victoria), Canada (Manitoba and Ontario), Denmark, Norway, Sweden and the United Kingdom (England, Northern Ireland, Scotland and Wales). We recruited jurisdictions with universal health care access, covering breast cancer investigations and screening programmes - and showing variation in breast cancer survival [20].

Identification of study population

For peer review only - http://bmjopen.bmj.com/site/about/guidelines.xhtml

Eligible patients were consecutively identified women aged 40 years or more with a first diagnosis of invasive breast cancer (ICD10 codes C50.0—C50.9), irrespective of symptomatic or screen-based diagnosis. Women with previous diagnoses of other cancers were included but women with a previous diagnosis of breast cancer or synchronous breast cancer were excluded.

Participants were identified via cancer registries and hospital databases, although processes varied locally. Processes were tested in each jurisdiction to ensure adaptation of data collection, questionnaire logistics and data management to the local settings [20]. Each jurisdiction aimed to recruit 200 symptomatic breast cancer patients, irrespective of the number of screen-based diagnoses. In Ontario and Victoria, the sampling continued beyond 200 patients for use in local studies. In Northern Ireland, the majority of screen-detected women were excluded at the sampling stage by the cancer registry.

Data sources

Data were collected primarily from three questionnaires sent to the eligible women (Additional File 1), their primary care physician (PCP; Additional File 2) and their cancer treatment specialists (CTSs; Additional File 3). This was supplemented with registry data (including diagnosis date, tumour stage and screening) in all jurisdictions except Ontario and Norway [19]. Data were anonymised prior to transfer to Aarhus University (Denmark) for analysis.

Questionnaire and registry data

The validated questionnaires included items on routes to diagnosis, symptoms, specific milestones from first noticing symptoms to starting treatment, patient socio-demographics and comorbidity [20]. Eligible patients were identified from cancer registries, and patients either contacted directly or via their PCP.

Women were sent questionnaires 3-9 months after diagnosis; PCPs and CTSs were sent questionnaires after patients consented to participation in the study. Completed questionnaires were returned to the local

For peer review only - http://bmjopen.bmj.com/site/about/guidelines.xhtml

research teams. Where possible, registries and disease databases provided additional checks and information on date of diagnosis, screening status and start of treatment. Date of diagnosis was based on the International Agency for Research on Cancer (IARC) hierarchy, and tumour stage using tumour, node, metastasis (TNM) classification [21,22].

Data handling

Curation of questionnaire data were primarily done centrally (Aarhus University, Demark) to ensure uniform application across jurisdictions.

A set of algorithms ('rules') to validate and combine data from the different sources in a standardised way was developed (Additional File 4); they employed a hierarchy-principle with regard to the order in which different data sources were used. Any queries were discussed and resolved with the local team.

Routes to diagnosis

Differences in the diagnostic routes were investigated drawing on the checklist from the Aarhus Statement [23]. Routes to diagnosis are outlined in Box 1:

BOX 1 – Routes to diagnosis

- **Screening**
- **Symptomatic:**
  - Visit PCP
  - Visit PCP and then accident and emergency department (A&E)
  - Direct to A&E
  - Investigation for another problem
- **Other/unknown routes to diagnosis**

Measures of time intervals

Time interval definitions were adapted from the Aarhus Statement and included the time-points outlined in Box 2 [23]; the intervals are represented graphically in Figure 1.

For peer review only - http://bmjopen.bmj.com/site/about/guidelines.xhtml

BOX 2 – Time intervals

a) First onset of symptoms: the time-point when first bodily change(s) and/or symptom(s) were first noticed by the patient.
b) First presentation to healthcare: the time-point at which the PCP noted a symptom that in retrospect could be due to the underlying cancer.
c) First referral to secondary care: the time-point at which the PCP referred the patient (and responsibility) to secondary/specialist care.
d) Date of diagnosis, following the IARC definition.[21]
e) Date of treatment start: when the patient started curative/palliative treatment (in Manitoba only curative treatment was registered).

INSERT FIGURE ONE ABOUT HERE

For screen-detected women, we used the date of the screening test as the start of the diagnostic and total interval measurements. The analyses include intervals for both symptomatic, screen-detected and all breast cancer patients. We included a categorical variable where the symptomatic women reported the waiting time for their appointment with the PCP. Cancers were only considered to be screen-detected if the woman or PCP had indicated a screening route and there were no symptoms nor symptomatic pathway reported. For Wales, England, Scotland, Northern Ireland, Denmark, Manitoba and Sweden the distinction between a screen-detected and non-screen-detected breast cancer was based on registry data from public screening programs (Table 1).

INSERT TABLE 1 ABOUT HERE

Covariates

General health status was measured using the self-reported general health item from the 12-Item Short Form Health Survey (SF12) [24]. Comorbidity was assessed through the patient questionnaire as presence of any of four chronic diseases (heart or lung diseases, stroke, or diabetes) and categorised into 'none', 'medium' (one or two), or 'high' (three or four). Educational level (from the patient questionnaire), was categorised as

For peer review only - http://bmjopen.bmj.com/site/about/guidelines.xhtml

For peer review only

'low' (vocational school or lower) and 'high' (university or higher). Smoking was categorised into 'current', 'past' or 'never' smoker. Symptoms reported by the PCP were divided into 'breast cancer specific symptoms' or 'other symptoms'. This was based on symptom coding, drawing on local clinical guidelines [17,24].

Statistical analysis

Patients with missing data on age, date of diagnosis and/or date of consent were excluded. Quantile regression [26] was used to estimate differences in intervals at the median (50th), 75th and 90th percentiles between all jurisdictions with Wales as the reference. Counting days, we used the 'qcount' procedure [27,28]. The differences in intervals were calculated as marginal effects after quantile regression by setting the continuous covariates to their mean values and the categorical covariates to their modes.

Due to zero-inflation, quantile regression could not be used for the primary care interval. Here a generalised linear model with Poisson family and log link and robust error variance was used to calculate the association (prevalence ratio (PR)) between longer primary care intervals and jurisdiction. Longer intervals were defined as over 5 or 14 days to test both intervals. The analyses were adjusted for age (continuous variable) and comorbidity (categorical variable). The significance level was set to 0.05 or less, and 95% confidence intervals (95%CI) were calculated when appropriate. Statistical analyses were carried out using STATA v14 software.

Sensitivity and validity analyses

Analyses were repeated using the per-protocol definition of a maximum of 6 months between diagnosis and patient consent, to assess the impact of including patients who consented at 9 months post-diagnosis. To estimate the effect of using patient-reported intervals only, a sensitivity analysis based solely on the patient questionnaire data was performed.

For peer review only

For peer review only - http://bmjopen.bmj.com/site/about/guidelines.xhtml

Kappa coefficients were used to assess the agreement on routes to diagnosis (screening and symptomatic presentation) between the different data sources. Lin's concordance correlation coefficient (CCC) was used to assess the agreement on dates between the different data sources [29].

Results

Patient characteristics and participation

Across all jurisdictions, 15,421 eligible women were identified between May 2013 and August 2015. A total of 4,593 (39.9%) of those contacted returned completed questionnaires and 3,471 (75.6%) could be included in the analyses. The patient flow, with identification, exclusion and responses for each jurisdiction is detailed in Supplementary Table 1. The response rates ranged from 24.1% in Norway to 77.9% in Denmark (Supplementary Table 1). Participating women were younger and had a less advanced tumour stage distribution than all eligible women in the study base (Additional File 5). The characteristics of the included women are detailed in Table 1.

INSERT TABLES 1 & 2 ABOUT HERE

First presentation and symptoms

Table 2 shows routes to diagnosis; 42.9% had a screen-detected breast cancer, with the highest proportion in Sweden (58.6%)[1]. For symptomatic patients, primary care was the place of first presentation for 85.6% of women, ranging from 69.2% in Ontario to 93.7% in England. According to the PCP, 81.1% of symptomatic women were urgently referred with a suspicion of cancer, ranging from 62.4% in Norway to 92.2% in Victoria (data not shown).

Table 3 shows the first symptom or sign reported by women, together with those reported by the PCP (for Sweden, only patient data were collected). Two thirds of symptomatic women noticed a lump or change in

[1] *note that the proportion of screen-detected cancers in Northern Ireland is lower due to the initial exclusion of screen-detected cancers*

For peer review only - http://bmjopen.bmj.com/site/about/guidelines.xhtml

the breast. This was also the most commonly reported sign by PCPs, followed by pain/tenderness, and change in the nipple. According to the PCP, 77% presented with a symptom or sign indicative of breast cancer. While one in ten women indicated fatigue as a symptom, this was rarely noted by the PCPs.

INSERT TABLES 5 & 6 ABOUT HERE

Time intervals

The median patient interval for symptomatic women varied from 3 days (Denmark) to 29 days (Sweden) (Table 4). Based on the quantile regression, the patient interval was 7 days shorter in Denmark and 17 days longer in Sweden compared with Wales (Figure 2 and Additional File 6). When comparing the 90th percentile, Norway (157 days) and Ontario (142 days) had the longest patient intervals (Table 4). Quantile regression showed that Norway had a significantly longer patient interval for the 90th percentile compared with Wales (74 days) (Figure 2).

INSERT FIGURE 2 ABOUT HERE

The median primary care interval for symptomatic women was 0 days, except in the two Canadian provinces and Victoria (Table 4). Women in these three jurisdictions had statistically significant higher likelihoods than women in Wales to have longer primary care intervals (both for more than 5 and 14 days) (Table 5).

INSERT TABLE 5 ABOUT HERE

The median diagnostic interval for symptomatic women ranged from 8 days in Denmark to 29 days in Wales (Table 4). At the 90th percentile, the diagnostic interval ranged from 36 days in England to 202 days in Ontario (Table 4). Compared with Wales, all jurisdictions except Ontario had shorter diagnostic intervals (Figure 2). For women with a screen-detected breast cancer the time intervals and pattern were similar, although screen-detected patients had significantly shorter 90th percentiles compared to symptomatic patients.

The median treatment intervals for all women ranged from 15 days in Norway and Victoria to 39 days in Manitoba. Women in England, Scotland, Ontario and Manitoba waited more than 28 days (four weeks) for

For peer review only - http://bmjopen.bmj.com/site/about/guidelines.xhtml

treatment after diagnosis (Table 4). Quantile regression showed smaller statistically significant differences in treatment intervals, with Manitoba and Ontario consistently having longer treatment intervals for the three percentiles (Figure 2). The treatment intervals were similar for screen-detected cancers.

The median total interval for all women from first symptom or date of screening test to start of treatment ranged from 42 days in Sweden and Victoria to 78 days in Ontario (Table 5). In Manitoba and Ontario, 25% of women had a total interval that was 30 days longer compared with Wales (81 days), whereas 25% of women in England, Northern Ireland, Denmark, Victoria and Sweden had a total interval that was between 12 to 19 days shorter compared with Wales (Figure 2 and Additional file 6). 10% of women from Ontario waited 73 days or more from first presentation until treatment start compared with women in Wales. Including the screen-detected breast cancers made the total interval shorter, with less variation between the jurisdictions.

There were differences in the time taken from women deciding to seek help and getting an appointment with a PCP. Getting an appointment 'within one week' was less often reported by women in Sweden (43%), Manitoba (50%), Ontario (58%) and Northern Ireland (64%) compared to women in other jurisdictions (above 70% in the other six jurisdiction with 84% in England and Victoria) (data not shown).

Validity tests

Comparing patient and PCP-reported screening with available screening registry data showed an almost perfect agreement (Kappa> 0.80) (Additional File 7). Comparing the dates between the different data sources showed a high agreement between all data sources (patient, PCP, CTS and register where applicable) for all types of dates (CCC = 0.94 for date of first presentation to primary care, CCC ≥ 0.94 for date of diagnosis and CCC = 0.93 for date of treatment).

Discussion

Main findings

For peer review only - http://bmjopen.bmj.com/site/about/guidelines.xhtml

This international survey of breast cancer patients diagnosed between 2013-15 revealed that four out of ten were screen-detected and more than half of women presented with symptoms, with the majority of these diagnosed after a visit to primary care. Three quarters of symptomatic patients had a symptom or sign indicative of breast cancer, and roughly eight in ten were urgently referred. Thus, despite the existence of screening programmes the majority still had their breast cancer diagnosed based on 'red-flag' symptoms.

The median time from women first noticing symptoms to start of treatment varied from 42 to 92 days across jurisdictions; this is attributable mainly to differences after first presentation - less variation is seen in the patient interval across jurisdictions.

We found that in some jurisdictions there is a 'tail' of women with excessively long waiting times from first noticing symptoms to presentation to the healthcare system.

Strengths and weaknesses

A major strength is our novel use of a standardised questionnaire survey in several countries to systematically examine routes to diagnosis and treatment. To ensure validity across jurisdictions, we drew on existing instruments and went through an extensive process of cognitive testing, piloting, translation and adaptation [20]. Data were enriched with information from national or state/provincial-level cancer registries for screening status, date of diagnosis and tumour stage. We developed algorithms to identify screen-detected cases, place of first presentation and time intervals, which showed good agreement for those jurisdictions where validation was possible. Using registries and undertaking clinical validation of breast cancer patients ensured minimal selection and information biases. It also made it possible to exclude women who previously had breast cancer, thus providing a reasonably homogeneous group of patients.

For peer review only

We included all first-time breast cancer patients, irrespective of route to diagnosis and assured at least 200 symptomatic cases per jurisdiction. We developed and implemented validated rules for the screening route, which showed high agreement with screening registries.

There were different classification systems for ethnicity and education across jurisdictions. We excluded these variables from the regression model to avoid introducing invalidating information bias in the model. Although this likely induced some residual confounding, it is unlikely that educational or ethnic differences could have produced the observed variation in time intervals.

We used a set of rules to ensure validity and consistency - and to preserve statistical precision [20]. The validity and sensitivity analyses suggested this approach was effective. To minimise misclassification, data interpretation during data entry by the local teams was reduced to an absolute minimum and all apparent data errors were checked against source data by local teams.

The effect of recall bias was minimised by the triangulation of data sources (e.g. patient, PCP, CTS and registry data), and by ensuring that the women received the questionnaire within a limited time window after diagnosis.

The overall response rate of 40% was comparable with similar studies among patients and PCPs [30], but varied markedly between jurisdictions, with a response rate of 78% in Denmark and 24% in Norway. This may have resulted in differential selection bias across jurisdictions. However, comparing the participants on a number of variables (e.g. tumour stage, presenting symptom, comorbidity, self-assessed health, smoking status) did not show any meaningful differences. Nevertheless, as recruitment strategies differed with some women contacted by the registries, some via PCPs (and via nurses in Northern Ireland) assessing eligibility, some selection bias might have been introduced via differing self-selection. This is underlined by the higher-than-expected proportion of stage I tumours amongst our respondents, when compared to an earlier

For peer review only - http://bmjopen.bmj.com/site/about/guidelines.xhtml

registry-based breast cancer study [3]. Women with early stage breast cancer are likely to be more able to participate in studies of this kind, compared to women with late stage disease.

The statistical precision of the study was sufficient, as we were able to show clinically significant differences of one week in time intervals, appropriate for clinically relevant differences.

Comparison with other studies

Median patient intervals for breast cancer have varied in previous studies – in Denmark the interval was 14 days in a 2004 study [30], and only 3 days in 2015 (using similar methods) [31], possibly reflecting cancer pathway improvements over that period. Other studies have identified the interval as 13 days (UK) [32], 14 days (New Zealand) [33] and 16 days (Germany) [34] and 7 days (Sweden) [35]. Reasons for discrepancies may reflect a difference in the time-points and data sources used to define the patient interval or changes over time in access to primary care.

Diagnostic intervals also vary; a UK study on patients diagnosed in 1999-2000 found a median diagnostic interval of 30 days [36], while other UK studies found diagnostic intervals of 14-27 days [37,38, 39] – with lower intervals in more recent studies, possibly reflecting the introduction of urgent referrals in 2010. A Danish study demonstrated an 18-day interval in 2010 [39], compared to 8 days in our study [39]. Similarly, a study in Manitoba in 2009-10 demonstrated an interval of 35 days (compared to 28 days in our study) [40]. The primary care interval has had less attention in the literature, although a Manitoban study reported an interval of 15 days (comparable with our finding of 17 days) [40]. It also reported a total interval of 78 days, which is comparable to the 76 days found in our study. A recent Swedish study focusing on the time from referral to treatment, which roughly corresponds to a combined diagnostic and treatment interval, reported a median interval of 20 days – compared to 35 days in our study.[35]

For peer review only

For peer review only - http://bmjopen.bmj.com/site/about/guidelines.xhtml

Clinical referral for a suspected breast cancer (e.g. lump) is often expedited [37,38]. Thus, the high proportion of women with 'red-flag' symptoms or signs may mean that time intervals for breast cancer diagnosis vary less than other common cancers, as differential diagnosis is easier and investigations and referral pathways more straightforward. Nevertheless, we found important variations in intervals for symptomatic women, possibly reflecting methodological differences – but also temporal and international differences in diagnostic pathways. We'd expect these differences to primarily impact on the median primary care interval (0 days for all jurisdictions except Manitoba, Ontario and Victoria). Note, however, that in these jurisdictions primary care is more often responsible for confirming the breast cancer diagnosis, thus extending the interval. Indeed, a study from Manitoba confirms that PCPs experience significant waiting time when they order a mammogram [40].

Conclusion and implications for clinical practice and healthcare organisation

Despite screening programmes, the diagnostic route for breast cancer remains critically important, as over half of patients are diagnosed this way. Awareness and recognition of warning signs impacts on patient intervals [41]; we found, in some jurisdictions, significant numbers of women waiting more than 90 days before presenting with symptoms. Targeted awareness interventions could facilitate more timely diagnosis of breast cancer. The variation in waiting times for an appointment in primary care, which might affect perceived access, could also be addressed.

The possible impact of variations in routes to diagnosis, diagnostic/treatment intervals and key outcomes (e.g. disease-free survival and mortality) cannot be assessed from our study. There is, nevertheless, growing evidence of an association between time intervals, the use of urgent referrals and mortality [10,41- 44]. More research is needed to explore this relationship.

This study illustrates the need to optimise diagnostic routes for breast cancer internationally. Awareness of international differences in key time intervals is an important step in optimising pathways. Ideally, routine

For peer review only

For peer review only - http://bmjopen.bmj.com/site/about/guidelines.xhtml

363 and standardised collection of time interval and route to diagnostic information will become the norm
364 internationally – this will greatly assist in optimisation of breast cancer diagnostic pathways.

For peer review only

For peer review only - http://bmjopen.bmj.com/site/about/guidelines.xhtml

List of abbreviations

ICBP M4 – International Cancer Benchmarking Partnership Module 4

PCP – primary care physician

TNM – tumour, node, metastasis

CTS – cancer treatment specialist

IARC – International Agency for Research on Cancer

Declarations

Ethics approval and consent to participate

For each local data collection, there were specific procedures and approvals. The approvals also included anonymised data transfer to University College London and Aarhus University. Approvals were received from the following institutions: Cancer Council Victoria Human Research Ethics Committee [HREC 1125]; Health Research Ethics Board, University of Manitoba [HS15227 (H2012:105)]; Research Resource Ethics Committee, CancerCare Manitoba [RRIC#28-2012]; University of Toronto Research Ethics Board [27881]; The Danish Data Protection Agency [2013-41-2030]; Swedish Ethics Review Board, Uppsala [2013/306]; Norway Regional committees for medical and health research ethics [2013/136/REK nord]; England, Wales and Scotland, NRES Committee East Midlands – Derby 2, local R&D for each health board [11/EM/0420]; Northern Ireland ORECNI Ethical approval, local governance for each health Trust [11/EM/0420].

Consent for publication

Not Applicable

Availability of data and material

The data that support the findings of this study are available from the named authors from each ICBP jurisdiction, but restrictions apply to the availability of these data and so are not publicly available. Data are however available from the authors upon reasonable request and with permission of the ICBP Programme

For peer review only

Board. Please contact the ICBP Programme Management team, based at Cancer Research UK, with any queries (icbp@cancer.org.uk).

Competing interests

The authors declare that they have no competing interests.

Funding

Funding for ICBP Module 4 was provided by: CancerCare Manitoba; Cancer Care Ontario; Canadian Partnership Against Cancer (CPAC); Cancer Council Victoria; Cancer Research Wales; Cancer Research UK; Danish Cancer Society; Danish Health and Medicines Authority; European Palliative Care Research Centre (PRC), Norwegian University of Science and Technology (NTNU); Guidelines Audit and Implementation Network (GAIN); Macmillan Cancer Support; National Cancer Action Team; NHS England; Northern Ireland Cancer Registry, funded by the Public Health Agency NI; Norwegian Directorate of Health; Research Centre for Cancer Diagnosis in Primary Care (CaP), Aarhus University, Denmark; Scottish Government; Swedish Association of Local Authorities and Regions; University College London and NIHR Biomedical Research Centre at University College London NHS Foundation Trust; University of Edinburgh (R42856); Victorian Department of Health and Human Services; Welsh Government.

The funding bodies had no influence on the design of the study and collection, analysis, and interpretation of data, in writing the manuscript or whether to publish the results.

Authors' contributions

- **Usha Menon** - planned the study design, data collection, carried out the analyses and wrote the draft manuscript. Also responsible for local data collection (alongside the Working Group), management and interpretation, and have participated in writing and approving the final manuscript version.

For peer review only - http://bmjopen.bmj.com/site/about/guidelines.xhtml

- **David Weller** - planned the study design, data collection, carried out the analyses and wrote the draft manuscript. Also responsible for local data collection (alongside the Working Group), management and interpretation, and have participated in writing and approving the final manuscript version.
- **Alina Zalounina Falborg** - planned the study design, data collection, carried out the analyses and wrote the draft manuscript
- Henry Jensen - planned the study design, data collection, carried out the analyses and wrote the draft manuscript. Also responsible for local data collection (alongside the Working Group), management and interpretation, and have participated in writing and approving the final manuscript version.
- **Peter Vedsted**- planned the study design, data collection, carried out the analyses and wrote the draft manuscript. Also responsible for local data collection (alongside the Working Group), management and interpretation, and have participated in writing and approving the final manuscript version.
- **Jatinderpal Kalsi** - Responsible for local data collection (alongside the ICBP Module 4Working Group), management and interpretation, and have participated in writing and approving the final manuscript version.
- **David H Brewster** - Responsible for local data collection (alongside the ICBP Module 4Working Group), management and interpretation, and have participated in writing and approving the final manuscript version.
- **Yulan Lin** - Responsible for local data collection (alongside the ICBP Module 4Working Group), management and interpretation, and have participated in writing and approving the final manuscript version.
- **Anna Gavin** - Responsible for local data collection (alongside the ICBP Module 4Working Group), management and interpretation, and have participated in writing and approving the final manuscript version.
- **Eva Grunfeld** - Responsible for local data collection (alongside the ICBP Module 4Working Group), management and interpretation, and have participated in writing and approving the final manuscript version.
- **Mats Lambe** - Responsible for local data collection (alongside the ICBP Module 4Working Group), management and interpretation, and have participated in writing and approving the final manuscript version.
- **Martin Malmberg** - Responsible for local data collection (alongside the ICBP Module 4Working Group), management and interpretation, and have participated in writing and approving the final manuscript version.
- **Donna Turner** - Responsible for local data collection (alongside the ICBP Module 4Working Group), management and interpretation, and have participated in writing and approving the final manuscript version.

For peer review only - http://bmjopen.bmj.com/site/about/guidelines.xhtml

- **Elizabeth Harland** - Responsible for local data collection (alongside the ICBP Module 4Working Group), management and interpretation, and have participated in writing and approving the final manuscript version.
- **Breann Hawryluk** - Responsible for local data collection (alongside the ICBP Module 4Working Group), management and interpretation, and have participated in writing and approving the final manuscript version.
- **Rebecca-Jane Law** - Responsible for local data collection (alongside the ICBP Module 4Working Group), management and interpretation, and have participated in writing and approving the final manuscript version.
- **Richard D Neal** - Responsible for local data collection (alongside the ICBP Module 4Working Group), management and interpretation, and have participated in writing and approving the final manuscript version.
- **Victoria White** - Responsible for local data collection (alongside the ICBP Module 4Working Group), management and interpretation, and have participated in writing and approving the final manuscript version.
- **Rebecca J Bergin** - Responsible for local data collection (alongside the ICBP Module 4Working Group), management and interpretation, and have participated in writing and approving the final manuscript version.
- **Samantha Harrison** - Responsible for local data collection (alongside the ICBP Module 4Working Group), management and interpretation, and have participated in writing and approving the final manuscript version.

Acknowledgements

We thank our colleagues and participating patients, primary care physicians and cancer treatment specialists in all participating ICBP jurisdictions. Catherine Foot, Martine Bomb, Brad Groves, Irene Reguilon, Charlotte Lynch and Samantha Harrison of Cancer Research UK for managing the programme. John Butler, Royal Marsden NHS Foundation Trust and ICBP Clinical Lead, for his advice. The ICBP M4 academic reference group for providing independent peer review and advice for the study protocol and analysis plan development. We would like to thank staff at the Information Services Division of NHS National Services Scotland for identifying potential study participants in Scotland, and mailing patient questionnaires to primary care physicians. The Danish Breast Cancer Group (DBCG) for providing secondary care (specialist) data to this study.

The ICBP Module 4 working Group:

For peer review only

- Alina Zalounina Falborg, Statistician, Research Unit for General Practice, Department of Public Health, Aarhus University, Bartholins Allé 2, 8000 Aarhus C, Denmark;
- Andriana Barisic, Research Associate, Department of Prevention and Cancer Control, Cancer Care Ontario, 620 University Avenue, Toronto, Ontario, M5G 2L7, Canada;
- Anna Gavin, Director, Northern Ireland Cancer Registry, Centre for Public Health, Queen's University Belfast, Mulhouse Building, Mulhouse Road, Belfast, BT12 6DP, United Kingdom;
- Anne Kari Knudsen, Administrative leader, Department of Cancer Research and Molecular Medicine, Norwegian University of Science and Technology, 7489 Trondheim, Norway;
- Breann Hawryluk, Project Planning Coordinator, Department of Patient Navigation, Cancer Care Manitoba, 675 McDermot Street, Winnipeg, Manitoba, Canada;
- Chantelle Anandan, Research Fellow, Centre for Population Health Sciences, University of Edinburgh, Doorway 1, Medical Quad Teviot Place, Edinburgh, EH8 9DX, United Kingdom;
- Conan Donnelly, Research Fellow, Centre for Public Health, Queen's University Belfast, Mulhouse Building, Mulhouse Road, Belfast, BT12 6DP, United Kingdom;
- David H Brewster, Scottish Cancer Registry, Information Services Division, NHS National Services Scotland, Gyle Square, 1 South Gyle Crescent, Edinburgh, EH12 9EB, United Kingdom;
- David Weller, James Mackenzie Professor of General Practice, Centre for Population Health Sciences, University of Edinburgh, Doorway 1, Medical Quad Teviot Place, Edinburgh, EH8 9DX, United Kingdom;
- Donna Turner, Epidemiologist/Provincial Director, Population Oncology, Cancer Care Manitoba, 675 McDermot Street, Winnipeg, Manitoba, Canada;
- Elizabeth Harland, Project Coordinator, Department of Epidemiology and Cancer Registry, CancerCare Manitoba, 675 McDermot Street, Winnipeg, Manitoba;
- Eva Grunfeld, Director, Knowledge Translation Research Network Health Services Research Program, Ontario Institute for Cancer Research; Professor and Vice Chair Research, Department of Family and Community Medicine, University of Toronto, 500 University Avenue, Toronto, Ontario, M5G 1V7, Canada;
- Evangelia Ourania Fourkala, Research Associate, Gynaecological Cancer Research Centre, Women's Cancer, Institute for Women's Health, University College London, United Kingdom;
- Henry Jensen, Research fellow, Research Unit for General Practice, Department of Public Health, Aarhus University, Bartholins Allé 2, 8000 Aarhus C, Denmark;
- Jackie Boylan, Research Fellow, Centre for Public Health, Queen's University Belfast, Mulhouse Building, Mulhouse Road, Belfast, BT12 6DP, United Kingdom;
- Jacqueline Kelly, Tumour Verification Officer, Northern Ireland Cancer Registry, Centre for Public Health, Queen’s University Belfast, Mulhouse Building, Mulhouse Road, Belfast, BT12 6DP, United Kingdom;
- Kerry Moore, Research Fellow, Centre for Public Health, Queen's University Belfast, Mulhouse Building, Mulhouse Road, Belfast, BT12 6DP, United Kingdom;
- Maria Rejmyr Davis, Head, Southern Sweden Regional Cancer Center, Medicon Village, Scheelevägen 8, building 404, 223 81 Lund, Sweden;
- Martin Malmberg, MD PhD, Senior Consultant, Department of Oncology, Lund University Hospital, SE-221 85 Lund, Sweden;
- Mats Lambe, Professor of Medical Epidemiology, Regional Cancer Center Uppsala and Department of Medical Epidemiology and Biostatics, Karolinska Institutet, SE-171 77 Stockholm, Sweden;
- Oliver Bucher, Epidemiologist, Department of Epidemiology and Cancer Registry, CancerCare Manitoba, 675 McDermot Street, Winnipeg, Manitoba;

For peer review only

For peer review only - http://bmjopen.bmj.com/site/about/guidelines.xhtml

- Peter Vedsted, Professor, Research Unit for General Practice, Department of Public Health, Aarhus University, Bartholins Allé 2, 8000 Aarhus C, Denmark;
- Rebecca Bergin, Senior Research Officer/ PhD Candidate, Centre for Behavioural Research in Cancer, 615 St Kilda Rd, Melbourne, Victoria, 3004, Australia;
- Rebecca-Jane Law, Research Project Support Officer, North Wales Centre for Primary Care Research, Bangor University, Gwenfro Units 4-8, Wrexham Technology Park, Wrexham, LL13 7YP, United Kingdom;
- Richard D Neal, Professor of Primary Care Oncology, Academic Unit of Primary Care, Leeds Institute of Health Sciences, University of Leeds, Leeds LS2 9NL, United Kingdom;
- Sigrun Saur Almberg, Researcher, Department of Cancer Research and Molecular Medicine, Faculty of Medicine, Norwegian University of Science and Technology (NTNU), N-7491 Trondheim, Norway;
- Therese Kearney, Research fellow, Northern Ireland Cancer Registry, Centre for Public Health, Queen's University Belfast, Mulhouse Building, Mulhouse Road, Belfast, BT12 6DP, United Kingdom;
- Jatinderpal Kalsi, Project Manager, Gynaecological Cancer Research Centre, Women's Cancer, Institute for Women's Health, University College London, United Kingdom;
- Victoria Cairnduff, Statistician, Northern Ireland Cancer Registry, Centre for Public Health, Queen's University Belfast, Mulhouse Building, Mulhouse Road, Belfast, BT12 6DP, United Kingdom;
- Victoria Hammersley, Researcher, Centre for Population Health Sciences, University of Edinburgh, Doorway 1, Medical Quad Teviot Place, Edinburgh, EH8 9DX, United Kingdom;
- Victoria White, Deputy Director, Centre for Behavioral Research in Cancer, Cancer Council Victoria, 615 St Kilda Road, Melbourne, Victoria, 3004, Australia;
- Usha Menon, Professor of Gynaecological Oncology and Head, Gynaecological Cancer Research Centre, Women's Cancer, Institute for Women's Health, University College London, United Kingdom;
- Yulan Lin, Postdoc, Department of Cancer Research and Molecular Medicine, Faculty of Medicine, Norwegian University of Science and Technology (NTNU), N-7491 Trondheim, Norway

ICBP Module 4 Academic Reference Group:

- Prof Jan Willem Coebergh, Professor of Cancer Surveillance, Department of Public Health, Erasmus Universiteit Rotterdam, Rotterdam, the Netherlands;
- Jon Emery, Professor of Primary Care Cancer Research, University of Melbourne and Clinical Professor of General Practice, University of Western Australia, Australia;
- Dr Stefan Bergström, Senior consultant oncologist, Department of Oncology, Gävle, Sweden;
- Dr Monique E van Leerdam, Erasmus MC University Medical Centre, the Netherlands;
- Prof Marie-Louise Essink-Bot, Academic Medical Centre, Amsterdam University, the Netherlands;
- Prof Una MacLeod, Senior Lecturer in General Practice and Primary Care, Hull-York Medical School, United Kingdom.

For peer review only - http://bmjopen.bmj.com/site/about/guidelines.xhtml

References

1. Global Burden of Disease Cancer C, Fitzmaurice C, Dicker D, Pain A, Hamavid H, Moradi-Lakeh M, MacIntyre MF, Allen C, Hansen G, Woodbrook R, et al: The Global Burden of Cancer 2013. JAMA Oncol 2015, 1:505-527.

2. Allemani C, Matsuda T, Di Carlo V, Harewood R, Matz M, Nikšić M, Bonaventure A, Valkov M, Johnson CJ, Estève J, et al: Global surveillance of trends in cancer survival 2000-14 (CONCORD-3): analysis of individual records for 37 513 025 patients diagnosed with one of 18 cancers from 322 population-based registries in 71 countries. Lancet 2018, S0140-6736(17)33326-3. [Epub ahead of print].

3. Walters S, Maringe C, Butler J, Rachet B, Barrett-Lee P, Bergh J, Boyages J, Christiansen P, Lee M, Warnberg F, et al: Breast cancer survival and stage at diagnosis in Australia, Canada, Denmark, Norway, Sweden and the UK, 2000-2007: a population-based study. Br J Cancer 2013, 108:1195-1208.

4. Coleman MP, Forman D, Bryant H, Butler J, Rachet B, Maringe C, Nur U, Tracey E, Coory M, Hatcher J, et al: Cancer survival in Australia, Canada, Denmark, Norway, Sweden, and the UK, 1995-2007 (the International Cancer Benchmarking Partnership): an analysis of population-based cancer registry data. Lancet 2011, 377:127-138.

5. Niksic M, Rachet B, Duffy SW, Quaresma M, Moller H, Forbes LJ: Is cancer survival associated with cancer symptom awareness and barriers to seeking medical help in England? An ecological study. Br J Cancer 2016, 115:876-886.

6. Yun YH, Kim YA, Min YH, Park S, Won YJ, Kim DY, Choi IJ, Kim YW, Park SJ, Kim JH, et al: The influence of hospital volume and surgical treatment delay on long-term survival after cancer surgery. Ann Oncol 2012, 23:2731-2737.

7. Smith EC, Ziogas A, Anton-Culver H. Delay in surgical treatment and survival after breast cancer diagnosis in young women by race/ethnicity. JAMA Surg. 2013;148:516-23.

8. Torring ML, Frydenberg M, Hamilton W, Hansen RP, Lautrup MD, Vedsted P: Diagnostic interval and mortality in colorectal cancer: U-shaped association demonstrated for three different datasets. J Clin Epidemiol 2012, 65:669-678.

9. Torring ML, Frydenberg M, Hansen RP, Olesen F, Vedsted P: Evidence of increasing mortality with longer diagnostic intervals for five common cancers: a cohort study in primary care. Eur J Cancer 2013, 49:2187-2198.

10. Neal RD, Tharmanathan P, France B, Din NU, Cotton S, Fallon-Ferguson J, Hamilton W, Hendry A, Hendry M, Lewis R, et al: Is increased time to diagnosis and treatment in symptomatic cancer associated with poorer outcomes? Systematic review. Br J Cancer 2015, 112 Suppl 1:S92-107.

11. Forbes LJ, Simon AE, Warburton F, Boniface D, Brain KE, Dessaix A, Donnelly C, Haynes K, Hvidberg L, Lagerlund M, et al: Differences in cancer awareness and beliefs between Australia, Canada, Denmark, Norway, Sweden and the UK (the International Cancer Benchmarking Partnership): do they contribute to differences in cancer survival? Br J Cancer 2013, 108:292-300.

12. Koo MM, von Wagner C, Abel GA, McPhail S, Rubin GP, Lyratzopoulos G: Typical and atypical presenting symptoms of breast cancer and their associations with diagnostic intervals: Evidence from a national audit of cancer diagnosis. Cancer Epidemiol 2017, 48:140-146.

For peer review only - http://bmjopen.bmj.com/site/about/guidelines.xhtml

13. Brown S, Castelli M, Hunter DJ, Erskine J, Vedsted P, Foot C, Rubin G: How might healthcare systems influence speed of cancer diagnosis: a narrative review. Soc Sci Med 2014, 116:56-63.

14. Lyratzopoulos G, Vedsted P, Singh H: Understanding missed opportunities for more timely diagnosis of cancer in symptomatic patients after presentation. Br J Cancer 2015, 112 Suppl 1:S84-91.

15. Rubin G, Berendsen A, Crawford SM, Dommett R, Earle C, Emery J, Fahey T, Grassi L, Grunfeld E, Gupta S, et al: The expanding role of primary care in cancer control. Lancet Oncol 2015, 16:1231-1272.

16. Rose PW, Rubin G, Perera-Salazar R, Almberg SS, Barisic A, Dawes M, Grunfeld E, Hart N, Neal RD, Pirotta M, et al: Explaining variation in cancer survival between 11 jurisdictions in the International Cancer Benchmarking Partnership: a primary care vignette survey. BMJ Open 2015, 5:e007212.

17. National Institute for Health and Care Excellence. Suspected cancer: recognition and referral; NICE Guideline [NG12]. National Institute for Health Care Excellence, 2015. Available at: https://www.nice.org.uk/guidance/ng12 [accessed 05 October 2017]

18. Murchie P, Campbell NC, Delaney EK, Dinant GJ, Hannaford PC, Johansson L, Lee AJ, Rollano P, Spigt M: Comparing diagnostic delay in cancer: a cross-sectional study in three European countries with primary care-led health care systems. Fam Pract 2012, 29:69-78.

19. Butler J, Foot C, Bomb M, Hiom S, Coleman M, Bryant H, Vedsted P, Hanson J, Richards M, Group IW: The International Cancer Benchmarking Partnership: an international collaboration to inform cancer policy in Australia, Canada, Denmark, Norway, Sweden and the United Kingdom. Health Policy 2013, 112:148-155.

20. Weller D, Vedsted P, Anandan C, Zalounina A, Fourkala EO, Desai R, Liston W, Jensen H, Barisic A, Gavin A, et al: An investigation of routes to cancer diagnosis in 10 international jurisdictions, as part of the International Cancer Benchmarking Partnership: survey development and implementation. BMJ Open 2016, 6:e009641.

21. European Network of Cancer Registries. Standards and guidelines for cancer registration in Europe: the ENCR recommendations. Edited by Tyczynski JE, Démaret E, Parkin DM. (IARC technical publication; no. 40), Lyon, 2003.

22. TNM classification of malignant tumours. Eds. L.H. Sobin, M.K. Gospodarowicz and Ch. Wittekind. — 7th ed. UICC International Union Against Cancer. ISBN 978-1-4443-3241-4

23. Weller D, Vedsted P, Rubin G, Walter FM, Emery J, Scott S, Campbell C, Andersen RS, Hamilton W, Olesen F, et al: The Aarhus statement: improving design and reporting of studies on early cancer diagnosis. Br J Cancer 2012, 106:1262-1267.

24. Macefield RC, Jacobs M, Korfage IJ, Nicklin J, Whistance RN, Brookes ST, et al. Developing core outcomes sets: methods for identifying and including patient-reported outcomes (PROs). Trials. 2014 Feb 5;15:49.

25. Danish Cancer Society. Suspicion of breast cancer criteria for referral for general practice. Danish Cancer Society, 2016. Available at: https://www.sst.dk/da/udgivelser/2016/~/media/4864545DA763400E9FD988FA4DA74EE5.ashx accessed 05 October 2017

26. Koenker R, Bassett G. Regression quantiles. *Econometrica* 1978; 46:33-50

For peer review only - http://bmjopen.bmj.com/site/about/guidelines.xhtml

27. Machado JAF, Santos Silva JMC. Quantiles for Counts. J Amer Statistical Assoc 2005, 100(472): 1226-1237.

28. Miranda A. QCOUNT: Stata program to fit quantile regression models for count data. Boston: Boston College Department of Economics; 2006

29. Lin LI. A concordance correlation coefficient to evaluate reproducibility. Biometrics 1989;45((1) March):255–68

30. Hansen RP, Vedsted P, Sokolowski I, Sondergaard J, Olesen F: Time intervals from first symptom to treatment of cancer: a cohort study of 2,212 newly diagnosed cancer patients. BMC Health Serv Res 2011, 11:284.

31. Olesen F, Hansen RP, Vedsted P: Delay in diagnosis: the experience in Denmark. Br J Cancer 2009, 101 Suppl 2:S5-8.

32. Nosarti C, Crayford T, Roberts JV, Elias E, McKenzie K, David AS: Delay in presentation of symptomatic referrals to a breast clinic: patient and system factors. Br J Cancer 2000, 82:742-748.

33. Meechan G, Collins J, Petrie K: Delay in seeking medical care for self-detected breast symptoms in New Zealand women. N Z Med J 2002, 115:U257.

34. Arndt V, Sturmer T, Stegmaier C, Ziegler H, Dhom G, Brenner H: Patient delay and stage of diagnosis among breast cancer patients in Germany -- a population based study. Br J Cancer 2002, 86:1034-1040.

35. Thulesius HO, Lindgren AC, Olsson HL, Hakansson A: Diagnosis and prognosis of breast and ovarian cancer--a population-based study of 234 women. Acta Oncol 2004, 43:175-181.

36. Allgar VL, Neal RD: Delays in the diagnosis of six cancers: analysis of data from the National Survey of NHS Patients: Cancer. Br J Cancer 2005, 92:1959-1970.

37. Din NU, Ukoumunne OC, Rubin G, Hamilton W, Carter B, Stapley S, Neal RD: Age and Gender Variations in Cancer Diagnostic Intervals in 15 Cancers: Analysis of Data from the UK Clinical Practice Research Datalink. PLoS One 2015, 10:e0127717.

38. Redaniel MT, Martin RM, Ridd MJ, Wade J, Jeffreys M: Diagnostic intervals and its association with breast, prostate, lung and colorectal cancer survival in England: historical cohort study using the Clinical Practice Research Datalink. PLoS One 2015, 10:e0126608.

39. Jensen H, Torring ML, Olesen F, Overgaard J, Fenger-Gron M, Vedsted P: Diagnostic intervals before and after implementation of cancer patient pathways - a GP survey and registry based comparison of three cohorts of cancer patients. BMC Cancer 2015, 15:308.

40. Nashed M, Carpenter-Kellett T, Lambert P, Musto G, Turner D, Cooke A: Wait Time from Suspicion to Surgery for Breast Cancer in Manitoba. Cureus 2016, 8:e680.

41. Quaife SL, Forbes LJ, Ramirez AJ, Brain KE, Donnelly C, Simon AE, Wardle J: Recognition of cancer warning signs and anticipated delay in help-seeking in a population sample of adults in the UK. Br J Cancer 2014, 110:12-18.

42. Richards MA, Westcombe AM, Love SB, Littlejohns P, Ramirez AJ. Influence of delay on survival in patients with breast cancer: a systematic review. Lancet. 1999 Apr 3;353(9159):1119-26.

For peer review only

43. Moller H, Gildea C, Meechan D, Rubin G, Round T, Vedsted P. Use of the English urgent referral pathway for suspected cancer and mortality in patients with cancer: cohort study. BMJ. 2015 Oct 13;351:h5102. doi: 10.1136/bmj.h5102.

44. Murchie P, Raja EA, Lee AJ, Brewster DH, Campbell NC, Gray NM, Ritchie LD, Robertson R, Samuel L: Effect of longer health service provider delays on stage at diagnosis and mortality in symptomatic breast cancer. Breast 2015, 24:248-255.

For peer review only

For peer review only - http://bmjopen.bmj.com/site/about/guidelines.xhtml

For peer review only

For peer review only - http://bmjopen.bmj.com/site/about/guidelines.xhtml

Table 1. *The characteristics of the 3,471 women (40+) with a first diagnosis of breast cancer (% if nothing else stated)*

	Wales (N=232)	England (N=363)	Scotland (N=371)	N Ireland (N=331)	Denmark (N=369)	Ontario (N=403)	Norway (N=372)	Manitoba (N=368)	Victoria (N=370)	Sweden (N=292)	Total (N=3471)
**Age** years											
Median (IQI)	63 (53,70)	63 (53,70)	63 (54,70)	60 (51,70)	64 (55,71)	63 (55,69)	61 (52, 69)	63 (55,72)	58 (50,67)	63 (54,71)	62 (53,70)
**Age** years (Symptomatic women)	(N=115)	(N=207)	(N=208)	(N=242)	(N=231)	(N=201)	(N=235)	(N=205)	(N=198)	(N=120)	(N=1962)
Median (IQI)	62 (49,76)	62 (50,75)	62 (52,75)	60 (49,72)	68 (54,77)	61 (53,69)	61 (50, 73)	62 (50,75)	56 (48,68)	68 (54,77)	62 (50,74)
**Age** years (Screen-detected women	(N=116)	(N=155)	(N=162)	(N=86)	(N=134)	(N=198)	(N=132)	(N=162)	(N=172)	(N=171)	(N=1488)
Median (IQI)	63 (58,68)	64 (58,68)	63 (58,67)	60 (54,67)	62 (56,66)	65 (59,69)	62 (57,67)	65 (59,70)	61 (53,67)	61 (54,69)	63 (57,68)
**Health State**											
Good	81.0	81.0	79.3	77.7	78.1	90.3	74.7	85.6	88.4	80.8	81.8
Fair	14.7	13.0	13.7	14.2	14.6	8.2	17.5	9.2	9.5	12.3	12.6
Poor	3.5	4.7	5.1	5.4	3.5	1	6.2	2.7	1.6	3.8	3.7
Missing	0.9	1.3	1.9	2.7	3.8	0.5	1.6	2.5	0.5	3.1	1.9
**Comorbidity**[1]											
No	74.1	75.1	69.0	78.6	62.9	61.3	73.9	70.4	76.5	80.5	71.8
Medium	24.6	23.1	29.4	19.9	34.1	25.1	23.7	27.7	23.2	16.1	25.0
High	0.9	0.8	1.1	0.9	0.8	0.0	1.1	0.5	0.0	0.3	0.6
Missing	0.4	0.8	0.5	0.6	2.2	13.6	1.3	1.4	0.3	3.1	2.6
**Education**											
Low	71.6	78.2	69.0	77.0	71.8	64.3	63.7	74.7	70.0	68.8	70.8
High	22.4	14.9	19.7	16.3	14.6	33.5	31.5	19.8	28.9	28.8	23.1
Missing	6.0	6.9	11.3	6.7	13.6	2.2	4.8	5.4	1.1	2.4	6.1
**Ethnicity**											
White	97.9	97.8	98.9	99.1	94.3	89.9	98.7	85.6	97.3	96.2	95.4
Asian	0.4	1.1	0.0	0.0	1.1	5.7	0.8	7.6	1.9	1.0	2.1
Black	0.4	0.8	0.3	0.0	0.0	2.0	0.0	0.5	0.0	0.0	0.4
Other	0.0	0.0	0.0	0.0	0.0	1.2	0.0	6.0	0.0	0.0	0.8
Missing	1.3	0.3	0.8	0.9	4.6	1.2	0.5	0.3	0.8	2.8	1.3
**Smoking**											
Currently	7.3	7.2	8.4	11.8	15.2	4.0	15.1	7.9	5.7	19.6	9.2
In the past	35.3	38.0	41.2	33.2	41.7	43.9	40.9	44.6	36.7	41.1	39.9
Never	56.5	54.5	49.3	53.5	40.9	51.6	43.5	46.2	57.3	46.9	49.8
Missing	0.9	0.3	1.1	1.5	2.2	0.5	0.5	1.3	0.3	2.4	1.1
**Tumour stage – TNM** (all women)											
0	1.7	0.6	0.0	0.0	0.3	0.3	0.0	0.0	0.3	0.0	0.3
I	50.4	44.9	47.4	45.3	45.0	51.6	11.3	50.8	52.7	62.0	45.7
II	37.9	36.4	35.6	35.1	33.1	38.0	4.6	34.5	34.6	31.5	31.9
III	5.6	8.3	7.8	13.6	9.5	8.2	1.9	10.3	10.8	4.1	8.1
IV	0.9	3.0	4.6	2.7	2.4	1.0	0.8	1.6	1.1	0.7	1.9
Missing	3.5	6.9	4.5	3.3	9.8	1.0	81.5	2.7	0.5	1.7	12.1

For peer review only - http://bmjopen.bmj.com/site/about/guidelines.xhtml

	Wales (N=232)	England (N=363)	Scotland (N=371)	N Ireland (N=331)	Denmark (N=369)	Ontario (N=403)	Norway (N=372)	Manitoba (N=368)	Victoria (N=370)	Sweden (N=292)	Total (N=3471)
**Tumour stage – TNM** (Symptomatic women)	(N=115)	(N=207)	(N=208)	(N=242)	(N=231)	(N=201)	(N=235)	(N=205)	(N=198)	(N=120)	(N=1966)
0	1.7	1.0	0.0	0.0	0.0	0.0	0.0	0.0	0.5	0.0	0.3
I	33.0	34.8	32.7	37.2	36.4	32.8	8.1	32.7	37.9	40.8	32.1
II	49.6	41.6	45.7	39.3	35.1	50.0	5.5	48.3	44.4	50.8	39.5
III	8.7	10.6	9.6	17.4	11.7	14.0	2.6	13.2	15.2	3.3	11.0
IV	1.7	4.4	6.7	2.9	3.9	2.0	0.9	2.4	1.0	1.7	2.9
Missing	5.2	7.7	5.3	3.3	13.0	0.5	83.0	3.4	1.0	3.3	14.3
**Tumour stage – TNM** (Screen-detected women)	(N=116)	(N=155)	(N=162)	(N=86)	(N=134)	(N=198)	(N=132)	(N=162)	(N=172)	(N=171)	(N=1484)
0	1.7	0.0	0.0	0.0	0.8	0.5	0.0	0.0	0.0	0.0	0.3
I	68.1	58.1	66.1	67.4	59.7	69.2	16.7	74.1	69.8	77.2	63.5
II	25.9	29.7	22.8	23.3	29.1	26.3	2.3	17.3	23.3	18.1	21.9
III	2.6	5.2	5.6	3.5	6.0	2.5	0.8	6.8	5.8	4.1	4.4
IV	0.0	1.3	1.9	2.3	0.0	0.0	0.0	0.6	1.2	0.0	0.7
Missing	1.7	5.8	3.7	3.5	4.5	1.5	80.3	1.2	0.0	0.6	9.3

[1] Comorbidity coded as none=no reported, medium=1-2 reported and high=3+ reported

Abbreviations: IQI=inter-quartile interval

Norwegian data on tumour stage were incomplete, resulting in a high number of missing stage data.

For peer review only

For peer review only - http://bmjopen.bmj.com/site/about/guidelines.xhtml

Table 2. Proportion of screen-detected or symptomatic patients (%), and place of initial symptomatic presentation across jurisdictions

	Wales (N=232)	England (N=363)	Scotland (N=371)	N Ireland[1] (N=331)	Denmark (N=369)	Ontario (N=403)	Norway (N=373)	Manitoba (N=368)	Victoria (N=370)	Sweden (N=292)	Total (N=3471)
**Symptomatic**	**49.6**	**57.0**	**56.1**	**73.1**	**62.6**	**49.9**	**63.2**	**55.7**	**53.5**	**41.1**	**56.5**
Visit PCP, Visit PCP and then A&E[2]	*91.3*	*93.7*	*92.3*	*88.0*	*84.4*	*69.2*	*81.3*	*88.3*	*90.9*	*75.0*	*85.6*
A&E directly[2]	*1.7*	*1.0*	*0.5*	*0.4*	*1.3*	*2.5*	*0.9*	*1.0*	*0.5*	*0.8*	*1.0*
Investigation for another problem[2]	*0.9*	*1.0*	*4.8*	*1.2*	*6.1*	*7.5*	*4.7*	*2.4*	*1.5*	*10.0*	*3.9*
Other[2]	*6.1*	*4.3*	*2.4*	*10.3*	*8.2*	*20.9*	*13.2*	*8.3*	*7.1*	*14.2*	*9.5*
**Screening**	**50.0**	**42.7**	**43.7**	**26.0**	**36.3**	**49.1**	**35.5**	**44.0**	**46.5**	**58.6**	**42.9**
**Other**	**0.4**	**0.3**	**0.3**	**0.9**	**1.1**	**1.0**	**1.2**	**0.3**	**0.0**	**0.3**	**0.6**

[1] In N Ireland the proportion of screen detected breast cancers was lower as many of these women were excluded from the eligible group in the start of the inclusion

[2] Percentage of the symptomatic route

For peer review only

Table 3. *The first symptom(s) reported by women[1] and the first presenting symptom(s) reported by PCPs (%)*

	**Wales**	**England**	**Scotland**	**N Ireland[1]**	**Denmark**	**Ontario**	**Norway**	**Manitoba**	**Victoria**	**Sweden[2]**	**Total**
First symptom *(reported by women)*	*(N=115)*	*(N=207)*	*(N=208)*	*(N=242)*	*(N=231)*	*(N=201)*	*(N=235)*	*(N=205)*	*(N=198)*	*(N=120)*	*(N=1962)*
Lump/swelling/thickening of breast	76	70	75	62	63	63	55	60	71	73	66
Change in the nipple	9	15	11	14	11	10	13	6	6	14	11
Fatigue	16	10	9	12	5	17	11	12	14	9	11
Change in the size, contour	12	13	10	10	5	16	8	10	15	11	11
Pain/tenderness in the breast	6	5	4	14	3	5	7	9	15	6	8
Flattening/indentation in the breast	5	5	0.5	4	4	0	2	2	0	0.8	2
Other	21	23	20	17	10	13	18	19	16	12	17
No symptoms	8	2	4	8	11	10	9	17	5	10	8
Missing	0	0.4	4	6	10	2	13	4	1	0	5
Presenting symptom *(reported by PCP)*	*(N=97)*	*(N=169)*	*(N=160)*	*(N=0)*	*(N=153)*	*(N=74)*	*(N=109)*	*(N=123)*	*(N=128)*	*(N=0)*	*(N=1013)*
Lump/swelling/thickening of breast	73	64	64	n/a	67	34	55	46	64	n/a	60
Change in the nipple	2	8	5	n/a	5	7	5	0.8	6	n/a	5
Fatigue	0	0	0	n/a	0	0	0.9	0.8	0	n/a	0.2
Change in the size, contour	4	4	0	n/a	6	0	0.9	3	5	n/a	3
Pain/tenderness in the breast	10	6	4	n/a	7	7	4	10	13	n/a	7
Flattening/indentation in the breast	2	2	1	n/a	0	3	2	3	0	n/a	2
Other	13	12	9	n/a	7	24	13	13	2	n/a	11
No symptoms	1	0.6	0.6	n/a	0.7	11	0	12	2	n/a	3
Missing	8	11	17	n/a	18	18	21	24	20	n/a	17
Cancer-specificity of presenting symptom[3]											
Cancer-specific symptom	88	85	79	n/a	78	69	70	59	78	n/a	77
Non-specific symptom	3	3	4	n/a	3	3	9	4	0	n/a	4
No symptoms / Missing	9	12	18	n/a	18	28	21	37	22	n/a	20

[1] *These figures represent only women who had a symptom-based cancer diagnosis. More than one symptom could be stated (sum over 100%)*
[2] *Sweden and Northern Ireland did not include PCP questionnaire data*
[3] *Based on the PCP reported symptom/sign and categorised by two researchers*

For peer review only - http://bmjopen.bmj.com/site/about/guidelines.xhtml

Table 4. *Time intervals (days) for each jurisdiction depicted as median, 75th and 90th percentiles.*

		Wales	England	Scotland	N Ireland	Denmark	Ontario	Norway	Manitoba	Victoria	Sweden[1]
Patient Interval (Symptomatic)	Number	111	201	198	219	213	170	206	172	189	101
	Median	11	7	7	8	3	19	12	14	7	29
	75th percentile	34	30	30	31	22	58	48	47	31	56
	90th percentile	73	92	88	114	63	142	157	86	117	90
Primary Care interval (Symptomatic)	Number	97	167	160	172	141	70	99	109	142	n/a
	Median	0	0	0	0	0	20	0	17	7	
	75th percentile	0	0	1	0	0	37	0	30	15	
	90th percentile	3	7	6	3	10	75	14	82	38	
Diagnostic interval (Symptomatic)	Number	111	200	197	216	207	166	203	184	191	101
	Median	29	12	19	14	8	25	20	28	13	13
	75th percentile	54	18	35	21	26	56	37	42	21	24
	90th percentile	92	36	49	49	49	202	71	79	46	48
Diagnostic interval (Screen-detected)	Number	113	153	159	86	131	184	126	125	152	158
	Median	25	15	20	19	24	26	27	23	21	15
	75th percentile	45	21	29	26	36	42	44	43	32	25
	90th percentile	58	34	42	41	48	66	66	113	40	41
Diagnostic interval (All)	Number	224	353	356	302	338	350	329	309	343	259
	Median	29	13	20	15	14	26	22	26	16	14
	75th percentile	49	21	33	21	31	46	39	42	28	25
	90th percentile	70	36	48	43	49	90	68	94	45	41
Treatment interval (Symptomatic)	Number	115	203	202	241	230	201	216	198	196	113
	Median	24	29	24	21	20	35	19	38	14	22
	75th percentile	33	41	37	29	29	49	27	55	24	29
	90th percentile	45	55	54	40	44	65	34	72	33	42
Treatment interval (Screen-detected)	Number	115	155	158	86	134	195	120	155	170	165
	Median	27	31	34	24	19	35	13	39	19	21
	75th percentile	36	40	44	35	29	48	21	51	28	29
	90th percentile	49	62	69	44	36	65	28	66	38	40
Treatment interval (All)	Number	231	359	361	330	368	400	341	353	366	278
	Median	25	30	29	22	20	35	15	39	15	22
	75th percentile	35	41	41	31	29	48	24	54	27	29
	90th percentile	46	57	61	41	41	65	33	71	36	41
Total interval (Symptomatic)	Number	104	190	186	205	189	173	184	155	177	98
	Median	70	57	58	50	42	92	54	92	42	71
	75th percentile	96	82	99	78	73	158	121	128	89	101

For peer review only

For peer review only - http://bmjopen.bmj.com/site/about/guidelines.xhtml

	90th percentile	218	138	149	147	170	273	231	188	170	169
Total interval (All)	Number	217	343	341	291	320	354	298	277	327	253
	Median	60	52	55	46	44	78	48	76	42	42
	75th percentile	81	70	84	69	68	116	79	116	63	68
	90th percentile	123	114	129	127	118	209	168	182	120	101

[1] In Sweden, data on the primary care interval were not collected.

For peer review only

For peer review only - http://bmjopen.bmj.com/site/about/guidelines.xhtml

Table 5. *The prevalence ratios[1] (PRs) of experiencing differing primary care intervals for symptomatic breast cancer patients.*

	Wales (N=97)	England (N=167)	Scotland (N=160)	N Ireland (N=172)	Denmark (N=141)	Ontario (N=70)	Norway (N=99)	Manitoba (N=109)	Victoria (N=142)	Sweden[2] (N=0)
Interval ≥ 5 days										
PR (95% CI)	ref.	1.7 (0.7-4.2)	2.0 (0.8-4.9)	1.3 (0.5-3.3)	2.0 (0.8-4.8)	**12.9** (5.9-28.2)	2.2 (0.9-5.6)	**12.9** (5.9-28.1)	**11.1** (5.1-24.4)	n/a
Interval ≥ 14 days										
PR (95% CI)	ref.	1.6 (0.5-4.9)	1.5 (0.5-4.7)	1.4 (0.5-4.3)	2.1 (0.7-6.3)	**15.1** (5.7-40.1)	2.4 (0.8-7.4)	**14.3** (5.4-37.8)	**6.7** (2.5-18.1)	n/a

Bold: significance level of p ≤0.05; n/a=not applicable

[1] Adjusted for differences in age and comorbidity among the patients, with Wales as reference.

[2] In Sweden, no information on primary care interval was collected.

For peer review only

For peer review only - http://bmjopen.bmj.com/site/about/guidelines.xhtml

Table 6. *The differences in interval lengths (days) between Wales, the reference, and the other nine jurisdictions.*

		Wales	England	Scotland	N Ireland	Denmark	Ontario	Norway	Manitoba	Victoria	Sweden
**Patient Interval (Symptomatic)**	Number	111	201	198	219	213	170	206	172	189	101
	Median	11 (ref.)	-3(-9,3)	-3 (-8,3)	-1 (-8,6)	**-7** (-12,-2)	7 (-1,16)	1 (-7,9)	4 (-2,11)	-4 (-9,2)	**17** (11,22)
	75th percentile	34 (ref.)	-7 (-21,7)	-4 (-12,3)	-2 (-8,5)	**-10** (-16,-4)	**19** (5,33)	**16** (7,25)	12 (-1,25)	-6 (-16,4)	**22** (8,36)
	90th percentile	73 (ref.)	21 (-19,61)	11 (-45,66)	30 (-13,73)	-14 (-64,35)	50 (-9,110)	**74** (31,117)	9 (-35,54)	33 (-13,80)	14 (-31,60)
**Diagnostic interval (Symptomatic)**	Number	111	200	197	216	207	166	203	184	191	101
	Median	29 (ref.)	**-18** (-22,-15)	**-10** (-14,-6)	**-16** (-19,-13)	**-22** (-25,-18)	**-6** (-9,-2)	**-10** (-15,-5)	-1 (-5,4)	**-18** (-22,-14)	**-17** (-23,-12)
	75th percentile	54 (ref.)	**-39** (-45,-33)	**-24** (-29,-19)	**-37** (-42,-33)	**-34** (-40,-28)	-3 (-13,6)	**-21** (-36,-6)	**-16** (-23,-8)	**-34** (-52,-15)	**-33** (-39,-27)
	90th percentile	92 (ref.)	**-68** (-88,-48)	**-58** (-91,-25)	**-62** (-80,-45)	**-55** (-75,-35)	**73** (35,112)	**-35** (-62,-8)	**-30** (-46,-15)	**-63** (-78,-47)	**-58** (-81,-35)
**Diagnostic interval (Screen-detected)**	Number	113	153	159	86	131	184	126	125	152	158
	Median	25 (ref.)	-11 (-22,1)	-7 (-18,5)	-7 (-19,4)	-1 (-17,15)	0 (-12,12)	1 (-11,13)	-2 (-15,10)	-5 (-17,6)	-12 (-24,0)
	75th percentile	45 (ref.)	**-24** (-36,-13)	**-17** (-28,-6)	**-20** (-31,-8)	-9 (-21,2)	-3 (-15,8)	-1 (-15,13)	-4 (-15,7)	**-14** (-25,-3)	**-21** (-34,-7)
	90th percentile	58 (ref.)	**-23** (-30,-16)	**-16** (-22,-11)	**-19** (-26,-12)	**-10** (-14,-6)	9 (-6,24)	8 (-3,20)	**55** (38,71)	**-17** (-26,-8)	**-17** (-20,-13)
**Diagnostic interval (All)**	Number	224	353	356	302	338	350	329	309	343	259
	Median	29 (ref.)	**-14** (-16,-12)	**-8** (-11,-6)	**-13** (-15,-11)	**-14** (-16,-11)	-2 (-5,1)	**-6** (-9,-2)	-1 (-5,2)	**-12** (-15,-10)	**-14** (-17,-11)
	75th percentile	49 (ref.)	**-30** (-34,-25)	**-18** (-23,-14)	**-29** (-33,-25)	**-20** (-25,-16)	-5 (-15,4)	**-11** (-20,-2)	**-8** (-12,-3)	**-22** (-26,-19)	**-25** (-32,-17)
	90th percentile	70 (ref.)	**-35** (-39,-31)	**-23** (-30,-16)	**-26** (-34,-19)	**-21** (-40,-2)	**20** (9,31)	-1 (-16,14)	**22** (7,38)	**-27** (-40,-13)	**-29** (-36,-21)
**Treatment interval (Symptomatic)**	Number	115	203	202	241	230	201	216	198	196	113
	Median	24 (ref.)	**6** (1,11)	1 (-3,5)	-3 (-7,1)	-3 (-7,1)	**12** (8,16)	-5 (-11,1)	**15** (11,19)	**-10** (-14,-6)	-2 (-6,2)
	75th percentile	33 (ref.)	**8** (4,12)	4 (-1,9)	-3 (-8,1)	-4 (-9,0)	**16** (11,21)	**-6** (-9,-2)	**22** (18,26)	**-9** (-16,-2)	-4 (-13,5)
	90th percentile	45 (ref.)	10 (-1,20)	11 (-4,26)	-4 (-19,11)	-1 (-14,12)	**20** (10,31)	-11 (-23,2)	**28** (10,46)	-11 (-26,4)	-3 (-15,10)
**Treatment interval (Screen-detected)**	Number	115	155	158	86	134	195	120	155	170	165
	Median	27 (ref.)	**5** (1,8)	**8** (4,11)	0 (-5,5)	**-6** (-10,-1)	**9** (7,12)	**-12** (-15,-10)	**12** (9,16)	-5 (-12,1)	**-3** (-6,-1)
	75th percentile	36 (ref.)	4 (-2,10)	10 (-3,22)	0 (-6,7)	-7 (-16,3)	**11** (3,20)	**-15** (-27,-4)	**15** (7,23)	-7 (-16,2)	-6 (-17,5)
	90th percentile	49 (ref.)	5 (-8,18)	**15** (4,25)	-9 (-20,3)	**-14** (-26,-2)	9 (-4,22)	**-24** (-35,-13)	12 (-1,24)	**-14** (-27,-1)	-11 (-21,0)
**Treatment interval (All)**	Number	231	359	361	330	368	400	341	353	366	278
	Median	25 (ref.)	5 (0,11)	4 (0,8)	-4 (-8,1)	**-5** (-9,-1)	**10** (6,14)	**-10** (-14,-6)	**14** (9,18)	**-10** (-14,-6)	-3 (-7,0)
	75th percentile	35 (ref.)	**6** (2,10)	**6** (1,10)	-4 (-8,0)	**-6** (-10,-2)	**13** (10,17)	**-11** (-16,-6)	**20** (16,23)	**-9** (-13,-5)	**-6** (-10,-2)
	90th percentile	46 (ref.)	**8** (2,14)	12 (-1,26)	**-6** (-10,-2)	**-6** (-9,-2)	**17** (11,23)	**-14** (-17,-12)	**23** (16,29)	**-11** (-14,-9)	**-7** (-10,-4)
**Total interval**	Number	104	190	186	205	189	173	184	155	177	98

For peer review only - http://bmjopen.bmj.com/site/about/guidelines.xhtml

**(Symptomatic)**	Median	70 (ref.)	**-14** (-24,-3)	**-12** (-23,-2)	**-20** (-31,-8)	**-25** (-38,-11)	**28** (15,42)	-15 (-34,5)	**22** (12,31)	**-28** (39,-17)	-1 (-11,10)
	75th percentile	96 (ref.)	**-16** (-27,-5)	-5 (-18,9)	**-18** (-33,-3)	**-25** (-39,-12)	**69** (31,107)	**27** (8,46)	**30** (8,53)	-13 (-27,0)	3 (-30,35)
	90th percentile	218 (ref.)	**-71** (-102,-41)	**-60** (-85,-34)	**-57** (-66,-48)	**-41** (-66,-16)	**79** (66,93)	8 (-15,30)	**-17** (-34,-1)	-21 (-67,26)	**-39** (-67,-10)
**Total interval (All)**	Number	217	343	337	291	320	354	298	277	327	253
	Median	60 (ref.)	**-9** (-14,-3)	-4 (-11,2)	**-13** (-18,-8)	**-15** (-26,-4)	**16** (6,26)	**-13** (-21,-6)	**17** (9,24)	**-18** (-24,-12)	**-17** (-25,-10)
	75th percentile	81 (ref.)	**-12** (-20,-4)	1 (-9,10)	**-13** (-20,-6)	**-14** (-20,-9)	**32** (24,41)	-4 (-12,4)	**35** (21,48)	**-19** (-25,-12)	**-17** (-26,-7)
	90th percentile	123 (ref.)	-13 (-34,9)	3 (-12,18)	-5 (-22,11)	-8 (-38,22)	**73** (33,114)	**32** (2,62)	**47** (13,82)	-12 (-37,13)	-24 (-51,4)

The differences for the median, 75th and 90th percentiles are calculated as marginal effects after quantile regression by setting the continuous covariate age to its mean value and comorbidity to the mode. Significant results are shown in bold. The actual number of days are included for Wales are shown in Table 5. Note that analyses of primary care intervals are presented in Table 6.

For peer review only

For peer review only - http://bmjopen.bmj.com/site/about/guidelines.xhtml

Figure One – Diagnostic and treatment intervals[23]

For peer review only

For peer review only - http://bmjopen.bmj.com/site/about/guidelines.xhtml

Figure Two – Time intervals for each jurisdiction compared with Wales (reference)*

**Adjusted for differences in comorbidity and age. Differences in interval lengths (in days) are shown for the median, 75th and 90th percentiles compared to the reference used for the regression analyses, Wales. Wales is represented by the axis, with jurisdictions with shorter intervals shown to the left of the axis, and jurisdictions with longer intervals shown to the right of the axis for each graph. Solid-fill bars indicate statistically significant differences compared with Wales. Primary care interval not shown, see Table 6.*

For peer review only

For peer review only - http://bmjopen.bmj.com/site/about/guidelines.xhtml

Diagnostic pathways for breast cancer in ten jurisdictions: Findings from the International Cancer Benchmarking Partnership (ICBP) _ Supplementary Materials

Table 1. *Patient flow from identification to analyses for all ten jurisdictions and totally*

	Patients approached via GP						Patient approached directly by registries/research teams														Total	
**Jurisdiction**	**Wales**		**England**		**Scotland**		**N Ireland**		**Denmark**		**Ontario**		**Norway**		**Manitoba**		**Victoria**		**Sweden**			
	n	(%)	n	(%)	n	(%)	n	(%)	n	(%)	n	(%)	n	(%)	n	(%)	n	(%)	n	(%)	n	(%)
**Eligible patients [a, b]**	**808**	**(100%)**	**1,222**	**(100%)**	**2,614**	**(100%)**	**696**	**(100%)**	**488**	**(100%)**	**4,475[i]**	**(100%)**	**1,962**	**(100%)**	**1.552**	**(100%)**	**1,164[j]**	**(100%)**	**440**	**(100%)**	**15,421**	**(100%)**
**Packs sent to GP [c, d]**	**808**	**(98.3%)**	**1,222**	**(99.7%)**	**1,113**	**(42.0%)**															**3,143**	**(67.7%)**
pack not forwarded by GP	45	(5.5%)	41	(3.3%)	76	(6.6%)															162	(5.2%)
unsure if pack forwarded by GP	777	(94.5%)	1,185	(96.7%)	1,073	(93.4%)															3,035	(96.6%)
**Patients contacted by GP [c, d]**	**533**	**(64.8%)**	**819**	**(66.8%)**	**823**	**(71.6%)**															**2,175**	**(69.2%)**
**Patients approached directly [c]**							**643**	**(92.4%)**	**488**	**(100%)**	**4,475**	**(100%)**	**1,875**	**(95.6%)**	**751**	**(48.4%)**	**964**	**(82.8%)**	**440**	**(100%)**	**9,636**	**(89.4%)**
patient died							1	(0.2%)	0	(0.0%)	21	(0.5%)	0	(0.0%)	17	(2.3%)	0	(0.0%)	0	(0.0%)	39	(0.4%)
no address							0	(0.0%)	0	(0.0%)	243	(5.4%)	0	(0.0%)	6	(0.8%)	0	(0.0%)	0	(0.0%)	249	(2.6%)
Other							12	(1.9%)	0	(0.0%)	341	(7.6%)	0	(0.0%)	3	(0.4%)	0	(0.0%)	0	(0.0%)	356	(3.7%)
**Patient responses (% of eligible patients)[c]**	**267**	**(33.0%)**	**384**	**(31.4%)**	**382**	**(14.6%)**	**348**	**(50.0%)**	**380**	**(77.9%)**	**1,217**	**(27.2%)**	**451**	**(23.0%)**	**376**	**(24.2%)**	**489[i]**	**(42.0%)**	**299**	**(68.0%)**	**4,593**	**(29.8%)**

For peer review only

**Patient responses (% of contacted)[e]**	**267**	**(50.1%)**	**384**	**(46.9%)**	**382**	**(46.4%)**	**348**	**(55.2%)**	**380**	**(77.9%)**	**1,217**	**(31.4%)**	**451**	**(24.1%)**	**376**	**(51.9%)**	**489**	**(50.7%)**	**299**	**(68.0%)**	**4,593**	**(39.9%)**
extra sample for local purpose	0	(0.0%)	0	(0.0%)	0	(0.0%)	0	(0.0%)	0	(0.0%)	606	(49.8%)	0	(0.0%)	0	(0.0%)	110	(22.5%)	0	(0.0%)	716	(15.6%)
received after submission	0	(0.0%)	0	(0.0%)	0	(0.0%)	0	(0.0%)	0	(0.0%)	53	(4.4%)	0	(0.0%)	0	(0.0%)	0	(0.0%)	0	(0.0%)	53	(1.2%)
**Patient questionnaires submitted for analyses [f]**	**267**	**(100%)**	**384**	**(100%)**	**382**	**(100%)**	**348**	**(100%)**	**380**	**(100%)**	**558**	**(45.9%)**	**451**	**(100%)**	**376**	**(100%)**	**379**	**(77.5%)**	**299**	**(100%)**	**3,824**	**(83.3%)**
excluded for analyses – total	35	(13.1%)	21	(5.5%)	11	(2.9%)	17	(4.9%)	11	(2.9%)	155	(27.8%)	79	(17.5%)	8	(2.1%)	9	(2.4%)	7	(2.3%)	353	(9.2%)
- previous cancer	0	(0.0%)	16	(4.2%)	0	(0.0%)	14	(4.0%)	0	(0.0%)	128	(22.9%)	5	(1.1%)	0	(0.0%)	0	(0.0%)	0	(0.0%)	163	(4.3%)
- unknown date of consent or diagnosis	0	(0.0%)	0	(0.0%)	1	(0.3%)	0	(0.0%)	2	(0.5%)	0	(0.0%)	15	(3.3%)	1	(0.3%)	0	(0.0%)	7	(2.3%)	26	(0.7%)
- consent too late/too early	35	(13.1%)	5	(1.3%)	10	(2.6%)	1	(0.3%)	9	(2.4%)	27	(4.8%)	56	(12.4%)	7	(1.9%)	9	(2.4%)	0	(0.0%)	159	(4.2%)
- Other	0	(0.0%)	0	(0.0%)	0	(0.0%)	2	(0.6%)	0	(0.0%)	0	(0.0%)	3	(0.7%)	0	(0.0%)	0	(0.0%)	0	(0.0%)	5	(0.1%)
**Patients included in analyses[k] (% of forwarded questionnaires)**	**232**	**(86.9%)**	**363**	**(94.5%)**	**371**	**(97.1%)**	**331**	**(95.1%)**	**369**	**(97.1%)**	**403**	**(72.2%)**	**372**	**(82.5%)**	**368**	**(97.9%)**	**370**	**(97.6%)**	**292**	**(97.7%)**	**3,471**	**(90.8%)[k]**
**GP questionnaire [l] (% of analysed patients)**	**191**	**(82.3%)**	**295**	**(81.3%)**	**301**	**(81.1%)**	**284**	**(85.8%)**	**260**	**(70.5%)**	**181**	**(44.9%)**	**197**	**(53.0%)**	**232**	**(63.0%)**	**240**	**(64.9%)**	**n/a [h]**		**2,181**	**(62.8%)**
**Specialist questionnaire [m] (% of analysed patients)**	**163**	**(70.3%)**	**225**	**(62.0%)**	**212**	**(57.1%)**	**n/a [g]**		**178[g]**	**(48.2%)**	**117**	**(29.0%)**	**102**	**(27.4%)**	**n/a [h]**		**184**	**(49.7%)**	**n/a [h]**		**1,181**	**(42.6%)[m]**

[a] Eligible according to protocol: i.e. woman, 40 years or more, alive, consented to participate within nine months of diagnosis, diagnosed with breast cancer (ICD-10: C50.0-C50.9), behaviour code ICD-O-3=3 and without prior history of cancer of the breast or synchronous primary breast cancer. [b] In some jurisdictions some 'eligible' patients had pre-opted out from being contacted and a small number where GP information was not available. [c] Percentages of eligible patients. [d] Maximum of potentially contacted patients, i.e. sum of packs forwarded by GP and packs unsure if forwarded by GP. [e] Percentages of patients contacted by GP (see note d) for Wales, England and Scotland or percentages of patients contacted directly by a registry excl. non-accessible patients (all other jurisdictions). [f] Percentages of patient responses. [g] Data obtained from registries instead: N. Ireland from N. Ireland Cancer Registry; Denmark from the Danish Breast Cancer Group.

[h] Data not collected in this jurisdiction. [i] Initially, additional 2,472 cases were eligible but excluded for this study as urban sample size was reached. [j] Additionally 92 cases were ineligible based on registry-criteria exclusions and a further 108 excluded after treating doctor approach. [k] Denominator = total number of forwarded cases excl. patients not included in analytic sample in Ontario. [l] Denominator = total number of forwarded cases excl. patients from Sweden. [m] Denominator = total number of forwarded cases excl. patients from Sweden, Manitoba & N Ireland.

For peer review only

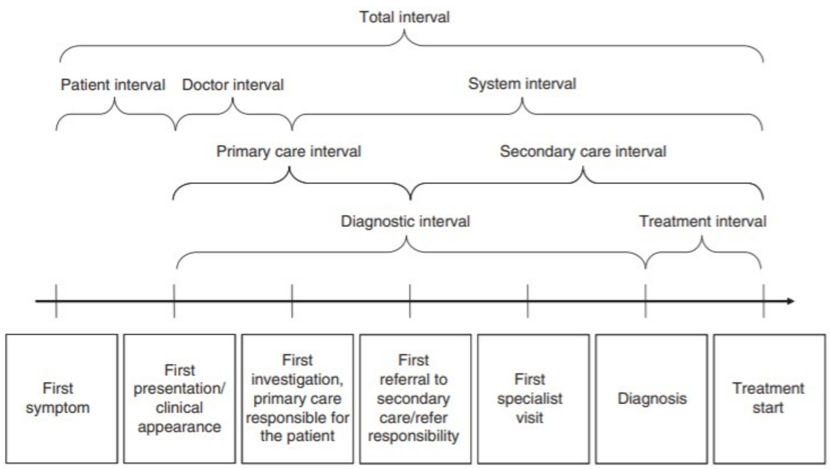

Diagnostic and treatment intervals

155x74mm (144 x 144 DPI)

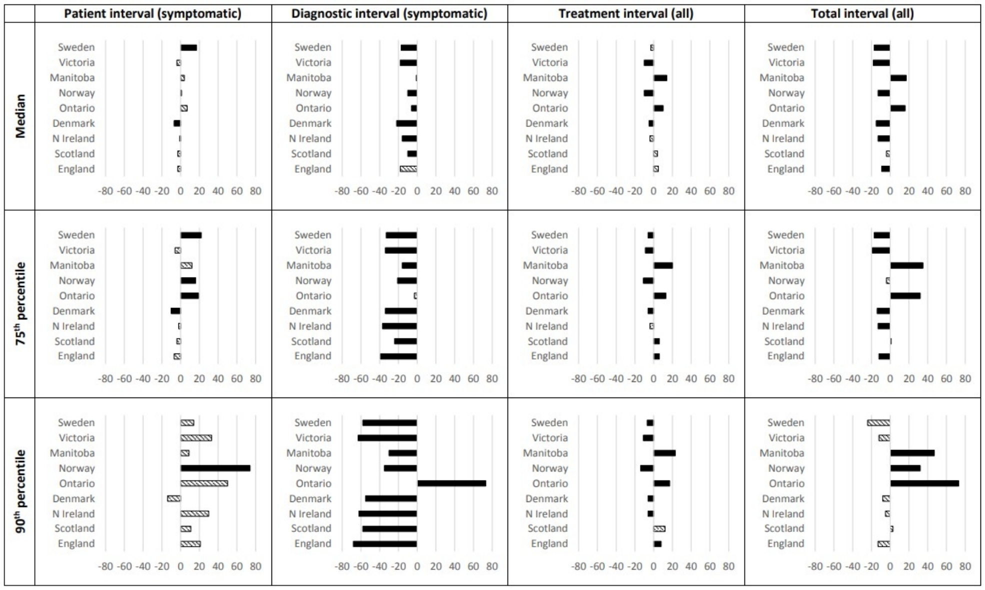

Time intervals for each jurisdiction compared with Wales (reference)*
*Adjusted for differences in comorbidity and age. Differences in interval lengths (in days) are shown for the median, 75th and 90th percentiles compared to the reference used for the regression analyses, Wales. Wales is represented by the axis, with jurisdictions with shorter intervals shown to the left of the axis, and jurisdictions with longer intervals shown to the right of the axis for each graph. Solid-fill bars indicate statistically significant differences compared with Wales. Primary care interval not shown, see Table 6.

208x126mm (144 x 144 DPI)

BMJ Open

Diagnostic pathways for breast cancer in ten International Cancer Benchmarking Partnership (ICBP) jurisdictions: An International comparative cohort study based on questionnaire and registry data

Journal:	*BMJ Open*
Manuscript ID	bmjopen-2021-059669.R1
Article Type:	Original research
Date Submitted by the Author:	10-Mar-2022
Complete List of Authors:	Vedsted, Peter; Aarhus Universitet, Research Unit for General Practice<br>Weller, David; University of Edinburgh, General Practice<br>Zalounina Falborg, Alina; Research Unit for General Practice, Department of Public Health, Aarhus University<br>Jensen, Henry; Research Unit for General Practice, Department of Public Health, Aarhus University; Research Unite for General Practice, Department of Public Health, Aarhus university<br>Kalsi, Jatinderpal; University College London, Gynaecological Cancer Research Centre<br>Brewster, David; NHS National Services Scotland, Scottish Registry, Information Services Division<br>Lin, Yulan; NTNU Fakultet for ingeniorvitenskap og teknologi Trondheim, Department of Cancer Research and Molecular Medicine<br>Gavin, Anna; Queen's University Belfast, N. Ireland Cancer Registry<br>Barisic, Andriana; Cancer Care Ontario, Renal Network<br>Grunfeld, Eva; Ontario Institute for Cancer Research, Dept. of Family and Community Medicine<br>Lambe, Mats; Regional Cancer Centre of central Sweden, University Hospital<br>Malmberg, Martin; Lund University Hospital, 9. Department of Oncology<br>Turner, Donna; Cancer Care Manitoba, Population Oncology<br>Harland, Elizabeth; CancerCare Manitoba, Department of Epidemiology and Cancer Registry<br>Hawryluk, Breann; CancerCare Manitoba, Patient Navigation<br>Law, Rebecca-Jane; Bangor University, North Wales Centre for Primary Care Research<br>Neal, Richard; University of Leeds,<br>White, Victoria; Cancer Council Victoria, CBRC; Deakin University Faculty of Health,<br>Bergin, Rebecca; Cancer Council Victoria, Centre for Behavioral Research in Cancer<br>Harrison, Samantha; Cancer Research UK, Policy and Information<br>Menon, Usha; University College London, Women's Cancer<br>Working Group, ICBP; Cacncer Research UK, Policy and Information
**Primary Subject Heading**:	Public health

Secondary Subject Heading:	Diagnostics, Epidemiology, Medical management
Keywords:	Breast tumours < ONCOLOGY, Epidemiology < ONCOLOGY, Adult oncology < ONCOLOGY, International health services < HEALTH SERVICES ADMINISTRATION & MANAGEMENT, PUBLIC HEALTH

SCHOLARONE™
Manuscripts

I, the Submitting Author has the right to grant and does grant on behalf of all authors of the Work (as defined in the below author licence), an exclusive licence and/or a non-exclusive licence for contributions from authors who are: i) UK Crown employees; ii) where BMJ has agreed a CC-BY licence shall apply, and/or iii) in accordance with the terms applicable for US Federal Government officers or employees acting as part of their official duties; on a worldwide, perpetual, irrevocable, royalty-free basis to BMJ Publishing Group Ltd ("BMJ") its licensees and where the relevant Journal is co-owned by BMJ to the co-owners of the Journal, to publish the Work in this journal and any other BMJ products and to exploit all rights, as set out in our licence.

The Submitting Author accepts and understands that any supply made under these terms is made by BMJ to the Submitting Author unless you are acting as an employee on behalf of your employer or a postgraduate student of an affiliated institution which is paying any applicable article publishing charge ("APC") for Open Access articles. Where the Submitting Author wishes to make the Work available on an Open Access basis (and intends to pay the relevant APC), the terms of reuse of such Open Access shall be governed by a Creative Commons licence – details of these licences and which Creative Commons licence will apply to this Work are set out in our licence referred to above.

Other than as permitted in any relevant BMJ Author's Self Archiving Policies, I confirm this Work has not been accepted for publication elsewhere, is not being considered for publication elsewhere and does not duplicate material already published. I confirm all authors consent to publication of this Work and authorise the granting of this licence.

For peer review only

Diagnostic pathways for breast cancer in ten International Cancer Benchmarking Partnership (ICBP) jurisdictions: An International comparative cohort study based on questionnaire and registry data

Corresponding author:

Peter Vedsted

Research Unit for General Practice, Aarhus University

Bartholins Alle 2, 8000, Aarhus, Denmark

p.vedsted@ph.au.dk

1. **Peter Vedsted** *, Research Unit for General Practice, Department of Public Health, Aarhus University, Bartholins Allé 2, 8000 Aarhus C, Denmark, p.vedsted@ph.au.dk
2. **David Weller** *, Centre for Population Health Sciences, University of Edinburgh, Doorway 1, Medical Quad Teviot Place, Edinburgh, EH8 9DX, United Kingdom, david.weller@ed.ac.uk
3. **Alina Zalounina Falborg**, Research Unit for General Practice, Department of Public Health, Aarhus University, Bartholins Allé 2, 8000 Aarhus C, Denmark, az@ph.au.dk
4. **Henry Jensen**, Research Unit for General Practice, Department of Public Health, Aarhus University, Bartholins Allé 2, 8000 Aarhus C, Denmark, henry.jensen@feap.dk
5. **Jatinderpal Kalsi**, Gynaecological Cancer Research Centre, Women's Cancer, Institute for Women's Health, University College London, United Kingdom, j.k.kalsi@ucl.ac.uk
6. **David H Brewster**, Scottish Cancer Registry, Information Services Division, NHS National Services Scotland, Gyle Square, 1 South Gyle Crescent, Edinburgh, EH12 9EB, United Kingdom, David.Brewster@isd.csa.scot.nhs.uk
7. **Yulan Lin**, Department of Cancer Research and Molecular Medicine, Faculty of Medicine, Norwegian University of Science and Technology (NTNU), N-7491 Trondheim, Norway, yulan1984_lin@hotmail.com
8. **Anna Gavin**, Northern Ireland Cancer Registry, Centre for Public Health, Queen's University Belfast, Mulhouse Building, Mulhouse Road, Belfast, BT12 6DP, United Kingdom, A.Gavin@qub.ac.uk
9. Andriana Barisic, Department of Prevention and Cancer Control, Cancer Care Ontario, 620 University Avenue, Toronto, Ontario, M5G 2L7, Canada, Andriana.Barisic@renalnetwork.on.ca
10. **Eva Grunfeld**, Department of Family and Community Medicine, University of Toronto, 500 University Avenue, Toronto, Ontario, M5G 1V7, Canada, eva.grunfeld@utoronto.ca
11. **Mats Lambe**, Regional Cancer Center Uppsala and Department of Medical Epidemiology and Biostatics, Karolinska Institutet, SE-171 77 Stockholm, Sweden, mats.lambe@ki.se
12. **Martin Malmberg**, Department of Oncology, Lund University Hospital, SE-221 85 Lund, Sweden, Martin.Malmberg@skane.se
13. **Donna Turner**, Population Oncology, Cancer Care Manitoba, 675 McDermot Street, Winnipeg, Manitoba, Canada, donna.turner@cancercare.mb.ca
14. **Elizabeth Harland**, Department of Epidemiology and Cancer Registry, CancerCare Manitoba, 675 McDermot Street, Winnipeg, Manitoba, eharland@cancercare.mb.ca

For peer review only

15. **Breann Hawryluk**, Department of Patient Navigation, Cancer Care Manitoba, 675 McDermot Street, Winnipeg, Manitoba, Canada, Breann_haw@hotmail.com
16. **Rebecca-Jane Law**, North Wales Centre for Primary Care Research, Bangor University, Wrexham, LL13 7YP, United Kingdom, r.law@bangor.ac.uk
17. **Richard D Neal**, Academic Unit of Primary Care, Leeds Institute of Health Sciences, University of Leeds, Leeds LS2 9NL, United Kingdom, r.d.neal@leeds.ac.uk
18. **Victoria White,** Centre for Behavioral Research in Cancer, Cancer Council Victoria, 615 St Kilda Road, Melbourne, Victoria, 3004, Australia, vicki.white@cancervic.org.au
19. **Rebecca J Bergin,** Cancer Epidemiology Division at Cancer Council Victoria, 615 St Kilda Road, Melbourne, Victoria, 3004, Australia, Rebecca.Bergin@cancervic.org.au
20. **Samantha Harrison,** Policy, Information & Communications, Cancer Research UK, 2 Redman Place, London, E20 1JQ, UK, samantha.harrison@cancer.org.uk
21. **Usha Menon** *, MRC Clinical Trials Unit, Institute of Clinical Trials and Methodology, University College London, United Kingdom, u.menon@ucl.ac.uk
22. **ICBP Module 4 Working Group**

* equal contribution

Number of words: 3598
Number of tables: 6
Number of figures: 2
Number of Appendices: 7

For peer review only - http://bmjopen.bmj.com/site/about/guidelines.xhtml

Abstract

Objectives*:* Growing evidence suggests longer times between symptom onset to start of treatment affects breast cancer prognosis. The International Cancer Benchmarking Partnership Module 4 examines differences in breast cancer diagnostic pathways in 10 jurisdictions across Australia, Canada, Denmark, Norway, Sweden, and the UK.

Design*:* Data was collated from 3,471 women aged >40 diagnosed first-time with breast cancer surveyed between 2013 and 2015, their primary care physician (PCP), cancer treatment specialists, and available registry data.

Results: Overall, 56% of women reported symptoms to primary care, and 66% first noticing lump/s or breast changes. PCPs reported 77% presented with symptom/s, of whom 81% were urgently referred with suspicion of cancer, (ranging 62-92%, Norway; Victoria). The median patient, primary care and diagnostic intervals (days) for symptomatic patients ranged: 3-29 (Denmark; Sweden), 0-20 (seven jurisdictions; Ontario), 8-29 (Denmark; Wales). The median treatment and total intervals (days) for all patients ranged: 15-39 (Norway, Victoria; Manitoba) and 4 -78 days (Sweden, Victoria; Ontario). The 10% longest waits ranged 101-209 days (Sweden; Ontario).

Conclusions: Large international differences in breast cancer diagnostic pathways exist, suggesting some jurisdictions examine strategies to optimise pathways and reduce time intervals. Targeted awareness interventions could also facilitate more timely diagnosis of breast cancer.

Keywords

Delayed diagnosis; Questionnaire; International comparisons, Early Detection of Cancer; Breast neoplasms; Health services, Health planning; Signs and Symptoms; Screening

For peer review only

Strengths and limitations of this study

- Internationally standardised survey methodology to explore and compare key intervals from symptom onset to treatment start.
- Comprehensive data pulled from state/provincial-level cancer registries and other sources to create as complete a record as possible of patient pathways to diagnosis and treatment
- Minimal recall bias by ensuring that the patients received the questionnaire with a limited time window after the cancer diagnosis and, through triangulation of different data sources, multiple perspectives to provide comprehensive understanding - from patients, primary care practitioners, cancer treatment specialist and registry sources
- Some jurisdictions were not able to recruit a sufficient number of patients to power this study
- Our cohort is not representative of all breast cancer patients as there was high self-selection of early stage breast cancer patients

Introduction

Breast cancer is the most common cancer among women in western countries. The incidence of female breast cancer is around 80 per 100,000 (standardised world population); by the age of 80, approximately 12% of women will have received a breast cancer diagnosis [1]. Although the prognosis of breast cancer has improved dramatically during recent decades [2], there are still important differences in disease-specific mortality and survival, both between and within countries [3,4]. For example, in 2010-14, the 5-year net survival varied from 85.6% in the UK, to 88.8% in Sweden, and 89.5% in Australia [2].

Differences in survival may relate to timely cancer diagnosis and access to optimal treatment and can also affect patient satisfaction with care and healthcare costs [5-10]. Therefore, it becomes important to examine international differences in routes to diagnosis and treatment together with time intervals from first noticing symptoms until start of treatment.

For peer review only - http://bmjopen.bmj.com/site/about/guidelines.xhtml

For peer review only

Women differ in their help-seeking behaviours for breast cancer symptoms [11]. These behaviours might influence the time interval between first noticing symptoms until they present to healthcare [12]. Further, a healthcare system's organisation might affect the way patients seek help, how healthcare professionals respond to these symptoms, and when and how they can refer for further investigations [13-16]. In some countries, breast cancer diagnosis is standardised or expedited with urgent referrals, where significant breast cancer specific 'red flag symptoms' are present – further, many countries have now implemented breast cancer screening programmes [17]. Therefore, routes to diagnosis and time intervals from first noticing symptoms to breast cancer diagnosis and treatment may vary between countries. This has consequences for patient outcomes and experience, as well as healthcare costs.

To date almost all breast cancer routes to diagnosis studies have been based on a single country [18]; therefore international comparisons can shed light on factors which underpin observed differences. Accordingly, we undertook a study to explore pathways to diagnosis and treatment for female breast cancer in ten jurisdictions within six countries.

Methods

We undertook an international comparative cohort study based on questionnaire and registry data on newly diagnosed, first-time female breast cancer patients aged 40 or over.

Study context and management

Within the International Cancer Benchmarking Partnership (ICBP), Module 4 (ICBP M4) aims to explore differences in routes to diagnosis for four cancers; the methods used are described in detail elsewhere [19,20]. Briefly, the study was undertaken in ten jurisdictions in six countries: Australia (Victoria), Canada (Manitoba and Ontario), Denmark, Norway, Sweden, and the United Kingdom (England, Northern Ireland, Scotland, and Wales). We recruited jurisdictions with universal health care access, covering breast cancer

For peer review only

investigations and screening programmes ,and that have previously shown variation in breast cancer survival [20].

Identification of study population

Eligible female patients, aged 40 years or more, were consecutively identified with a first diagnosis of invasive breast cancer (ICD10 codes C50.0—C50.9), irrespective of symptomatic or screen-based diagnosis. Women with previous diagnoses of other cancers were included but women with a previous diagnosis of breast cancer or synchronous breast cancer were excluded.

Participants were identified via cancer registries and hospital databases, although processes varied locally. Processes were tested in each jurisdiction to ensure adaptation of data collection, questionnaire logistics and data management to the local settings [20]. Each jurisdiction aimed to recruit 200 symptomatic breast cancer patients, irrespective of the number of screen-based diagnoses. In Ontario and Victoria, the sampling continued beyond 200 patients for use in local studies. In Northern Ireland, most screen-detected women were excluded at the sampling stage by the cancer registry.

Data sources

Data were collected primarily from three questionnaires sent to the eligible women (Additional File 1), their primary care physician (PCP; Additional File 2) and their cancer treatment specialists (CTSs; Additional File 3). This was supplemented with registry data (including diagnosis date, tumour stage and screening) in all jurisdictions except Ontario and Norway [19]. Data were anonymised prior to transfer to Aarhus University (Denmark) for analysis.

Questionnaire and registry data

For peer review only - http://bmjopen.bmj.com/site/about/guidelines.xhtml

The validated questionnaires included items on routes to diagnosis, symptoms, specific milestones from first noticing symptoms to starting treatment, patient socio-demographics and comorbidities [20]. Eligible patients were identified from cancer registries, and patients either contacted directly or via their PCP.

Women were sent questionnaires 3-9 months after diagnosis; PCPs and CTSs were sent questionnaires after patients consented to participation in the study. Completed questionnaires were returned to the local research teams. Where possible, registries and disease databases provided additional checks and information on date of diagnosis, screening status and start of treatment. Date of diagnosis was based on the International Agency for Research on Cancer (IARC) hierarchy, and tumour stage using tumour, node, metastasis (TNM) classification [21,22].

Data handling

Curation of questionnaire data were primarily done centrally (Aarhus University, Demark) to ensure uniform application across jurisdictions.

A set of algorithms ('rules') to validate and combine data from the different sources in a standardised way was developed (Additional File 4); they employed a hierarchy-principle regarding the order in which different data sources were used. Any queries were discussed and resolved with the local team.

Routes to diagnosis

Differences in the diagnostic routes were investigated drawing on the checklist from the Aarhus Statement [23]. Routes to diagnosis are outlined in Box 1:

BOX 1 – Routes to diagnosis

- **Screening**
- **Symptomatic:**
  - Visit PCP
  - Visit PCP and then accident and emergency department (A&E)

For peer review only - http://bmjopen.bmj.com/site/about/guidelines.xhtml

○ Direct to A&E
○ Investigation for another problem
- **Other/unknown routes to diagnosis**

Measures of time intervals

Time interval definitions were adapted from the Aarhus Statement and included the time-points outlined in Box 2 [23]; the intervals are represented graphically in Figure 1.

BOX 2 – Time intervals

a) First onset of symptoms: the time-point when first bodily change(s) and/or symptom(s) were first noticed by the patient.
b) First presentation to healthcare: the time-point at which the PCP noted a symptom that in retrospect could be due to the underlying cancer.
c) First referral to secondary care: the time-point at which the PCP referred the patient (and responsibility) to secondary/specialist care.
d) Date of diagnosis, following the IARC definition.[21]
e) Date of treatment start: when the patient started curative/palliative treatment (in Manitoba only curative treatment was registered).

INSERT FIGURE ONE

For screen-detected women, we used the date of the screening test as the start of the diagnostic and total interval measurements. The analyses include intervals for both symptomatic, screen-detected and all breast cancer patients. We included a categorical variable where the symptomatic women reported the waiting time for their appointment with the PCP. Cancers were only considered to be screen-detected if the woman or PCP had indicated a screening route and there were no symptoms nor symptomatic pathway reported. For Wales, England, Scotland, Northern Ireland, Denmark, Manitoba, and Sweden the distinction between a screen-detected and non-screen-detected breast cancer was based on registry data from public screening programs (Table 1).

INSERT TABLE 1

For peer review only - http://bmjopen.bmj.com/site/about/guidelines.xhtml

Covariates

General health status was measured using the self-reported general health item from the 12-Item Short Form Health Survey (SF12) [24]. Comorbidity was assessed through the patient questionnaire as presence of any of four chronic diseases (heart disease, lung diseases, stroke, or diabetes) and categorised into 'none', 'medium' (one or two), or 'high' (three or four). Educational level (from the patient questionnaire) was categorised as 'low' (vocational school or lower) and 'high' (university or higher). Smoking was categorised into 'current', 'past' or 'never' smoker. Symptoms reported by the PCP were divided into 'breast cancer specific symptoms' or 'other symptoms'. This was based on symptom coding, drawing on local clinical guidelines [17,24].

Patient and Public Involvement

The research question and questionnaires were developed based and validated on input from cancer patients and cancer organisations in several countries. The study design has emerged from studies on the subject in Denmark in 2004 and refined trough the International Cancer Benchmarking Project. The results from the several arts of the ICBP are continuously communicated to cancer organisations and health authorities. We thank the large number of patients and their relatives in taking part.

Statistical analysis

Patients with missing data on age, date of diagnosis and/or date of consent were excluded. Quantile regression [26] was used to estimate differences in intervals at the median (50th), 75th and 90th percentiles between all jurisdictions with Wales as the reference. Counting days, we used the quantile regression for count data [27] (Supplementary Box 1) and the corresponding 'qcount' and 'qcount_mfx' procedures in Stata [28]. The analyses were adjusted for age (continuous variable) and comorbidity (categorical variable). The differences in intervals were calculated as marginal effects after quantile regression by setting the continuous covariates to their mean values and the categorical covariates to their modes.

For peer review only - http://bmjopen.bmj.com/site/about/guidelines.xhtml

Due to zero-inflation, quantile regression could not be used for the primary care interval. Here, a generalised linear model with Poisson family and log link and robust error variance was used to calculate the association (prevalence ratio (PR)) between longer primary care intervals and jurisdiction. Longer intervals were defined as over 5 or 14 days to test both intervals. The analyses were adjusted for age (continuous variable) and comorbidity (categorical variable). The significance level was set to 0.05 or lower, and 95% confidence intervals (95%CI) were calculated when appropriate. Statistical analyses were carried out using Stata v16 software.

Sensitivity and validity analyses

Analyses were repeated using the per-protocol definition of a maximum of 6 months between diagnosis and patient consent, to assess the impact of including patients who consented at 9 months post-diagnosis. To estimate the effect of using patient-reported intervals only, a sensitivity analysis based solely on the patient questionnaire data was performed.

Kappa coefficients were used to assess the agreement on routes to diagnosis (screening and symptomatic presentation) between the different data sources. Lin's concordance correlation coefficient (CCC) was used to assess the agreement on dates between the different data sources [29].

Results

Patient characteristics and participation

Across all jurisdictions, 15,421 eligible women were identified between May 2013 and August 2015. A total of 4,593 (38.9%) of those contacted returned completed questionnaires and 3,471 (75.6%) could be included in the analyses. The patient flow - with identification, exclusion and responses for each jurisdiction - is detailed in Supplementary Figure 1. The response rates ranged from 24.1% (451/1,875) in Norway to 77.9% (380/488) in Denmark (Supplementary Figure 1). Participating women were younger and had a less advanced

For peer review only - http://bmjopen.bmj.com/site/about/guidelines.xhtml

tumour stage distribution than all eligible women in the study base (Additional File 5). The characteristics of the included women are detailed in Table 1.

INSERT TABLES 1 & 2

First presentation and symptoms

Table 2 shows routes to diagnosis; 42.9% had a screen-detected breast cancer, with the highest proportion in Sweden (58.6%)[1]. For symptomatic patients, primary care was the place of first presentation for 85.6% of women, ranging from 69.2% in Ontario to 93.7% in England. According to PCPs, 81.1% of symptomatic women were urgently referred with a suspicion of cancer, ranging from 62.4% in Norway to 92.2% in Victoria (data not shown).

Table 3 shows the first symptom or sign reported by women, together with those reported by PCPs (for Sweden, only patient data were collected). Two thirds of symptomatic women noticed a lump or change in the breast. This was also the most commonly reported sign by PCPs, followed by pain/tenderness, and change in the nipple. According to PCPs, 77% presented with a symptom or sign indicative of breast cancer. While one in ten women indicated fatigue as a symptom, this was rarely noted by PCPs.

INSERT TABLES 5 & 6

Time intervals

The median patient interval for symptomatic women varied from 3 days (Denmark) to 29 days (Sweden) (Table 4). Based on the quantile regression, the patient interval was 7 days shorter in Denmark and 17 days longer in Sweden compared with Wales (Figure 2 and Table 5). When comparing the 90th percentile, Norway (157 days) and Ontario (142 days) had the longest patient intervals (Table 4). Quantile regression showed

[1] *Note that the proportion of screen-detected cancers in Northern Ireland is lower due to the initial exclusion of screen-detected cancers*

For peer review only - http://bmjopen.bmj.com/site/about/guidelines.xhtml

that Norway had a significantly longer patient interval for the 90th percentile compared with Wales (74 days) (Figure 2).

INSERT FIGURE 2

The median primary care interval for symptomatic women was 0 days, except in the Ontario, Manitoba, and Victoria (Table 4). Women in these three jurisdictions had statistically significant higher likelihoods than women in Wales to have longer primary care intervals (both for more than 5 and 14 days) (Table 6).

INSERT TABLE 5

The median diagnostic interval for symptomatic women ranged from 8 days in Denmark to 29 days in Wales (Table 4). At the 90th percentile, the diagnostic interval ranged from 36 days in England to 202 days in Ontario (Table 4). Compared with Wales, all jurisdictions except Ontario had shorter diagnostic intervals (Figure 2). For women with a screen-detected breast cancer, the time intervals and pattern were similar, although screen-detected patients had significantly shorter 90th percentiles compared to symptomatic patients.

The median treatment intervals for all women ranged from 15 days in Norway and Victoria to 39 days in Manitoba. Women in England, Scotland, Ontario, and Manitoba waited more than 28 days (four weeks) for treatment after diagnosis (Table 4). Quantile regression showed smaller statistically significant differences in treatment intervals, with Manitoba and Ontario consistently having longer treatment intervals for the three percentiles (Figure 2). The treatment intervals were similar for screen-detected cancers.

The median total interval for all women from first symptom or date of screening test to start of treatment ranged from 42 days in Sweden and Victoria to 78 days in Ontario (Table 4). In Manitoba and Ontario, 25% of women had a total interval that was 30 days longer compared with Wales (81 days), whereas 25% of women in England, Northern Ireland, Denmark, Victoria, and Sweden had a total interval that was between 12 to 19 days shorter compared with Wales (Figure 2 and Table 5). 10% of women from Ontario waited 73

For peer review only - http://bmjopen.bmj.com/site/about/guidelines.xhtml

days or more from first presentation until treatment start compared with women in Wales. Including the screen-detected breast cancers made the total interval shorter, with less variation between the jurisdictions. There were differences in the time taken from women deciding to seek help and obtaining an appointment with a PCP. Obtaining an appointment 'within one week' was less often reported by women in Sweden (43%), Manitoba (50%), Ontario (58%) and Northern Ireland (64%) compared to women in other jurisdictions (above 70% in the other six jurisdiction with 84% in England and Victoria) (data not shown).

Validity tests

Comparing patient and PCP-reported screening with available screening registry data showed an almost perfect agreement (Kappa> 0.80) (Additional File 7). Comparing the dates between the different data sources showed a high agreement between all data sources (patient, PCP, CTS and registry datawhere applicable) for all types of dates (CCC = 0.94 for date of first presentation to primary care, CCC ≥ 0.94 for date of diagnosis and CCC = 0.93 for date of treatment).

Discussion

Main findings

This international survey of breast cancer patients diagnosed between 2013-15 revealed that four out of ten women were screen-detected, with the majority of these diagnosed after a visit to primary care. Three quarters of symptomatic patients had a symptom or sign indicative of breast cancer, and roughly eight in ten were urgently referred. Thus, despite the existence of screening programmes the majority still had their breast cancer diagnosed based on 'red-flag' symptoms.

The median time from women first noticing symptoms to start of treatment varied from 42 to 92 days across jurisdictions; this is attributable mainly to differences after first presentation as less variation is seen in the patient interval across jurisdictions.

For peer review only - http://bmjopen.bmj.com/site/about/guidelines.xhtml

We found that in some jurisdictions there is a 'tail' of women with excessively long waiting times from first noticing symptoms to presentation to the healthcare system.

Strengths and weaknesses

A major strength is our novel use of a standardised questionnaire survey in several countries to systematically examine routes to diagnosis and treatment. To ensure validity across jurisdictions, we drew on existing instruments and went through an extensive process of cognitive testing, piloting, translation, and adaptation [20]. Data were enriched with information from national or state/provincial-level cancer registries for screening status, date of diagnosis and tumour stage. We developed algorithms to identify screen-detected cases, place of first presentation and time intervals, and these all showed good agreement for those jurisdictions where validation was possible. Using registries and undertaking clinical validation of breast cancer patients ensured minimal selection and information biases. It also allowed exclusion of women who previously had breast cancer, thus providing a reasonably homogeneous group of patients.

We included all first-time breast cancer patients, irrespective of route to diagnosis, and assured at least 200 symptomatic cases per jurisdiction. We developed and implemented validated rules for the screening route, which showed high agreement with screening registries.

There were different classification systems for ethnicity and education across jurisdictions. We excluded these variables from the regression model to avoid introducing invalidating information bias in the model. Although this likely induced some residual confounding, it is unlikely that educational or ethnic differences could have produced the observed variation in time intervals. Indeed, we were limited in the extent we could adjust for covariates – principally because of definitional differences across jurisdictions (despite our best efforts to standardise all of the questions, terminology and definitions in the questionnaires). Further, limiting our covariates avoided small cell counts in our model (this was a prerequisite of the model).

For peer review only - http://bmjopen.bmj.com/site/about/guidelines.xhtml

Nevertheless, this was a trade-off, and there was some (albeit modest) reduction in our ability to examine the influence of covariate variables.

We used a set of rules to ensure validity and consistency, and to preserve statistical precision [20]. The validity and sensitivity analyses suggested this approach was effective. To minimise misclassification, data interpretation during data entry was reduced to an absolute minimum and all apparent data errors were checked against source data by local teams.

For peer review only

The effect of recall bias was minimised by the triangulation of data sources (e.g. patient, PCP, CTS and registry data), and by ensuring all women received the questionnaire within a limited time window after diagnosis.

The overall response rate of 40% was comparable with similar studies among patients and PCPs [30], but varied markedly between jurisdictions, with a response rate of 78% in Denmark and 24% in Norway. This may have resulted in differential selection bias across jurisdictions, something we did our best to avoid – ideally future studies would have access to more routinely available data, without the need to rely so much on self-report questionnaires. Nevertheless, the variable response rates potentially had only a modest impact on the validity of our findings; comparing the participants on a number of variables (e.g. tumour stage, presenting symptom, comorbidity, self-assessed health, smoking status) did not show any meaningful differences. Recruitment strategies did, however, differ with some women contacted by the registries (e.g. some via PCPs (and via nurses in Northern Ireland) assessing eligibility), so some further selection bias might have been introduced via differing self-selection. This is underlined by the higher-than-expected proportion of stage I tumours amongst our respondents when compared to an earlier registry-based breast cancer study [3]. Women with early stage breast cancer are likely to be more able to participate in studies of this kind compared to women with late stage disease.

For peer review only - http://bmjopen.bmj.com/site/about/guidelines.xhtml

The statistical precision of the study was sufficient, as we were able to show clinically significant differences of one week in time intervals, appropriate for clinically relevant differences.

Comparison with other studies

Median patient intervals for breast cancer have varied in previous studies – in Denmark the interval was 14 days in a 2004 study [30], and only 3 days in 2015 (using similar methods) [31], possibly reflecting cancer pathway improvements over that period. Other studies have identified the interval as 13 days (UK) [32], 14 days (New Zealand) [33] and 16 days (Germany) [34] and 7 days (Sweden) [35]. Reasons for discrepancies may reflect a difference in the time-points and data sources used to define the patient interval, or changes over time in access to primary care.

Diagnostic intervals also vary; a UK study on patients diagnosed in 1999-2000 found a median diagnostic interval of 30 days [36], while other UK studies found diagnostic intervals of 14-27 days [37,38, 39]; the lower intervals in more recent studies possibly reflect the introduction of urgent referrals in 2010. A Danish study demonstrated an 18-day interval in 2010 [39], compared to 8 days in our study [39]. Similarly, a study in Manitoba in 2009-2010 demonstrated an interval of 35 days (compared to 28 days in our study) [40]. The primary care interval has had less attention in the literature, although a Manitoban study reported an interval of 15 days (comparable with our finding of 17 days) [40]. It also reported a total interval of 78 days, which is comparable to the 76 days found in our study. A recent Swedish study focusing on the time from referral to treatment, which roughly corresponds to a combined diagnostic and treatment interval, reported a median interval of 20 days,compared to 35 days in our study.[35]

Clinical referral for a suspected breast cancer (e.g., lump) is often expedited [37,38]. Thus, the high proportion of women with 'red-flag' symptoms or signs may mean that time intervals for breast cancer diagnosis vary less than other common cancers, as differential diagnosis is easier and investigations and referral pathways more straightforward. Nevertheless, we found important variations in intervals for

For peer review only

symptomatic women, possibly reflecting methodological differences, but we also noted temporal and international differences in diagnostic pathways. These differences are expected to primarily impact on the median primary care interval (0 days for all jurisdictions except Manitoba, Ontario and Victoria). Note, however, that in these jurisdictions primary care is more often responsible for confirming the breast cancer diagnosis, thus extending the interval. Indeed, a study from Manitoba confirms that PCPs experience significant waiting time when they order a mammogram [40]. We believe our findings not only highlight international differences in diagnostic intervals (which suggests potential for interval reduction), but also provide important data for health care planners in individual participating jurisdictions. For example, what are the implications of an above-average primary care interval? Does it reflect education and training needs, access to investigations, referral difficulties or other critical interval determinants?

Conclusion and implications for clinical practice and healthcare organisation

Despite screening programmes, the diagnostic route for breast cancer remains critically important, as over half of patients are diagnosed this way. Awareness and recognition of warning signs which impact on patient intervals is especially important [41] as the current study shows a significant number of women wait more than 90 days before presenting with symptoms. Targeted awareness interventions could facilitate more timely diagnosis of breast cancer. The variation in waiting times for an appointment in primary care, which might affect perceived access, could also be addressed.

The precise impact of variations in routes to diagnosis, diagnostic/treatment intervals and key outcomes (e.g., disease-free survival and mortality) cannot be assessed from this study. There is, nevertheless, growing evidence of an association between time intervals, the use of urgent referrals and mortality [10,41- 44]. More research is needed to explore this relationship.

This study illustrates the need to optimise diagnostic routes for breast cancer internationally. Awareness of international differences in key time intervals is an important step in optimising pathways. Ideally, routine,

For peer review only - http://bmjopen.bmj.com/site/about/guidelines.xhtml

For peer review only

386 standardised collection of time interval and route to diagnostic information will become the norm

387 internationally as this this will greatly assist in optimisation of breast cancer diagnostic pathways.

For peer review only

For peer review only - http://bmjopen.bmj.com/site/about/guidelines.xhtml

List of abbreviations

ICBP M4 – International Cancer Benchmarking Partnership Module 4

PCP – primary care physician

TNM – tumour, node, metastasis

CTS – cancer treatment specialist

IARC – International Agency for Research on Cancer

Declarations

Ethics approval and consent to participate

For each local data collection, there were specific procedures and approvals. The approvals also included anonymised data transfer to University College London and Aarhus University. Approvals were received from the following institutions: Cancer Council Victoria Human Research Ethics Committee [HREC 1125]; Health Research Ethics Board, University of Manitoba [HS15227 (H2012:105)]; Research Resource Ethics Committee, CancerCare Manitoba [RRIC#28-2012]; University of Toronto Research Ethics Board [27881]; The Danish Data Protection Agency [2013-41-2030]; Swedish Ethics Review Board, Uppsala [2013/306]; Norway Regional committees for medical and health research ethics [2013/136/REK nord]; England, Wales and Scotland, NRES Committee East Midlands – Derby 2, local R&D for each health board [11/EM/0420]; Northern Ireland ORECNI Ethical approval, local governance for each health Trust [11/EM/0420].

Consent for publication

Not Applicable

Availability of data and material

The data that support the findings of this study are available from the named authors from each ICBP jurisdiction, but restrictions apply to the availability of these data and so are not publicly available. Data are however available from the authors upon reasonable request and with permission of the ICBP Programme

For peer review only

Board. Please contact the ICBP Programme Management team, based at Cancer Research UK, with any queries (icbp@cancer.org.uk).

Competing interests

The authors declare that they have no competing interests.

Funding

Funding for ICBP Module 4 was provided by: CancerCare Manitoba; Cancer Care Ontario; Canadian Partnership Against Cancer (CPAC); Cancer Council Victoria; Cancer Research Wales; Cancer Research UK; Danish Cancer Society; Danish Health and Medicines Authority; European Palliative Care Research Centre (PRC), Norwegian University of Science and Technology (NTNU); Guidelines Audit and Implementation Network (GAIN); Macmillan Cancer Support; National Cancer Action Team; NHS England; Northern Ireland Cancer Registry, funded by the Public Health Agency NI; Norwegian Directorate of Health; Research Centre for Cancer Diagnosis in Primary Care (CaP), Aarhus University, Denmark; Scottish Government; Swedish Association of Local Authorities and Regions; University College London and NIHR Biomedical Research Centre at University College London NHS Foundation Trust; University of Edinburgh (R42856); Victorian Department of Health and Human Services; Welsh Government.

The funding bodies had no influence on the design of the study and collection, analysis, and interpretation of data, in writing the manuscript or whether to publish the results.

Authors' contributions

- **Usha Menon** - planned the study design, data collection, carried out the analyses and wrote the draft manuscript. Also responsible for local data collection (alongside the Working Group), management and interpretation, and have participated in writing and approving the final manuscript version.

For peer review only

- **David Weller** - planned the study design, data collection, carried out the analyses and wrote the draft manuscript. Also responsible for local data collection (alongside the Working Group), management and interpretation, and have participated in writing and approving the final manuscript version.
- **Alina Zalounina Falborg** - planned the study design, data collection, carried out the analyses and wrote the draft manuscript
- **Henry Jensen** - planned the study design, data collection, carried out the analyses and wrote the draft manuscript. Also responsible for local data collection (alongside the Working Group), management and interpretation, and have participated in writing and approving the final manuscript version.
- **Peter Vedsted**- planned the study design, data collection, carried out the analyses and wrote the draft manuscript. Also responsible for local data collection (alongside the Working Group), management and interpretation, and have participated in writing and approving the final manuscript version.
- **Jatinderpal Kalsi** - Responsible for local data collection (alongside the ICBP Module 4Working Group), management and interpretation, and have participated in writing and approving the final manuscript version.
- **David H Brewster** - Responsible for local data collection (alongside the ICBP Module 4Working Group), management and interpretation, and have participated in writing and approving the final manuscript version.
- **Yulan Lin** - Responsible for local data collection (alongside the ICBP Module 4Working Group), management and interpretation, and have participated in writing and approving the final manuscript version.
- **Anna Gavin** - Responsible for local data collection (alongside the ICBP Module 4Working Group), management and interpretation, and have participated in writing and approving the final manuscript version.
- **Eva Grunfeld** - Responsible for local data collection (alongside the ICBP Module 4Working Group), management and interpretation, and have participated in writing and approving the final manuscript version.
- **Mats Lambe** - Responsible for local data collection (alongside the ICBP Module 4Working Group), management and interpretation, and have participated in writing and approving the final manuscript version.
- **Martin Malmberg** - Responsible for local data collection (alongside the ICBP Module 4Working Group), management and interpretation, and have participated in writing and approving the final manuscript version.
- **Donna Turner** - Responsible for local data collection (alongside the ICBP Module 4Working Group), management and interpretation, and have participated in writing and approving the final manuscript version.

For peer review only

- **Elizabeth Harland** - Responsible for local data collection (alongside the ICBP Module 4Working Group), management and interpretation, and have participated in writing and approving the final manuscript version.
- **Breann Hawryluk** - Responsible for local data collection (alongside the ICBP Module 4Working Group), management and interpretation, and have participated in writing and approving the final manuscript version.
- **Rebecca-Jane Law** - Responsible for local data collection (alongside the ICBP Module 4Working Group), management and interpretation, and have participated in writing and approving the final manuscript version.
- **Richard D Neal** - Responsible for local data collection (alongside the ICBP Module 4Working Group), management and interpretation, and have participated in writing and approving the final manuscript version.
- **Victoria White** - Responsible for local data collection (alongside the ICBP Module 4Working Group), management and interpretation, and have participated in writing and approving the final manuscript version.
- **Rebecca J Bergin** - Responsible for local data collection (alongside the ICBP Module 4Working Group), management and interpretation, and have participated in writing and approving the final manuscript version.
- **Samantha Harrison** - Responsible for local data collection (alongside the ICBP Module 4Working Group), management and interpretation, and have participated in writing and approving the final manuscript version.

Acknowledgements

We thank our colleagues and participating patients, primary care physicians and cancer treatment specialists in all participating ICBP jurisdictions. Catherine Foot, Martine Bomb, Brad Groves, Irene Reguilon, Charlotte Lynch and Samantha Harrison of Cancer Research UK for managing the programme. We also thank John Butler, Royal Marsden NHS Foundation Trust and ICBP Clinical Lead, for his advice; and the ICBP M4 academic reference group for providing independent peer review and advice for the study protocol and analysis plan development. We would like to thank staff at the Information Services Division of NHS National Services Scotland for identifying potential study participants in Scotland, and mailing patient questionnaires to primary care physicians; as well as theDanish Breast Cancer Group (DBCG) for providing secondary care (specialist) data to this study.

For peer review only - http://bmjopen.bmj.com/site/about/guidelines.xhtml

The ICBP Module 4 working Group:

- Alina Zalounina Falborg, Statistician, Research Unit for General Practice, Department of Public Health, Aarhus University, Bartholins Allé 2, 8000 Aarhus C, Denmark;
- Andriana Barisic, Research Associate, Department of Prevention and Cancer Control, Cancer Care Ontario, 620 University Avenue, Toronto, Ontario, M5G 2L7, Canada;
- Anna Gavin, Director, Northern Ireland Cancer Registry, Centre for Public Health, Queen's University Belfast, Mulhouse Building, Mulhouse Road, Belfast, BT12 6DP, United Kingdom;
- Anne Kari Knudsen, Administrative leader, Department of Cancer Research and Molecular Medicine, Norwegian University of Science and Technology, 7489 Trondheim, Norway;
- Breann Hawryluk, Project Planning Coordinator, Department of Patient Navigation, Cancer Care Manitoba, 675 McDermot Street, Winnipeg, Manitoba, Canada;
- Chantelle Anandan, Research Fellow, Centre for Population Health Sciences, University of Edinburgh, Doorway 1, Medical Quad Teviot Place, Edinburgh, EH8 9DX, United Kingdom;
- Conan Donnelly, Research Fellow, Centre for Public Health, Queen's University Belfast, Mulhouse Building, Mulhouse Road, Belfast, BT12 6DP, United Kingdom;
- David H Brewster, Scottish Cancer Registry, Information Services Division, NHS National Services Scotland, Gyle Square, 1 South Gyle Crescent, Edinburgh, EH12 9EB, United Kingdom;
- David Weller, James Mackenzie Professor of General Practice, Centre for Population Health Sciences, University of Edinburgh, Doorway 1, Medical Quad Teviot Place, Edinburgh, EH8 9DX, United Kingdom;
- Donna Turner, Epidemiologist/Provincial Director, Population Oncology, Cancer Care Manitoba, 675 McDermot Street, Winnipeg, Manitoba, Canada;
- Elizabeth Harland, Project Coordinator, Department of Epidemiology and Cancer Registry, CancerCare Manitoba, 675 McDermot Street, Winnipeg, Manitoba;
- Eva Grunfeld, Director, Knowledge Translation Research Network Health Services Research Program, Ontario Institute for Cancer Research; Professor and Vice Chair Research, Department of Family and Community Medicine, University of Toronto, 500 University Avenue, Toronto, Ontario, M5G 1V7, Canada;
- Evangelia Ourania Fourkala, Research Associate, Gynaecological Cancer Research Centre, Women's Cancer, Institute for Women's Health, University College London, United Kingdom;
- Henry Jensen, Research fellow, Research Unit for General Practice, Department of Public Health, Aarhus University, Bartholins Allé 2, 8000 Aarhus C, Denmark;
- Jackie Boylan, Research Fellow, Centre for Public Health, Queen's University Belfast, Mulhouse Building, Mulhouse Road, Belfast, BT12 6DP, United Kingdom;
- Jacqueline Kelly, Tumour Verification Officer, Northern Ireland Cancer Registry, Centre for Public Health, Queen's University Belfast, Mulhouse Building, Mulhouse Road, Belfast, BT12 6DP, United Kingdom;
- Kerry Moore, Research Fellow, Centre for Public Health, Queen's University Belfast, Mulhouse Building, Mulhouse Road, Belfast, BT12 6DP, United Kingdom;
- Maria Rejmyr Davis, Head, Southern Sweden Regional Cancer Center, Medicon Village, Scheelevägen 8, building 404, 223 81 Lund, Sweden;
- Martin Malmberg, MD PhD, Senior Consultant, Department of Oncology, Lund University Hospital, SE-221 85 Lund, Sweden;
- Mats Lambe, Professor of Medical Epidemiology, Regional Cancer Center Uppsala and Department of Medical Epidemiology and Biostatics, Karolinska Institutet, SE-171 77 Stockholm, Sweden;

For peer review only - http://bmjopen.bmj.com/site/about/guidelines.xhtml

- Oliver Bucher, Epidemiologist, Department of Epidemiology and Cancer Registry, CancerCare Manitoba, 675 McDermot Street, Winnipeg, Manitoba;
- Peter Vedsted, Professor, Research Unit for General Practice, Department of Public Health, Aarhus University, Bartholins Allé 2, 8000 Aarhus C, Denmark;
- Rebecca Bergin, Senior Research Officer/ PhD Candidate, Centre for Behavioural Research in Cancer, 615 St Kilda Rd, Melbourne, Victoria, 3004, Australia;
- Rebecca-Jane Law, Research Project Support Officer, North Wales Centre for Primary Care Research, Bangor University, Gwenfro Units 4-8, Wrexham Technology Park, Wrexham, LL13 7YP, United Kingdom;
- Richard D Neal, Professor of Primary Care Oncology, Academic Unit of Primary Care, Leeds Institute of Health Sciences, University of Leeds, Leeds LS2 9NL, United Kingdom;
- Sigrun Saur Almberg, Researcher, Department of Cancer Research and Molecular Medicine, Faculty of Medicine, Norwegian University of Science and Technology (NTNU), N-7491 Trondheim, Norway;
- Therese Kearney, Research fellow, Northern Ireland Cancer Registry, Centre for Public Health, Queen's University Belfast, Mulhouse Building, Mulhouse Road, Belfast, BT12 6DP, United Kingdom;
- Jatinderpal Kalsi, Project Manager, Gynaecological Cancer Research Centre, Women's Cancer, Institute for Women's Health, University College London, United Kingdom;
- Victoria Cairnduff, Statistician, Northern Ireland Cancer Registry, Centre for Public Health, Queen's University Belfast, Mulhouse Building, Mulhouse Road, Belfast, BT12 6DP, United Kingdom;
- Victoria Hammersley, Researcher, Centre for Population Health Sciences, University of Edinburgh, Doorway 1, Medical Quad Teviot Place, Edinburgh, EH8 9DX, United Kingdom;
- Victoria White, Deputy Director, Centre for Behavioral Research in Cancer, Cancer Council Victoria, 615 St Kilda Road, Melbourne, Victoria, 3004, Australia;
- Usha Menon, Professor of Gynaecological Oncology and Head, Gynaecological Cancer Research Centre, Women's Cancer, Institute for Women's Health, University College London, United Kingdom;
- Yulan Lin, Postdoc, Department of Cancer Research and Molecular Medicine, Faculty of Medicine, Norwegian University of Science and Technology (NTNU), N-7491 Trondheim, Norway

ICBP Module 4 Academic Reference Group:

- Prof Jan Willem Coebergh, Professor of Cancer Surveillance, Department of Public Health, Erasmus Universiteit Rotterdam, Rotterdam, the Netherlands;
- Jon Emery, Professor of Primary Care Cancer Research, University of Melbourne and Clinical Professor of General Practice, University of Western Australia, Australia;
- Dr Stefan Bergström, Senior consultant oncologist, Department of Oncology, Gävle, Sweden;
- Dr Monique E van Leerdam, Erasmus MC University Medical Centre, the Netherlands;
- Prof Marie-Louise Essink-Bot, Academic Medical Centre, Amsterdam University, the Netherlands;
- Prof Una MacLeod, Senior Lecturer in General Practice and Primary Care, Hull-York Medical School, United Kingdom.

For peer review only - http://bmjopen.bmj.com/site/about/guidelines.xhtml

For peer review only

For peer review only - http://bmjopen.bmj.com/site/about/guidelines.xhtml

References

1. Global Burden of Disease Cancer C, Fitzmaurice C, Dicker D, Pain A, Hamavid H, Moradi-Lakeh M, MacIntyre MF, Allen C, Hansen G, Woodbrook R, et al: The Global Burden of Cancer 2013. JAMA Oncol 2015, 1:505-527.

2. Allemani C, Matsuda T, Di Carlo V, Harewood R, Matz M, Nikšić M, Bonaventure A, Valkov M, Johnson CJ, Estève J, et al: Global surveillance of trends in cancer survival 2000-14 (CONCORD-3): analysis of individual records for 37 513 025 patients diagnosed with one of 18 cancers from 322 population-based registries in 71 countries. Lancet 2018, S0140-6736(17)33326-3. [Epub ahead of print].

3. Walters S, Maringe C, Butler J, Rachet B, Barrett-Lee P, Bergh J, Boyages J, Christiansen P, Lee M, Warnberg F, et al: Breast cancer survival and stage at diagnosis in Australia, Canada, Denmark, Norway, Sweden and the UK, 2000-2007: a population-based study. Br J Cancer 2013, 108:1195-1208.

4. Coleman MP, Forman D, Bryant H, Butler J, Rachet B, Maringe C, Nur U, Tracey E, Coory M, Hatcher J, et al: Cancer survival in Australia, Canada, Denmark, Norway, Sweden, and the UK, 1995-2007 (the International Cancer Benchmarking Partnership): an analysis of population-based cancer registry data. Lancet 2011, 377:127-138.

5. Niksic M, Rachet B, Duffy SW, Quaresma M, Moller H, Forbes LJ: Is cancer survival associated with cancer symptom awareness and barriers to seeking medical help in England? An ecological study. Br J Cancer 2016, 115:876-886.

6. Yun YH, Kim YA, Min YH, Park S, Won YJ, Kim DY, Choi IJ, Kim YW, Park SJ, Kim JH, et al: The influence of hospital volume and surgical treatment delay on long-term survival after cancer surgery. Ann Oncol 2012, 23:2731-2737.

7. Smith EC, Ziogas A, Anton-Culver H. Delay in surgical treatment and survival after breast cancer diagnosis in young women by race/ethnicity. JAMA Surg. 2013;148:516-23.

8. Torring ML, Frydenberg M, Hamilton W, Hansen RP, Lautrup MD, Vedsted P: Diagnostic interval and mortality in colorectal cancer: U-shaped association demonstrated for three different datasets. J Clin Epidemiol 2012, 65:669-678.

9. Torring ML, Frydenberg M, Hansen RP, Olesen F, Vedsted P: Evidence of increasing mortality with longer diagnostic intervals for five common cancers: a cohort study in primary care. Eur J Cancer 2013, 49:2187-2198.

10. Neal RD, Tharmanathan P, France B, Din NU, Cotton S, Fallon-Ferguson J, Hamilton W, Hendry A, Hendry M, Lewis R, et al: Is increased time to diagnosis and treatment in symptomatic cancer associated with poorer outcomes? Systematic review. Br J Cancer 2015, 112 Suppl 1:S92-107.

11. Forbes LJ, Simon AE, Warburton F, Boniface D, Brain KE, Dessaix A, Donnelly C, Haynes K, Hvidberg L, Lagerlund M, et al: Differences in cancer awareness and beliefs between Australia, Canada, Denmark, Norway, Sweden and the UK (the International Cancer Benchmarking Partnership): do they contribute to differences in cancer survival? Br J Cancer 2013, 108:292-300.

12. Koo MM, von Wagner C, Abel GA, McPhail S, Rubin GP, Lyratzopoulos G: Typical and atypical presenting symptoms of breast cancer and their associations with diagnostic intervals: Evidence from a national audit of cancer diagnosis. Cancer Epidemiol 2017, 48:140-146.

For peer review only

13. Brown S, Castelli M, Hunter DJ, Erskine J, Vedsted P, Foot C, Rubin G: How might healthcare systems influence speed of cancer diagnosis: a narrative review. Soc Sci Med 2014, 116:56-63.

14. Lyratzopoulos G, Vedsted P, Singh H: Understanding missed opportunities for more timely diagnosis of cancer in symptomatic patients after presentation. Br J Cancer 2015, 112 Suppl 1:S84-91.

15. Rubin G, Berendsen A, Crawford SM, Dommett R, Earle C, Emery J, Fahey T, Grassi L, Grunfeld E, Gupta S, et al: The expanding role of primary care in cancer control. Lancet Oncol 2015, 16:1231-1272.

16. Rose PW, Rubin G, Perera-Salazar R, Almberg SS, Barisic A, Dawes M, Grunfeld E, Hart N, Neal RD, Pirotta M, et al: Explaining variation in cancer survival between 11 jurisdictions in the International Cancer Benchmarking Partnership: a primary care vignette survey. BMJ Open 2015, 5:e007212.

17. National Institute for Health and Care Excellence. Suspected cancer: recognition and referral; NICE Guideline [NG12]. National Institute for Health Care Excellence, 2015. Available at: https://www.nice.org.uk/guidance/ng12 [accessed 05 October 2017]

18. Murchie P, Campbell NC, Delaney EK, Dinant GJ, Hannaford PC, Johansson L, Lee AJ, Rollano P, Spigt M: Comparing diagnostic delay in cancer: a cross-sectional study in three European countries with primary care-led health care systems. Fam Pract 2012, 29:69-78.

19. Butler J, Foot C, Bomb M, Hiom S, Coleman M, Bryant H, Vedsted P, Hanson J, Richards M, Group IW: The International Cancer Benchmarking Partnership: an international collaboration to inform cancer policy in Australia, Canada, Denmark, Norway, Sweden and the United Kingdom. Health Policy 2013, 112:148-155.

20. Weller D, Vedsted P, Anandan C, Zalounina A, Fourkala EO, Desai R, Liston W, Jensen H, Barisic A, Gavin A, et al: An investigation of routes to cancer diagnosis in 10 international jurisdictions, as part of the International Cancer Benchmarking Partnership: survey development and implementation. BMJ Open 2016, 6:e009641.

21. European Network of Cancer Registries. Standards and guidelines for cancer registration in Europe: the ENCR recommendations. Edited by Tyczynski JE, Démaret E, Parkin DM. (IARC technical publication; no. 40), Lyon, 2003.

22. TNM classification of malignant tumours. Eds. L.H. Sobin, M.K. Gospodarowicz and Ch. Wittekind. — 7th ed. UICC International Union Against Cancer. ISBN 978-1-4443-3241-4

23. Weller D, Vedsted P, Rubin G, Walter FM, Emery J, Scott S, Campbell C, Andersen RS, Hamilton W, Olesen F, et al: The Aarhus statement: improving design and reporting of studies on early cancer diagnosis. Br J Cancer 2012, 106:1262-1267.

24. Macefield RC, Jacobs M, Korfage IJ, Nicklin J, Whistance RN, Brookes ST, et al. Developing core outcomes sets: methods for identifying and including patient-reported outcomes (PROs). Trials. 2014 Feb 5;15:49.

25. Danish Cancer Society. Suspicion of breast cancer criteria for referral for general practice. Danish Cancer Society, 2016. Available at: https://www.sst.dk/da/udgivelser/2016/~/media/4864545DA763400E9FD988FA4DA74EE5.ashx accessed 05 October 2017

26. Koenker R, Bassett G. Regression quantiles. *Econometrica* 1978; 46:33-50

For peer review only - http://bmjopen.bmj.com/site/about/guidelines.xhtml

27. Machado JAF, Santos Silva JMC. Quantiles for Counts. J Amer Statistical Assoc 2005, 100(472): 1226-1237.

28. Miranda A. QCOUNT: Stata program to fit quantile regression models for count data. Boston: Boston College Department of Economics; 2006

29. Lin LI. A concordance correlation coefficient to evaluate reproducibility. Biometrics 1989;45((1) March):255–68

30. Hansen RP, Vedsted P, Sokolowski I, Sondergaard J, Olesen F: Time intervals from first symptom to treatment of cancer: a cohort study of 2,212 newly diagnosed cancer patients. BMC Health Serv Res 2011, 11:284.

31. Olesen F, Hansen RP, Vedsted P: Delay in diagnosis: the experience in Denmark. Br J Cancer 2009, 101 Suppl 2:S5-8.

32. Nosarti C, Crayford T, Roberts JV, Elias E, McKenzie K, David AS: Delay in presentation of symptomatic referrals to a breast clinic: patient and system factors. Br J Cancer 2000, 82:742-748.

33. Meechan G, Collins J, Petrie K: Delay in seeking medical care for self-detected breast symptoms in New Zealand women. N Z Med J 2002, 115:U257.

34. Arndt V, Sturmer T, Stegmaier C, Ziegler H, Dhom G, Brenner H: Patient delay and stage of diagnosis among breast cancer patients in Germany -- a population based study. Br J Cancer 2002, 86:1034-1040.

35. Thulesius HO, Lindgren AC, Olsson HL, Hakansson A: Diagnosis and prognosis of breast and ovarian cancer--a population-based study of 234 women. Acta Oncol 2004, 43:175-181.

36. Allgar VL, Neal RD: Delays in the diagnosis of six cancers: analysis of data from the National Survey of NHS Patients: Cancer. Br J Cancer 2005, 92:1959-1970.

37. Din NU, Ukoumunne OC, Rubin G, Hamilton W, Carter B, Stapley S, Neal RD: Age and Gender Variations in Cancer Diagnostic Intervals in 15 Cancers: Analysis of Data from the UK Clinical Practice Research Datalink. PLoS One 2015, 10:e0127717.

38. Redaniel MT, Martin RM, Ridd MJ, Wade J, Jeffreys M: Diagnostic intervals and its association with breast, prostate, lung and colorectal cancer survival in England: historical cohort study using the Clinical Practice Research Datalink. PLoS One 2015, 10:e0126608.

39. Jensen H, Torring ML, Olesen F, Overgaard J, Fenger-Gron M, Vedsted P: Diagnostic intervals before and after implementation of cancer patient pathways - a GP survey and registry based comparison of three cohorts of cancer patients. BMC Cancer 2015, 15:308.

40. Nashed M, Carpenter-Kellett T, Lambert P, Musto G, Turner D, Cooke A: Wait Time from Suspicion to Surgery for Breast Cancer in Manitoba. Cureus 2016, 8:e680.

41. Quaife SL, Forbes LJ, Ramirez AJ, Brain KE, Donnelly C, Simon AE, Wardle J: Recognition of cancer warning signs and anticipated delay in help-seeking in a population sample of adults in the UK. Br J Cancer 2014, 110:12-18.

42. Richards MA, Westcombe AM, Love SB, Littlejohns P, Ramirez AJ. Influence of delay on survival in patients with breast cancer: a systematic review. Lancet. 1999 Apr 3;353(9159):1119-26.

For peer review only - http://bmjopen.bmj.com/site/about/guidelines.xhtml

43. Moller H, Gildea C, Meechan D, Rubin G, Round T, Vedsted P. Use of the English urgent referral pathway for suspected cancer and mortality in patients with cancer: cohort study. BMJ. 2015 Oct 13;351:h5102. doi: 10.1136/bmj.h5102.

44. Murchie P, Raja EA, Lee AJ, Brewster DH, Campbell NC, Gray NM, Ritchie LD, Robertson R, Samuel L: Effect of longer health service provider delays on stage at diagnosis and mortality in symptomatic breast cancer. Breast 2015, 24:248-255.

For peer review only

For peer review only

For peer review only - http://bmjopen.bmj.com/site/about/guidelines.xhtml

Table 1. *The characteristics of the 3,471 women (40+) with a first diagnosis of breast cancer (% if nothing else stated)*

	**Wales (N=232)**	**England (N=363)**	**Scotland (N=371)**	**N Ireland (N=331)**	**Denmark (N=369)**	**Ontario (N=403)**	**Norway (N=372)**	**Manitoba (N=368)**	**Victoria (N=370)**	**Sweden (N=292)**	**Total (N=3471)**
**Age** years											
Median (IQI)	63 (53,70)	63 (53,70)	63 (54,70)	60 (51,70)	64 (55,71)	63 (55,69)	61 (52, 69)	63 (55,72)	58 (50,67)	63 (54,71)	62 (53,70)
**Age** years (Symptomatic women)	(N=115)	(N=207)	(N=208)	(N=242)	(N=231)	(N=201)	(N=235)	(N=205)	(N=198)	(N=120)	(N=1962)
Median (IQI)	62 (49,76)	62 (50,75)	62 (52,75)	60 (49,72)	68 (54,77)	61 (53,69)	61 (50, 73)	62 (50,75)	56 (48,68)	68 (54,77)	62 (50,74)
**Age** years (Screen-detected women	(N=116)	(N=155)	(N=162)	(N=86)	(N=134)	(N=198)	(N=132)	(N=162)	(N=172)	(N=171)	(N=1488)
Median (IQI)	63 (58,68)	64 (58,68)	63 (58,67)	60 (54,67)	62 (56,66)	65 (59,69)	62 (57,67)	65 (59,70)	61 (53,67)	61 (54,69)	63 (57,68)
**Health State**											
Good	81.0	81.0	79.3	77.7	78.1	90.3	74.7	85.6	88.4	80.8	81.8
Fair	14.7	13.0	13.7	14.2	14.6	8.2	17.5	9.2	9.5	12.3	12.6
Poor	3.5	4.7	5.1	5.4	3.5	1	6.2	2.7	1.6	3.8	3.7
Missing	0.9	1.3	1.9	2.7	3.8	0.5	1.6	2.5	0.5	3.1	1.9
**Comorbidity**[1]											
No	74.1	75.1	69.0	78.6	62.9	61.3	73.9	70.4	76.5	80.5	71.8
Medium	24.6	23.1	29.4	19.9	34.1	25.1	23.7	27.7	23.2	16.1	25.0
High	0.9	0.8	1.1	0.9	0.8	0.0	1.1	0.5	0.0	0.3	0.6
Missing	0.4	0.8	0.5	0.6	2.2	13.6	1.3	1.4	0.3	3.1	2.6
**Education**											
Low	71.6	78.2	69.0	77.0	71.8	64.3	63.7	74.7	70.0	68.8	70.8
High	22.4	14.9	19.7	16.3	14.6	33.5	31.5	19.8	28.9	28.8	23.1
Missing	6.0	6.9	11.3	6.7	13.6	2.2	4.8	5.4	1.1	2.4	6.1
**Ethnicity**											
White	97.9	97.8	98.9	99.1	94.3	89.9	98.7	85.6	97.3	96.2	95.4
Asian	0.4	1.1	0.0	0.0	1.1	5.7	0.8	7.6	1.9	1.0	2.1
Black	0.4	0.8	0.3	0.0	0.0	2.0	0.0	0.5	0.0	0.0	0.4
Other	0.0	0.0	0.0	0.0	0.0	1.2	0.0	6.0	0.0	0.0	0.8
Missing	1.3	0.3	0.8	0.9	4.6	1.2	0.5	0.3	0.8	2.8	1.3
**Smoking**											
Currently	7.3	7.2	8.4	11.8	15.2	4.0	15.1	7.9	5.7	19.6	9.2
In the past	35.3	38.0	41.2	33.2	41.7	43.9	40.9	44.6	36.7	41.1	39.9
Never	56.5	54.5	49.3	53.5	40.9	51.6	43.5	46.2	57.3	46.9	49.8
Missing	0.9	0.3	1.1	1.5	2.2	0.5	0.5	1.3	0.3	2.4	1.1
**Tumour stage – TNM** (all women)											
0	1.7	0.6	0.0	0.0	0.3	0.3	0.0	0.0	0.3	0.0	0.3
I	50.4	44.9	47.4	45.3	45.0	51.6	11.3	50.8	52.7	62.0	45.7
II	37.9	36.4	35.6	35.1	33.1	38.0	4.6	34.5	34.6	31.5	31.9
III	5.6	8.3	7.8	13.6	9.5	8.2	1.9	10.3	10.8	4.1	8.1
IV	0.9	3.0	4.6	2.7	2.4	1.0	0.8	1.6	1.1	0.7	1.9
Missing	3.5	6.9	4.5	3.3	9.8	1.0	81.5	2.7	0.5	1.7	12.1

For peer review only

	Wales (N=232)	England (N=363)	Scotland (N=371)	N Ireland (N=331)	Denmark (N=369)	Ontario (N=403)	Norway (N=372)	Manitoba (N=368)	Victoria (N=370)	Sweden (N=292)	Total (N=3471)
**Tumour stage – TNM** (Symptomatic women)	(N=115)	(N=207)	(N=208)	(N=242)	(N=231)	(N=201)	(N=235)	(N=205)	(N=198)	(N=120)	(N=1966)
0	1.7	1.0	0.0	0.0	0.0	0.0	0.0	0.0	0.5	0.0	0.3
I	33.0	34.8	32.7	37.2	36.4	32.8	8.1	32.7	37.9	40.8	32.1
II	49.6	41.6	45.7	39.3	35.1	50.0	5.5	48.3	44.4	50.8	39.5
III	8.7	10.6	9.6	17.4	11.7	14.0	2.6	13.2	15.2	3.3	11.0
IV	1.7	4.4	6.7	2.9	3.9	2.0	0.9	2.4	1.0	1.7	2.9
Missing	5.2	7.7	5.3	3.3	13.0	0.5	83.0	3.4	1.0	3.3	14.3
**Tumour stage – TNM** (Screen-detected women)	(N=116)	(N=155)	(N=162)	(N=86)	(N=134)	(N=198)	(N=132)	(N=162)	(N=172)	(N=171)	(N=1484)
0	1.7	0.0	0.0	0.0	0.8	0.5	0.0	0.0	0.0	0.0	0.3
I	68.1	58.1	66.1	67.4	59.7	69.2	16.7	74.1	69.8	77.2	63.5
II	25.9	29.7	22.8	23.3	29.1	26.3	2.3	17.3	23.3	18.1	21.9
III	2.6	5.2	5.6	3.5	6.0	2.5	0.8	6.8	5.8	4.1	4.4
IV	0.0	1.3	1.9	2.3	0.0	0.0	0.0	0.6	1.2	0.0	0.7
Missing	1.7	5.8	3.7	3.5	4.5	1.5	80.3	1.2	0.0	0.6	9.3

[1] Comorbidity coded as none=no reported, medium=1-2 reported and high=3+ reported
Abbreviations: IQI=inter-quartile interval
Norwegian data on tumour stage were incomplete, resulting in a high number of missing stage data.

For peer review only - http://bmjopen.bmj.com/site/about/guidelines.xhtml

Table 2. Proportion of screen-detected or symptomatic patients (%), and place of initial symptomatic presentation across jurisdictions

	Wales (N=232)	England (N=363)	Scotland (N=371)	N Ireland[1] (N=331)	Denmark (N=369)	Ontario (N=403)	Norway (N=373)	Manitoba (N=368)	Victoria (N=370)	Sweden (N=292)	Total (N=3471)
**Symptomatic**	**49.6**	**57.0**	**56.1**	**73.1**	**62.6**	**49.9**	**63.2**	**55.7**	**53.5**	**41.1**	**56.5**
Visit PCP, Visit PCP and then A&E[2]	*91.3*	*93.7*	*92.3*	*88.0*	*84.4*	*69.2*	*81.3*	*88.3*	*90.9*	*75.0*	*85.6*
A&E directly[2]	*1.7*	*1.0*	*0.5*	*0.4*	*1.3*	*2.5*	*0.9*	*1.0*	*0.5*	*0.8*	*1.0*
Investigation for another problem[2]	*0.9*	*1.0*	*4.8*	*1.2*	*6.1*	*7.5*	*4.7*	*2.4*	*1.5*	*10.0*	*3.9*
Other[2]	*6.1*	*4.3*	*2.4*	*10.3*	*8.2*	*20.9*	*13.2*	*8.3*	*7.1*	*14.2*	*9.5*
**Screening**	**50.0**	**42.7**	**43.7**	**26.0**	**36.3**	**49.1**	**35.5**	**44.0**	**46.5**	**58.6**	**42.9**
**Other**	**0.4**	**0.3**	**0.3**	**0.9**	**1.1**	**1.0**	**1.2**	**0.3**	**0.0**	**0.3**	**0.6**

[1] In N Ireland the proportion of screen detected breast cancers was lower as many of these women were excluded from the eligible group in the start of the inclusion

[2] Percentage of the symptomatic route

For peer review only

Table 3. *The first symptom(s) reported by women[1] and the first presenting symptom(s) reported by PCPs (%)*

	**Wales**	**England**	**Scotland**	**N Ireland[1]**	**Denmark**	**Ontario**	**Norway**	**Manitoba**	**Victoria**	**Sweden[2]**	**Total**
First symptom *(reported by women)*	*(N=115)*	*(N=207)*	*(N=208)*	*(N=242)*	*(N=231)*	*(N=201)*	*(N=235)*	*(N=205)*	*(N=198)*	*(N=120)*	*(N=1962)*
Lump/swelling/thickening of breast	76	70	75	62	63	63	55	60	71	73	66
Change in the nipple	9	15	11	14	11	10	13	6	6	14	11
Fatigue	16	10	9	12	5	17	11	12	14	9	11
Change in the size, contour	12	13	10	10	5	16	8	10	15	11	11
Pain/tenderness in the breast	6	5	4	14	3	5	7	9	15	6	8
Flattening/indentation in the breast	5	5	0.5	4	4	0	2	2	0	0.8	2
Other	21	23	20	17	10	13	18	19	16	12	17
No symptoms	8	2	4	8	11	10	9	17	5	10	8
Missing	0	0.4	4	6	10	2	13	4	1	0	5
Presenting symptom *(reported by PCP)*	*(N=97)*	*(N=169)*	*(N=160)*	*(N=0)*	*(N=153)*	*(N=74)*	*(N=109)*	*(N=123)*	*(N=128)*	*(N=0)*	*(N=1013)*
Lump/swelling/thickening of breast	73	64	64	n/a	67	34	55	46	64	n/a	60
Change in the nipple	2	8	5	n/a	5	7	5	0.8	6	n/a	5
Fatigue	0	0	0	n/a	0	0	0.9	0.8	0	n/a	0.2
Change in the size, contour	4	4	0	n/a	6	0	0.9	3	5	n/a	3
Pain/tenderness in the breast	10	6	4	n/a	7	7	4	10	13	n/a	7
Flattening/indentation in the breast	2	2	1	n/a	0	3	2	3	0	n/a	2
Other	13	12	9	n/a	7	24	13	13	2	n/a	11
No symptoms	1	0.6	0.6	n/a	0.7	11	0	12	2	n/a	3
Missing	8	11	17	n/a	18	18	21	24	20	n/a	17
Cancer-specificity of presenting symptom[3]											
Cancer-specific symptom	88	85	79	n/a	78	69	70	59	78	n/a	77
Non-specific symptom	3	3	4	n/a	3	3	9	4	0	n/a	4
No symptoms / Missing	9	12	18	n/a	18	28	21	37	22	n/a	20

[1] *These figures represent only women who had a symptom-based cancer diagnosis. More than one symptom could be stated (sum over 100%)*

[2] *Sweden and Northern Ireland did not include PCP questionnaire data*

[3] *Based on the PCP reported symptom/sign and categorised by two researchers*

For peer review only

Table 4. *Time intervals (days) for each jurisdiction depicted as median, 75th and 90th percentiles.*

		Wales	England	Scotland	N Ireland	Denmark	Ontario	Norway	Manitoba	Victoria	Sweden[1]
Patient Interval (Symptomatic)	Number	111	201	198	219	213	170	206	172	189	101
	Median	11	7	7	8	3	19	12	14	7	29
	75th percentile	34	30	30	31	22	58	48	47	31	56
	90th percentile	73	92	88	114	63	142	157	86	117	90
Primary Care interval (Symptomatic)	Number	97	167	160	172	141	70	99	109	142	n/a
	Median	0	0	0	0	0	20	0	17	7	
	75th percentile	0	0	1	0	0	37	0	30	15	
	90th percentile	3	7	6	3	10	75	14	82	38	
Diagnostic interval (Symptomatic)	Number	111	200	197	216	207	166	203	184	191	101
	Median	29	12	19	14	8	25	20	28	13	13
	75th percentile	54	18	35	21	26	56	37	42	21	24
	90th percentile	92	36	49	49	49	202	71	79	46	48
Diagnostic interval (Screen-detected)	Number	113	153	159	86	131	184	126	125	152	158
	Median	25	15	20	19	24	26	27	23	21	15
	75th percentile	45	21	29	26	36	42	44	43	32	25
	90th percentile	58	34	42	41	48	66	66	113	40	41
Diagnostic interval (All)	Number	224	353	356	302	338	350	329	309	343	259
	Median	29	13	20	15	14	26	22	26	16	14
	75th percentile	49	21	33	21	31	46	39	42	28	25
	90th percentile	70	36	48	43	49	90	68	94	45	41
Treatment interval (Symptomatic)	Number	115	203	202	241	230	201	216	198	196	113
	Median	24	29	24	21	20	35	19	38	14	22
	75th percentile	33	41	37	29	29	49	27	55	24	29
	90th percentile	45	55	54	40	44	65	34	72	33	42
Treatment interval (Screen-detected)	Number	115	155	158	86	134	195	120	155	170	165
	Median	27	31	34	24	19	35	13	39	19	21
	75th percentile	36	40	44	35	29	48	21	51	28	29
	90th percentile	49	62	69	44	36	65	28	66	38	40
Treatment interval (All)	Number	231	359	361	330	368	400	341	353	366	278
	Median	25	30	29	22	20	35	15	39	15	22
	75th percentile	35	41	41	31	29	48	24	54	27	29
	90th percentile	46	57	61	41	41	65	33	71	36	41
Total interval (Symptomatic)	Number	104	190	186	205	189	173	184	155	177	98
	Median	70	57	58	50	42	92	54	92	42	71
	75th percentile	96	82	99	78	73	158	121	128	89	101

For peer review only

For peer review only - http://bmjopen.bmj.com/site/about/guidelines.xhtml

	90th percentile	218	138	149	147	170	273	231	188	170	169
Total interval (All)	Number	217	343	341	291	320	354	298	277	327	253
	Median	60	52	55	46	44	78	48	76	42	42
	75th percentile	81	70	84	69	68	116	79	116	63	68
	90th percentile	123	114	129	127	118	209	168	182	120	101

[1] *In Sweden, data on the primary care interval were not collected.*

For peer review only

For peer review only - http://bmjopen.bmj.com/site/about/guidelines.xhtml

Table 5. *The differences in interval lengths (days) between Wales, the reference, and the other nine jurisdictions.*

		Wales	England	Scotland	N Ireland	Denmark	Ontario	Norway	Manitoba	Victoria	Sweden
**Patient Interval (Symptomatic)**	Number	111	201	198	219	213	170	206	172	189	101
	Median	11 (ref.)	-3(-9,3)	-3 (-8,3)	-1 (-8,6)	**-7** (-12,-2)	7 (-1,16)	1 (-7,9)	4 (-2,11)	-4 (-9,2)	**17** (11,22)
	75th percentile	34 (ref.)	-7 (-21,7)	-4 (-12,3)	-2 (-8,5)	**-10** (-16,-4)	**19** (5,33)	**16** (7,25)	12 (-1,25)	-6 (-16,4)	**22** (8,36)
	90th percentile	73 (ref.)	21 (-19,61)	11 (-45,66)	30 (-13,73)	-14 (-64,35)	50 (-9,110)	**74** (31,117)	9 (-35,54)	33 (-13,80)	14 (-31,60)
**Diagnostic interval (Symptomatic)**	Number	111	200	197	216	207	166	203	184	191	101
	Median	29 (ref.)	**-18** (-22,-15)	**-10** (-14,-6)	**-16** (-19,-13)	**-22** (-25,-18)	**-6** (-9,-2)	**-10** (-15,-5)	-1 (-5,4)	**-18** (-22,-14)	**-17** (-23,-12)
	75th percentile	54 (ref.)	**-39** (-45,-33)	**-24** (-29,-19)	**-37** (-42,-33)	**-34** (-40,-28)	-3 (-13,6)	**-21** (-36,-6)	**-16** (-23,-8)	**-34** (-52,-15)	**-33** (-39,-27)

For peer review only - http://bmjopen.bmj.com/site/about/guidelines.xhtml

For peer review only

	90th percentile	92 (ref.)	**-68** (-88,-48)	**-58** (-91,-25)	**-62** (-80,-45)	**-55** (-75,-35)	**73** (35,112)	**-35** (-62,-8)	**-30** (-46,-15)	**-63** (-78,-47)	**-58** (-81,-35)
**Diagnostic interval (Screen-detected)**	Number	113	153	159	86	131	184	126	125	152	158
	Median	25 (ref.)	-11 (-22,1)	-7 (-18,5)	-7 (-19,4)	-1 (-17,15)	0 (-12,12)	1 (-11,13)	-2 (-15,10)	-5 (-17,6)	-12 (-24,0)
	75th percentile	45 (ref.)	**-24** (-36,-13)	**-17** (-28,-6)	**-20** (-31,-8)	-9 (-21,2)	-3 (-15,8)	-1 (-15,13)	-4 (-15,7)	**-14** (-25,-3)	**-21** (-34,-7)
	90th percentile	58 (ref.)	**-23** (-30,-16)	**-16** (-22,-11)	**-19** (-26,-12)	**-10** (-14,-6)	9 (-6,24)	8 (-3,20)	**55** (38,71)	**-17** (-26,-8)	**-17** (-20,-13)
**Diagnostic interval (All)**	Number	224	353	356	302	338	350	329	309	343	259
	Median	29 (ref.)	**-14** (-16,-12)	**-8** (-11,-6)	**-13** (-15,-11)	**-14** (-16,-11)	-2 (-5,1)	**-6** (-9,-2)	-1 (-5,2)	**-12** (-15,-10)	**-14** (-17,-11)
	75th percentile	49 (ref.)	**-30** (-34,-25)	**-18** (-23,-14)	**-29** (-33,-25)	**-20** (-25,-16)	-5 (-15,4)	**-11** (-20,-2)	**-8** (-12,-3)	**-22** (-26,-19)	**-25** (-32,-17)
	90th percentile	70 (ref.)	**-35** (-39,-31)	**-23** (-30,-16)	**-26** (-34,-19)	**-21** (-40,-2)	**20** (9,31)	-1 (-16,14)	**22** (7,38)	**-27** (-40,-13)	**-29** (-36,-21)
**Treatment interval (Symptomatic)**	Number	115	203	202	241	230	201	216	198	196	113
	Median	24 (ref.)	**6** (1,11)	1 (-3,5)	-3 (-7,1)	-3 (-7,1)	**12** (8,16)	-5 (-11,1)	**15** (11,19)	**-10** (-14,-6)	-2 (-6,2)
	75th percentile	33 (ref.)	**8** (4,12)	4 (-1,9)	-3 (-8,1)	-4 (-9,0)	**16** (11,21)	**-6** (-9,-2)	**22** (18,26)	**-9** (-16,-2)	-4 (-13,5)
	90th percentile	45 (ref.)	10 (-1,20)	11 (-4,26)	-4 (-19,11)	-1 (-14,12)	**20** (10,31)	-11 (-23,2)	**28** (10,46)	-11 (-26,4)	-3 (-15,10)
**Treatment interval (Screen-detected)**	Number	115	155	158	86	134	195	120	155	170	165
	Median	27 (ref.)	**5** (1,8)	**8** (4,11)	0 (-5,5)	**-6** (-10,-1)	**9** (7,12)	**-12** (-15,-10)	**12** (9,16)	-5 (-12,1)	**-3** (-6,-1)
	75th percentile	36 (ref.)	4 (-2,10)	10 (-3,22)	0 (-6,7)	-7 (-16,3)	**11** (3,20)	**-15** (-27,-4)	**15** (7,23)	-7 (-16,2)	-6 (-17,5)
	90th percentile	49 (ref.)	5 (-8,18)	**15** (4,25)	-9 (-20,3)	**-14** (-26,-2)	9 (-4,22)	**-24** (-35,-13)	12 (-1,24)	**-14** (-27,-1)	-11 (-21,0)
**Treatment interval (All)**	Number	231	359	361	330	368	400	341	353	366	278
	Median	25 (ref.)	5 (0,11)	4 (0,8)	-4 (-8,1)	**-5** (-9,-1)	**10** (6,14)	**-10** (-14,-6)	**14** (9,18)	**-10** (-14,-6)	-3 (-7,0)
	75th percentile	35 (ref.)	**6** (2,10)	**6** (1,10)	-4 (-8,0)	**-6** (-10,-2)	**13** (10,17)	**-11** (-16,-6)	**20** (16,23)	**-9** (-13,-5)	**-6** (-10,-2)
	90th percentile	46 (ref.)	**8** (2,14)	12 (-1,26)	**-6** (-10,-2)	**-6** (-9,-2)	**17** (11,23)	**-14** (-17,-12)	**23** (16,29)	**-11** (-14,-9)	**-7** (-10,-4)
**Total interval (Symptomatic)**	Number	104	190	186	205	189	173	184	155	177	98
	Median	70 (ref.)	**-14** (-24,-3)	**-12** (-23,-2)	**-20** (-31,-8)	**-25** (-38,-11)	**28** (15,42)	-15 (-34,5)	**22** (12,31)	**-28** (39,-17)	-1 (-11,10)
	75th percentile	96 (ref.)	**-16** (-27,-5)	-5 (-18,9)	**-18** (-33,-3)	**-25** (-39,-12)	**69** (31,107)	**27** (8,46)	**30** (8,53)	-13 (-27,0)	3 (-30,35)
	90th percentile	218 (ref.)	**-71** (-102,-41)	**-60** (-85,-34)	**-57** (-66,-48)	**-41** (-66,-16)	**79** (66,93)	8 (-15,30)	**-17** (-34,-1)	-21 (-67,26)	**-39** (-67,-10)
**Total interval (All)**	Number	217	343	337	291	320	354	298	277	327	253
	Median	60 (ref.)	**-9** (-14,-3)	-4 (-11,2)	**-13** (-18,-8)	**-15** (-26,-4)	**16** (6,26)	**-13** (-21,-6)	**17** (9,24)	**-18** (-24,-12)	**-17** (-25,-10)
	75th percentile	81 (ref.)	**-12** (-20,-4)	1 (-9,10)	**-13** (-20,-6)	**-14** (-20,-9)	**32** (24,41)	-4 (-12,4)	**35** (21,48)	**-19** (-25,-12)	**-17** (-26,-7)
	90th percentile	123 (ref.)	-13 (-34,9)	3 (-12,18)	-5 (-22,11)	-8 (-38,22)	**73** (33,114)	**32** (2,62)	**47** (13,82)	-12 (-37,13)	-24 (-51,4)

For peer review only - http://bmjopen.bmj.com/site/about/guidelines.xhtml

The differences for the median, 75th and 90th percentiles were calculated as marginal effects after quantile regression for count data. The continuous covariate age was set to its mean value and categorical covariate comorbidity was set to the mode. Significant results are shown in bold. The actual number of days are included for Wales are shown in Table 5. Note that analyses of primary care intervals are presented in Table 6.

Table 6. *The prevalence ratios[1] (PRs) of experiencing differing primary care intervals for symptomatic breast cancer patients.*

	Wales (N=97)	England (N=167)	Scotland (N=160)	N Ireland (N=172)	Denmark (N=141)	Ontario (N=70)	Norway (N=99)	Manitoba (N=109)	Victoria (N=142)	Sweden[2] (N=0)
Interval ≥ 5 days										
PR (95% CI)	ref.	1.7 (0.7-4.2)	2.0 (0.8-4.9)	1.3 (0.5-3.3)	2.0 (0.8-4.8)	**12.9** (5.9-28.2)	2.2 (0.9-5.6)	**12.9** (5.9-28.1)	**11.1** (5.1-24.4)	n/a
Interval ≥ 14 days										
PR (95% CI)	ref.	1.6 (0.5-4.9)	1.5 (0.5-4.7)	1.4 (0.5-4.3)	2.1 (0.7-6.3)	**15.1** (5.7-40.1)	2.4 (0.8-7.4)	**14.3** (5.4-37.8)	**6.7** (2.5-18.1)	n/a

Bold: significance level of $p \leq 0.05$; n/a=not applicable

[1] Adjusted for differences in age and comorbidity among the patients, with Wales as reference.

[2] In Sweden, no information on primary care interval was collected.

For peer review only

For peer review only - http://bmjopen.bmj.com/site/about/guidelines.xhtml

Figure One – Diagnostic and treatment intervals[23]

For peer review only

For peer review only - http://bmjopen.bmj.com/site/about/guidelines.xhtml

Figure Two – Time intervals for each jurisdiction compared with Wales (reference)*

**Adjusted for differences in comorbidity and age. Differences in interval lengths (in days) are shown for the median, 75th and 90th percentiles compared to the reference used for the regression analyses, Wales. Wales is represented by the axis, with jurisdictions with shorter intervals shown to the left of the axis, and jurisdictions with longer intervals shown to the right of the axis for each graph. Solid-fill bars indicate statistically significant differences compared with Wales. Primary care interval not shown, see Table 6.*

For peer review only

For peer review only - http://bmjopen.bmj.com/site/about/guidelines.xhtml

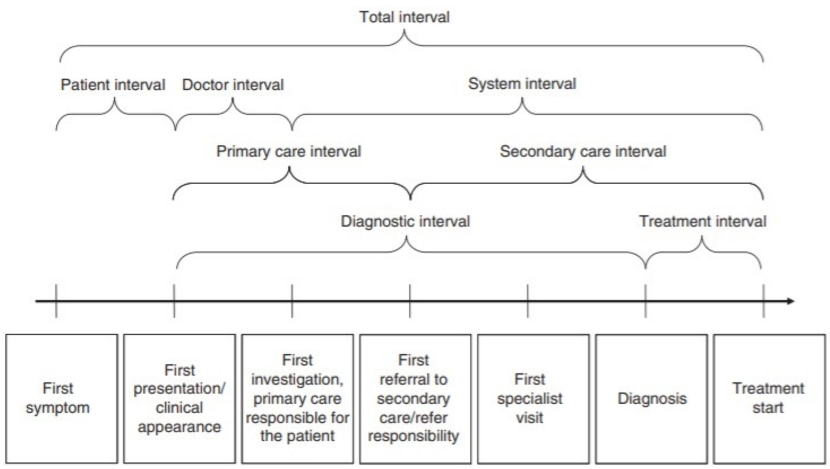

Diagnostic and treatment intervals

155x74mm (144 x 144 DPI)

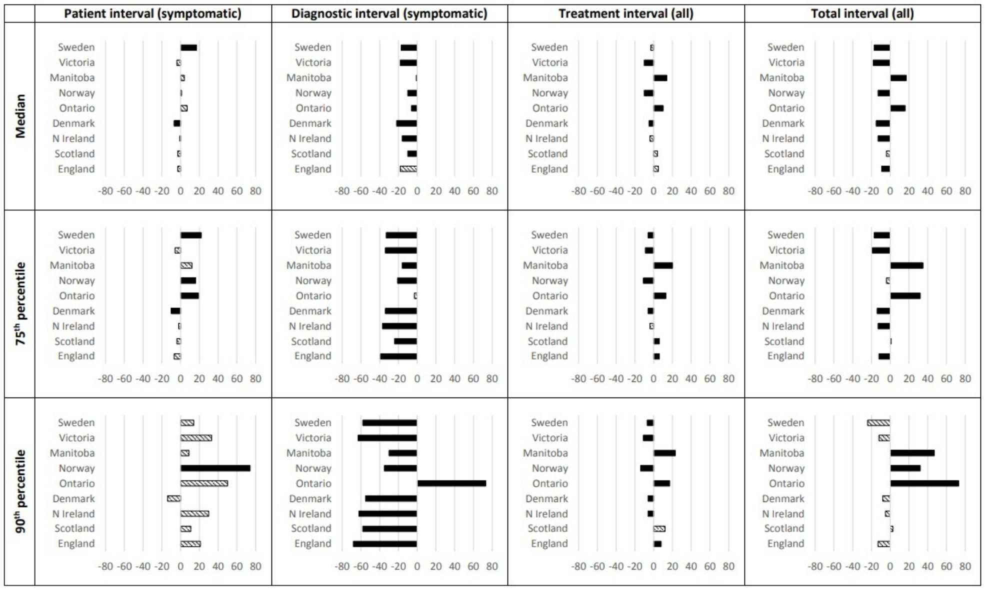

Time intervals for each jurisdiction compared with Wales (chosen as the reference jurisdiction as it had the lowest ovarian cancer survival in the ICBP Module 1 cancer survival analysis). Adjusted for differences in comorbidity and age. Differences in interval lengths (in days) are shown for the median, 75th and 90th percentiles compared to the reference used for the regression analyses, Wales. Wales is represented by the axis, with jurisdictions with shorter intervals shown to the left of the axis, and jurisdictions with longer intervals shown to the right of the axis for each graph. Solid-fill bars indicate statistically significant differences compared with Wales. Primary care interval not shown, see Table 6.

208x126mm (144 x 144 DPI)

Diagnostic pathways for breast cancer in ten jurisdictions: Findings from the International Cancer Benchmarking Partnership (ICBP) _ Supplementary Materials

BOX 1 – *Short introduction to quantile regression for count data*

The quantile regression (QR) estimates the relationship between the qth quantile $Qq(y/x)$ of the response y and explanatory variable x and was originally developed for continuous responses [26]. Machado and Santos Silva [27] proposed QR for count data based on adding uniform noise u ~ U(0,1) to count response y (i.e. jittering count response), giving a continuous random variable z = y + u. The form of the QR model for these data is $Qq(z/x)=q+exp(x\beta q)$, where βq is the estimated coefficient. It is possible to recover $Qq(y/x)$ based on $Qq(z/x)$ using the postestimation transformation of the form $Qq(y/x)=[Qq(z/x)-1]$, where [.] represents the ceiling function. In Stata, the QR for counts can be performed by using the 'qcount' command [28]. The parameters of the model estimated use an exponential functional form for the conditional quantile. To interpret results, it is easier to use the marginal effects (MEs). The subsequent command 'qcount_mfx' gives the associated MEs.

For peer review only

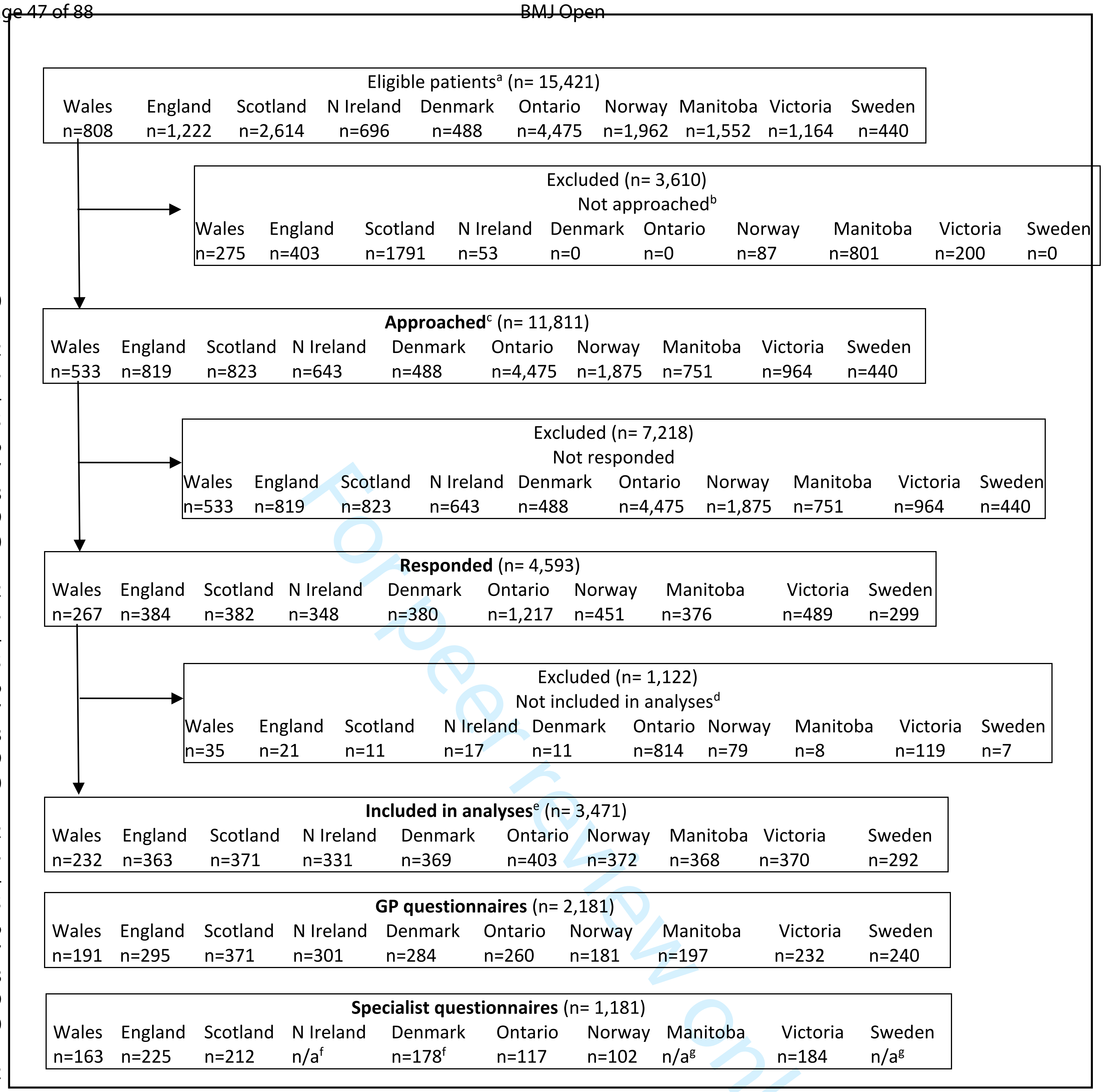

Fig.1. Flowchart of study population. [a]Eligible according to protocol: i.e. woman, 40 years or more, alive, consented to participate within nine months of diagnosis, diagnosed with breast cancer (ICD-10: C50.0-C50.9), behaviour code ICD-O-3=3 and without prior history of cancer of the breast or synchronous primary breast cancer. [b]Excluded due to: pack not forwarded by GP; unsure if pack forwarded by GP; patient died; no address; other reasons. [c]Patients In Wales, England and Scotland were approached via GP. Patients in other jurisdictions were approached directly by registries/research teams. [d]Excluded due to: extra sample for local purpose; received after submission; previous cancer; unknown date of consent or diagnosis; consent too late/early; other reasons. [e]Number of patient questionnaires. [f]Data obtained from registries instead: N.Ireland Cancer Registry in N.Ireland; the Danish Breast Cancer Group in Denmark. [g]Data not collected for this jurisdiction

For peer review only

For peer review only

ICBP
International Cancer
Benchmarking Partnership

International Cancer Benchmarking Partnership Module 4

Patient questionnaire

Breast Cancer

Thank you very much for taking the time to fill in this questionnaire – it should take about 20 minutes to complete. We are sending the questionnaire to a large sample of people who we understand have had a diagnosis of breast cancer. If this has been sent to you in error and you do not have cancer, please do not continue and return the documents in the prepaid envelope.

Our aim is to gain a better understanding of the process by which people have their cancer diagnosed. We would also like to find out more about the symptoms they experience (if any), and the pathway they follow from start of symptoms to treatment of their cancer. This will help in identifying ways in which cancers can be diagnosed quickly and effectively. Thank you once again for your time.

This information is confidential and will not be passed to anyone involved in your treatment.

Name:

Date of Birth:

Address:

Copyright property of the International Benchmarking Partnership (ICBP) and Module 4 collaborators. Usage must be authorised

For peer review only - http://bmjopen.bmj.com/site/about/guidelines.xhtml

Sample

ICBP
International Cancer
Benchmarking Partnership

Consent form

Please read the consent form and sign your name and date **BELOW**.

If you require any clarification, please do not hesitate to ring the study team members. Their contact details are found on the information sheet.

Please be reassured that your responses are completely confidential and will not be passed to anyone involved in your treatment. For the purposes of the study it is important that you agree to consent to all the statements listed below.

- I confirm that I have read the attached information sheet and I understand why the research is being done.
- I am willing for the team to request information from my GP and hospital doctors which is relevant to the audit as described in the information sheet.
- I give permission for my details (name, address) to be given to the cancer registry (NHS Information Centre for Health and Social Care) for follow up.
- I agree for the information I have provided and any other relevant information from my medical records to be stored as described in the information sheet under the custodianship of University College London.
- I consent to sharing of coded data which contains no personal identifiers between researchers, some of whom are located outside the European Union.
- I consent for use of my data if I become mentally incapacitated during the course of the project.

I agree to all the statements listed and consent to participate in the study.

Name (Please print)

Signature: Date:

If we have any questions, may we phone you for clarification? (Please tick) ☐ **Yes** ☐ **No**

If **Yes**, please provide your telephone number:

Copyright property of the International Benchmarking Partnership (ICBP) and Module 4 collaborators. Usage must be authorised

Sample
For peer review only
For peer review only - http://bmjopen.bmj.com/site/about/guidelines.xhtml

1. **Please can you confirm the details of your GP/GP practice (name, practice address – as best as you can remember): We appreciate that you may have more than one GP involved in your care – in which case, we are interested in the GP you would say provides the majority of your care, particularly relating to the cancer you've had diagnosed.**

Name of doctor

Name of practice

Address

Postcode

Town

Sample

Copyright property of the International Benchmarking Partnership (ICBP) and Module 4 collaborators. Usage must be authorised

For peer review only - http://bmjopen.bmj.com/site/about/guidelines.xhtml

ICBP International Cancer Benchmarking Partnership

2. Which of the following best describes the events which led to your diagnosis of cancer? (please tick only **ONE** answer)

	✓
I had symptoms/I noticed a bodily change and went to see a doctor (e.g. GP)	
I had symptoms/I noticed a bodily change and went/was taken to Accident and Emergency (A&E)	
I had seen a doctor/GP with symptoms, but went/was taken to Accident and Emergency (A&E) when things worsened	
I was being investigated by my doctor(s) for another problem during which time the cancer was discovered	
I had a cancer screening test (mammogram) as part of a breast screening programme (e.g. the NHS Breast Screening Programme in England – NHSBSP)	
Other (please describe):	

What date did you have this screening test? If you cannot remember the exact date, you can fill in the month and the year.

Day (optional), month, year

D	D	M	M	Y	Y	Y	Y

Sample

Copyright property of the International Benchmarking Partnership (ICBP) and Module 4 collaborators. Usage must be authorised

For peer review only - http://bmjopen.bmj.com/site/about/guidelines.xhtml

3. The following health concerns or symptoms are commonly experienced with breast cancer.

Changes in the appearance of the breast, eg:<br>■ the nipple<br>■ the skin of the breast<br>■ the shape of the breast<br>■ the size of the breast
Nipple discharge including bleeding
Lump, swelling or thickening in breast or armpit
Fatigue
Unexplained weight loss
Loss of appetite

Sample

Please write down ALL health concern(s) or symptom(s) you may have had before contacting a doctor or taking part in screening. It does not matter if they are not included in the list above:

Please write your health concern(s) or symptom(s) in the boxes below:
1)
2)
3)
4)
5)
6)

This is not applicable to me (e.g. I did not have any symptoms), please tick	

Copyright property of the International Benchmarking Partnership (ICBP) and Module 4 collaborators.
Usage must be authorised

For peer review only - http://bmjopen.bmj.com/site/about/guidelines.xhtml

ICBP
International Cancer
Benchmarking Partnership

4. Please write down your best estimate of the date you noticed the first of these health concern(s) or symptom(s). If you cannot remember the exact date, you can fill in the month and the year.

Day (optional), month, year

D	D	M	M	Y	Y	Y	Y

This is not applicable to me (e.g. I had no symptoms), please tick	✓

5. Approximately how long did you have health concern(s) or symptom(s) before contacting a doctor? (Please think of the first visit to the doctor, not re-visits after that). Please tick only **ONE** answer.

	✓
Less than 1 week	
1-2 weeks	
3-4 weeks	
5-7 weeks	
2-5 months	
6-12 months	
More than 12 months	

This is not applicable to me (e.g. I had no symptoms), please tick	✓

Sample
For peer review only

Copyright property of the International Benchmarking Partnership (ICBP) and Module 4 collaborators.
Usage must be authorised
For peer review only - http://bmjopen.bmj.com/site/about/guidelines.xhtml

6a. Once you contacted a practice about your health concern(s) or symptom(s), how long did it take to get an appointment with a doctor? (Please think of the first visit to the doctor, to discuss your health concern(s) or symptom(s)). Please tick only **ONE** answer.

	✓
Same day/next day	
Within 1 week	
1-2 weeks	
3-4 weeks	
Longer	

If there was no waiting time (e.g. you went/were taken to A&E), please tick this box	

This is not applicable to me (e.g. I had no symptoms), please tick	

6b. What was the date you first saw your doctor about your health concern(s) or symptom(s)? If you cannot remember the exact date, you can fill in the month and the year.

Day (optional), month, year

D	D	M	M	Y	Y	Y	Y

	✓
This is not applicable to me (e.g. I had no symptoms), please tick	

Sample

Copyright property of the International Benchmarking Partnership (ICBP) and Module 4 collaborators. Usage must be authorised

For peer review only - http://bmjopen.bmj.com/site/about/guidelines.xhtml

ICBP International Cancer Benchmarking Partnership

7. How many times did you visit the following for the investigation of your symptoms before your cancer was diagnosed?

	Please write down the number of visits
GP	
Hospital	
Consultant/specialist outside of a hospital	

	✓
This is not applicable to me (e.g. I had no symptoms)	

8a. After your doctor referred you to a specialist, how long did it take you to get an appointment? Please tick only **ONE** answer.

	✓
Less than 1 week	
1-2 weeks	
3-4 weeks	
5-7 weeks	
2-5 months	
6-12 months	
More than 12 months	

	✓
This is not applicable to me (eg my doctor did not refer me), please tick	

Copyright property of the International Benchmarking Partnership (ICBP) and Module 4 collaborators. Usage must be authorised
For peer review only - http://bmjopen.bmj.com/site/about/guidelines.xhtml

8b. What was the date of your first appointment with a doctor, involved in investigating and/or treating your cancer, to whom you were referred?
If you cannot remember the exact date, you can fill in the month and the year.

Day (optional), month, year

D	D	M	M	Y	Y	Y	Y

This is not applicable to me (e.g. my doctor did not refer me), please tick	✓

9. What was the date you were told you had cancer? If you cannot remember the exact date, you can fill in the month and the year.

Day (optional), month, year

D	D	M	M	Y	Y	Y	Y

Sample

For peer review only

Copyright property of the International Benchmarking Partnership (ICBP) and Module 4 collaborators. Usage must be authorised

ICBP International Cancer Benchmarking Partnership

10. Have you had any of the following treatments for your cancer yet? If so, please can you estimate the date this treatment started? Please tick **ALL** that apply. If you cannot remember the exact date, you can fill in the month and the year.

	Type of treatment		Date of treatment (give first date if you had more than one)
a.	Surgery	☐ Yes ☐ No	Day (optional), month, year<br>D D M M Y Y Y Y
b.	Chemotherapy	☐ Yes ☐ No	Day (optional), month, year<br>D D M M Y Y Y Y
c.	Radiotherapy	☐ Yes ☐ No	Day (optional), month, year<br>D D M M Y Y Y Y
d.	Other<br>Please specify:	☐ Yes ☐ No	Day (optional), month, year<br>D D M M Y Y Y Y
e.	Treatment not started yet	☐ Yes	

Sample

Copyright property of the International Benchmarking Partnership (ICBP) and Module 4 collaborators. Usage must be authorised

For peer review only - http://bmjopen.bmj.com/site/about/guidelines.xhtml

11. Who is the consultant doctor who has taken responsibility for diagnosing and or/treating your cancer?

Name of consultant:

Hospital name:

Hospital department:

Please can you answer some more general questions about your health?
It will help us in interpreting your responses to this questionnaire to know about your general health and other health problems you may have had in the past.

12. Looking back to the 2 years before you were diagnosed with cancer, would you say your general health was (Please tick only **ONE** answer.)**:** ✓

Very good	
Good	
Fair	
Poor	
Very poor	

13. Have you been treated before for any of the conditions below?
Please tick 'yes' or 'no' for each condition:

Heart disease	☐ **Yes**	☐ **No**
Stroke	☐ **Yes**	☐ **No**
Lung disease	☐ **Yes**	☐ **No**
Diabetes	☐ **Yes**	☐ **No**

Copyright property of the International Benchmarking Partnership (ICBP) and Module 4 collaborators.
Usage must be authorised

Sample

For peer review only

ICBP International Cancer Benchmarking Partnership

Finally, a little more information about you. The information you provide below will help us to analyse the results of the survey in more detail.

14. Which of these best describes your ethnic group? (please tick one box, as appropriate). If you are descended from more than one ethnic or racial group, please tick the group you consider you belong to, or tick 'any other ethnic group'.

	✓		✓		✓		✓
White		Chinese		Black - Caribbean		Black - African	
Black - other		Indian		Pakistani		Bangladeshi	
Any other ethnic group, please specify:							

15. What is the main language spoken in your home? Please tick

	✓
English	
Other, please specify:	

16. What is the highest level of education you have achieved? Please tick only **ONE** answer.

	✓
Finished school at or before the age of fifteen	
Completed GCSEs, O-levels or equivalent	
Completed A Levels or equivalent	
Completed further education but not a degree	
Completed a Bachelor's degree / Masters degree / PhD	
Other, please specify:	

Sample

Copyright property of the International Benchmarking Partnership (ICBP) and Module 4 collaborators. Usage must be authorised

For peer review only - http://bmjopen.bmj.com/site/about/guidelines.xhtml

17. **Have you ever smoked cigarettes, including hand-rolled ones, pipes or cigars?**

☐ Yes ☐ No

18. **Are you a current smoker, smoking either cigarettes, including hand-rolled ones, pipes or cigars?**

☐ Yes ☐ No

19. **If you are a current smoker or have smoked in the past, how many cigarettes, including hand-rolled ones, pipes or cigars on average do you smoke/have you smoked per day?**

Number per day:	

Sample

For peer review only

Copyright property of the International Benchmarking Partnership (ICBP) and Module 4 collaborators.
Usage must be authorised

For peer review only - http://bmjopen.bmj.com/site/about/guidelines.xhtml

ICBP
International Cancer Benchmarking Partnership

20. Further comments

Please add anything else that you would like to tell us about your cancer diagnosis or treatment.

Sample

For peer review only

Thank you very much for taking the time to complete this questionnaire.

Copyright property of the International Benchmarking Partnership (ICBP) and Module 4 collaborators.
Usage must be authorised

ICBP
International Cancer
Benchmarking Partnership

International Cancer Benchmarking Partnership Module 4

Primary Care Audit
Breast Cancer

Thank you very much for agreeing to fill in this questionnaire. As part of an international study examining differences in cancer survival, we are sending the questionnaire to health care providers of a sample of consented patients with cancer.

Our aim is to gain a better understanding of the process by which people have their cancer diagnosed – the symptoms they experience, and the pathway they follow from onset of symptoms to treatment of their cancer. This will help in identifying ways in which cancers can be diagnosed and treated quickly and effectively. Thank you once again for your time.

Please can you refer to your patient's notes in completing the questionnaire as this will help in obtaining accurate data on time points.

If you would prefer to return this questionnaire without the patient details, please tear off along the dotted line

ID-number: Jurisdiction-ID + Patient-ID:

Copyright property of the International Benchmarking Partnership (ICBP) and Module 4 collaborators. Usage must be authorised

Sample

ICBP
International Cancer Benchmarking Partnership

Sample

For peer review only

Patient information

ID-number: Jurisdiction-ID + Patient-ID:

Full name:

Address:

Postcode:

Date of birth: D D M M Y Y Y Y

Copyright property of the International Benchmarking Partnership (ICBP) and Module 4 collaborators. Usage must be authorised

For peer review only - http://bmjopen.bmj.com/site/about/guidelines.xhtml

1. Duration of symptoms

Please estimate how long your patient had symptom(s), attributable to breast cancer, before attending your practice (or other health service).
We appreciate that identifying a 'date of first symptom' is not always straightforward – particularly when there are multiple and/or chronic symptoms. Nevertheless, we hope you can provide a 'best estimate'.

**Estimate of symptom duration** (please tick **one**):	✓	**What were the symptoms?** Please describe:
Less than 1 week		
1 to 4 weeks		
5 to 7 weeks		
2-5 months		
6-12 months		
More than 12 months		
Not possible to estimate		
No symptoms (e.g. screen detected cancers)		

Sample

Copyright property of the International Benchmarking Partnership (ICBP) and Module 4 collaborators. Usage must be authorised

For peer review only

For peer review only - http://bmjopen.bmj.com/site/about/guidelines.xhtml

ICBP International Cancer Benchmarking Partnership

2. Pathway of presentation

2.1 Through what route did the patient first present? Please tick **ONE**.

	✓	
Your patient first presented to primary care (either in-hours or out-of-hours)		Please can you provide your best approximation of the date of this primary care visit<br>D D M M Y Y Y Y
Your patient presented straight to A&E (with or without your involvement)		
Your patient first presented to primary care, but then at a later date presented to A&E as an emergency (with or without your involvement)		Please can you provide your best approximation of the date of this primary care visit<br>D D M M Y Y Y Y
Your patient's breast cancer was diagnosed through an organised screening programme (e.g. not as a result of investigation of symptoms)		
Other – please describe:		

Sample

Copyright property of the International Benchmarking Partnership (ICBP) and Module 4 collaborators. Usage must be authorised

For peer review only - http://bmjopen.bmj.com/site/about/guidelines.xhtml

3. Date you ordered any tests/investigations in response to symptom(s).

We are interested in any kind of tests/investigations (e.g. imaging etc) that you may have ordered. Please only consider the tests/investigations that you ordered yourself. Please tick **ALL** that apply and put in the date that the test/investigation was ordered:

	✓	
Mammogram		D D M M Y Y Y Y
Breast ultrasound		D D M M Y Y Y Y
Breast biopsy		D D M M Y Y Y Y
Other (please specify):		D D M M Y Y Y Y

4. Date of referral to specialist medical services

At what date did you **first** refer the patient to hospital or another specialist transferring the responsibility for on-going investigation/treatment to other medical services?

D	D	M	M	Y	Y	Y	Y

Sample

For peer review only

Copyright property of the International Benchmarking Partnership (ICBP) and Module 4 collaborators.
Usage must be authorised

ICBP
International Cancer Benchmarking Partnership

5. Nature of this referral

5.1 Do you know the date that the patient was seen for this referral?

- ☐ **Yes**, please provide the date: D D M M Y Y Y Y
- ☐ **No**

5.2 If you did make a referral to specialist services, which of the following best describes the nature/characteristics of this referral? Please tick **one**.

	✓
Emergency admission: a referral to A&E (or equivalent) for immediate admission	
An urgent referral for assessment of cancer symptoms/signs/test results (Note this will be within 2 weeks for England/Wales)	
A less urgent referral in which cancer is raised as a possibility (Note this will be greater than 2 weeks for England/Wales)	
A more general referral for investigation and assessment without cancer mentioned	
No referral was made	
Other – please describe:	

5.3 Would you say this patient's diagnostic pathway was conducted predominantly in the public or private system? Please tick **one**.

	✓
Public healthcare system	
Private healthcare system	

Sample

For peer review only

Copyright property of the International Benchmarking Partnership (ICBP) and Module 4 collaborators.
Usage must be authorised

6. Date of breast cancer diagnosis

This can be decided in different ways. Please provide whichever of the following dates you have to hand. Please tick **all** that apply.

	✓	
Date of histological confirmation [ideal]		D D M M Y Y Y Y
Date results of investigation (histological or other) confirming cancer received		D D M M Y Y Y Y
Date patient was told		D D M M Y Y Y Y
Date biopsy undertaken		D D M M Y Y Y Y
Date patient was first admitted to hospital because of the malignancy		D D M M Y Y Y Y
Other (please specify):		D D M M Y Y Y Y

Sample
For peer review only

Copyright property of the International Benchmarking Partnership (ICBP) and Module 4 collaborators. Usage must be authorised

For peer review only - http://bmjopen.bmj.com/site/about/guidelines.xhtml

ICBP
International Cancer Benchmarking Partnership

7. Additional information

Finally, we are interested to know what other conditions your patient has, and the severity/impact of these conditions

Have you and/or any of your partners treated this patient (or has the patient been to hospital) for any of the following conditions? Please tick **ALL** that apply:

Cardiovascular disease	☐ **Yes** ☐ **No**
Stroke	☐ **Yes** ☐ **No**
Lung disease	☐ **Yes** ☐ **No**
Diabetes	☐ **Yes** ☐ **No**

Are there any other comments you would like to make about this patient?

Name (and title):

Signature:

Date:

Thank you very much for taking the time to complete this questionnaire.

Copyright property of the International Benchmarking Partnership (ICBP) and Module 4 collaborators. Usage must be authorised

For peer review only - http://bmjopen.bmj.com/site/about/guidelines.xhtml

Sample

ICBP International Cancer Benchmarking Partnership

International Cancer Benchmarking Partnership Module 4

Specialist Care Audit
Breast Cancer

Thank you very much for agreeing to fill in this questionnaire – it should take about 10 minutes to complete. As part of an international study examining differences in cancer survival, we are sending the questionnaire to health care providers of a sample of patients with cancer.

Our aim is to gain a better understanding of the process by which people have their cancer diagnosed – the symptoms they experience, and the pathway they follow from onset of symptoms to treatment of their cancer. We hope you can help us with information on this patient's cancer journey once they were referred to specialist cancer services. This will help in identifying ways in which cancers can be diagnosed and treated quickly and effectively.

Thank you once again for your time

Please can you refer to your patient's notes in completing the questionnaire, as this will help in obtaining accurate data on time points.

If you would prefer to return this questionnaire without the patient details, please tear off along the dotted line.

Your patient ______________________________

is participating in the study.

Copyright property of the International Benchmarking Partnership (ICBP) and Module 4 collaborators. Usage must be authorised

For peer review only - http://bmjopen.bmj.com/site/about/guidelines.xhtml

Sample

ICBP
International Cancer Benchmarking Partnership

Sample

For peer review only

Patient information

ID-number: Jurisdiction-ID + Patient-ID:

Full name:

Address:

Postcode:

Date of birth: | D | D | M | M | Y | Y | Y | Y |

Copyright property of the International Benchmarking Partnership (ICBP) and Module 4 collaborators.
Usage must be authorised

For peer review only - http://bmjopen.bmj.com/site/about/guidelines.xhtml

1. Date patient first attended hospital/specialist services related to their cancer diagnosis. We appreciate this date can at times be difficult to identify, particularly when there have been multiple visits in the lead up to a definitive diagnosis. Put another way, it's the date that the hospital/specialist service assumed responsibility for on-going investigation/treatment for your patient.

Day (optional), month, year

D	D	M	M	Y	Y	Y	Y

2. How was the patient referred to the hospital/specialist services related to their cancer diagnosis? Please tick.

Was it through a:

	✓		✓
GP referral		Screening	
Referral from general surgery clinic		Medical specialist/ Consultant referral	
Other referral – please specify:			

3. Where did this first contact/appointment happen? Please tick.
Which of the following best describes where this first contact/appointment took place?

	✓		✓
Emergency department ('A&E')		Medical outpatient department, please specify which department	
Oncology general outpatient department		Surgical outpatient department, please specify which department	
Other – please specify:			

Sample
For peer review only
Copyright property of the International Benchmarking Partnership (ICBP) and Module 4 collaborators. Usage must be authorised
For peer review only - http://bmjopen.bmj.com/site/about/guidelines.xhtml

ICBP International Cancer Benchmarking Partnership

4. Date of diagnosis

This can be decided in different ways.
Please tick and complete as many of the following dates as possible.

	✓	
Date of histological confirmation (ideal)		Day (optional), month, year<br>D D M M Y Y Y Y
Date results of investigation confirming cancer received		Day (optional), month, year<br>D D M M Y Y Y Y
Date patient was told		Day (optional), month, year<br>D D M M Y Y Y Y
Date of biopsy		Day (optional), month, year<br>D D M M Y Y Y Y
Date patient was first admitted to hospital because of the malignancy		Day (optional), month, year<br>D D M M Y Y Y Y
Date of MDT confirmation of diagnosis		Day (optional), month, year<br>D D M M Y Y Y Y
Other (please specify):		Day (optional), month, year<br>D D M M Y Y Y Y

Sample

For peer review only

Copyright property of the International Benchmarking Partnership (ICBP) and Module 4 collaborators.
Usage must be authorised

For peer review only - http://bmjopen.bmj.com/site/about/guidelines.xhtml

5. Date treatment for the cancer commenced

Based on your records, when would you say that any treatment specifically targeting the patient's cancer started?

Day (optional), month, year

D	D	M	M	Y	Y	Y	Y

6. Additional information

Please can you provide any further information on the patient's cancer:

**TNM**, please tick as appropriate:	
0	
I	
IIA	
IIB	
IIC	
IIIA	
IIIB	
IIIC	
IV	
Not able to stage	

Sample

Copyright property of the International Benchmarking Partnership (ICBP) and Module 4 collaborators. Usage must be authorised

For peer review only - http://bmjopen.bmj.com/site/about/guidelines.xhtml

ICBP International Cancer Benchmarking Partnership

6.1 Histological subtype:

	✓
Invasive Ductal	
Invasive Lobular	
Invasive Tubular	
DCIS	
Other (please specify):	

Sample

For peer review only

Copyright property of the International Benchmarking Partnership (ICBP) and Module 4 collaborators.
Usage must be authorised
For peer review only - http://bmjopen.bmj.com/site/about/guidelines.xhtml

Further comments

Sample
For peer review only

Copyright property of the International Benchmarking Partnership (ICBP) and Module 4 collaborators.
Usage must be authorised

ICBP
International Cancer Benchmarking Partnership

Name (and title):

Signature:

Date:

Are you a ... (please tick below):

Surgeon	
Medical Oncologist	
Clinical Oncologist	
Clinical Nurse Specialist	
Other (please specify):	

Thank you very much for taking the time to complete this questionnaire.

Copyright property of the International Benchmarking Partnership (ICBP) and Module 4 collaborators.
Usage must be authorised
For peer review only - http://bmjopen.bmj.com/site/about/guidelines.xhtml
Sample

Additional File 4. *ICBP M4 Rules for missing, incomplete, multiple response and out of range data*

1. Oversampling
   To handle oversampling in Ontario, include only the first 430 consecutive breast cancer patients
2. Language/Participation in study/Presence of cancer
   Exclude patients who checked “No, I don’t understand the language” or “I don’t want to participate in this study” or “I don’t have cancer”.
3. Survey responders
   a) Exclude Patient/PCP/Specialist survey from the analysis, if it was not written by Patient/PCP/Specialist (example: a medical oncologist completed a PCP survey);
   b) In the case of duplicates, include only the first survey (example: 2 specialists completed surveys for the same patient).
4. Age
   a) Exclude patients with unknown age;
   b) Exclude patients younger 40 years;
   c) Use registry data, if Age is reported by both patient and registry.
5. No cancer or Previous cancer in the same organ
   a) Exclude patents with no cancer based on registry data;
   b) Exclude patients with previous cancer in the same organ based on data from registry or free-text for Presentation in the patient survey.
6. Date of consent
   Exclude patients with date of consent, which is unknown, before 01.01.2013 or in the future.
7. Multiple responses to Dates
   If multiple responses were given to the dates (of first symptom; screening; first presentation to primary care; referral; diagnosis; treatment start), then use the earliest date.
8. Order of Dates
   The dates must be in the following order –
   a) First symptom; first presentation to Primary Care; referral; diagnosis; treatment start.
   b) Screening; diagnosis; treatment start.
   If not, check for mistakes.
9. Date of first symptom
   Date of first symptom is defined as date of first symptom from patient data.
10. Date of first presentation
    Date of first presentation to Primary Care is defined as (in the order of declining priority):
    a) date of first presentation to Primary Care from PCP data;
    b) date of first presentation to Primary Care and A&E from PCP data;
    c) date of first presentation to Primary Care from patient data.
11. Date of referral
    Date of referral is defined as date of referral from PCP data.
12. Date of screening
    Date of screening is defined as (in the order of declining priority):
    a) date of screening from registry;
    b) date of screening from patient data.
13. Date of diagnosis
    Definition
    a) If Registry reports both date of histological confirmation and date of confirming investigation, then use date of histological confirmation.
    b) Date of diagnosis (based on patient data, PCP data, specialist data, registry data) is defined as (in the order of declining priority):
       - date of diagnosis from registry;
       - date of histological confirmation (from specialist data, PCP data);
       - date of biopsy (from specialist data, PCP data);
       - date of confirming investigation (from specialist data, PCP data);
       - date of first hospital admission (from specialist data, PCP data);
       - date of MDT confirmation (from specialist data);
       - date patient was told (from specialist data, PCP data);
       - other date of diagnosis (from specialist data, PCP data, patient data);

    Choose a Date from a lower level of hierarchy, if the Date from a higher level is after the Date of consent or more than 9 months (=271 days) before the Date of consent.

    Exclusion criteria
    a) Unknown date of diagnosis;

For peer review only

b) Date of diagnosis is after the date of consent;
c) Date of diagnosis is more than 9 months before the Date of consent.

14. Date of treatment start
    a) Date of treatment start from patient data is defined as the earliest of the treatment dates for Surgery, Chemo, Radio and Other;
    b) Date of treatment start (based on registry data, specialist data, patient data) is defined as (in the order of declining priority):
        - date of treatment start from registry data,
        - date of treatment start from specialist data,
        - date of treatment start from patient data,
        - anticipated date of treatment from patient data.

15. Imputation of missing day in the date
    Imputation rules for missing day (given month and year are known):
    a) Set missing day to '16';
    b) Consider adjacent dates in a backwards order (from "Treatment" to "First symptom"). For each pair of such adjacent dates: If dates are not in a logical order (e.g. "Treatment" is before "Diagnosis"), but month and year are the same in both dates, and the day was imputed to '16' in one of the dates:
    - Recode the day imputed earlier to '16' to the day from the adjacent date.

16. Considering time
    If patient gave multiple answers to the "How long did you have symptoms before contacting a doctor?" question, then use the option with the shortest time interval.

17. Delay arranging appointment
    If patient gave multiple answers to the "How long did it take to get an appointment with PCP?" question, then use the option with the shortest time interval.

18. Duration of symptoms
    If PCP gave multiple answers to the "Duration of symptoms" question, then use the option with the shortest time interval.

19. Definition of Presentation
    A. Define Presentation within a Data Source (Patient, PCP)
    1. Review the free-text for Presentation (Patient, PCP) and re-code, if possible.
    2. If PCP reports 'VisitPCP and AE' or 'VisitPCP' as Presentation and no symptoms, then check Patient's records. If Patient reports 'Screening' and no symptoms, then re-code Presentation for this case as 'Screening'.
    3. If PCP reports 'Screening' as Presentation and at least one symptom (or "Duration of Symptoms", rule 18), then re-code Presentation to 'Other non-screen-detected'-option.
    4. If PCP reports 'Other' as Presentation and at least one symptom (or "Duration of Symptoms", rule 18), then re-code Presentation to 'Other non-screen-detected'-option.
    5. If Patient reports 'Screening' as Presentation and at least one symptom (or date of first symptom), then re-code Presentation to 'Other non-screen-detected'- option.
    6. If Patient reports 'Other' as Presentation and at least one symptom (or date of first symptom or "Considering time" (rule 16) or "Delay arranging appointment" (rule 17), then re-code Presentation to 'Other non-screen-detected'-option.
    7. In the case of multiple Presentation responses (Patient, PCP sources) - use a single option (in the order of declining priority):
        a) 'VisitPCP and AE',
        b) 'VisitPCP´, 'AE´ (if both 'VisitPCP´ and 'AE´ are given, then re-code as 'VisitPCP and AE'),
        c) 'Other non-screen-detected',
        d) 'Screening',
        e) 'Investigation for another problem' ,
        f) 'Other"

    B. Define Presentation from Alternative Data
    If Presentation hasn't been reported in either of data sources, then define it as (in the order of declining priority):
    1. 'Other non-screen-detected ', if PCP reports at least one symptom (or "Duration of symptoms", rule 18);
    2. 'Other non-screen-detected ', if Patient reports at least one symptom (or date of first symptom);
    3. 'Other non-screen-detected ', if Patient reports "Considering time" (rule 16) or "Delay arranging appointment" (rule 17) and no screening date;
    4. 'Screening', if Patient reports screening date and no symptoms and no date of first symptom;

    C. Define Presentation from Data Source Hierarchy

For peer review only

1. In Wales/England/Scotland (patients of 48-72 years old), Manitoba (patients 48-76 years old), N Ireland, Sweden and Denmark: if Registry reports 'Screening' – use Presentation-data from Registry data.
2. In Wales/England/Scotland (patients of 48-72 years old), Manitoba (patients 48-76 years old), N Ireland, Sweden and Denmark: if Registry reports ' No Screening'– use Presentation-data from (in the order of declining priority):
   a. PCP data (not available in Sweden);
   b. Patient data;

If PCP (or Patient, in the case of PCP data is not available) reports 'Screening', then code Presentation as 'Other non-screen-detected'.

If Presentation from PCP and Patient datasets is missing, then code Presentation as 'Other non-screen-detected'.

3. In Wales/England/Scotland (patients of 48-72 years old), Manitoba (patients 48-76 years old), N Ireland, Sweden and Denmark: if screening status from Registry is missing – use Presentation-data from (in the order of declining priority):
   a. PCP data (not available in Sweden);
   b. Patient data;

4. In Wales/England/Scotland (patients of <48 or >72 years old), Manitoba (patients of <48 or >76 years old), Norway and Ontario – use Presentation-data from (in the order of declining priority):
   a. PCP data;
   b. Patient data.

5. In Victoria: if Registry reports 'Screening organised by government' – use Presentation-data from Registry data.

6. In Victoria: if Registry reports 'No Screening organised by government' – use Presentation-data from (in the order of declining priority):
   a. PCP data;
   b. Patient data.

20. Patient interval
The Patient interval for non-screen-detected patients is defined as (in the order of declining priority):
a) "Date of first presentation to Primary Care" (rule 10) minus "Date of first symptom" (rule 9);
b) If the interval in (a) is unknown or negative: Calculate the interval as the middle of "Considering time" (rule 16) plus the middle of "Delay arranging appointment" (rule 17);
c) If the interval in (a) is unknown or negative and the interval in (b) is unknown: Calculate the interval as the middle of "Duration of symptoms interval" (rule 18).

21. Primary Care interval
The Primary Care interval for non-screen-detected is defined as "Date of referral" (rule 11) minus "Date of first presentation to Primary Care" (rule 10).

22. Diagnostic interval
a) The Diagnostic interval for non-screen-detected is defined as "Date of diagnosis" (rule 13) minus "Date of first presentation to Primary Care" (rule 10);
b) The Diagnostic interval for screen-detected patients is defined as "Date of diagnosis" (rule 13) minus "Date of screening" (rule 12).

23. Treatment interval
The Treatment interval is defined as "Date of treatment start" (rule 14) minus "Date of diagnosis" (rule 13).

24. Total interval
a) The Total interval for non-screen-detected patients is defined as "Date of treatment start" (rule 14) minus "Date of first symptom" (rule 9);
b) The Total interval for screen-detected patients is defined as "Date of treatment start" (rule 14) minus "Date of screening" (rule 12).

25. Range of Time intervals
The time intervals (Patient, Primary Care, Diagnosis, Treatment, Total) must be in range 0-1 year.

If > 1 year: set the interval to 365 days
If negative: set the interval to 0.

For each jurisdiction calculate the number of imputations due to:

For peer review only

a. unknown day in a date (given known month and year);<br>b. very large(>1 year) interval;<br>c. negative interval.
26. Number of visits<br>If patient gave multiple answers to the "Number of visits" questions, then use the option with a fewer number of visits.
27. Specialist waiting time interval<br>If patient gave multiple answers to the ""How long did it take to get an appointment with specialist?" question, then use the option with the shortest time interval.
28. Type of treatment<br>If patient ticked both "Yes" and "No" as answers to the "Type of treatment (Surgery, Chemotherapy, Radiotherapy)" questions, then choose "Yes" answer.
29. Health state<br>If patient gave multiple answers to the "Health state" question, then use the option with a better health condition.
30. Comorbidity<br>a) If patient ticked both "Yes" and "No" as answers to the "Comorbidity (Heart disease, Stroke, Lung disease, Diabetes)" questions, then choose "Yes" answer;<br>b) If both patient and PCP report "Comorbidity", then use the PCP Data.
31. Ethnicity<br>a) If patient didn't report "Ethnicity", then use the information from (in the order of declining priority):<br>- "Ethnicity_Other_Details";<br>- "Other main language spoken at home";<br>- "The main language spoken at home" (only for Victoria);<br>- "The main language spoken at home is the chief one for this jurisdiction"="Yes" given "Main language spoken at home is other than the main one for this jurisdiction"="No";<br>b) Consider Ethnicity as unknown, if answers to the "Ethnicity" question are multiple and belong to different categories ('white', 'Asian', 'black', 'other').
32. Education<br>If patient gave multiple answers to the "Education" question, then use the option with a higher level of education.
33. Smoking Current<br>a) If patient ticked both "Yes" and "No" as answers to the "Smoking Current" question, then use "Yes" answer;<br>b) If patient hasn't ticked neither "Yes" nor "No, then consider this case as Unknown.
34. Smoking Number<br>If patient reports "SmokingNumber" as text, then re-code using following rules:<br>a) Where there is a number smoked /day – accept number;<br>b) Where a range has been given – take the upper value;<br>c) Where patient has put 10+ or 20+ - capture this as 11 or 21;<br>d) Where number of cigarettes smoked in the past and currently being smoked are provided - average the numbers;<br>e) Non entries code as "." ;<br>f) Non-smokers (eg, "nil", "N/A") are coded as "0".
35. Smoked ever<br>a) If patient ticked both "Yes" and "No" as answers to the "Smoking ever" question, then use "Yes" answer;<br>b) If patient hasn't ticked neither "Yes" nor "No": consider it as "Yes", if patient is a current smoker ("Smoking_Current="Yes"") or has specified a number of cigarettes ("SmokingNumber">0). Otherwise consider this case as Unknown.<br>c) If patient has ticked "No": recode it to "Yes", if patient is a current smoker "Smoking_Current="Yes").
36. Nature of referral<br>a) Review free-text for " Nature of referral" (PCP Data) and re-code, if possible;<br>b) In the case of multiple responses, use a single option as (in the order of declining priority):<br>- "Referral for immediate admission";<br>- "Urgent referral";<br>- "Less urgent referral";<br>- "General referral" ;<br>- "No referral";<br>- "Other".
37. Refer Public or Private<br>a) If PCP ticked both "Public" and "Private" as answers to the "Refer Public or Private" question, then use "Private" answer;<br>b) If PCP hasn't ticked neither "Public" nor "Private", then consider this case as Unknown.

For peer review only

38. Type of referral

If specialist gave multiple responses to the "How was the patient referred…" question, then use a single option (in the order of declining priority):

- "Screening";
- "Respiratory clinic";
- "General surgery clinic";
- "General gynaecology";
- "Specialist/consultant";
- "PCP";
- "Other".

39. First Attendance Place

If specialist gave multiple responses to the "First Attendance Place" question, then consider this case as Unknown.

40. Stage-TNM

a) If specialist gave multiple responses to the "Stage_TNM" question, then use the highest category;

b) If registry gave multiple responses to the "Stage_TNM", then use a single option (in the order of declining priority):

- stage at time of diagnosis
- stage at surgery
- stage at oncology

c) If "Stage_TNM" is reported by both the specialist and registry, then use the registry data.

For peer review only

Additional File 5. *Comparisons of the participants and the study base of the eligible breast cancer women*

	Eligible women		Participating women		p-value
	n	(%)	n	(%)	
Women, total	19,419	(81.3%)	4,458	(18.7%)	
**Age**					
Under 40	83	(0.4%)	11	(0.2%)	
40-49	2,874	(14.8%)	676	(15.2%)	
50-59	4,756	(24.5%)	1,222	(27.4%)	
60-69	5,436	(28%)	1,434	(32.2%)	<0.001*
70-79	3,696	(19%)	791	(17.7%)	
80-89	2,092	(10.8%)	297	(6.7%)	
90+	383	(2.0%)	27	(0.6%)	
Missing	99	(0.5%)	0	(0.0%)	
**Deceased**					
3 months	176	(18.6%)	2	(5.1%)	
6 months	213	(22.5%)	5	(12.8%)	<0.05
9 months	236	(25.0%)	12	(30.8%)	
12 months	320	(33.9%)	20	(51.3%)	
**Tumour stage**					
I	7,220	(42.9%)	1,807	(49.0%)	
II	6,231	(37.0%)	1,311	(35.6%)	
III	2,028	(12.0%)	341	(9.3%)	<0.001*
IV	653	(3.9%)	87	(2.4%)	
missing	702	(4.2%)	139	(3.8%)	

* both with and without missing

For peer review only

Additional file 6. *The differences in interval lengths (days) between Wales, the reference, and the other nine jurisdictions.*

		Wales	England	Scotland	N Ireland	Denmark	Ontario	Norway	Manitoba	Victoria	Sweden
**Patient Interval (Symptomatic)**	Number	111	201	198	219	213	170	206	172	189	101
	Median	11 (ref.)	-3(-9,3)	-3 (-8,3)	-1 (-8,6)	**-7** (-12,-2)	7 (-1,16)	1 (-7,9)	4 (-2,11)	-4 (-9,2)	**17** (11,22)
	75th percentile	34 (ref.)	-7 (-21,7)	-4 (-12,3)	-2 (-8,5)	**-10** (-16,-4)	**19** (5,33)	**16** (7,25)	12 (-1,25)	-6 (-16,4)	**22** (8,36)
	90th percentile	73 (ref.)	21 (-19,61)	11 (-45,66)	30 (-13,73)	-14 (-64,35)	50 (-9,110)	**74** (31,117)	9 (-35,54)	33 (-13,80)	14 (-31,60)
**Diagnostic interval (Symptomatic)**	Number	111	200	197	216	207	166	203	184	191	101
	Median	29 (ref.)	**-18** (-22,-15)	**-10** (-14,-6)	**-16** (-19,-13)	**-22** (-25,-18)	**-6** (-9,-2)	**-10** (-15,-5)	-1 (-5,4)	**-18** (-22,-14)	**-17** (-23,-12)
	75th percentile	54 (ref.)	**-39** (-45,-33)	**-24** (-29,-19)	**-37** (-42,-33)	**-34** (-40,-28)	-3 (-13,6)	**-21** (-36,-6)	**-16** (-23,-8)	**-34** (-52,-15)	**-33** (-39,-27)
	90th percentile	92 (ref.)	**-68** (-88,-48)	**-58** (-91,-25)	**-62** (-80,-45)	**-55** (-75,-35)	**73** (35,112)	**-35** (-62,-8)	**-30** (-46,-15)	**-63** (-78,-47)	**-58** (-81,-35)
**Diagnostic interval (Screen-detected)**	Number	113	153	159	86	131	184	126	125	152	158
	Median	25 (ref.)	-11 (-22,1)	-7 (-18,5)	-7 (-19,4)	-1 (-17,15)	0 (-12,12)	1 (-11,13)	-2 (-15,10)	-5 (-17,6)	-12 (-24,0)
	75th percentile	45 (ref.)	**-24** (-36,-13)	**-17** (-28,-6)	**-20** (-31,-8)	-9 (-21,2)	-3 (-15,8)	-1 (-15,13)	-4 (-15,7)	**-14** (-25,-3)	**-21** (-34,-7)
	90th percentile	58 (ref.)	**-23** (-30,-16)	**-16** (-22,-11)	**-19** (-26,-12)	**-10** (-14,-6)	9 (-6,24)	8 (-3,20)	**55** (38,71)	**-17** (-26,-8)	**-17** (-20,-13)
**Diagnostic interval (All)**	Number	224	353	356	302	338	350	329	309	343	259
	Median	29 (ref.)	**-14** (-16,-12)	**-8** (-11,-6)	**-13** (-15,-11)	**-14** (-16,-11)	-2 (-5,1)	**-6** (-9,-2)	-1 (-5,2)	**-12** (-15,-10)	**-14** (-17,-11)
	75th percentile	49 (ref.)	**-30** (-34,-25)	**-18** (-23,-14)	**-29** (-33,-25)	**-20** (-25,-16)	-5 (-15,4)	**-11** (-20,-2)	**-8** (-12,-3)	**-22** (-26,-19)	**-25** (-32,-17)
	90th percentile	70 (ref.)	**-35** (-39,-31)	**-23** (-30,-16)	**-26** (-34,-19)	**-21** (-40,-2)	**20** (9,31)	-1 (-16,14)	**22** (7,38)	**-27** (-40,-13)	**-29** (-36,-21)
**Treatment interval (Symptomatic)**	Number	115	203	202	241	230	201	216	198	196	113
	Median	24 (ref.)	**6** (1,11)	1 (-3,5)	-3 (-7,1)	-3 (-7,1)	**12** (8,16)	-5 (-11,1)	**15** (11,19)	**-10** (-14,-6)	-2 (-6,2)
	75th percentile	33 (ref.)	**8** (4,12)	4 (-1,9)	-3 (-8,1)	-4 (-9,0)	**16** (11,21)	**-6** (-9,-2)	**22** (18,26)	**-9** (-16,-2)	-4 (-13,5)
	90th percentile	45 (ref.)	10 (-1,20)	11 (-4,26)	-4 (-19,11)	-1 (-14,12)	**20** (10,31)	-11 (-23,2)	**28** (10,46)	-11 (-26,4)	-3 (-15,10)
**Treatment interval (Screen-detected)**	Number	115	155	158	86	134	195	120	155	170	165
	Median	27 (ref.)	**5** (1,8)	**8** (4,11)	0 (-5,5)	**-6** (-10,-1)	**9** (7,12)	**-12** (-15,-10)	**12** (9,16)	-5 (-12,1)	**-3** (-6,-1)
	75th percentile	36 (ref.)	4 (-2,10)	10 (-3,22)	0 (-6,7)	-7 (-16,3)	**11** (3,20)	**-15** (-27,-4)	**15** (7,23)	-7 (-16,2)	-6 (-17,5)
	90th percentile	49 (ref.)	5 (-8,18)	**15** (4,25)	-9 (-20,3)	**-14** (-26,-2)	9 (-4,22)	**-24** (-35,-13)	12 (-1,24)	**-14** (-27,-1)	-11 (-21,0)
**Treatment interval (All)**	Number	231	359	361	330	368	400	341	353	366	278
	Median	25 (ref.)	5 (0,11)	4 (0,8)	-4 (-8,1)	**-5** (-9,-1)	**10** (6,14)	**-10** (-14,-6)	**14** (9,18)	**-10** (-14,-6)	-3 (-7,0)
	75th percentile	35 (ref.)	**6** (2,10)	**6** (1,10)	-4 (-8,0)	**-6** (-10,-2)	**13** (10,17)	**-11** (-16,-6)	**20** (16,23)	**-9** (-13,-5)	**-6** (-10,-2)
	90th percentile	46 (ref.)	**8** (2,14)	12 (-1,26)	**-6** (-10,-2)	**-6** (-9,-2)	**17** (11,23)	**-14** (-17,-12)	**23** (16,29)	**-11** (-14,-9)	**-7** (-10,-4)
**Total interval (Symptomatic)**	Number	104	190	186	205	189	173	184	155	177	98
	Median	70 (ref.)	**-14** (-24,-3)	**-12** (-23,-2)	**-20** (-31,-8)	**-25** (-38,-11)	**28** (15,42)	-15 (-34,5)	**22** (12,31)	**-28** (39,-17)	-1 (-11,10)

	75th percentile	96 (ref.)	**-16** (-27,-5)	-5 (-18,9)	**-18** (-33,-3)	**-25** (-39,-12)	**69** (31,107)	**27** (8,46)	**30** (8,53)	-13 (-27,0)	3 (-30,35)
	90th percentile	218 (ref.)	**-71** (-102,-41)	**-60** (-85,-34)	**-57** (-66,-48)	**-41** (-66,-16)	**79** (66,93)	8 (-15,30)	**-17** (-34,-1)	-21 (-67,26)	**-39** (-67,-10)
**Total interval (All)**	Number	217	343	337	291	320	354	298	277	327	253
	Median	60 (ref.)	**-9** (-14,-3)	-4 (-11,2)	**-13** (-18,-8)	**-15** (-26,-4)	**16** (6,26)	**-13** (-21,-6)	**17** (9,24)	**-18** (-24,-12)	**-17** (-25,-10)
	75th percentile	81 (ref.)	**-12** (-20,-4)	1 (-9,10)	**-13** (-20,-6)	**-14** (-20,-9)	**32** (24,41)	-4 (-12,4)	**35** (21,48)	**-19** (-25,-12)	**-17** (-26,-7)
	90th percentile	123 (ref.)	-13 (-34,9)	3 (-12,18)	-5 (-22,11)	-8 (-38,22)	**73** (33,114)	**32** (2,62)	**47** (13,82)	-12 (-37,13)	-24 (-51,4)

The differences for the median, 75th and 90th percentiles are calculated as marginal effects after quantile regression by setting the continuous covariate age to its mean value and comorbidity to the mode. Significant results are shown in bold. The actual number of days are included for Wales are shown in Table 5. Note that analyses of primary care intervals are presented in Table 6.

For peer review only

Additional File 7. *Agreement between the ICBP M4 Presentation-rule (without using registry data) and registry information to define if a breast cancer case was screen detected, in Wales, England, Scotland, N Ireland, Denmark, Manitoba and Sweden*

Jurisdiction	Number of cases	Agreement on screening-status between ICBP M4 and Registry %	Kappa	(95%CI)
Wales	227	96	0.91	(0.86-0.97)
England	341	95	0.89	(0.84-0.93)
Scotland	360	97	0.94	(0.90-0.97)
N Ireland	331	91	0.77	(0.69-0.84)
Denmark	342	91	0.81	(0.74-0.87)
Manitoba	364	84	0.68	(0.60-0.76)
Sweden	284	87	0.75	(0.67-0.82)

For peer review only

Additional File 8. ICBP M4 ethics, working and academic reference groups

Section 1 - Summary of the (ethical) approvals obtained in the ten jurisdictions for local data collection and data transfer to University College London (United Kingdom) and Aarhus University (Denmark).

	Date of Ethics Approval	Approvals obtained	Reference
Victoria	4 September 2012	Cancer Council Victoria Human Research Ethics Committee	HREC 1125
Manitoba	7 March 2013<br>15 April 2013	Health Research Ethics Board, University of Manitoba<br>Research Resource Ethics Committee, CancerCare Manitoba	HS15227 (H2012:105)<br>RRIC#28-2012
Ontario	7 November 2013<br>28 January 2014	University of Toronto Research Ethics Board	27881
Denmark	6 August 2013<br>19 June 2013	The Danish Data Protection Agency<br>According to Danish law and the Central Denmark Region Committees on Health Research Ethics, approval by the National Committee on Health Research Ethics was not required as no biomedical intervention was performed.	2013-41-2030<br>1-10-72-20-13
Sweden	23 October 2013	Ethics Review Board, Uppsala	2013/306
Norway	04 April 2013	Regional committees for medical and health research ethics	2013/136/REK nord
Wales	16 November 2012	NRES Committee East Midlands – Derby 2,<br>local R&D for each health board	11/EM/0420
Scotland	16 November 2012	NRES Committee East Midlands – Derby 2,<br>R&D for each health board,<br>Privacy Advisory Committee,<br>CHI Advisory Group	11/EM/0420
N Ireland	1 June 2012	ORECNI Ethical approval, local governance for each health Trust	12/NI/0053
England	16 November 2012	NRES Committee East Midlands – Derby 2<br>R&D for each Clinical Research Network	11/EM/0420

For peer review only

Section 2 – ICBP Module 4 Working Group

Alina Zalounina Falborg, Statistician, Research Unit for General Practice, Department of Public Health, Aarhus University, Bartholins Allé 2, 8000 Aarhus C, Denmark

Andriana Barisic, Research Associate, Department of Prevention and Cancer Control, Cancer Care Ontario, 620 University Avenue, Toronto, Ontario, M5G 2L7, Canada

Anna Gavin, Director, Northern Ireland Cancer Registry, Centre for Public Health, Queen's University Belfast, Mulhouse Building, Mulhouse Road, Belfast, BT12 6DP, United Kingdom

Anne Kari Knudsen, Administrative leader, Department of Cancer Research and Molecular Medicine, Norwegian University of Science and Technology, 7489 Trondheim, Norway

Breann Hawryluk, Project Planning Coordinator, Department of Patient Navigation, Cancer Care Manitoba, 675 McDermot Street, Winnipeg, Manitoba, Canada

Chantelle Anandan, Research Fellow, Centre for Population Health Sciences, University of Edinburgh, Doorway 1, Medical Quad Teviot Place, Edinburgh, EH8 9DX, United Kingdom

Conan Donnelly, Research Fellow, Centre for Public Health, Queen's University Belfast, Mulhouse Building, Mulhouse Road, Belfast, BT12 6DP, United Kingdom

David H Brewster, Scottish Cancer Registry, Information Services Division, NHS National Services Scotland, Gyle Square, 1 South Gyle Crescent, Edinburgh, EH12 9EB, United Kingdom

David Weller, James Mackenzie Professor of General Practice, Centre for Population Health Sciences, University of Edinburgh, Doorway 1, Medical Quad Teviot Place, Edinburgh, EH8 9DX, United Kingdom

Donna Turner, Epidemiologist/Provincial Director, Population Oncology, Cancer Care Manitoba, 675 McDermot Street, Winnipeg, Manitoba, Canada

Elizabeth Harland, Project Coordinator, Department of Epidemiology and Cancer Registry, CancerCare Manitoba, 675 McDermot Street, Winnipeg, Manitoba

Eva Grunfeld, Director, Knowledge Translation Research Network Health Services Research Program, Ontario Institute for Cancer Research; Professor and Vice Chair Research, Department of Family and Community Medicine, University of Toronto, 500 University Avenue, Toronto, Ontario, M5G 1V7, Canada

Evangelia Ourania Fourkala, Research Associate, Gynaecological Cancer Research Centre, Women's Cancer, Institute for Women's Health, University College London, United Kingdom

Henry Jensen, Research fellow, Research Unit for General Practice, Department of Public Health, Aarhus University, Bartholins Allé 2, 8000 Aarhus C, Denmark

Jackie Boylan, Research Fellow, Centre for Public Health, Queen's University Belfast, Mulhouse Building, Mulhouse Road, Belfast, BT12 6DP, United Kingdom

Jacqueline Kelly, Tumour Verification Officer, Northern Ireland Cancer Registry, Centre for Public Health, Queen's University Belfast, Mulhouse Building, Mulhouse Road, Belfast, BT12 6DP, United Kingdom

Kerry Moore, Research Fellow, Centre for Public Health, Queen's University Belfast, Mulhouse Building, Mulhouse Road, Belfast, BT12 6DP, United Kingdom

Maria Rejmyr Davis, Head, Southern Sweden Regional Cancer Center, Medicon Village, Scheelevägen 8, building 404, 223 81 Lund, Sweden

Martin Malmberg, MD PhD, Senior Consultant, Department of Oncology, Lund University Hospital, SE-221 85 Lund, Sweden

Mats Lambe, Professor of Medical Epidemiology, Regional Cancer Center Uppsala and Department of Medical Epidemiology and Biostatics, Karolinska Institutet, SE-171 77 Stockholm, Sweden

For peer review only - http://bmjopen.bmj.com/site/about/guidelines.xhtml

Oliver Bucher, Epidemiologist, Department of Epidemiology and Cancer Registry, CancerCare Manitoba, 675 McDermot Street, Winnipeg, Manitoba

Peter Vedsted, Professor, Research Unit for General Practice, Department of Public Health, Aarhus University, Bartholins Allé 2, 8000 Aarhus C, Denmark

Rebecca Bergin, Senior Research Officer/ PhD Candidate, Centre for Behavioural Research in Cancer, 615 St Kilda Rd, Melbourne, Victoria, 3004, Australia

Rebecca-Jane Law, Research Project Support Officer, North Wales Centre for Primary Care Research, Bangor University, Gwenfro Units 4-8, Wrexham Technology Park, Wrexham, LL13 7YP, United Kingdom

Richard D Neal, Professor of Primary Care Oncology, Academic Unit of Primary Care, Leeds Institute of Health Sciences, University of Leeds, Leeds LS2 9NL, United Kingdom

Sigrun Saur Almberg, Researcher, Department of Cancer Research and Molecular Medicine, Faculty of Medicine, Norwegian University of Science and Technology (NTNU), N-7491 Trondheim, Norway

Therese Kearney, Research fellow, Northern Ireland Cancer Registry, Centre for Public Health, Queen's University Belfast, Mulhouse Building, Mulhouse Road, Belfast, BT12 6DP, United Kingdom

Tindie Kalsi, Project Manager, Gynaecological Cancer Research Centre, Women's Cancer, Institute for Women's Health, University College London, United Kingdom

Victoria Cairnduff, Statistician, Northern Ireland Cancer Registry, Centre for Public Health, Queen's University Belfast, Mulhouse Building, Mulhouse Road, Belfast, BT12 6DP, United Kingdom

Victoria Hammersley, Researcher, Centre for Population Health Sciences, University of Edinburgh, Doorway 1, Medical Quad Teviot Place, Edinburgh, EH8 9DX, United Kingdom

Victoria White, Deputy Director, Centre for Behavioral Research in Cancer, Cancer Council Victoria, 615 St Kilda Road, Melbourne, Victoria, 3004, Australia

Usha Menon, Professor of Gynaecological Oncology and Head, Gynaecological Cancer Research Centre, Women's Cancer, Institute for Women's Health, University College London, United Kingdom

Yulan Lin, Postdoc, Department of Cancer Research and Molecular Medicine, Faculty of Medicine, Norwegian University of Science and Technology (NTNU), N-7491 Trondheim, Norway

For peer review only

Section 3 – ICBP Module 4 Academic Reference Group:

Prof Jan Willem Coebergh, Professor of Cancer Surveillance, Department of Public Health, Erasmus Universiteit Rotterdam, Rotterdam, the Netherlands

Jon Emery, Professor of Primary Care Cancer Research, University of Melbourne and Clinical Professor of General Practice, University of Western Australia, Australia

Dr Stefan Bergström, Senior consultant oncologist, Department of Oncology, Gävle, Sweden

Dr Monique E van Leerdam, Erasmus MC University Medical Centre, the Netherlands

Prof Marie-Louise Essink-Bot, Academic Medical Centre, Amsterdam University, the Netherlands

Prof Una MacLeod, Senior Lecturer in General Practice and Primary Care, Hull-York Medical School, United Kingdom

BMJ Open

Diagnostic pathways for breast cancer in ten International Cancer Benchmarking Partnership (ICBP) jurisdictions: An International comparative cohort study based on questionnaire and registry data

Journal:	*BMJ Open*
Manuscript ID	bmjopen-2021-059669.R2
Article Type:	Original research
Date Submitted by the Author:	17-Nov-2022
Complete List of Authors:	Vedsted, Peter; Aarhus Universitet<br>Weller, David; University of Edinburgh, General Practice<br>Zalounina Falborg, Alina; Research Unit for General Practice, Department of Public Health, Aarhus University<br>Jensen, Henry; Research Unit for General Practice, Department of Public Health, Aarhus University; Research Unite for General Practice, Department of Public Health, Aarhus university<br>Kalsi, Jatinderpal; University College London, Gynaecological Cancer Research Centre<br>Brewster, David; NHS National Services Scotland, Scottish Registry, Information Services Division<br>Lin, Yulan; Fujian Medical University, Department of Epidemiology and Health Statistics, School of Public Health<br>Gavin, Anna; Queen's University Belfast, N. Ireland Cancer Registry<br>Barisic, Andriana; Cancer Care Ontario, Renal Network<br>Grunfeld, Eva; Ontario Institute for Cancer Research, Dept. of Family and Community Medicine<br>Lambe, Mats; Regional Cancer Centre of central Sweden, University Hospital<br>Malmberg, Martin; Lund University Hospital, 9. Department of Oncology<br>Turner, Donna; Cancer Care Manitoba, Population Oncology<br>Harland, Elizabeth; CancerCare Manitoba, Department of Epidemiology and Cancer Registry<br>Hawryluk, Breann; CancerCare Manitoba, Patient Navigation<br>Law, Rebecca-Jane; Bangor University, North Wales Centre for Primary Care Research<br>Neal, Richard; University of Leeds,<br>White, Victoria; Cancer Council Victoria, CBRC; Deakin University Faculty of Health,<br>Bergin, Rebecca; Cancer Council Victoria, Centre for Behavioral Research in Cancer<br>Harrison, Samantha; Cancer Research UK, Policy and Information<br>Menon, Usha; University College London, Women's Cancer<br>Working Group, ICBP; Cacncer Research UK, Policy and Information
**Primary Subject Heading**:	Public health

Secondary Subject Heading:	Diagnostics, Epidemiology, Medical management
Keywords:	Breast tumours < ONCOLOGY, Epidemiology < ONCOLOGY, Adult oncology < ONCOLOGY, International health services < HEALTH SERVICES ADMINISTRATION & MANAGEMENT, PUBLIC HEALTH

SCHOLARONE™
Manuscripts

I, the Submitting Author has the right to grant and does grant on behalf of all authors of the Work (as defined in the below author licence), an exclusive licence and/or a non-exclusive licence for contributions from authors who are: i) UK Crown employees; ii) where BMJ has agreed a CC-BY licence shall apply, and/or iii) in accordance with the terms applicable for US Federal Government officers or employees acting as part of their official duties; on a worldwide, perpetual, irrevocable, royalty-free basis to BMJ Publishing Group Ltd ("BMJ") its licensees and where the relevant Journal is co-owned by BMJ to the co-owners of the Journal, to publish the Work in this journal and any other BMJ products and to exploit all rights, as set out in our licence.

The Submitting Author accepts and understands that any supply made under these terms is made by BMJ to the Submitting Author unless you are acting as an employee on behalf of your employer or a postgraduate student of an affiliated institution which is paying any applicable article publishing charge ("APC") for Open Access articles. Where the Submitting Author wishes to make the Work available on an Open Access basis (and intends to pay the relevant APC), the terms of reuse of such Open Access shall be governed by a Creative Commons licence – details of these licences and which Creative Commons licence will apply to this Work are set out in our licence referred to above.

Other than as permitted in any relevant BMJ Author's Self Archiving Policies, I confirm this Work has not been accepted for publication elsewhere, is not being considered for publication elsewhere and does not duplicate material already published. I confirm all authors consent to publication of this Work and authorise the granting of this licence.

For peer review only

Diagnostic pathways for breast cancer in ten International Cancer Benchmarking Partnership (ICBP) jurisdictions: An International comparative cohort study based on questionnaire and registry data

Corresponding author:

Peter Vedsted

Research Unit for General Practice, Aarhus University

Bartholins Alle 2, 8000, Aarhus, Denmark

p.vedsted@ph.au.dk

1. **Peter Vedsted** *, Research Unit for General Practice, Department of Public Health, Aarhus University, Bartholins Allé 2, 8000 Aarhus C, Denmark, p.vedsted@ph.au.dk
2. **David Weller** *, Centre for Population Health Sciences, University of Edinburgh, Doorway 1, Medical Quad Teviot Place, Edinburgh, EH8 9DX, United Kingdom, david.weller@ed.ac.uk
3. **Alina Zalounina Falborg**, Research Unit for General Practice, Department of Public Health, Aarhus University, Bartholins Allé 2, 8000 Aarhus C, Denmark, az@ph.au.dk
4. **Henry Jensen**, Research Unit for General Practice, Department of Public Health, Aarhus University, Bartholins Allé 2, 8000 Aarhus C, Denmark, henry.jensen@feap.dk
5. **Jatinderpal Kalsi**, Gynaecological Cancer Research Centre, Women's Cancer, Institute for Women's Health, University College London, United Kingdom, j.k.kalsi@ucl.ac.uk
6. **David H Brewster**, Scottish Cancer Registry, Information Services Division, NHS National Services Scotland, Gyle Square, 1 South Gyle Crescent, Edinburgh, EH12 9EB, United Kingdom, David.Brewster@isd.csa.scot.nhs.uk
7. **Yulan Lin**, Department of Epidemiology and Health Statistics, School of Public Health, Fujian Medical University, Fuzhou 350122, China.
8. Department of Cancer Research and Molecular Medicine, Faculty of Medicine, Norwegian University of Science and Technology (NTNU), N-7491 Trondheim, Norway, yulanlin@fjmu.edu.cn
9. **Anna Gavin**, Northern Ireland Cancer Registry, Centre for Public Health, Queen's University Belfast, Mulhouse Building, Mulhouse Road, Belfast, BT12 6DP, United Kingdom, A.Gavin@qub.ac.uk
10. **Andriana Barisic,** Department of Prevention and Cancer Control, Cancer Care Ontario, 620 University Avenue, Toronto, Ontario, M5G 2L7, Canada, Andriana.Barisic@renalnetwork.on.ca
11. **Eva Grunfeld**, Department of Family and Community Medicine, University of Toronto, 500 University Avenue, Toronto, Ontario, M5G 1V7, Canada, eva.grunfeld@utoronto.ca
12. **Mats Lambe**, Regional Cancer Center Uppsala and Department of Medical Epidemiology and Biostatics, Karolinska Institutet, SE-171 77 Stockholm, Sweden, mats.lambe@ki.se
13. **Martin Malmberg**, Department of Oncology, Lund University Hospital, SE-221 85 Lund, Sweden, Martin.Malmberg@skane.se
14. **Donna Turner**, Population Oncology, Cancer Care Manitoba, 675 McDermot Street, Winnipeg, Manitoba, Canada, donna.turner@cancercare.mb.ca

For peer review only

15. **Elizabeth Harland**, Department of Epidemiology and Cancer Registry, CancerCare Manitoba, 675 McDermot Street, Winnipeg, Manitoba, eharland@cancercare.mb.ca
16. **Breann Hawryluk**, Department of Patient Navigation, Cancer Care Manitoba, 675 McDermot Street, Winnipeg, Manitoba, Canada, Breann_haw@hotmail.com
17. **Rebecca-Jane Law**, North Wales Centre for Primary Care Research, Bangor University, Wrexham, LL13 7YP, United Kingdom, r.law@bangor.ac.uk
18. **Richard D Neal**, Academic Unit of Primary Care, Leeds Institute of Health Sciences, University of Leeds, Leeds LS2 9NL, United Kingdom, r.d.neal@leeds.ac.uk
19. **Victoria White,** Centre for Behavioral Research in Cancer, Cancer Council Victoria, 615 St Kilda Road, Melbourne, Victoria, 3004, Australia, vicki.white@cancervic.org.au
20. **Rebecca J Bergin,** Cancer Epidemiology Division at Cancer Council Victoria, 615 St Kilda Road, Melbourne, Victoria, 3004, Australia, Rebecca.Bergin@cancervic.org.au
21. **Samantha Harrison,** Policy, Information & Communications, Cancer Research UK, 2 Redman Place, London, E20 1JQ, UK, samantha.harrison@cancer.org.uk
22. **Usha Menon** *, MRC Clinical Trials Unit, Institute of Clinical Trials and Methodology, University College London, United Kingdom, u.menon@ucl.ac.uk
23. **ICBP Module 4 Working Group**

* equal contribution

Number of words: 3598
Number of tables: 6
Number of figures: 1
Number of Appendices: 7

For peer review only

For peer review only - http://bmjopen.bmj.com/site/about/guidelines.xhtml

Abstract

Objectives*:* A growing body of evidence suggests longer times between symptom onset and start of treatment affects breast cancer prognosis. To explore this association the International Cancer Benchmarking Partnership Module 4 examined differences in breast cancer diagnostic pathways in 10 jurisdictions across Australia, Canada, Denmark, Norway, Sweden and the UK

Design*:* Data were collated from 3,471 women aged >40 diagnosed for the first-time with breast cancer and surveyed between 2013 and 2015. Data were supplemented by feedback from their primary care physician (PCP), cancer treatment specialists, and available registry data.

Results: Overall, 56% of women reported symptoms to primary care, with 66% first noticing lump/s or breast changes. PCPs reported 77% presented with symptom/s, of whom 81% were urgently referred with suspicion of cancer, (ranging 62-92%, Norway; Victoria). Ranges for the median patient, primary care and diagnostic intervals (days) for symptomatic patients were: 3-29 (Denmark; Sweden), 0-20 (seven jurisdictions; Ontario), 8-29 (Denmark; Wales). Ranges for the median treatment and total intervals (days) for all patients were: 15-39 (Norway, Victoria; Manitoba) and 4 -78 days (Sweden, Victoria; Ontario). The 10% longest waits ranged between 101-209 days (Sweden; Ontario).

Conclusions: Large international differences in breast cancer diagnostic pathways exist, suggesting some jurisdictions develop more effective strategies to optimise pathways and reduce time intervals. Targeted awareness interventions could also facilitate more timely diagnosis of breast cancer.

Keywords

Delayed diagnosis; Questionnaire; International comparisons, Early Detection of Cancer; Breast neoplasms; Health services, Health planning; Signs and Symptoms; Screening

For peer review only

Strengths and limitations of this study

- Internationally standardised survey methodology to explore and compare key intervals from symptom onset to start of treatment.
- Comprehensive data extracted from state/provincial-level cancer registries and other sources to create as complete a record as possible of patient pathways to diagnosis and treatment.
- Minimal recall bias through triangulation of different data sources and by ensuring that the patients received the questionnaire with a limited time window after the cancer diagnosis.
- Some jurisdictions were not able to recruit a sufficient number of patients to meet power requirements.
- The cohort is not representative of all breast cancer patients as there was high self-selection of early-stage breast cancer patients

Introduction

Breast cancer is the most common cancer among women in western countries. The incidence of female breast cancer is around 80 per 100,000 (standardised world population); by the age of 80, approximately 12% of women will have received a breast cancer diagnosis [1]. Although the prognosis of breast cancer has improved dramatically during recent decades [2], there are still important differences in disease-specific mortality and survival, both between and within countries [3,4]. For example, in 2010-14, the 5-year net survival varied from 85.6% in the UK, to 88.8% in Sweden, and 89.5% in Australia [2].

Differences in survival may relate to timely cancer diagnosis and access to optimal treatment and can also affect patient experience and healthcare costs [5-10]. Examining international differences in routes to diagnosis and treatment, together with time intervals from first noticing symptoms until start of treatment, may help explain these differences.

For peer review only - http://bmjopen.bmj.com/site/about/guidelines.xhtml

Women differ in their help-seeking behaviours for breast cancer symptoms [11]. These behaviours might influence the time interval between first noticing symptoms until they present to a healthcare professional [12]. Further, organisational features of a healthcare system might affect the way patients seek help, how healthcare professionals respond to these symptoms, and when and how they can refer for further investigations [13-16]. In some countries, breast cancer diagnosis is standardised or expedited with urgent referrals, where significant breast cancer specific 'red flag symptoms' are present – further, many countries have now implemented breast cancer screening programmes [17]. Therefore, routes to diagnosis and time intervals from first noticing symptoms to breast cancer diagnosis and treatment may vary between countries. This has consequences for patient outcomes and experience, as well as healthcare costs.

To date almost all breast cancer routes to diagnosis studies have been based within a single country [18]; international comparisons can shed light on factors which underpin observed differences. Accordingly, in this paper we explore pathways to diagnosis and treatment for female breast cancer in ten jurisdictions across six countries.

Methods

We undertook an international comparative cohort study based on questionnaire and registry data on newly diagnosed, first-time female breast cancer patients aged 40 or over.

Study context and management

Within the International Cancer Benchmarking Partnership (ICBP), Module 4 (ICBP M4) aims to explore differences in routes to diagnosis for four cancers; the methods used are described in detail elsewhere [19,20]. Briefly, the study was undertaken in ten jurisdictions across six countries: Australia (Victoria), Canada (Manitoba and Ontario), Denmark, Norway, Sweden and the United Kingdom (England, Northern Ireland, Scotland and Wales). We recruited jurisdictions with universal health care access (with coverage including breast cancer investigations and screening programmes) and showing variation in breast cancer survival [20].

For peer review only - http://bmjopen.bmj.com/site/about/guidelines.xhtml

Identification of study population

Eligible patients were women aged 40 years or more with a first diagnosis of invasive breast cancer (ICD10 codes C50.0—C50.9), irrespective of symptomatic or screen-based diagnosis. Women with previous diagnoses of other cancers were included but women with a previous diagnosis of breast cancer or synchronous breast cancer were excluded.

Participants were identified via cancer registries and hospital databases, although processes varied locally. Processes were tested in each jurisdiction to ensure adaptation of data collection, questionnaire logistics and data management to the local settings [20]. Each jurisdiction aimed to recruit 200 symptomatic breast cancer patients, irrespective of the number of screen-based diagnoses. In Ontario and Victoria, the sampling continued beyond 200 patients for use in local studies. In Northern Ireland, the majority of screen-detected women were excluded at the sampling stage by the cancer registry.

Patient and Public Involvement

The development of the research question, the questionnaire and the presentation was done in collaboration with patient representatives and cancer charities. Both in the jurisdictions where the questionnaire was developed, evaluated and validated among patients and GPs and as part of the collaborative work in the International Cancer Benchmarking Partnership. Patients were involved in the design of how to approach a person with a new cancer diagnosis. The results of this study will be disseminated through the various cancer charities in each jurisdiction.

Data sources

Data were collected primarily from three questionnaires sent to eligible women (Additional File 1), their primary care physician (PCP; Additional File 2) and their cancer treatment specialists (CTSs; Additional File 3). This was supplemented with registry data (including diagnosis date, tumour stage and screening) in all

For peer review only - http://bmjopen.bmj.com/site/about/guidelines.xhtml

jurisdictions except Ontario and Norway [19]. Data were anonymised prior to transfer to Aarhus University (Denmark) for analysis.

Questionnaire and registry data

The validated questionnaires included items on routes to diagnosis, symptoms, specific milestones from first noticing symptoms to starting treatment, patient socio-demographics and comorbidity [20]. Eligible patients were identified from cancer registries, and patients were either contacted directly or via their PCP.

Women were sent questionnaires 3-9 months after diagnosis; PCPs and CTSs were sent questionnaires after patients consented to participate in the study. Completed questionnaires were returned to the local research teams. Where possible, registries and disease databases provided additional checks and information on date of diagnosis, screening status and start of treatment. Date of diagnosis was based on the International Agency for Research on Cancer (IARC) hierarchy, and tumour stage using tumour, node, metastasis (TNM) classification [21,22].

Data handling

Curation of questionnaire data was undertaken primarily at a single location (Aarhus University, Demark) to ensure uniform application across jurisdictions.

A set of algorithms (or ‘rules’) to standardise the validation and combination of data from the different sources was developed (Additional File 4); they employed a hierarchy principle with regard to the order in which different data sources were used. Any queries were discussed and resolved with the study team.

Routes to diagnosis

Differences in the diagnostic routes were investigated drawing on the checklist from the Aarhus Statement [23]. Routes to diagnosis are outlined in Box 1:

For peer review only - http://bmjopen.bmj.com/site/about/guidelines.xhtml

BOX 1 – Routes to diagnosis

- **Screening**
- **Symptomatic:**
  - Visit PCP
  - Visit PCP and then accident and emergency department (A&E)
  - Direct to A&E
  - Investigation for another problem
- **Other/unknown routes to diagnosis**

Measures of time intervals

Time interval definitions were adapted from the Aarhus Statement and included the time-points outlined in Box 2 [23]; the intervals are represented graphically in Figure 1.

BOX 2 – Time intervals

a) First onset of symptoms: the time-point when first bodily change(s) and/or symptom(s) were first noticed by the patient.
b) First presentation to healthcare: the time-point at which the PCP noted a symptom that in retrospect could be due to the underlying cancer.
c) First referral to secondary care: the time-point at which the PCP referred the patient (and responsibility) to secondary/specialist care.
d) Date of diagnosis, following the IARC definition.[21]
e) Date of treatment start: when the patient started curative/palliative treatment (in Manitoba only curative treatment was registered).

INSERT FIGURE ONE ABOUT HERE

For screen-detected women, we used the date of the screening test as the start of the diagnostic and total interval measurements. The analyses include intervals for both symptomatic, screen-detected and all breast cancer patients. We included a categorical variable where the symptomatic women reported the waiting time for their appointment with the PCP. Cancers were only considered to be screen-detected if the woman or PCP had indicated a screening route and there were no symptoms nor symptomatic pathway reported. For Wales, England, Scotland, Northern Ireland, Denmark, Manitoba and Sweden the distinction between a

For peer review only - http://bmjopen.bmj.com/site/about/guidelines.xhtml

screen-detected and non-screen-detected breast cancer was based on registry data from public screening programmes (Table 1).

INSERT TABLE 1 ABOUT HERE

Covariates

General health status was measured using the self-reported general health item from the 12-Item Short Form Health Survey (SF12) [24]. Comorbidity was assessed through the patient questionnaire as presence of any of four chronic diseases (heart or lung diseases, stroke, or diabetes) and categorised into 'none', 'medium' (one or two), or 'high' (three or four). Educational level (from the patient questionnaire), was categorised as 'low' (vocational school or lower) and 'high' (university or higher). Smoking was categorised into 'current', 'past' or 'never' smoker. Symptoms reported by the PCP were divided into 'breast cancer specific symptoms' or 'other symptoms'. This was based on symptom coding, drawing on local clinical guidelines [17,24-25].

Statistical analysis

Patients with missing data on age, date of diagnosis and/or date of consent were excluded. Quantile regression [26] was used to estimate differences in intervals at the 50th, 75th and 90th percentiles between all jurisdictions, with Wales as the reference. Intervals were derived by counting days, using the 'qcount' procedure [27,28]. The differences in intervals were calculated as marginal effects after quantile regression by setting the continuous covariates to their mean values and the categorical covariates to their modes.

Due to zero-inflation, quantile regression could not be used for the primary care interval. Here a generalised linear model with Poisson family, log link and robust error variance was used to calculate the association based on prevalence ratio (PR), between longer primary care intervals and jurisdiction. Longer intervals were defined as those over 5 or 14 days to test both intervals. The analyses were adjusted for age (as a continuous variable) and comorbidity (as a categorical variable). The significance level was set to 0.05 or less, and 95%

For peer review only - http://bmjopen.bmj.com/site/about/guidelines.xhtml

confidence intervals (95%CI) were calculated when appropriate. Statistical analyses were carried out using STATA v14 software.

Sensitivity and validity analyses

Analyses were repeated using the per-protocol definition of a maximum of 6 months between diagnosis and patient consent, to assess the impact of including patients who consented at 9 months post-diagnosis. To estimate the effect of using patient-reported intervals only, a sensitivity analysis based solely on the patient questionnaire data was performed.

Kappa coefficients were used to assess the agreement on routes to diagnosis (screening and symptomatic presentation) between the different data sources. Lin's concordance correlation coefficient (CCC) was used to assess the agreement on dates between the different data sources [29].

Results

Patient characteristics and participation

Across all jurisdictions, 15,421 eligible women were identified between May 2013 and August 2015. A total of 4,593 (39.9%) of those contacted returned completed questionnaires of which 3,471 (75.6%) were included in the analyses. The patient flow, with identification, exclusion and responses for each jurisdiction is detailed in Supplementary Table 1. The response rates ranged from 24.1% in Norway to 77.9% in Denmark (Supplementary Table 1). Participating women were younger and had a less advanced tumour stage distribution than the total sample of women eligible for inclusion (Additional File 5). The characteristics of the included women are detailed in Table 1.

INSERT TABLES 2 & 3 ABOUT HERE

First presentation and symptoms

For peer review only - http://bmjopen.bmj.com/site/about/guidelines.xhtml

Table 2 shows routes to diagnosis; 42.9% had a screen-detected breast cancer, with the highest proportion in Sweden (58.6%)[1]. For symptomatic patients, primary care was the place of first presentation for 85.6% of women, ranging from 69.2% in Ontario to 93.7% in England. According to the PCP, 81.1% of symptomatic women were urgently referred with a suspicion of cancer, ranging from 62.4% in Norway to 92.2% in Victoria (data not shown).

Table 3 shows the first symptom or sign reported by women, together with those reported by the PCP (for Sweden, only patient data were collected). Two thirds of symptomatic women noticed a lump or change in the breast. This was also the most commonly reported sign by PCPs, followed by pain/tenderness, and change in the nipple. According to the PCP, 77% of women presented with a symptom or sign indicative of breast cancer. While one in ten women indicated fatigue as a symptom, this was rarely noted by the PCPs.

INSERT TABLES 4 ABOUT HERE

Time intervals

The median patient interval for symptomatic women varied from 3 days in Denmark to 29 days in Sweden – see Table 4. Based on the quantile regression, the patient interval was 7 days shorter in Denmark and 17 days longer in Sweden compared with Wales (Figure 2 and Additional File 6). When comparing the 90th percentile, Norway (157 days) and Ontario (142 days) had the longest patient intervals (Table 4). Quantile regression showed that Norway had a significantly longer patient interval for the 90th percentile compared with Wales (74 days) (Figure 2).

INSERT FIGURE 2 ABOUT HERE

[1] *note that the proportion of screen-detected cancers in Northern Ireland is lower due to the initial exclusion of screen-detected cancers*

For peer review only - http://bmjopen.bmj.com/site/about/guidelines.xhtml

The median primary care interval for symptomatic women (in days) was zero, except in the two Canadian provinces and Victoria (Table 4). Women in these three jurisdictions had longer primary care intervals (both for >5 and >14 days) than women in Wales; this was statistically significant (Table 5).

INSERT TABLE 5 ABOUT HERE

The median diagnostic interval for symptomatic women ranged from 8 days in Denmark to 29 days in Wales (Table 4). At the 90th percentile, the diagnostic interval ranged from 36 days in England to 202 days in Ontario (Table 4). Compared with Wales, all jurisdictions except Ontario had shorter diagnostic intervals (Figure 2). For women with a screen-detected breast cancer the time intervals and pattern were similar, although screen-detected patients had significantly shorter 90th percentiles compared to symptomatic patients.

The median treatment intervals for all women ranged from 15 days in Norway and Victoria to 39 days in Manitoba. Women in England, Scotland, Ontario and Manitoba waited more than 28 days (four weeks) for treatment after diagnosis (Table 4). Quantile regression showed smaller statistically significant differences in treatment intervals, with Manitoba and Ontario consistently having longer treatment intervals for the three percentiles (Figure 2). The treatment intervals were similar for screen-detected cancers.

The median total interval for all women from first symptom or date of screening test to start of treatment ranged from 42 days in Sweden and Victoria to 78 days in Ontario (Table 6). In Manitoba and Ontario, 25% of women had a total interval that was 30 days longer compared with Wales (81 days), whereas 25% of women in England, Northern Ireland, Denmark, Victoria and Sweden had a total interval that was between 12 to 19 days shorter compared with Wales (Figure 2 and Additional file 6). In Ontario, 10% of women waited 73 days or more from first presentation until treatment started compared with women in Wales. Including screen-detected breast cancers made the total interval shorter, with less variation between the jurisdictions. There were differences in the time taken from women deciding to seek help getting an appointment with a PCP. Getting an appointment 'within one week' was less often reported by women in Sweden (43%),

For peer review only - http://bmjopen.bmj.com/site/about/guidelines.xhtml

Manitoba (50%), Ontario (58%) and Northern Ireland (64%) compared to women in other jurisdictions (above 70% in the other six jurisdiction with 84% in England and Victoria) (data not shown).

Validity tests

Comparing patient and PCP-reported screening with available screening registry data showed an almost perfect agreement (Kappa> 0.80) (Additional File 7). Comparing the dates between the different data sources showed a high agreement between all data sources (patient, PCP, CTS and register where applicable) for all types of dates (CCC = 0.94 for date of first presentation to primary care, CCC ≥ 0.94 for date of diagnosis and CCC = 0.93 for date of treatment).

Discussion

Main findings

This international survey of breast cancer patients diagnosed between 2013-15 revealed that four out of ten were screen-detected and more than half of women presented with symptoms, with the majority of these diagnosed after a visit to primary care. Three quarters of symptomatic patients had a symptom or sign indicative of breast cancer, and roughly eight in ten were urgently referred. Thus, despite the existence of screening programmes the majority still had their breast cancer diagnosed based on ‘red-flag’ symptoms.

The median time from women first noticing symptoms to start of treatment varied from 42 to 92 days across jurisdictions; this is attributable mainly to differences after first presentation - less variation is seen in the patient interval across jurisdictions.

We found that in some jurisdictions there is a ‘tail’ of women with excessively long waiting times from first noticing symptoms to presentation to the healthcare system.

Strengths and weaknesses

For peer review only - http://bmjopen.bmj.com/site/about/guidelines.xhtml

A major strength is our novel use of a standardised questionnaire survey in several countries to systematically examine routes to diagnosis and treatment. To ensure validity across jurisdictions, we drew on existing instruments and went through an extensive process of cognitive testing, piloting, translation and adaptation [20]. Data were enriched with information from national or state/provincial-level cancer registries via our ICBP M4 Working group (Additional File 8) for screening status, date of diagnosis and tumour stage. We developed algorithms to identify screen-detected cases, place of first presentation and time intervals, which showed good agreement for those jurisdictions where validation was possible. Using registries and undertaking clinical validation of breast cancer patients ensured minimal selection and information biases. It also made it possible to exclude women who previously had breast cancer, thus providing a reasonably homogeneous group of patients.

We included all first-diagnosis breast cancer patients, irrespective of route to diagnosis and recruited at least 200 symptomatic cases per jurisdiction. We developed and implemented validated rules for identifying the screening route, which showed high agreement with screening registries.

There were different classification systems for ethnicity and education across jurisdictions. We excluded these variables from the regression model to avoid introducing information bias in the model, which would have compromised its validity. Although this likely induced some residual confounding, it is unlikely that educational or ethnic differences could have produced the observed variation in time intervals.

We used a set of rules to ensure validity, consistency and to preserve statistical precision [20]. The validity and sensitivity analyses suggested this approach was effective. To minimise misclassification, data interpretation during data entry by the local teams was reduced to an absolute minimum and all apparent data errors were checked against source data by local teams.

For peer review only - http://bmjopen.bmj.com/site/about/guidelines.xhtml

The effect of recall bias was minimised by the triangulation of data sources (eg patient, PCP, CTS and registry data), and by ensuring that the women received the questionnaire within a limited time window after diagnosis.

The overall response rate of 40% was comparable with similar studies among patients and PCPs [30], but varied markedly between jurisdictions, with a response rate of 78% in Denmark and 24% in Norway. This may have resulted in differential selection bias across jurisdictions. However, comparing the participants on a number of variables (e.g. tumour stage, presenting symptom, comorbidity, self-assessed health, smoking status) did not show any meaningful differences. Nevertheless, as recruitment strategies differed, with some women contacted directly by the registries, and others via PCPs (and via nurses in Northern Ireland) assessing eligibility, some selection bias might have been introduced through differing impact of self-selection. This is underlined by the higher-than-expected proportion of stage I tumours amongst our respondents, when compared to an earlier registry-based breast cancer study [3]. Women with early-stage breast cancer are more able to participate in studies of this kind, compared to women with late-stage disease.

The statistical precision of the study was sufficient, as we were able to show clinically significant differences of one week in time intervals, appropriate for clinically relevant differences.

Comparison with other studies

Median patient intervals for breast cancer have varied in previous studies – in Denmark the interval was 14 days in a 2004 study [30], and only 3 days in 2015 (using similar methods) [31], possibly reflecting cancer pathway improvements over that period. Other studies have identified the interval as 13 days (UK) [32], 14 days (New Zealand) [33], 16 days (Germany) [34] and 7 days (Sweden) [35]. Reasons for discrepancies may reflect a difference in the time-points and data sources used to define the patient interval or changes over time in access to primary care.

For peer review only

For peer review only - http://bmjopen.bmj.com/site/about/guidelines.xhtml

Diagnostic intervals vary; a UK study on patients diagnosed in 1999-2000 found a median diagnostic interval of 30 days [36], while other UK studies found diagnostic intervals of 14-27 days [37,38, 39].Lower intervals in more recent studies possibly reflect the introduction of urgent referrals in 2010. A Danish study demonstrated an 18-day interval in 2010 [39], compared to 8 days in our study [39]. Similarly, a study in Manitoba in 2009-10 demonstrated an interval of 35 days compared to 28 days in our study [40]. The primary care interval has had less attention in the literature, although a Manitoban study reported an interval of 15 days, comparable with our finding of 17 days [40]. It also reported a total interval of 78 days, which is comparable to the 76 days found in our study. A recent Swedish study focusing on the time from referral to treatment, which roughly corresponds to a combined diagnostic and treatment interval, reported a median interval of 20 days, compared to 35 days in our study.[35]

Clinical referral for a suspected breast cancer (e.g. lump) is often expedited [37,38]. Thus, the high proportion of women with 'red-flag' symptoms or signs may mean that time intervals for breast cancer diagnosis vary less than other common cancers, as differential diagnosis is easier and investigations and referral pathways more straightforward. Nevertheless, we found important variations in intervals for symptomatic women, possibly reflecting methodological differences – but also temporal and international differences in diagnostic pathways. We would expect these differences to primarily impact on the median primary care interval, 0 days for all jurisdictions except Manitoba, Ontario and Victoria. Note, however, that in these jurisdictions primary care is more often responsible for confirming the breast cancer diagnosis, thus extending the interval. Indeed, a study from Manitoba confirms that PCPs experience significant waiting time when they order a mammogram [40].

Conclusion and implications for clinical practice and healthcare organisation

Despite the existence of well-established screening programmes, the diagnostic route for breast cancer remains critically important, as over half of patients are diagnosed this way. Awareness and recognition of warning signs impact patient intervals [41]; we found, in some jurisdictions, significant numbers of women

For peer review only - http://bmjopen.bmj.com/site/about/guidelines.xhtml

waiting more than 90 days before presenting with symptoms. Targeted awareness interventions could facilitate more timely diagnosis of breast cancer. The variation in waiting times for an appointment in primary care, which might affect perceived access, could also be addressed.

The possible impact of variations in routes to diagnosis, diagnostic/treatment intervals and key outcomes (eg disease-free survival and mortality) cannot be assessed from our study. There is, nevertheless, growing evidence of an association between time intervals, the use of urgent referrals and mortality [10,41- 44]. More research is warranted to explore this relationship.

This study illustrates the need to optimise diagnostic routes for breast cancer internationally. Awareness of international differences in key time intervals is an important step in optimising pathways. Ideally, routine and standardised collection of time interval and route to diagnostic information will become the norm internationally – this will greatly assist in optimisation of breast cancer diagnostic pathways.

For peer review only

For peer review only - http://bmjopen.bmj.com/site/about/guidelines.xhtml

List of abbreviations

ICBP M4 – International Cancer Benchmarking Partnership Module 4

PCP – primary care physician

TNM – tumour, node, metastasis

CTS – cancer treatment specialist

IARC – International Agency for Research on Cancer

Declarations

Ethics approval and consent to participate

For each local data collection, there were specific procedures and approvals. The approvals also included anonymised data transfer to University College London and Aarhus University (Additional File 8). Approvals were received from the following institutions: Cancer Council Victoria Human Research Ethics Committee [HREC 1125]; Health Research Ethics Board, University of Manitoba [HS15227 (H2012:105)]; Research Resource Ethics Committee, CancerCare Manitoba [RRIC#28-2012]; University of Toronto Research Ethics Board [27881]; The Danish Data Protection Agency [2013-41-2030]; Swedish Ethics Review Board, Uppsala [2013/306]; Norway Regional committees for medical and health research ethics [2013/136/REK nord]; England, Wales and Scotland, NRES Committee East Midlands – Derby 2, local R&D for each health board [11/EM/0420]; Northern Ireland ORECNI Ethical approval, local governance for each health Trust [11/EM/0420].

Consent for publication

Not Applicable

Availability of data and material

The data that support the findings of this study are available from the named authors from each ICBP jurisdiction, but restrictions apply to the availability of these data and so are not publicly available. Data are

For peer review only

however available from the authors upon reasonable request and with permission of the ICBP Programme Board. Please contact the ICBP Programme Management team, based at Cancer Research UK, with any queries (icbp@cancer.org.uk).

Competing interests

The authors declare that they have no competing interests.

Funding

Funding for ICBP Module 4 was provided by: CancerCare Manitoba; Cancer Care Ontario; Canadian Partnership Against Cancer (CPAC); Cancer Council Victoria; Cancer Research Wales; Cancer Research UK; Danish Cancer Society; Danish Health and Medicines Authority; European Palliative Care Research Centre (PRC), Norwegian University of Science and Technology (NTNU); Guidelines Audit and Implementation Network (GAIN); Macmillan Cancer Support; National Cancer Action Team; NHS England; Northern Ireland Cancer Registry, funded by the Public Health Agency NI; Norwegian Directorate of Health; Research Centre for Cancer Diagnosis in Primary Care (CaP), Aarhus University, Denmark; Scottish Government; Swedish Association of Local Authorities and Regions; University College London and NIHR Biomedical Research Centre at University College London NHS Foundation Trust; University of Edinburgh (R42856); Victorian Department of Health and Human Services; Welsh Government.

The funding bodies had no influence on the design of the study and collection, analysis, and interpretation of data, in writing the manuscript or whether to publish the results.

Authors' contributions

- **Usha Menon** - planned the study design, data collection, carried out the analyses and wrote the draft manuscript. Also responsible for local data collection (alongside the Working Group), management and interpretation, and have participated in writing and approving the final manuscript version.

For peer review only - http://bmjopen.bmj.com/site/about/guidelines.xhtml

- **David Weller** - planned the study design, data collection, carried out the analyses and wrote the draft manuscript. Also responsible for local data collection (alongside the Working Group), management and interpretation, and have participated in writing and approving the final manuscript version.
- **Alina Zalounina Falborg** - planned the study design, data collection, carried out the analyses and wrote the draft manuscript
- **Henry Jensen** - planned the study design, data collection, carried out the analyses and wrote the draft manuscript. Also responsible for local data collection (alongside the Working Group), management and interpretation, and have participated in writing and approving the final manuscript version.
- **Peter Vedsted**- planned the study design, data collection, carried out the analyses and wrote the draft manuscript. Also responsible for local data collection (alongside the Working Group), management and interpretation, and have participated in writing and approving the final manuscript version.
- **Jatinderpal Kalsi** - Responsible for local data collection (alongside the ICBP Module 4Working Group), management and interpretation, and have participated in writing and approving the final manuscript version.
- **David H Brewster** - Responsible for local data collection (alongside the ICBP Module 4Working Group), management and interpretation, and have participated in writing and approving the final manuscript version.
- **Yulan Lin** - Responsible for local data collection (alongside the ICBP Module 4Working Group), management and interpretation, and have participated in writing and approving the final manuscript version.
- **Anna Gavin** - Responsible for local data collection (alongside the ICBP Module 4Working Group), management and interpretation, and have participated in writing and approving the final manuscript version.
- **Andriana Barisic -** Responsible for local data collection (alongside the ICBP Module 4Working Group), management and interpretation, and have participated in writing and approving the final manuscript version.
- **Eva Grunfeld** - Responsible for local data collection (alongside the ICBP Module 4Working Group), management and interpretation, and have participated in writing and approving the final manuscript version.
- **Mats Lambe** - Responsible for local data collection (alongside the ICBP Module 4Working Group), management and interpretation, and have participated in writing and approving the final manuscript version.
- **Martin Malmberg** - Responsible for local data collection (alongside the ICBP Module 4Working Group), management and interpretation, and have participated in writing and approving the final manuscript version.

For peer review only - http://bmjopen.bmj.com/site/about/guidelines.xhtml

- **Donna Turner** - Responsible for local data collection (alongside the ICBP Module 4Working Group), management and interpretation, and have participated in writing and approving the final manuscript version.
- **Elizabeth Harland** - Responsible for local data collection (alongside the ICBP Module 4Working Group), management and interpretation, and have participated in writing and approving the final manuscript version.
- **Breann Hawryluk** - Responsible for local data collection (alongside the ICBP Module 4Working Group), management and interpretation, and have participated in writing and approving the final manuscript version.
- **Rebecca-Jane Law** - Responsible for local data collection (alongside the ICBP Module 4Working Group), management and interpretation, and have participated in writing and approving the final manuscript version.
- **Richard D Neal** - Responsible for local data collection (alongside the ICBP Module 4Working Group), management and interpretation, and have participated in writing and approving the final manuscript version.
- **Victoria White** - Responsible for local data collection (alongside the ICBP Module 4Working Group), management and interpretation, and have participated in writing and approving the final manuscript version.
- **Rebecca J Bergin** - Responsible for local data collection (alongside the ICBP Module 4Working Group), management and interpretation, and have participated in writing and approving the final manuscript version.
- **Samantha Harrison** - Responsible for local data collection (alongside the ICBP Module 4Working Group), management and interpretation, and have participated in writing and approving the final manuscript version.
- **ICBP Module 4 Working Group** – Responsible for local data collection

Acknowledgements

We thank our colleagues and participating patients, primary care physicians and cancer treatment specialists in all participating ICBP jurisdictions. Catherine Foot, Martine Bomb, Brad Groves, Irene Reguilon, Charlotte Lynch and Samantha Harrison of Cancer Research UK for managing the programme. John Butler, Royal Marsden NHS Foundation Trust and ICBP Clinical Lead, for his advice. The ICBP M4 academic reference group for providing independent peer review and advice for the study protocol and analysis plan development. We would like to thank staff at the Information Services Division of NHS National Services Scotland for identifying potential study participants in Scotland, and mailing patient questionnaires to primary care physicians. The Danish Breast Cancer Group (DBCG) for providing secondary care (specialist) data to this study.

For peer review only - http://bmjopen.bmj.com/site/about/guidelines.xhtml

The ICBP Module 4 working Group:

- Alina Zalounina Falborg, Statistician, Research Unit for General Practice, Department of Public Health, Aarhus University, Bartholins Allé 2, 8000 Aarhus C, Denmark;
- Andriana Barisic, Research Associate, Department of Prevention and Cancer Control, Cancer Care Ontario, 620 University Avenue, Toronto, Ontario, M5G 2L7, Canada;
- Anna Gavin, Director, Northern Ireland Cancer Registry, Centre for Public Health, Queen's University Belfast, Mulhouse Building, Mulhouse Road, Belfast, BT12 6DP, United Kingdom;
- Anne Kari Knudsen, Administrative leader, Department of Cancer Research and Molecular Medicine, Norwegian University of Science and Technology, 7489 Trondheim, Norway;
- Breann Hawryluk, Project Planning Coordinator, Department of Patient Navigation, Cancer Care Manitoba, 675 McDermot Street, Winnipeg, Manitoba, Canada;
- Chantelle Anandan, Research Fellow, Centre for Population Health Sciences, University of Edinburgh, Doorway 1, Medical Quad Teviot Place, Edinburgh, EH8 9DX, United Kingdom;
- Conan Donnelly, Research Fellow, Centre for Public Health, Queen's University Belfast, Mulhouse Building, Mulhouse Road, Belfast, BT12 6DP, United Kingdom;
- David H Brewster, Scottish Cancer Registry, Information Services Division, NHS National Services Scotland, Gyle Square, 1 South Gyle Crescent, Edinburgh, EH12 9EB, United Kingdom;
- David Weller, James Mackenzie Professor of General Practice, Centre for Population Health Sciences, University of Edinburgh, Doorway 1, Medical Quad Teviot Place, Edinburgh, EH8 9DX, United Kingdom;
- Donna Turner, Epidemiologist/Provincial Director, Population Oncology, Cancer Care Manitoba, 675 McDermot Street, Winnipeg, Manitoba, Canada;
- Elizabeth Harland, Project Coordinator, Department of Epidemiology and Cancer Registry, CancerCare Manitoba, 675 McDermot Street, Winnipeg, Manitoba;
- Eva Grunfeld, Director, Knowledge Translation Research Network Health Services Research Program, Ontario Institute for Cancer Research; Professor and Vice Chair Research, Department of Family and Community Medicine, University of Toronto, 500 University Avenue, Toronto, Ontario, M5G 1V7, Canada;
- Evangelia Ourania Fourkala, Research Associate, Gynaecological Cancer Research Centre, Women's Cancer, Institute for Women's Health, University College London, United Kingdom;
- Henry Jensen, Research fellow, Research Unit for General Practice, Department of Public Health, Aarhus University, Bartholins Allé 2, 8000 Aarhus C, Denmark;
- Jackie Boylan, Research Fellow, Centre for Public Health, Queen's University Belfast, Mulhouse Building, Mulhouse Road, Belfast, BT12 6DP, United Kingdom;
- Jacqueline Kelly, Tumour Verification Officer, Northern Ireland Cancer Registry, Centre for Public Health, Queen's University Belfast, Mulhouse Building, Mulhouse Road, Belfast, BT12 6DP, United Kingdom;
- Kerry Moore, Research Fellow, Centre for Public Health, Queen's University Belfast, Mulhouse Building, Mulhouse Road, Belfast, BT12 6DP, United Kingdom;
- Maria Rejmyr Davis, Head, Southern Sweden Regional Cancer Center, Medicon Village, Scheelevägen 8, building 404, 223 81 Lund, Sweden;
- Martin Malmberg, MD PhD, Senior Consultant, Department of Oncology, Lund University Hospital, SE-221 85 Lund, Sweden;
- Mats Lambe, Professor of Medical Epidemiology, Regional Cancer Center Uppsala and Department of Medical Epidemiology and Biostatics, Karolinska Institutet, SE-171 77 Stockholm, Sweden;

For peer review only - http://bmjopen.bmj.com/site/about/guidelines.xhtml

- Oliver Bucher, Epidemiologist, Department of Epidemiology and Cancer Registry, CancerCare Manitoba, 675 McDermot Street, Winnipeg, Manitoba;
- Peter Vedsted, Professor, Research Unit for General Practice, Department of Public Health, Aarhus University, Bartholins Allé 2, 8000 Aarhus C, Denmark;
- Rebecca Bergin, Senior Research Officer/ PhD Candidate, Centre for Behavioural Research in Cancer, 615 St Kilda Rd, Melbourne, Victoria, 3004, Australia;
- Rebecca-Jane Law, Research Project Support Officer, North Wales Centre for Primary Care Research, Bangor University, Gwenfro Units 4-8, Wrexham Technology Park, Wrexham, LL13 7YP, United Kingdom;
- Richard D Neal, Professor of Primary Care Oncology, Academic Unit of Primary Care, Leeds Institute of Health Sciences, University of Leeds, Leeds LS2 9NL, United Kingdom;
- Sigrun Saur Almberg, Researcher, Department of Cancer Research and Molecular Medicine, Faculty of Medicine, Norwegian University of Science and Technology (NTNU), N-7491 Trondheim, Norway;
- Therese Kearney, Research fellow, Northern Ireland Cancer Registry, Centre for Public Health, Queen's University Belfast, Mulhouse Building, Mulhouse Road, Belfast, BT12 6DP, United Kingdom;
- Jatinderpal Kalsi, Project Manager, Gynaecological Cancer Research Centre, Women's Cancer, Institute for Women's Health, University College London, United Kingdom;
- Victoria Cairnduff, Statistician, Northern Ireland Cancer Registry, Centre for Public Health, Queen's University Belfast, Mulhouse Building, Mulhouse Road, Belfast, BT12 6DP, United Kingdom;
- Victoria Hammersley, Researcher, Centre for Population Health Sciences, University of Edinburgh, Doorway 1, Medical Quad Teviot Place, Edinburgh, EH8 9DX, United Kingdom;
- Victoria White, Deputy Director, Centre for Behavioral Research in Cancer, Cancer Council Victoria, 615 St Kilda Road, Melbourne, Victoria, 3004, Australia;
- Usha Menon, Professor of Gynaecological Oncology and Head, Gynaecological Cancer Research Centre, Women's Cancer, Institute for Women's Health, University College London, United Kingdom;
- Yulan Lin, Postdoc, Department of Cancer Research and Molecular Medicine, Faculty of Medicine, Norwegian University of Science and Technology (NTNU), N-7491 Trondheim, Norway

ICBP Module 4 Academic Reference Group:

- Prof Jan Willem Coebergh, Professor of Cancer Surveillance, Department of Public Health, Erasmus Universiteit Rotterdam, Rotterdam, the Netherlands;
- Jon Emery, Professor of Primary Care Cancer Research, University of Melbourne and Clinical Professor of General Practice, University of Western Australia, Australia;
- Dr Stefan Bergström, Senior consultant oncologist, Department of Oncology, Gävle, Sweden;
- Dr Monique E van Leerdam, Erasmus MC University Medical Centre, the Netherlands;
- Prof Marie-Louise Essink-Bot, Academic Medical Centre, Amsterdam University, the Netherlands;
- Prof Una MacLeod, Senior Lecturer in General Practice and Primary Care, Hull-York Medical School, United Kingdom.

For peer review only

For peer review only

For peer review only - http://bmjopen.bmj.com/site/about/guidelines.xhtml

References

1. Global Burden of Disease Cancer C, Fitzmaurice C, Dicker D, Pain A, Hamavid H, Moradi-Lakeh M, MacIntyre MF, Allen C, Hansen G, Woodbrook R, et al: The Global Burden of Cancer 2013. JAMA Oncol 2015, 1:505-527.

2. Allemani C, Matsuda T, Di Carlo V, Harewood R, Matz M, Nikšić M, Bonaventure A, Valkov M, Johnson CJ, Estève J, et al: Global surveillance of trends in cancer survival 2000-14 (CONCORD-3): analysis of individual records for 37 513 025 patients diagnosed with one of 18 cancers from 322 population-based registries in 71 countries. Lancet 2018, S0140-6736(17)33326-3. [Epub ahead of print].

3. Walters S, Maringe C, Butler J, Rachet B, Barrett-Lee P, Bergh J, Boyages J, Christiansen P, Lee M, Warnberg F, et al: Breast cancer survival and stage at diagnosis in Australia, Canada, Denmark, Norway, Sweden and the UK, 2000-2007: a population-based study. Br J Cancer 2013, 108:1195-1208.

4. Coleman MP, Forman D, Bryant H, Butler J, Rachet B, Maringe C, Nur U, Tracey E, Coory M, Hatcher J, et al: Cancer survival in Australia, Canada, Denmark, Norway, Sweden, and the UK, 1995-2007 (the International Cancer Benchmarking Partnership): an analysis of population-based cancer registry data. Lancet 2011, 377:127-138.

5. Niksic M, Rachet B, Duffy SW, Quaresma M, Moller H, Forbes LJ: Is cancer survival associated with cancer symptom awareness and barriers to seeking medical help in England? An ecological study. Br J Cancer 2016, 115:876-886.

6. Yun YH, Kim YA, Min YH, Park S, Won YJ, Kim DY, Choi IJ, Kim YW, Park SJ, Kim JH, et al: The influence of hospital volume and surgical treatment delay on long-term survival after cancer surgery. Ann Oncol 2012, 23:2731-2737.

7. Smith EC, Ziogas A, Anton-Culver H. Delay in surgical treatment and survival after breast cancer diagnosis in young women by race/ethnicity. JAMA Surg. 2013;148:516-23.

8. Torring ML, Frydenberg M, Hamilton W, Hansen RP, Lautrup MD, Vedsted P: Diagnostic interval and mortality in colorectal cancer: U-shaped association demonstrated for three different datasets. J Clin Epidemiol 2012, 65:669-678.

9. Torring ML, Frydenberg M, Hansen RP, Olesen F, Vedsted P: Evidence of increasing mortality with longer diagnostic intervals for five common cancers: a cohort study in primary care. Eur J Cancer 2013, 49:2187-2198.

10. Neal RD, Tharmanathan P, France B, Din NU, Cotton S, Fallon-Ferguson J, Hamilton W, Hendry A, Hendry M, Lewis R, et al: Is increased time to diagnosis and treatment in symptomatic cancer associated with poorer outcomes? Systematic review. Br J Cancer 2015, 112 Suppl 1:S92-107.

11. Forbes LJ, Simon AE, Warburton F, Boniface D, Brain KE, Dessaix A, Donnelly C, Haynes K, Hvidberg L, Lagerlund M, et al: Differences in cancer awareness and beliefs between Australia, Canada, Denmark, Norway, Sweden and the UK (the International Cancer Benchmarking Partnership): do they contribute to differences in cancer survival? Br J Cancer 2013, 108:292-300.

12. Koo MM, von Wagner C, Abel GA, McPhail S, Rubin GP, Lyratzopoulos G: Typical and atypical presenting symptoms of breast cancer and their associations with diagnostic intervals: Evidence from a national audit of cancer diagnosis. Cancer Epidemiol 2017, 48:140-146.

For peer review only

For peer review only - http://bmjopen.bmj.com/site/about/guidelines.xhtml

13. Brown S, Castelli M, Hunter DJ, Erskine J, Vedsted P, Foot C, Rubin G: How might healthcare systems influence speed of cancer diagnosis: a narrative review. Soc Sci Med 2014, 116:56-63.

14. Lyratzopoulos G, Vedsted P, Singh H: Understanding missed opportunities for more timely diagnosis of cancer in symptomatic patients after presentation. Br J Cancer 2015, 112 Suppl 1:S84-91.

15. Rubin G, Berendsen A, Crawford SM, Dommett R, Earle C, Emery J, Fahey T, Grassi L, Grunfeld E, Gupta S, et al: The expanding role of primary care in cancer control. Lancet Oncol 2015, 16:1231-1272.

16. Rose PW, Rubin G, Perera-Salazar R, Almberg SS, Barisic A, Dawes M, Grunfeld E, Hart N, Neal RD, Pirotta M, et al: Explaining variation in cancer survival between 11 jurisdictions in the International Cancer Benchmarking Partnership: a primary care vignette survey. BMJ Open 2015, 5:e007212.

17. National Institute for Health and Care Excellence. Suspected cancer: recognition and referral; NICE Guideline [NG12]. National Institute for Health Care Excellence, 2015. Available at: https://www.nice.org.uk/guidance/ng12 [accessed 05 October 2017]

18. Murchie P, Campbell NC, Delaney EK, Dinant GJ, Hannaford PC, Johansson L, Lee AJ, Rollano P, Spigt M: Comparing diagnostic delay in cancer: a cross-sectional study in three European countries with primary care-led health care systems. Fam Pract 2012, 29:69-78.

19. Butler J, Foot C, Bomb M, Hiom S, Coleman M, Bryant H, Vedsted P, Hanson J, Richards M, Group IW: The International Cancer Benchmarking Partnership: an international collaboration to inform cancer policy in Australia, Canada, Denmark, Norway, Sweden and the United Kingdom. Health Policy 2013, 112:148-155.

20. Weller D, Vedsted P, Anandan C, Zalounina A, Fourkala EO, Desai R, Liston W, Jensen H, Barisic A, Gavin A, et al: An investigation of routes to cancer diagnosis in 10 international jurisdictions, as part of the International Cancer Benchmarking Partnership: survey development and implementation. BMJ Open 2016, 6:e009641.

21. European Network of Cancer Registries. Standards and guidelines for cancer registration in Europe: the ENCR recommendations. Edited by Tyczynski JE, Démaret E, Parkin DM. (IARC technical publication; no. 40), Lyon, 2003.

22. TNM classification of malignant tumours. Eds. L.H. Sobin, M.K. Gospodarowicz and Ch. Wittekind. — 7th ed. UICC International Union Against Cancer. ISBN 978-1-4443-3241-4

23. Weller D, Vedsted P, Rubin G, Walter FM, Emery J, Scott S, Campbell C, Andersen RS, Hamilton W, Olesen F, et al: The Aarhus statement: improving design and reporting of studies on early cancer diagnosis. Br J Cancer 2012, 106:1262-1267.

24. Macefield RC, Jacobs M, Korfage IJ, Nicklin J, Whistance RN, Brookes ST, et al. Developing core outcomes sets: methods for identifying and including patient-reported outcomes (PROs). Trials. 2014 Feb 5;15:49.

25. Danish Cancer Society. Suspicion of breast cancer criteria for referral for general practice. Danish Cancer Society, 2016. Available at: https://www.sst.dk/da/udgivelser/2016/~/media/4864545DA763400E9FD988FA4DA74EE5.ashx accessed 05 October 2017

For peer review only - http://bmjopen.bmj.com/site/about/guidelines.xhtml

26. Koenker R, Bassett G. Regression quantiles. *Econometrica* 1978; 46:33-50

27. Machado JAF, Santos Silva JMC. Quantiles for Counts. J Amer Statistical Assoc 2005, 100(472): 1226-1237.

28. Miranda A. QCOUNT: Stata program to fit quantile regression models for count data. Boston: Boston College Department of Economics; 2006

29. Lin LI. A concordance correlation coefficient to evaluate reproducibility. Biometrics 1989;45((1) March):255–68

30. Hansen RP, Vedsted P, Sokolowski I, Sondergaard J, Olesen F: Time intervals from first symptom to treatment of cancer: a cohort study of 2,212 newly diagnosed cancer patients. BMC Health Serv Res 2011, 11:284.

31. Olesen F, Hansen RP, Vedsted P: Delay in diagnosis: the experience in Denmark. Br J Cancer 2009, 101 Suppl 2:S5-8.

32. Nosarti C, Crayford T, Roberts JV, Elias E, McKenzie K, David AS: Delay in presentation of symptomatic referrals to a breast clinic: patient and system factors. Br J Cancer 2000, 82:742-748.

33. Meechan G, Collins J, Petrie K: Delay in seeking medical care for self-detected breast symptoms in New Zealand women. N Z Med J 2002, 115:U257.

34. Arndt V, Sturmer T, Stegmaier C, Ziegler H, Dhom G, Brenner H: Patient delay and stage of diagnosis among breast cancer patients in Germany -- a population based study. Br J Cancer 2002, 86:1034-1040.

35. Thulesius HO, Lindgren AC, Olsson HL, Hakansson A: Diagnosis and prognosis of breast and ovarian cancer--a population-based study of 234 women. Acta Oncol 2004, 43:175-181.

36. Allgar VL, Neal RD: Delays in the diagnosis of six cancers: analysis of data from the National Survey of NHS Patients: Cancer. Br J Cancer 2005, 92:1959-1970.

37. Din NU, Ukoumunne OC, Rubin G, Hamilton W, Carter B, Stapley S, Neal RD: Age and Gender Variations in Cancer Diagnostic Intervals in 15 Cancers: Analysis of Data from the UK Clinical Practice Research Datalink. PLoS One 2015, 10:e0127717.

38. Redaniel MT, Martin RM, Ridd MJ, Wade J, Jeffreys M: Diagnostic intervals and its association with breast, prostate, lung and colorectal cancer survival in England: historical cohort study using the Clinical Practice Research Datalink. PLoS One 2015, 10:e0126608.

39. Jensen H, Torring ML, Olesen F, Overgaard J, Fenger-Gron M, Vedsted P: Diagnostic intervals before and after implementation of cancer patient pathways - a GP survey and registry based comparison of three cohorts of cancer patients. BMC Cancer 2015, 15:308.

40. Nashed M, Carpenter-Kellett T, Lambert P, Musto G, Turner D, Cooke A: Wait Time from Suspicion to Surgery for Breast Cancer in Manitoba. Cureus 2016, 8:e680.

41. Quaife SL, Forbes LJ, Ramirez AJ, Brain KE, Donnelly C, Simon AE, Wardle J: Recognition of cancer warning signs and anticipated delay in help-seeking in a population sample of adults in the UK. Br J Cancer 2014, 110:12-18.

For peer review only

42. Richards MA, Westcombe AM, Love SB, Littlejohns P, Ramirez AJ. Influence of delay on survival in patients with breast cancer: a systematic review. Lancet. 1999 Apr 3;353(9159):1119-26.

43. Moller H, Gildea C, Meechan D, Rubin G, Round T, Vedsted P. Use of the English urgent referral pathway for suspected cancer and mortality in patients with cancer: cohort study. BMJ. 2015 Oct 13;351:h5102. doi: 10.1136/bmj.h5102.

44. Murchie P, Raja EA, Lee AJ, Brewster DH, Campbell NC, Gray NM, Ritchie LD, Robertson R, Samuel L: Effect of longer health service provider delays on stage at diagnosis and mortality in symptomatic breast cancer. Breast 2015, 24:248-255.

For peer review only

For peer review only - http://bmjopen.bmj.com/site/about/guidelines.xhtml

For peer review only

For peer review only - http://bmjopen.bmj.com/site/about/guidelines.xhtml

Table 1. *The characteristics of the 3,471 women (40+) with a first diagnosis of breast cancer (% if nothing else stated)*

	**Wales (N=232)**	**England (N=363)**	**Scotland (N=371)**	**N Ireland (N=331)**	**Denmark (N=369)**	**Ontario (N=403)**	**Norway (N=372)**	**Manitoba (N=368)**	**Victoria (N=370)**	**Sweden (N=292)**	**Total (N=3471)**
**Age** years											
Median (IQI)	63 (53,70)	63 (53,70)	63 (54,70)	60 (51,70)	64 (55,71)	63 (55,69)	61 (52, 69)	63 (55,72)	58 (50,67)	63 (54,71)	62 (53,70)
**Age** years (Symptomatic women)	(N=115)	(N=207)	(N=208)	(N=242)	(N=231)	(N=201)	(N=235)	(N=205)	(N=198)	(N=120)	(N=1962)
Median (IQI)	62 (49,76)	62 (50,75)	62 (52,75)	60 (49,72)	68 (54,77)	61 (53,69)	61 (50, 73)	62 (50,75)	56 (48,68)	68 (54,77)	62 (50,74)
**Age** years (Screen-detected women	(N=116)	(N=155)	(N=162)	(N=86)	(N=134)	(N=198)	(N=132)	(N=162)	(N=172)	(N=171)	(N=1488)
Median (IQI)	63 (58,68)	64 (58,68)	63 (58,67)	60 (54,67)	62 (56,66)	65 (59,69)	62 (57,67)	65 (59,70)	61 (53,67)	61 (54,69)	63 (57,68)
**Health State**											
Good	81.0	81.0	79.3	77.7	78.1	90.3	74.7	85.6	88.4	80.8	81.8
Fair	14.7	13.0	13.7	14.2	14.6	8.2	17.5	9.2	9.5	12.3	12.6
Poor	3.5	4.7	5.1	5.4	3.5	1	6.2	2.7	1.6	3.8	3.7
Missing	0.9	1.3	1.9	2.7	3.8	0.5	1.6	2.5	0.5	3.1	1.9
**Comorbidity**[1]											
No	74.1	75.1	69.0	78.6	62.9	61.3	73.9	70.4	76.5	80.5	71.8
Medium	24.6	23.1	29.4	19.9	34.1	25.1	23.7	27.7	23.2	16.1	25.0
High	0.9	0.8	1.1	0.9	0.8	0.0	1.1	0.5	0.0	0.3	0.6
Missing	0.4	0.8	0.5	0.6	2.2	13.6	1.3	1.4	0.3	3.1	2.6
**Education**											
Low	71.6	78.2	69.0	77.0	71.8	64.3	63.7	74.7	70.0	68.8	70.8
High	22.4	14.9	19.7	16.3	14.6	33.5	31.5	19.8	28.9	28.8	23.1
Missing	6.0	6.9	11.3	6.7	13.6	2.2	4.8	5.4	1.1	2.4	6.1
**Ethnicity**											
White	97.9	97.8	98.9	99.1	94.3	89.9	98.7	85.6	97.3	96.2	95.4
Asian	0.4	1.1	0.0	0.0	1.1	5.7	0.8	7.6	1.9	1.0	2.1
Black	0.4	0.8	0.3	0.0	0.0	2.0	0.0	0.5	0.0	0.0	0.4
Other	0.0	0.0	0.0	0.0	0.0	1.2	0.0	6.0	0.0	0.0	0.8
Missing	1.3	0.3	0.8	0.9	4.6	1.2	0.5	0.3	0.8	2.8	1.3
**Smoking**											
Currently	7.3	7.2	8.4	11.8	15.2	4.0	15.1	7.9	5.7	19.6	9.2
In the past	35.3	38.0	41.2	33.2	41.7	43.9	40.9	44.6	36.7	41.1	39.9
Never	56.5	54.5	49.3	53.5	40.9	51.6	43.5	46.2	57.3	46.9	49.8
Missing	0.9	0.3	1.1	1.5	2.2	0.5	0.5	1.3	0.3	2.4	1.1
**Tumour stage – TNM** (all women)											
0	1.7	0.6	0.0	0.0	0.3	0.3	0.0	0.0	0.3	0.0	0.3
I	50.4	44.9	47.4	45.3	45.0	51.6	11.3	50.8	52.7	62.0	45.7
II	37.9	36.4	35.6	35.1	33.1	38.0	4.6	34.5	34.6	31.5	31.9
III	5.6	8.3	7.8	13.6	9.5	8.2	1.9	10.3	10.8	4.1	8.1
IV	0.9	3.0	4.6	2.7	2.4	1.0	0.8	1.6	1.1	0.7	1.9
Missing	3.5	6.9	4.5	3.3	9.8	1.0	81.5	2.7	0.5	1.7	12.1

For peer review only - http://bmjopen.bmj.com/site/about/guidelines.xhtml

	Wales (N=232)	England (N=363)	Scotland (N=371)	N Ireland (N=331)	Denmark (N=369)	Ontario (N=403)	Norway (N=372)	Manitoba (N=368)	Victoria (N=370)	Sweden (N=292)	Total (N=3471)
**Tumour stage – TNM** (Symptomatic women)	(N=115)	(N=207)	(N=208)	(N=242)	(N=231)	(N=201)	(N=235)	(N=205)	(N=198)	(N=120)	(N=1966)
0	1.7	1.0	0.0	0.0	0.0	0.0	0.0	0.0	0.5	0.0	0.3
I	33.0	34.8	32.7	37.2	36.4	32.8	8.1	32.7	37.9	40.8	32.1
II	49.6	41.6	45.7	39.3	35.1	50.0	5.5	48.3	44.4	50.8	39.5
III	8.7	10.6	9.6	17.4	11.7	14.0	2.6	13.2	15.2	3.3	11.0
IV	1.7	4.4	6.7	2.9	3.9	2.0	0.9	2.4	1.0	1.7	2.9
Missing	5.2	7.7	5.3	3.3	13.0	0.5	83.0	3.4	1.0	3.3	14.3
**Tumour stage – TNM** (Screen-detected women)	(N=116)	(N=155)	(N=162)	(N=86)	(N=134)	(N=198)	(N=132)	(N=162)	(N=172)	(N=171)	(N=1484)
0	1.7	0.0	0.0	0.0	0.8	0.5	0.0	0.0	0.0	0.0	0.3
I	68.1	58.1	66.1	67.4	59.7	69.2	16.7	74.1	69.8	77.2	63.5
II	25.9	29.7	22.8	23.3	29.1	26.3	2.3	17.3	23.3	18.1	21.9
III	2.6	5.2	5.6	3.5	6.0	2.5	0.8	6.8	5.8	4.1	4.4
IV	0.0	1.3	1.9	2.3	0.0	0.0	0.0	0.6	1.2	0.0	0.7
Missing	1.7	5.8	3.7	3.5	4.5	1.5	80.3	1.2	0.0	0.6	9.3

[1] Comorbidity coded as none=no reported, medium=1-2 reported and high=3+ reported

Abbreviations: IQI=inter-quartile interval

Norwegian data on tumour stage were incomplete, resulting in a high number of missing stage data.

For peer review only

For peer review only - http://bmjopen.bmj.com/site/about/guidelines.xhtml

Table 2. Proportion of screen-detected or symptomatic patients (%), and place of initial symptomatic presentation across jurisdictions

	Wales (N=232)	England (N=363)	Scotland (N=371)	N Ireland[1] (N=331)	Denmark (N=369)	Ontario (N=403)	Norway (N=373)	Manitoba (N=368)	Victoria (N=370)	Sweden (N=292)	Total (N=3471)
**Symptomatic**	**49.6**	**57.0**	**56.1**	**73.1**	**62.6**	**49.9**	**63.2**	**55.7**	**53.5**	**41.1**	**56.5**
Visit PCP, Visit PCP and then A&E[2]	*91.3*	*93.7*	*92.3*	*88.0*	*84.4*	*69.2*	*81.3*	*88.3*	*90.9*	*75.0*	*85.6*
A&E directly[2]	*1.7*	*1.0*	*0.5*	*0.4*	*1.3*	*2.5*	*0.9*	*1.0*	*0.5*	*0.8*	*1.0*
Investigation for another problem[2]	*0.9*	*1.0*	*4.8*	*1.2*	*6.1*	*7.5*	*4.7*	*2.4*	*1.5*	*10.0*	*3.9*
Other[2]	*6.1*	*4.3*	*2.4*	*10.3*	*8.2*	*20.9*	*13.2*	*8.3*	*7.1*	*14.2*	*9.5*
**Screening**	**50.0**	**42.7**	**43.7**	**26.0**	**36.3**	**49.1**	**35.5**	**44.0**	**46.5**	**58.6**	**42.9**
**Other**	**0.4**	**0.3**	**0.3**	**0.9**	**1.1**	**1.0**	**1.2**	**0.3**	**0.0**	**0.3**	**0.6**

[1] In N Ireland the proportion of screen detected breast cancers was lower as many of these women were excluded from the eligible group in the start of the inclusion

[2] Percentage of the symptomatic route

For peer review only

For peer review only - http://bmjopen.bmj.com/site/about/guidelines.xhtml

Table 3. *The first symptom(s) reported by women[1] and the first presenting symptom(s) reported by PCPs (%)*

	**Wales**	**England**	**Scotland**	**N Ireland[1]**	**Denmark**	**Ontario**	**Norway**	**Manitoba**	**Victoria**	**Sweden[2]**	**Total**
First symptom *(reported by women)*	*(N=115)*	*(N=207)*	*(N=208)*	*(N=242)*	*(N=231)*	*(N=201)*	*(N=235)*	*(N=205)*	*(N=198)*	*(N=120)*	*(N=1962)*
Lump/swelling/thickening of breast	76	70	75	62	63	63	55	60	71	73	66
Change in the nipple	9	15	11	14	11	10	13	6	6	14	11
Fatigue	16	10	9	12	5	17	11	12	14	9	11
Change in the size, contour	12	13	10	10	5	16	8	10	15	11	11
Pain/tenderness in the breast	6	5	4	14	3	5	7	9	15	6	8
Flattening/indentation in the breast	5	5	0.5	4	4	0	2	2	0	0.8	2
Other	21	23	20	17	10	13	18	19	16	12	17
No symptoms	8	2	4	8	11	10	9	17	5	10	8
Missing	0	0.4	4	6	10	2	13	4	1	0	5
Presenting symptom *(reported by PCP)*	*(N=97)*	*(N=169)*	*(N=160)*	*(N=0)*	*(N=153)*	*(N=74)*	*(N=109)*	*(N=123)*	*(N=128)*	*(N=0)*	*(N=1013)*
Lump/swelling/thickening of breast	73	64	64	n/a	67	34	55	46	64	n/a	60
Change in the nipple	2	8	5	n/a	5	7	5	0.8	6	n/a	5
Fatigue	0	0	0	n/a	0	0	0.9	0.8	0	n/a	0.2
Change in the size, contour	4	4	0	n/a	6	0	0.9	3	5	n/a	3
Pain/tenderness in the breast	10	6	4	n/a	7	7	4	10	13	n/a	7
Flattening/indentation in the breast	2	2	1	n/a	0	3	2	3	0	n/a	2
Other	13	12	9	n/a	7	24	13	13	2	n/a	11
No symptoms	1	0.6	0.6	n/a	0.7	11	0	12	2	n/a	3
Missing	8	11	17	n/a	18	18	21	24	20	n/a	17
Cancer-specificity of presenting symptom[3]											
Cancer-specific symptom	88	85	79	n/a	78	69	70	59	78	n/a	77
Non-specific symptom	3	3	4	n/a	3	3	9	4	0	n/a	4
No symptoms / Missing	9	12	18	n/a	18	28	21	37	22	n/a	20

[1] These figures represent only women who had a symptom-based cancer diagnosis. More than one symptom could be stated (sum over 100%)

[2] Sweden and Northern Ireland did not include PCP questionnaire data

[3] Based on the PCP reported symptom/sign and categorised by two researchers

For peer review only

Table 4. *Time intervals (days) for each jurisdiction depicted as median, 75th and 90th percentiles.*

		Wales	England	Scotland	N Ireland	Denmark	Ontario	Norway	Manitoba	Victoria	Sweden[1]
Patient Interval (Symptomatic)	Number	111	201	198	219	213	170	206	172	189	101
	Median	11	7	7	8	3	19	12	14	7	29
	75th percentile	34	30	30	31	22	58	48	47	31	56
	90th percentile	73	92	88	114	63	142	157	86	117	90
Primary Care interval (Symptomatic)	Number	97	167	160	172	141	70	99	109	142	
	Median	0	0	0	0	0	20	0	17	7	n/a
	75th percentile	0	0	1	0	0	37	0	30	15	
	90th percentile	3	7	6	3	10	75	14	82	38	
Diagnostic interval (Symptomatic)	Number	111	200	197	216	207	166	203	184	191	101
	Median	29	12	19	14	8	25	20	28	13	13
	75th percentile	54	18	35	21	26	56	37	42	21	24
	90th percentile	92	36	49	49	49	202	71	79	46	48
Diagnostic interval (Screen-detected)	Number	113	153	159	86	131	184	126	125	152	158
	Median	25	15	20	19	24	26	27	23	21	15
	75th percentile	45	21	29	26	36	42	44	43	32	25
	90th percentile	58	34	42	41	48	66	66	113	40	41
Diagnostic interval (All)	Number	224	353	356	302	338	350	329	309	343	259
	Median	29	13	20	15	14	26	22	26	16	14
	75th percentile	49	21	33	21	31	46	39	42	28	25
	90th percentile	70	36	48	43	49	90	68	94	45	41
Treatment interval (Symptomatic)	Number	115	203	202	241	230	201	216	198	196	113
	Median	24	29	24	21	20	35	19	38	14	22
	75th percentile	33	41	37	29	29	49	27	55	24	29
	90th percentile	45	55	54	40	44	65	34	72	33	42
Treatment interval (Screen-detected)	Number	115	155	158	86	134	195	120	155	170	165
	Median	27	31	34	24	19	35	13	39	19	21
	75th percentile	36	40	44	35	29	48	21	51	28	29
	90th percentile	49	62	69	44	36	65	28	66	38	40
Treatment interval (All)	Number	231	359	361	330	368	400	341	353	366	278
	Median	25	30	29	22	20	35	15	39	15	22
	75th percentile	35	41	41	31	29	48	24	54	27	29
	90th percentile	46	57	61	41	41	65	33	71	36	41
Total interval (Symptomatic)	Number	104	190	186	205	189	173	184	155	177	98
	Median	70	57	58	50	42	92	54	92	42	71
	75th percentile	96	82	99	78	73	158	121	128	89	101

For peer review only - http://bmjopen.bmj.com/site/about/guidelines.xhtml

For peer review only

	90th percentile	218	138	149	147	170	273	231	188	170	169
Total interval (All)	Number	217	343	341	291	320	354	298	277	327	253
	Median	60	52	55	46	44	78	48	76	42	42
	75th percentile	81	70	84	69	68	116	79	116	63	68
	90th percentile	123	114	129	127	118	209	168	182	120	101

[1] In Sweden, data on the primary care interval were not collected.

For peer review only

Table 5. *The prevalence ratios[1] (PRs) of experiencing differing primary care intervals for symptomatic breast cancer patients.*

	**Wales** (N=97)	**England** (N=167)	**Scotland** (N=160)	**N Ireland** (N=172)	**Denmark** (N=141)	**Ontario** (N=70)	**Norway** (N=99)	**Manitoba** (N=109)	**Victoria** (N=142)	**Sweden[2]** (N=0)
Interval ≥ 5 days										
PR (95% CI)	ref.	1.7 (0.7-4.2)	2.0 (0.8-4.9)	1.3 (0.5-3.3)	2.0 (0.8-4.8)	**12.9** (5.9-28.2)	2.2 (0.9-5.6)	**12.9** (5.9-28.1)	**11.1** (5.1-24.4)	n/a
Interval ≥ 14 days										
PR (95% CI)	ref.	1.6 (0.5-4.9)	1.5 (0.5-4.7)	1.4 (0.5-4.3)	2.1 (0.7-6.3)	**15.1** (5.7-40.1)	2.4 (0.8-7.4)	**14.3** (5.4-37.8)	**6.7** (2.5-18.1)	n/a

Bold: significance level of p ≤0.05; n/a=not applicable
[1] Adjusted for differences in age and comorbidity among the patients, with Wales as reference.
[2] In Sweden, no information on primary care interval was collected.

For peer review only

Table 6. *The differences in interval lengths (days) between Wales, the reference, and the other nine jurisdictions.*

		Wales	England	Scotland	N Ireland	Denmark	Ontario	Norway	Manitoba	Victoria	Sweden
**Patient Interval (Symptomatic)**	Number	111	201	198	219	213	170	206	172	189	101
	Median	11 (ref.)	-3(-9,3)	-3 (-8,3)	-1 (-8,6)	**-7** (-12,-2)	7 (-1,16)	1 (-7,9)	4 (-2,11)	-4 (-9,2)	**17** (11,22)
	75th percentile	34 (ref.)	-7 (-21,7)	-4 (-12,3)	-2 (-8,5)	**-10** (-16,-4)	**19** (5,33)	**16** (7,25)	12 (-1,25)	-6 (-16,4)	**22** (8,36)
	90th percentile	73 (ref.)	21 (-19,61)	11 (-45,66)	30 (-13,73)	-14 (-64,35)	50 (-9,110)	**74** (31,117)	9 (-35,54)	33 (-13,80)	14 (-31,60)
**Diagnostic interval (Symptomatic)**	Number	111	200	197	216	207	166	203	184	191	101
	Median	29 (ref.)	**-18** (-22,-15)	**-10** (-14,-6)	**-16** (-19,-13)	**-22** (-25,-18)	**-6** (-9,-2)	**-10** (-15,-5)	-1 (-5,4)	**-18** (-22,-14)	**-17** (-23,-12)
	75th percentile	54 (ref.)	**-39** (-45,-33)	**-24** (-29,-19)	**-37** (-42,-33)	**-34** (-40,-28)	-3 (-13,6)	**-21** (-36,-6)	**-16** (-23,-8)	**-34** (-52,-15)	**-33** (-39,-27)
	90th percentile	92 (ref.)	**-68** (-88,-48)	**-58** (-91,-25)	**-62** (-80,-45)	**-55** (-75,-35)	**73** (35,112)	**-35** (-62,-8)	**-30** (-46,-15)	**-63** (-78,-47)	**-58** (-81,-35)
**Diagnostic interval (Screen-detected)**	Number	113	153	159	86	131	184	126	125	152	158
	Median	25 (ref.)	-11 (-22,1)	-7 (-18,5)	-7 (-19,4)	-1 (-17,15)	0 (-12,12)	1 (-11,13)	-2 (-15,10)	-5 (-17,6)	-12 (-24,0)
	75th percentile	45 (ref.)	**-24** (-36,-13)	**-17** (-28,-6)	**-20** (-31,-8)	-9 (-21,2)	-3 (-15,8)	-1 (-15,13)	-4 (-15,7)	**-14** (-25,-3)	**-21** (-34,-7)
	90th percentile	58 (ref.)	**-23** (-30,-16)	**-16** (-22,-11)	**-19** (-26,-12)	**-10** (-14,-6)	9 (-6,24)	8 (-3,20)	**55** (38,71)	**-17** (-26,-8)	**-17** (-20,-13)
**Diagnostic interval (All)**	Number	224	353	356	302	338	350	329	309	343	259
	Median	29 (ref.)	**-14** (-16,-12)	**-8** (-11,-6)	**-13** (-15,-11)	**-14** (-16,-11)	-2 (-5,1)	**-6** (-9,-2)	-1 (-5,2)	**-12** (-15,-10)	**-14** (-17,-11)
	75th percentile	49 (ref.)	**-30** (-34,-25)	**-18** (-23,-14)	**-29** (-33,-25)	**-20** (-25,-16)	-5 (-15,4)	**-11** (-20,-2)	**-8** (-12,-3)	**-22** (-26,-19)	**-25** (-32,-17)
	90th percentile	70 (ref.)	**-35** (-39,-31)	**-23** (-30,-16)	**-26** (-34,-19)	**-21** (-40,-2)	**20** (9,31)	-1 (-16,14)	**22** (7,38)	**-27** (-40,-13)	**-29** (-36,-21)
**Treatment interval (Symptomatic)**	Number	115	203	202	241	230	201	216	198	196	113
	Median	24 (ref.)	**6** (1,11)	1 (-3,5)	-3 (-7,1)	-3 (-7,1)	**12** (8,16)	-5 (-11,1)	**15** (11,19)	**-10** (-14,-6)	-2 (-6,2)
	75th percentile	33 (ref.)	**8** (4,12)	4 (-1,9)	-3 (-8,1)	-4 (-9,0)	**16** (11,21)	**-6** (-9,-2)	**22** (18,26)	**-9** (-16,-2)	-4 (-13,5)
	90th percentile	45 (ref.)	10 (-1,20)	11 (-4,26)	-4 (-19,11)	-1 (-14,12)	**20** (10,31)	-11 (-23,2)	**28** (10,46)	-11 (-26,4)	-3 (-15,10)
**Treatment interval (Screen-detected)**	Number	115	155	158	86	134	195	120	155	170	165
	Median	27 (ref.)	**5** (1,8)	**8** (4,11)	0 (-5,5)	**-6** (-10,-1)	**9** (7,12)	**-12** (-15,-10)	**12** (9,16)	-5 (-12,1)	**-3** (-6,-1)
	75th percentile	36 (ref.)	4 (-2,10)	10 (-3,22)	0 (-6,7)	-7 (-16,3)	**11** (3,20)	**-15** (-27,-4)	**15** (7,23)	-7 (-16,2)	-6 (-17,5)
	90th percentile	49 (ref.)	5 (-8,18)	**15** (4,25)	-9 (-20,3)	**-14** (-26,-2)	9 (-4,22)	**-24** (-35,-13)	12 (-1,24)	**-14** (-27,-1)	-11 (-21,0)
**Treatment interval (All)**	Number	231	359	361	330	368	400	341	353	366	278
	Median	25 (ref.)	5 (0,11)	4 (0,8)	-4 (-8,1)	**-5** (-9,-1)	**10** (6,14)	**-10** (-14,-6)	**14** (9,18)	**-10** (-14,-6)	-3 (-7,0)
	75th percentile	35 (ref.)	**6** (2,10)	**6** (1,10)	-4 (-8,0)	**-6** (-10,-2)	**13** (10,17)	**-11** (-16,-6)	**20** (16,23)	**-9** (-13,-5)	**-6** (-10,-2)
	90th percentile	46 (ref.)	**8** (2,14)	12 (-1,26)	**-6** (-10,-2)	**-6** (-9,-2)	**17** (11,23)	**-14** (-17,-12)	**23** (16,29)	**-11** (-14,-9)	**-7** (-10,-4)
**Total interval**	Number	104	190	186	205	189	173	184	155	177	98

For peer review only - http://bmjopen.bmj.com/site/about/guidelines.xhtml

**(Symptomatic)**	Median	70 (ref.)	**-14** (-24,-3)	**-12** (-23,-2)	**-20** (-31,-8)	**-25** (-38,-11)	**28** (15,42)	-15 (-34,5)	**22** (12,31)	**-28** (39,-17)	-1 (-11,10)
	75th percentile	96 (ref.)	**-16** (-27,-5)	-5 (-18,9)	**-18** (-33,-3)	**-25** (-39,-12)	**69** (31,107)	**27** (8,46)	**30** (8,53)	-13 (-27,0)	3 (-30,35)
	90th percentile	218 (ref.)	**-71** (-102,-41)	**-60** (-85,-34)	**-57** (-66,-48)	**-41** (-66,-16)	**79** (66,93)	8 (-15,30)	**-17** (-34,-1)	-21 (-67,26)	**-39** (-67,-10)
**Total interval (All)**	Number	217	343	337	291	320	354	298	277	327	253
	Median	60 (ref.)	**-9** (-14,-3)	-4 (-11,2)	**-13** (-18,-8)	**-15** (-26,-4)	**16** (6,26)	**-13** (-21,-6)	**17** (9,24)	**-18** (-24,-12)	**-17** (-25,-10)
	75th percentile	81 (ref.)	**-12** (-20,-4)	1 (-9,10)	**-13** (-20,-6)	**-14** (-20,-9)	**32** (24,41)	-4 (-12,4)	**35** (21,48)	**-19** (-25,-12)	**-17** (-26,-7)
	90th percentile	123 (ref.)	-13 (-34,9)	3 (-12,18)	-5 (-22,11)	-8 (-38,22)	**73** (33,114)	**32** (2,62)	**47** (13,82)	-12 (-37,13)	-24 (-51,4)

The differences for the median, 75th and 90th percentiles are calculated as marginal effects after quantile regression by setting the continuous covariate age to its mean value and comorbidity to the mode. Significant results are shown in bold. The actual number of days are included for Wales are shown in Table 5. Note that analyses of primary care intervals are presented in Table 6.

For peer review only

Figure One – Diagnostic and treatment intervals[23]

For peer review only

For peer review only - http://bmjopen.bmj.com/site/about/guidelines.xhtml

Figure Two – Time intervals for each jurisdiction compared with Wales (reference)*

**Adjusted for differences in comorbidity and age. Differences in interval lengths (in days) are shown for the median, 75th and 90th percentiles compared to the reference used for the regression analyses, Wales. Wales is represented by the axis, with jurisdictions with shorter intervals shown to the left of the axis, and jurisdictions with longer intervals shown to the right of the axis for each graph. Solid-fill bars indicate statistically significant differences compared with Wales. Primary care interval not shown, see Table 6.*

For peer review only

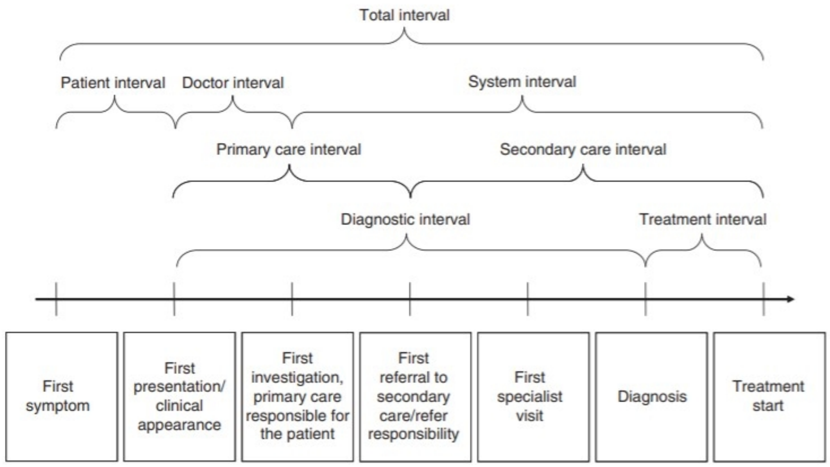

Diagnostic and treatment intervals

155x74mm (144 x 144 DPI)

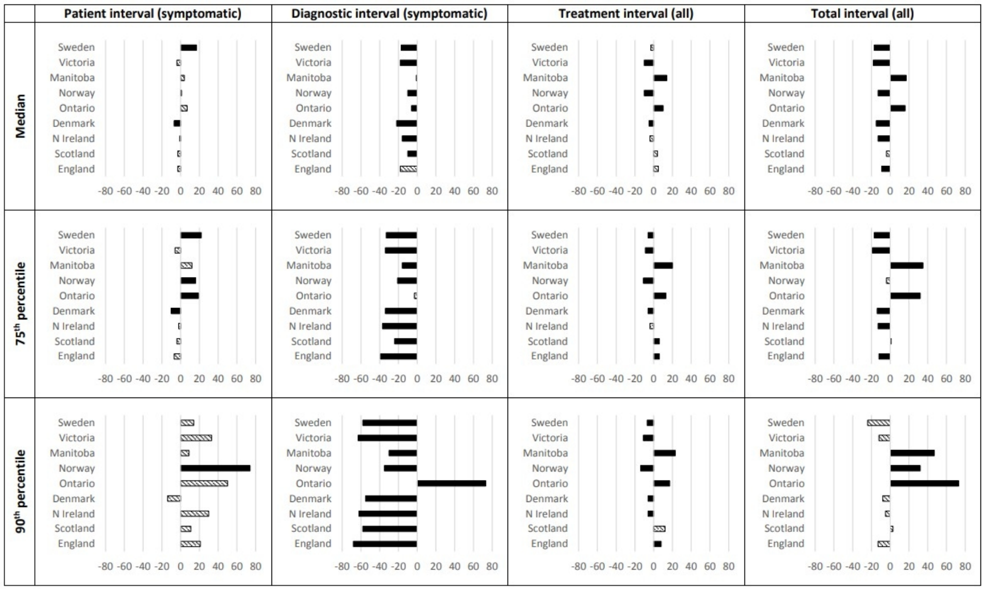

Time intervals for each jurisdiction compared with Wales (chosen as the reference jurisdiction as it had the lowest ovarian cancer survival in the ICBP Module 1 cancer survival analysis). Adjusted for differences in comorbidity and age. Differences in interval lengths (in days) are shown for the median, 75th and 90th percentiles compared to the reference used for the regression analyses, Wales. Wales is represented by the axis, with jurisdictions with shorter intervals shown to the left of the axis, and jurisdictions with longer intervals shown to the right of the axis for each graph. Solid-fill bars indicate statistically significant differences compared with Wales. Primary care interval not shown, see Table 6.

208x126mm (144 x 144 DPI)

ICBP
International Cancer Benchmarking Partnership

International Cancer Benchmarking Partnership Module 4

Patient questionnaire
Breast Cancer

Thank you very much for taking the time to fill in this questionnaire – it should take about 20 minutes to complete. We are sending the questionnaire to a large sample of people who we understand have had a diagnosis of breast cancer. If this has been sent to you in error and you do not have cancer, please do not continue and return the documents in the prepaid envelope.

Our aim is to gain a better understanding of the process by which people have their cancer diagnosed. We would also like to find out more about the symptoms they experience (if any), and the pathway they follow from start of symptoms to treatment of their cancer. This will help in identifying ways in which cancers can be diagnosed quickly and effectively. Thank you once again for your time.

This information is confidential and will not be passed to anyone involved in your treatment.

Name: __________

Date of Birth: __________

Address: __________

____

____

Copyright property of the International Benchmarking Partnership (ICBP) and Module 4 collaborators. Usage must be authorised

For peer review only - http://bmjopen.bmj.com/site/about/guidelines.xhtml

Sample

ICBP
International Cancer Benchmarking Partnership

Consent form

Please read the consent form and sign your name and date **BELOW**.

If you require any clarification, please do not hesitate to ring the study team members. Their contact details are found on the information sheet.

Please be reassured that your responses are completely confidential and will not be passed to anyone involved in your treatment. For the purposes of the study it is important that you agree to consent to all the statements listed below.

- I confirm that I have read the attached information sheet and I understand why the research is being done.
- I am willing for the team to request information from my GP and hospital doctors which is relevant to the audit as described in the information sheet.
- I give permission for my details (name, address) to be given to the cancer registry (NHS Information Centre for Health and Social Care) for follow up.
- I agree for the information I have provided and any other relevant information from my medical records to be stored as described in the information sheet under the custodianship of University College London.
- I consent to sharing of coded data which contains no personal identifiers between researchers, some of whom are located outside the European Union.
- I consent for use of my data if I become mentally incapacitated during the course of the project.

I agree to all the statements listed and consent to participate in the study.

Name (Please print) ____________________

Signature: ____________________ Date: ____________________

If we have any questions, may we phone you for clarification? (Please tick) ☐ **Yes** ☐ **No**

If **Yes**, please provide your telephone number: ____________________

Sample

Copyright property of the International Benchmarking Partnership (ICBP) and Module 4 collaborators. Usage must be authorised

27 May 2015 version 4

For peer review only - http://bmjopen.bmj.com/site/about/guidelines.xhtml

1. **Please can you confirm the details of your GP/GP practice (name, practice address – as best as you can remember): We appreciate that you may have more than one GP involved in your care – in which case, we are interested in the GP you would say provides the majority of your care, particularly relating to the cancer you've had diagnosed.**

Name of doctor

Name of practice

Address

Postcode

Town

Sample

Copyright property of the International Benchmarking Partnership (ICBP) and Module 4 collaborators. Usage must be authorised

ICBP International Cancer Benchmarking Partnership

2. Which of the following best describes the events which led to your diagnosis of cancer? (please tick only **ONE** answer)

	✓
I had symptoms/I noticed a bodily change and went to see a doctor (e.g. GP)	
I had symptoms/I noticed a bodily change and went/was taken to Accident and Emergency (A&E)	
I had seen a doctor/GP with symptoms, but went/was taken to Accident and Emergency (A&E) when things worsened	
I was being investigated by my doctor(s) for another problem during which time the cancer was discovered	
I had a cancer screening test (mammogram) as part of a breast screening programme (e.g. the NHS Breast Screening Programme in England – NHSBSP)	
Other (please describe):	

What date did you have this screening test? If you cannot remember the exact date, you can fill in the month and the year.

Day (optional), month, year

D	D	M	M	Y	Y	Y	Y

Sample

For peer review only

Copyright property of the International Benchmarking Partnership (ICBP) and Module 4 collaborators. Usage must be authorised

4 / May 2013 Version 4

3. The following health concerns or symptoms are commonly experienced with breast cancer.

Changes in the appearance of the breast, eg:<br>■ the nipple<br>■ the skin of the breast<br>■ the shape of the breast<br>■ the size of the breast
Nipple discharge including bleeding
Lump, swelling or thickening in breast or armpit
Fatigue
Unexplained weight loss
Loss of appetite

Sample

Please write down ALL health concern(s) or symptom(s) you may have had before contacting a doctor or taking part in screening. It does not matter if they are not included in the list above:

Please write your health concern(s) or symptom(s) in the boxes below:
1)
2)
3)
4)
5)
6)

	✓
This is not applicable to me (e.g. I did not have any symptoms), please tick	

Copyright property of the International Benchmarking Partnership (ICBP) and Module 4 collaborators. Usage must be authorised

For peer review only - http://bmjopen.bmj.com/site/about/guidelines.xhtml

ICBP International Cancer Benchmarking Partnership

4. Please write down your best estimate of the date you noticed the first of these health concern(s) or symptom(s). If you cannot remember the exact date, you can fill in the month and the year.

Day (optional), month, year

D	D	M	M	Y	Y	Y	Y

	✓
This is not applicable to me (e.g. I had no symptoms), please tick	

5. Approximately how long did you have health concern(s) or symptom(s) before contacting a doctor? (Please think of the first visit to the doctor, not re-visits after that). Please tick only **ONE** answer.

	✓
Less than 1 week	
1-2 weeks	
3-4 weeks	
5-7 weeks	
2-5 months	
6-12 months	
More than 12 months	

	✓
This is not applicable to me (e.g. I had no symptoms), please tick	

Sample

For peer review only

Copyright property of the International Benchmarking Partnership (ICBP) and Module 4 collaborators. Usage must be authorised

6a. Once you contacted a practice about your health concern(s) or symptom(s), how long did it take to get an appointment with a doctor? (Please think of the first visit to the doctor, to discuss your health concern(s) or symptom(s)).
Please tick only **ONE** answer. ✓

Same day/next day	
Within 1 week	
1-2 weeks	
3-4 weeks	
Longer	

If there was no waiting time (e.g. you went/were taken to A&E), please tick this box	

This is not applicable to me (e.g. I had no symptoms), please tick	

6b. What was the date you first saw your doctor about your health concern(s) or symptom(s)? If you cannot remember the exact date, you can fill in the month and the year.

Day (optional), month, year

D	D	M	M	Y	Y	Y	Y

✓

This is not applicable to me (e.g. I had no symptoms), please tick	

Sample

Copyright property of the International Benchmarking Partnership (ICBP) and Module 4 collaborators. Usage must be authorised

For peer review only - http://bmjopen.bmj.com/site/about/guidelines.xhtml

ICBP
International Cancer Benchmarking Partnership

7. How many times did you visit the following for the investigation of your symptoms before your cancer was diagnosed?

	Please write down the number of visits
GP	
Hospital	
Consultant/specialist outside of a hospital	

This is not applicable to me (e.g. I had no symptoms)	

8a. After your doctor referred you to a specialist, how long did it take you to get an appointment? Please tick only **ONE** answer.

Less than 1 week	
1-2 weeks	
3-4 weeks	
5-7 weeks	
2-5 months	
6-12 months	
More than 12 months	

This is not applicable to me (eg my doctor did not refer me), please tick	

Sample
For peer review only

Copyright property of the International Benchmarking Partnership (ICBP) and Module 4 collaborators. Usage must be authorised
For peer review only - http://bmjopen.bmj.com/site/about/guidelines.xhtml

8b. What was the date of your first appointment with a doctor, involved in investigating and/or treating your cancer, to whom you were referred?
If you cannot remember the exact date, you can fill in the month and the year.

Day (optional), month, year

D	D	M	M	Y	Y	Y	Y

This is not applicable to me (e.g. my doctor did not refer me), please tick	

9. What was the date you were told you had cancer? If you cannot remember the exact date, you can fill in the month and the year.

Day (optional), month, year

D	D	M	M	Y	Y	Y	Y

Sample

For peer review only

Copyright property of the International Benchmarking Partnership (ICBP) and Module 4 collaborators. Usage must be authorised

For peer review only - http://bmjopen.bmj.com/site/about/guidelines.xhtml

ICBP
International Cancer
Benchmarking Partnership

10. Have you had any of the following treatments for your cancer yet? If so, please can you estimate the date this treatment started? Please tick **ALL** that apply. If you cannot remember the exact date, you can fill in the month and the year.

	Type of treatment		Date of treatment (give first date if you had more than one)
a.	Surgery	☐ Yes ☐ No	Day (optional), month, year D D M M Y Y Y Y
b.	Chemotherapy	☐ Yes ☐ No	Day (optional), month, year D D M M Y Y Y Y
c.	Radiotherapy	☐ Yes ☐ No	Day (optional), month, year D D M M Y Y Y Y
d.	Other Please specify:	☐ Yes ☐ No	Day (optional), month, year D D M M Y Y Y Y
e.	Treatment not started yet	☐ Yes	

Sample

For peer review only

Copyright property of the International Benchmarking Partnership (ICBP) and Module 4 collaborators. Usage must be authorised

For peer review only - http://bmjopen.bmj.com/site/about/guidelines.xhtml

11. Who is the consultant doctor who has taken responsibility for diagnosing and or/treating your cancer?

Name of consultant:
Hospital name:
Hospital department:

Please can you answer some more general questions about your health?
It will help us in interpreting your responses to this questionnaire to know about your general health and other health problems you may have had in the past.

12. Looking back to the 2 years before you were diagnosed with cancer, would you say your general health was (Please tick only **ONE** answer.)**:**

Very good	
Good	
Fair	
Poor	
Very poor	

13. Have you been treated before for any of the conditions below?
Please tick 'yes' or 'no' for each condition:

Heart disease	☐ **Yes**	☐ **No**
Stroke	☐ **Yes**	☐ **No**
Lung disease	☐ **Yes**	☐ **No**
Diabetes	☐ **Yes**	☐ **No**

Copyright property of the International Benchmarking Partnership (ICBP) and Module 4 collaborators. Usage must be authorised

Sample

For peer review only - http://bmjopen.bmj.com/site/about/guidelines.xhtml

ICBP International Cancer Benchmarking Partnership

Finally, a little more information about you. The information you provide below will help us to analyse the results of the survey in more detail.

14. Which of these best describes your ethnic group? (please tick one box, as appropriate). If you are descended from more than one ethnic or racial group, please tick the group you consider you belong to, or tick 'any other ethnic group'.

	✓		✓		✓		✓
White		Chinese		Black - Caribbean		Black - African	
Black - other		Indian		Pakistani		Bangladeshi	
Any other ethnic group, please specify:							

15. What is the main language spoken in your home? Please tick

	✓
English	
Other, please specify:	

16. What is the highest level of education you have achieved?
Please tick only **ONE** answer.

	✓
Finished school at or before the age of fifteen	
Completed GCSEs, O-levels or equivalent	
Completed A Levels or equivalent	
Completed further education but not a degree	
Completed a Bachelor's degree / Masters degree / PhD	
Other, please specify:	

Sample

For peer review only

Copyright property of the International Benchmarking Partnership (ICBP) and Module 4 collaborators. Usage must be authorised.

17. **Have you ever smoked cigarettes, including hand-rolled ones, pipes or cigars?**

☐ **Yes** ☐ **No**

18. **Are you a current smoker, smoking either cigarettes, including hand-rolled ones, pipes or cigars?**

☐ **Yes** ☐ **No**

19. **If you are a current smoker or have smoked in the past, how many cigarettes, including hand-rolled ones, pipes or cigars on average do you smoke/have you smoked per day?**

Number per day:	

Sample

For peer review only

Copyright property of the International Benchmarking Partnership (ICBP) and Module 4 collaborators. Usage must be authorised

ICBP
International Cancer Benchmarking Partnership

20. Further comments

Please add anything else that you would like to tell us about your cancer diagnosis or treatment.

Sample

For peer review only

Thank you very much for taking the time to complete this questionnaire.

Copyright property of the International Benchmarking Partnership (ICBP) and Module 4 collaborators.
Usage must be authorised

For peer review only - http://bmjopen.bmj.com/site/about/guidelines.xhtml

ICBP International Cancer Benchmarking Partnership

International Cancer Benchmarking Partnership Module 4

Primary Care Audit
Breast Cancer

Thank you very much for agreeing to fill in this questionnaire. As part of an international study examining differences in cancer survival, we are sending the questionnaire to health care providers of a sample of consented patients with cancer.

Our aim is to gain a better understanding of the process by which people have their cancer diagnosed – the symptoms they experience, and the pathway they follow from onset of symptoms to treatment of their cancer. This will help in identifying ways in which cancers can be diagnosed and treated quickly and effectively. Thank you once again for your time.

Please can you refer to your patient's notes in completing the questionnaire as this will help in obtaining accurate data on time points.

If you would prefer to return this questionnaire without the patient details, please tear off along the dotted line

ID-number: Jurisdiction-ID + Patient-ID:

Copyright property of the International Benchmarking Partnership (ICBP) and Module 4 collaborators. Usage must be authorised

Sample

For peer review only - http://bmjopen.bmj.com/site/about/guidelines.xhtml

International Cancer Benchmarking Partnership

Sample

For peer review only

Patient information

ID-number: Jurisdiction-ID + Patient-ID:

Full name:

Address:

Postcode:

Date of birth: D D M M Y Y Y Y

Copyright property of the International Benchmarking Partnership (ICBP) and Module 4 collaborators. Usage must be authorised

For peer review only - http://bmjopen.bmj.com/site/about/guidelines.xhtml

1. Duration of symptoms

Please estimate how long your patient had symptom(s), attributable to breast cancer, before attending your practice (or other health service).
We appreciate that identifying a 'date of first symptom' is not always straightforward – particularly when there are multiple and/or chronic symptoms. Nevertheless, we hope you can provide a 'best estimate'.

**Estimate of symptom duration** (please tick **one**):	✓	**What were the symptoms?** Please describe:
Less than 1 week		
1 to 4 weeks		
5 to 7 weeks		
2-5 months		
6-12 months		
More than 12 months		
Not possible to estimate		
No symptoms (e.g. screen detected cancers)		

Sample

Copyright property of the International Benchmarking Partnership (ICBP) and Module 4 collaborators. Usage must be authorised

For peer review only - http://bmjopen.bmj.com/site/about/guidelines.xhtml

ICBP International Cancer Benchmarking Partnership

2. Pathway of presentation

2.1 Through what route did the patient first present? Please tick **ONE**.

	✓	
Your patient first presented to primary care (either in-hours or out-of-hours)		Please can you provide your best approximation of the date of this primary care visit D D M M Y Y Y Y
Your patient presented straight to A&E (with or without your involvement)		
Your patient first presented to primary care, but then at a later date presented to A&E as an emergency (with or without your involvement)		Please can you provide your best approximation of the date of this primary care visit D D M M Y Y Y Y
Your patient's breast cancer was diagnosed through an organised screening programme (e.g. not as a result of investigation of symptoms)		
Other – please describe:		

Sample

For peer review only

Copyright property of the International Benchmarking Partnership (ICBP) and Module 4 collaborators. Usage must be authorised

For peer review only - http://bmjopen.bmj.com/site/about/guidelines.xhtml

3. Date you ordered any tests/investigations in response to symptom(s).

We are interested in any kind of tests/investigations (e.g. imaging etc) that you may have ordered. Please only consider the tests/investigations that you ordered yourself. Please tick **ALL** that apply and put in the date that the test/investigation was ordered:

	✓	
Mammogram		D D M M Y Y Y Y
Breast ultrasound		D D M M Y Y Y Y
Breast biopsy		D D M M Y Y Y Y
Other (please specify):		D D M M Y Y Y Y

Sample

4. Date of referral to specialist medical services

At what date did you **first** refer the patient to hospital or another specialist transferring the responsibility for on-going investigation/treatment to other medical services?

D	D	M	M	Y	Y	Y	Y

Copyright property of the International Benchmarking Partnership (ICBP) and Module 4 collaborators. Usage must be authorised

For peer review only - http://bmjopen.bmj.com/site/about/guidelines.xhtml

ICBP
International Cancer Benchmarking Partnership

5. Nature of this referral

5.1 Do you know the date that the patient was seen for this referral?

☐ **Yes**, please provide the date: | D | D | M | M | Y | Y | Y | Y |

☐ **No**

5.2 If you did make a referral to specialist services, which of the following best describes the nature/characteristics of this referral? Please tick **one**.

	✓
Emergency admission: a referral to A&E (or equivalent) for immediate admission	
An urgent referral for assessment of cancer symptoms/signs/test results (Note this will be within 2 weeks for England/Wales)	
A less urgent referral in which cancer is raised as a possibility (Note this will be greater than 2 weeks for England/Wales)	
A more general referral for investigation and assessment without cancer mentioned	
No referral was made	
Other – please describe:	

5.3 Would you say this patient's diagnostic pathway was conducted predominantly in the public or private system? Please tick **one**.

	✓
Public healthcare system	
Private healthcare system	

Sample

For peer review only

Copyright property of the International Benchmarking Partnership (ICBP) and Module 4 collaborators.
Usage must be authorised

For peer review only - http://bmjopen.bmj.com/site/about/guidelines.xhtml

6. Date of breast cancer diagnosis

This can be decided in different ways. Please provide whichever of the following dates you have to hand. Please tick **all** that apply.

	✓	
Date of histological confirmation [ideal]		D D M M Y Y Y Y
Date results of investigation (histological or other) confirming cancer received		D D M M Y Y Y Y
Date patient was told		D D M M Y Y Y Y
Date biopsy undertaken		D D M M Y Y Y Y
Date patient was first admitted to hospital because of the malignancy		D D M M Y Y Y Y
Other (please specify):		D D M M Y Y Y Y

Sample
For peer review only

Copyright property of the International Benchmarking Partnership (ICBP) and Module 4 collaborators.
Usage must be authorised
For peer review only - http://bmjopen.bmj.com/site/about/guidelines.xhtml

ICBP International Cancer Benchmarking Partnership

7. Additional information

Finally, we are interested to know what other conditions your patient has, and the severity/impact of these conditions

Have you and/or any of your partners treated this patient (or has the patient been to hospital) for any of the following conditions? Please tick **ALL** that apply:

Cardiovascular disease	☐ Yes ☐ No
Stroke	☐ Yes ☐ No
Lung disease	☐ Yes ☐ No
Diabetes	☐ Yes ☐ No

Are there any other comments you would like to make about this patient?

Name (and title):

Signature:

Date:

Thank you very much for taking the time to complete this questionnaire.

Copyright property of the International Benchmarking Partnership (ICBP) and Module 4 collaborators. Usage must be authorised

Sample

For peer review only - http://bmjopen.bmj.com/site/about/guidelines.xhtml

ICBP
International Cancer Benchmarking Partnership

International Cancer Benchmarking Partnership Module 4

Specialist Care Audit

Breast Cancer

Thank you very much for agreeing to fill in this questionnaire – it should take about 10 minutes to complete. As part of an international study examining differences in cancer survival, we are sending the questionnaire to health care providers of a sample of patients with cancer.

Our aim is to gain a better understanding of the process by which people have their cancer diagnosed – the symptoms they experience, and the pathway they follow from onset of symptoms to treatment of their cancer. We hope you can help us with information on this patient's cancer journey once they were referred to specialist cancer services. This will help in identifying ways in which cancers can be diagnosed and treated quickly and effectively.

Thank you once again for your time

Please can you refer to your patient's notes in completing the questionnaire, as this will help in obtaining accurate data on time points.

If you would prefer to return this questionnaire without the patient details, please tear off along the dotted line.

Your patient

is participating in the study.

Sample

Copyright property of the International Benchmarking Partnership (ICBP) and Module 4 collaborators. Usage must be authorised

For peer review only - http://bmjopen.bmj.com/site/about/guidelines.xhtml

ICBP
International Cancer
Benchmarking Partnership

Sample

For peer review only

Patient information

ID-number: Jurisdiction-ID + Patient-ID:

Full name:

Address:

Postcode:

Date of birth: | D | D | M | M | Y | Y | Y | Y |

Copyright property of the International Benchmarking Partnership (ICBP) and Module 4 collaborators.
Usage must be authorised

For peer review only - http://bmjopen.bmj.com/site/about/guidelines.xhtml

1. Date patient first attended hospital/specialist services related to their cancer diagnosis. We appreciate this date can at times be difficult to identify, particularly when there have been multiple visits in the lead up to a definitive diagnosis. Put another way, it's the date that the hospital/specialist service assumed responsibility for on-going investigation/treatment for your patient.

Day (optional), month, year

D	D	M	M	Y	Y	Y	Y

2. How was the patient referred to the hospital/specialist services related to their cancer diagnosis? Please tick.

Was it through a:

	✓		✓
GP referral		Screening	
Referral from general surgery clinic		Medical specialist/ Consultant referral	
Other referral – please specify:			

3. Where did this first contact/appointment happen? Please tick.
Which of the following best describes where this first contact/appointment took place?

	✓		✓
Emergency department ('A&E')		Medical outpatient department, please specify which department	
Oncology general outpatient department		Surgical outpatient department, please specify which department	
Other – please specify:			

Copyright property of the International Benchmarking Partnership (ICBP) and Module 4 collaborators.
Usage must be authorised

Sample

For peer review only

ICBP International Cancer Benchmarking Partnership

4. Date of diagnosis

This can be decided in different ways.
Please tick and complete as many of the following dates as possible.

	✓	
Date of histological confirmation (ideal)		Day (optional), month, year D D M M Y Y Y Y
Date results of investigation confirming cancer received		Day (optional), month, year D D M M Y Y Y Y
Date patient was told		Day (optional), month, year D D M M Y Y Y Y
Date of biopsy		Day (optional), month, year D D M M Y Y Y Y
Date patient was first admitted to hospital because of the malignancy		Day (optional), month, year D D M M Y Y Y Y
Date of MDT confirmation of diagnosis		Day (optional), month, year D D M M Y Y Y Y
Other (please specify):		Day (optional), month, year D D M M Y Y Y Y

Sample
For peer review only

Copyright property of the International Benchmarking Partnership (ICBP) and Module 4 collaborators.
Usage must be authorised

5. Date treatment for the cancer commenced

Based on your records, when would you say that any treatment specifically targeting the patient's cancer started?

Day (optional), month, year

D	D	M	M	Y	Y	Y	Y

6. Additional information

Please can you provide any further information on the patient's cancer:

**TNM**, please tick as appropriate:	
0	
I	
IIA	
IIB	
IIC	
IIIA	
IIIB	
IIIC	
IV	
Not able to stage	

Sample

For peer review only

Copyright property of the International Benchmarking Partnership (ICBP) and Module 4 collaborators. Usage must be authorised

International Cancer Benchmarking Partnership

6.1 Histological subtype:

	✓
Invasive Ductal	
Invasive Lobular	
Invasive Tubular	
DCIS	
Other (please specify):	

Sample

For peer review only

Copyright property of the International Benchmarking Partnership (ICBP) and Module 4 collaborators.
Usage must be authorised

For peer review only - http://bmjopen.bmj.com/site/about/guidelines.xhtml

Further comments

Sample

Copyright property of the International Benchmarking Partnership (ICBP) and Module 4 collaborators.
Usage must be authorised

For peer review only - http://bmjopen.bmj.com/site/about/guidelines.xhtml

ICBP
International Cancer
Benchmarking Partnership

Name (and title):

Signature:

Date:

Sample

Are you a ... (please tick below):

Surgeon	
Medical Oncologist	
Clinical Oncologist	
Clinical Nurse Specialist	
Other (please specify):	

Thank you very much for taking the time to complete this questionnaire.

Copyright property of the International Benchmarking Partnership (ICBP) and Module 4 collaborators.
Usage must be authorised

For peer review only - http://bmjopen.bmj.com/site/about/guidelines.xhtml

Additional File 4. *ICBP M4 Rules for missing, incomplete, multiple response and out of range data*

1. Oversampling
To handle oversampling in Ontario, include only the first 430 consecutive breast cancer patients

2. Language/Participation in study/Presence of cancer
Exclude patients who checked "No, I don't understand the language" or "I don't want to participate in this study" or "I don't have cancer".

3. Survey responders
a) Exclude Patient/PCP/Specialist survey from the analysis, if it was not written by Patient/PCP/Specialist (example: a medical oncologist completed a PCP survey);
b) In the case of duplicates, include only the first survey (example: 2 specialists completed surveys for the same patient).

4. Age
a) Exclude patients with unknown age;
b) Exclude patients younger 40 years;
c) Use registry data, if Age is reported by both patient and registry.

5. No cancer or Previous cancer in the same organ
a) Exclude patents with no cancer based on registry data;
b) Exclude patients with previous cancer in the same organ based on data from registry or free-text for Presentation in the patient survey.

6. Date of consent
Exclude patients with date of consent, which is unknown, before 01.01.2013 or in the future.

7. Multiple responses to Dates
If multiple responses were given to the dates (of first symptom; screening; first presentation to primary care; referral; diagnosis; treatment start), then use the earliest date.

8. Order of Dates
The dates must be in the following order –
a) First symptom; first presentation to Primary Care; referral; diagnosis; treatment start.
b) Screening; diagnosis; treatment start.
If not, check for mistakes.

9. Date of first symptom
Date of first symptom is defined as date of first symptom from patient data.

10. Date of first presentation
Date of first presentation to Primary Care is defined as (in the order of declining priority):
a) date of first presentation to Primary Care from PCP data;
b) date of first presentation to Primary Care and A&E from PCP data;
c) date of first presentation to Primary Care from patient data.

11. Date of referral
Date of referral is defined as date of referral from PCP data.

12. Date of screening
Date of screening is defined as (in the order of declining priority):
a) date of screening from registry;
b) date of screening from patient data.

13. Date of diagnosis
Definition
a) If Registry reports both date of histological confirmation and date of confirming investigation, then use date of histological confirmation.
b) Date of diagnosis (based on patient data, PCP data, specialist data, registry data) is defined as (in the order of declining priority):
- date of diagnosis from registry;
- date of histological confirmation (from specialist data, PCP data);
- date of biopsy (from specialist data, PCP data);
- date of confirming investigation (from specialist data, PCP data);
- date of first hospital admission (from specialist data, PCP data);
- date of MDT confirmation (from specialist data);
- date patient was told (from specialist data, PCP data);
- other date of diagnosis (from specialist data, PCP data, patient data);

Choose a Date from a lower level of hierarchy, if the Date from a higher level is after the Date of consent or more than 9 months (=271 days) before the Date of consent.

Exclusion criteria
a) Unknown date of diagnosis;

For peer review only

b) Date of diagnosis is after the date of consent;
c) Date of diagnosis is more than 9 months before the Date of consent.

14. Date of treatment start
 a) Date of treatment start from patient data is defined as the earliest of the treatment dates for Surgery, Chemo, Radio and Other;
 b) Date of treatment start (based on registry data, specialist data, patient data) is defined as (in the order of declining priority):
 - date of treatment start from registry data,
 - date of treatment start from specialist data,
 - date of treatment start from patient data,
 - anticipated date of treatment from patient data.

15. Imputation of missing day in the date
 Imputation rules for missing day (given month and year are known):
 a) Set missing day to '16';
 b) Consider adjacent dates in a backwards order (from "Treatment" to "First symptom"). For each pair of such adjacent dates: If dates are not in a logical order (e.g. "Treatment" is before "Diagnosis"), but month and year are the same in both dates, and the day was imputed to '16' in one of the dates:
 - Recode the day imputed earlier to '16' to the day from the adjacent date.

16. Considering time
 If patient gave multiple answers to the "How long did you have symptoms before contacting a doctor?" question, then use the option with the shortest time interval.

17. Delay arranging appointment
 If patient gave multiple answers to the "How long did it take to get an appointment with PCP?" question, then use the option with the shortest time interval.

18. Duration of symptoms
 If PCP gave multiple answers to the "Duration of symptoms" question, then use the option with the shortest time interval.

19. Definition of Presentation
 A. Define Presentation within a Data Source (Patient, PCP)
 1. Review the free-text for Presentation (Patient, PCP) and re-code, if possible.
 2. If PCP reports 'VisitPCP and AE' or 'VisitPCP' as Presentation and no symptoms, then check Patient's records. If Patient reports 'Screening' and no symptoms, then re-code Presentation for this case as 'Screening'.
 3. If PCP reports 'Screening' as Presentation and at least one symptom (or "Duration of Symptoms", rule 18), then re-code Presentation to 'Other non-screen-detected'-option.
 4. If PCP reports 'Other' as Presentation and at least one symptom (or "Duration of Symptoms", rule 18), then re-code Presentation to 'Other non-screen-detected'-option.
 5. If Patient reports 'Screening' as Presentation and at least one symptom (or date of first symptom), then re-code Presentation to 'Other non-screen-detected'- option.
 6. If Patient reports 'Other' as Presentation and at least one symptom (or date of first symptom or "Considering time" (rule 16) or "Delay arranging appointment" (rule 17), then re-code Presentation to 'Other non-screen-detected'-option.
 7. In the case of multiple Presentation responses (Patient, PCP sources) - use a single option (in the order of declining priority):
 a) 'VisitPCP and AE',
 b) 'VisitPCP´, 'AE´ (if both 'VisitPCP´ and 'AE´ are given, then re-code as 'VisitPCP and AE'),
 c) 'Other non-screen-detected',
 d) 'Screening',
 e) 'Investigation for another problem' ,
 f) 'Other"

 B. Define Presentation from Alternative Data
 If Presentation hasn't been reported in either of data sources, then define it as (in the order of declining priority):
 1. 'Other non-screen-detected ', if PCP reports at least one symptom (or "Duration of symptoms", rule 18);
 2. 'Other non-screen-detected ', if Patient reports at least one symptom (or date of first symptom);
 3. 'Other non-screen-detected ', if Patient reports "Considering time" (rule 16) or "Delay arranging appointment" (rule 17) and no screening date;
 4. 'Screening', if Patient reports screening date and no symptoms and no date of first symptom;

 C. Define Presentation from Data Source Hierarchy

For peer review only

1. In Wales/England/Scotland (patients of 48-72 years old), Manitoba (patients 48-76 years old), N Ireland, Sweden and Denmark: if Registry reports 'Screening' – use Presentation-data from Registry data.
2. In Wales/England/Scotland (patients of 48-72 years old), Manitoba (patients 48-76 years old), N Ireland, Sweden and Denmark: if Registry reports ' No Screening'– use Presentation-data from (in the order of declining priority):
   a. PCP data (not available in Sweden);
   b. Patient data;

If PCP (or Patient, in the case of PCP data is not available) reports 'Screening', then code Presentation as 'Other non-screen-detected'.

If Presentation from PCP and Patient datasets is missing, then code Presentation as 'Other non-screen-detected'.

3. In Wales/England/Scotland (patients of 48-72 years old), Manitoba (patients 48-76 years old), N Ireland, Sweden and Denmark: if screening status from Registry is missing – use Presentation-data from (in the order of declining priority):
   a. PCP data (not available in Sweden);
   b. Patient data;
4. In Wales/England/Scotland (patients of <48 or >72 years old), Manitoba (patients of <48 or >76 years old), Norway and Ontario – use Presentation-data from (in the order of declining priority):
   a. PCP data;
   b. Patient data.
5. In Victoria: if Registry reports 'Screening organised by government' – use Presentation-data from Registry data.
6. In Victoria: if Registry reports 'No Screening organised by government' – use Presentation-data from (in the order of declining priority):
   a. PCP data;
   b. Patient data.

20. Patient interval
The Patient interval for non-screen-detected patients is defined as (in the order of declining priority):
a) "Date of first presentation to Primary Care" (rule 10) minus "Date of first symptom" (rule 9);
b) If the interval in (a) is unknown or negative: Calculate the interval as the middle of "Considering time" (rule 16) plus the middle of "Delay arranging appointment" (rule 17);
c) If the interval in (a) is unknown or negative and the interval in (b) is unknown: Calculate the interval as the middle of "Duration of symptoms interval" (rule 18).

21. Primary Care interval
The Primary Care interval for non-screen-detected is defined as "Date of referral" (rule 11) minus "Date of first presentation to Primary Care" (rule 10).

22. Diagnostic interval
a) The Diagnostic interval for non-screen-detected is defined as "Date of diagnosis" (rule 13) minus "Date of first presentation to Primary Care" (rule 10);
b) The Diagnostic interval for screen-detected patients is defined as "Date of diagnosis" (rule 13) minus "Date of screening" (rule 12).

23. Treatment interval
The Treatment interval is defined as "Date of treatment start" (rule 14) minus "Date of diagnosis" (rule 13).

24. Total interval
a) The Total interval for non-screen-detected patients is defined as "Date of treatment start" (rule 14) minus "Date of first symptom" (rule 9);
b) The Total interval for screen-detected patients is defined as "Date of treatment start" (rule 14) minus "Date of screening" (rule 12).

25. Range of Time intervals
The time intervals (Patient, Primary Care, Diagnosis, Treatment, Total) must be in range 0-1 year.

If > 1 year: set the interval to 365 days
If negative: set the interval to 0.

For each jurisdiction calculate the number of imputations due to:

For peer review only

a. unknown day in a date (given known month and year);<br>b. very large(>1 year) interval;<br>c. negative interval.
26. <u>Number of visits</u><br>If patient gave multiple answers to the "Number of visits" questions, then use the option with a fewer number of visits.
27. <u>Specialist waiting time interval</u><br>If patient gave multiple answers to the ""How long did it take to get an appointment with specialist?" question, then use the option with the shortest time interval.
28. <u>Type of treatment</u><br>If patient ticked both "Yes" and "No" as answers to the "Type of treatment (Surgery, Chemotherapy, Radiotherapy)" questions, then choose "Yes" answer.
29. <u>Health state</u><br>If patient gave multiple answers to the "Health state" question, then use the option with a better health condition.
30. <u>Comorbidity</u><br>a) If patient ticked both "Yes" and "No" as answers to the "Comorbidity (Heart disease, Stroke, Lung disease, Diabetes)" questions, then choose "Yes" answer;<br>b) If both patient and PCP report "Comorbidity", then use the PCP Data.
31. <u>Ethnicity</u><br>a) If patient didn't report "Ethnicity", then use the information from (in the order of declining priority):<br>- "Ethnicity_Other_Details";<br>- "Other main language spoken at home";<br>- "The main language spoken at home" (only for Victoria);<br>- "The main language spoken at home is the chief one for this jurisdiction"="Yes" given "Main language spoken at home is other than the main one for this jurisdiction"="No";<br><br>b) Consider Ethnicity as unknown, if answers to the "Ethnicity" question are multiple and belong to different categories ('white', 'Asian', 'black', 'other').
32. <u>Education</u><br>If patient gave multiple answers to the "Education" question, then use the option with a higher level of education.
33. <u>Smoking Current</u><br>a) If patient ticked both "Yes" and "No" as answers to the "Smoking Current" question, then use "Yes" answer;<br>b) If patient hasn't ticked neither "Yes" nor "No, then consider this case as Unknown.
34. <u>Smoking Number</u><br>If patient reports "SmokingNumber" as text, then re-code using following rules:<br><br>a) Where there is a number smoked /day – accept number;<br>b) Where a range has been given – take the upper value;<br>c) Where patient has put 10+ or 20+ - capture this as 11 or 21;<br>d) Where number of cigarettes smoked in the past and currently being smoked are provided - average the numbers;<br>e) Non entries code as "." ;<br>f) Non-smokers (eg, "nil", "N/A") are coded as "0".
35. <u>Smoked ever</u><br>a) If patient ticked both "Yes" and "No" as answers to the "Smoking ever" question, then use "Yes" answer;<br>b) If patient hasn't ticked neither "Yes" nor "No": consider it as "Yes", if patient is a current smoker ("Smoking_Current="Yes"") or has specified a number of cigarettes ("SmokingNumber">0). Otherwise consider this case as Unknown.<br>c) If patient has ticked "No": recode it to "Yes", if patient is a current smoker "Smoking_Current="Yes").
36. <u>Nature of referral</u><br>a) Review free-text for " Nature of referral" (PCP Data) and re-code, if possible;<br>b) In the case of multiple responses, use a single option as (in the order of declining priority):<br>- "Referral for immediate admission";<br>- "Urgent referral";<br>- "Less urgent referral";<br>- "General referral" ;<br>- "No referral";<br>- "Other".
37. <u>Refer Public or Private</u><br>a) If PCP ticked both "Public" and "Private" as answers to the "Refer Public or Private" question, then use "Private" answer;<br>b) If PCP hasn't ticked neither "Public" nor "Private", then consider this case as Unknown.

For peer review only

38. <u>Type of referral</u><br>If specialist gave multiple responses to the "How was the patient referred…" question, then use a single option (in the order of declining priority):<br>- "Screening";<br>- "Respiratory clinic";<br>- "General surgery clinic";<br>- "General gynaecology";<br>- "Specialist/consultant";<br>- "PCP";<br>- "Other".
39. <u>First Attendance Place</u><br>If specialist gave multiple responses to the "First Attendance Place" question, then consider this case as Unknown.
40. <u>Stage-TNM</u><br>a) If specialist gave multiple responses to the "Stage_TNM" question, then use the highest category;<br>b) If registry gave multiple responses to the "Stage_TNM", then use a single option (in the order of declining priority):<br>- stage at time of diagnosis<br>- stage at surgery<br>- stage at oncology<br>c) If "Stage_TNM" is reported by both the specialist and registry, then use the registry data.

For peer review only

Additional File 5. *Comparisons of the participants and the study base of the eligible breast cancer women*

	Eligible women		Participating women		p-value
	n	(%)	n	(%)	
Women, total	19,419	(81.3%)	4,458	(18.7%)	
**Age**					
Under 40	83	(0.4%)	11	(0.2%)	<0.001*
40-49	2,874	(14.8%)	676	(15.2%)	
50-59	4,756	(24.5%)	1,222	(27.4%)	
60-69	5,436	(28%)	1,434	(32.2%)	
70-79	3,696	(19%)	791	(17.7%)	
80-89	2,092	(10.8%)	297	(6.7%)	
90+	383	(2.0%)	27	(0.6%)	
Missing	99	(0.5%)	0	(0.0%)	
**Deceased**					
3 months	176	(18.6%)	2	(5.1%)	<0.05
6 months	213	(22.5%)	5	(12.8%)	
9 months	236	(25.0%)	12	(30.8%)	
12 months	320	(33.9%)	20	(51.3%)	
**Tumour stage**					
I	7,220	(42.9%)	1,807	(49.0%)	<0.001*
II	6,231	(37.0%)	1,311	(35.6%)	
III	2,028	(12.0%)	341	(9.3%)	
IV	653	(3.9%)	87	(2.4%)	
missing	702	(4.2%)	139	(3.8%)	

* both with and without missing

For peer review only

Additional file 6. *The differences in interval lengths (days) between Wales, the reference, and the other nine jurisdictions.*

		Wales	England	Scotland	N Ireland	Denmark	Ontario	Norway	Manitoba	Victoria	Sweden
**Patient Interval (Symptomatic)**	Number	111	201	198	219	213	170	206	172	189	101
	Median	11 (ref.)	-3(-9,3)	-3 (-8,3)	-1 (-8,6)	**-7** (-12,-2)	7 (-1,16)	1 (-7,9)	4 (-2,11)	-4 (-9,2)	**17** (11,22)
	75th percentile	34 (ref.)	-7 (-21,7)	-4 (-12,3)	-2 (-8,5)	**-10** (-16,-4)	**19** (5,33)	**16** (7,25)	12 (-1,25)	-6 (-16,4)	**22** (8,36)
	90th percentile	73 (ref.)	21 (-19,61)	11 (-45,66)	30 (-13,73)	-14 (-64,35)	50 (-9,110)	**74** (31,117)	9 (-35,54)	33 (-13,80)	14 (-31,60)
**Diagnostic interval (Symptomatic)**	Number	111	200	197	216	207	166	203	184	191	101
	Median	29 (ref.)	**-18** (-22,-15)	**-10** (-14,-6)	**-16** (-19,-13)	**-22** (-25,-18)	**-6** (-9,-2)	**-10** (-15,-5)	-1 (-5,4)	**-18** (-22,-14)	**-17** (-23,-12)
	75th percentile	54 (ref.)	**-39** (-45,-33)	**-24** (-29,-19)	**-37** (-42,-33)	**-34** (-40,-28)	-3 (-13,6)	**-21** (-36,-6)	**-16** (-23,-8)	**-34** (-52,-15)	**-33** (-39,-27)
	90th percentile	92 (ref.)	**-68** (-88,-48)	**-58** (-91,-25)	**-62** (-80,-45)	**-55** (-75,-35)	**73** (35,112)	**-35** (-62,-8)	**-30** (-46,-15)	**-63** (-78,-47)	**-58** (-81,-35)
**Diagnostic interval (Screen-detected)**	Number	113	153	159	86	131	184	126	125	152	158
	Median	25 (ref.)	-11 (-22,1)	-7 (-18,5)	-7 (-19,4)	-1 (-17,15)	0 (-12,12)	1 (-11,13)	-2 (-15,10)	-5 (-17,6)	-12 (-24,0)
	75th percentile	45 (ref.)	**-24** (-36,-13)	**-17** (-28,-6)	**-20** (-31,-8)	-9 (-21,2)	-3 (-15,8)	-1 (-15,13)	-4 (-15,7)	**-14** (-25,-3)	**-21** (-34,-7)
	90th percentile	58 (ref.)	**-23** (-30,-16)	**-16** (-22,-11)	**-19** (-26,-12)	**-10** (-14,-6)	9 (-6,24)	8 (-3,20)	**55** (38,71)	**-17** (-26,-8)	**-17** (-20,-13)
**Diagnostic interval (All)**	Number	224	353	356	302	338	350	329	309	343	259
	Median	29 (ref.)	**-14** (-16,-12)	**-8** (-11,-6)	**-13** (-15,-11)	**-14** (-16,-11)	-2 (-5,1)	**-6** (-9,-2)	-1 (-5,2)	**-12** (-15,-10)	**-14** (-17,-11)
	75th percentile	49 (ref.)	**-30** (-34,-25)	**-18** (-23,-14)	**-29** (-33,-25)	**-20** (-25,-16)	-5 (-15,4)	**-11** (-20,-2)	**-8** (-12,-3)	**-22** (-26,-19)	**-25** (-32,-17)
	90th percentile	70 (ref.)	**-35** (-39,-31)	**-23** (-30,-16)	**-26** (-34,-19)	**-21** (-40,-2)	**20** (9,31)	-1 (-16,14)	**22** (7,38)	**-27** (-40,-13)	**-29** (-36,-21)
**Treatment interval (Symptomatic)**	Number	115	203	202	241	230	201	216	198	196	113
	Median	24 (ref.)	**6** (1,11)	1 (-3,5)	-3 (-7,1)	-3 (-7,1)	**12** (8,16)	-5 (-11,1)	**15** (11,19)	**-10** (-14,-6)	-2 (-6,2)
	75th percentile	33 (ref.)	**8** (4,12)	4 (-1,9)	-3 (-8,1)	-4 (-9,0)	**16** (11,21)	**-6** (-9,-2)	**22** (18,26)	**-9** (-16,-2)	-4 (-13,5)
	90th percentile	45 (ref.)	10 (-1,20)	11 (-4,26)	-4 (-19,11)	-1 (-14,12)	**20** (10,31)	-11 (-23,2)	**28** (10,46)	-11 (-26,4)	-3 (-15,10)
**Treatment interval (Screen-detected)**	Number	115	155	158	86	134	195	120	155	170	165
	Median	27 (ref.)	**5** (1,8)	**8** (4,11)	0 (-5,5)	**-6** (-10,-1)	**9** (7,12)	**-12** (-15,-10)	**12** (9,16)	-5 (-12,1)	**-3** (-6,-1)
	75th percentile	36 (ref.)	4 (-2,10)	10 (-3,22)	0 (-6,7)	-7 (-16,3)	**11** (3,20)	**-15** (-27,-4)	**15** (7,23)	-7 (-16,2)	-6 (-17,5)
	90th percentile	49 (ref.)	5 (-8,18)	**15** (4,25)	-9 (-20,3)	**-14** (-26,-2)	9 (-4,22)	**-24** (-35,-13)	12 (-1,24)	**-14** (-27,-1)	-11 (-21,0)
**Treatment interval (All)**	Number	231	359	361	330	368	400	341	353	366	278
	Median	25 (ref.)	5 (0,11)	4 (0,8)	-4 (-8,1)	**-5** (-9,-1)	**10** (6,14)	**-10** (-14,-6)	**14** (9,18)	**-10** (-14,-6)	-3 (-7,0)
	75th percentile	35 (ref.)	**6** (2,10)	**6** (1,10)	-4 (-8,0)	**-6** (-10,-2)	**13** (10,17)	**-11** (-16,-6)	**20** (16,23)	**-9** (-13,-5)	**-6** (-10,-2)
	90th percentile	46 (ref.)	**8** (2,14)	12 (-1,26)	**-6** (-10,-2)	**-6** (-9,-2)	**17** (11,23)	**-14** (-17,-12)	**23** (16,29)	**-11** (-14,-9)	**-7** (-10,-4)
**Total interval (Symptomatic)**	Number	104	190	186	205	189	173	184	155	177	98
	Median	70 (ref.)	**-14** (-24,-3)	**-12** (-23,-2)	**-20** (-31,-8)	**-25** (-38,-11)	**28** (15,42)	-15 (-34,5)	**22** (12,31)	**-28** (39,-17)	-1 (-11,10)

	75th percentile	96 (ref.)	**-16** (-27,-5)	-5 (-18,9)	**-18** (-33,-3)	**-25** (-39,-12)	**69** (31,107)	**27** (8,46)	**30** (8,53)	-13 (-27,0)	3 (-30,35)
	90th percentile	218 (ref.)	**-71** (-102,-41)	**-60** (-85,-34)	**-57** (-66,-48)	**-41** (-66,-16)	**79** (66,93)	8 (-15,30)	**-17** (-34,-1)	-21 (-67,26)	**-39** (-67,-10)
**Total interval (All)**	Number	217	343	337	291	320	354	298	277	327	253
	Median	60 (ref.)	**-9** (-14,-3)	-4 (-11,2)	**-13** (-18,-8)	**-15** (-26,-4)	**16** (6,26)	**-13** (-21,-6)	**17** (9,24)	**-18** (-24,-12)	**-17** (-25,-10)
	75th percentile	81 (ref.)	**-12** (-20,-4)	1 (-9,10)	**-13** (-20,-6)	**-14** (-20,-9)	**32** (24,41)	-4 (-12,4)	**35** (21,48)	**-19** (-25,-12)	**-17** (-26,-7)
	90th percentile	123 (ref.)	-13 (-34,9)	3 (-12,18)	-5 (-22,11)	-8 (-38,22)	**73** (33,114)	**32** (2,62)	**47** (13,82)	-12 (-37,13)	-24 (-51,4)

The differences for the median, 75th and 90th percentiles are calculated as marginal effects after quantile regression by setting the continuous covariate age to its mean value and comorbidity to the mode. Significant results are shown in bold. The actual number of days are included for Wales are shown in Table 5. Note that analyses of primary care intervals are presented in Table 6.

For peer review only

Additional File 7. *Agreement between the ICBP M4 Presentation-rule (without using registry data) and registry information to define if a breast cancer case was screen detected, in Wales, England, Scotland, N Ireland, Denmark, Manitoba and Sweden*

Jurisdiction	Number of cases	Agreement on screening-status between ICBP M4 and Registry %	Kappa	(95%CI)
Wales	227	96	0.91	(0.86-0.97)
England	341	95	0.89	(0.84-0.93)
Scotland	360	97	0.94	(0.90-0.97)
N Ireland	331	91	0.77	(0.69-0.84)
Denmark	342	91	0.81	(0.74-0.87)
Manitoba	364	84	0.68	(0.60-0.76)
Sweden	284	87	0.75	(0.67-0.82)

For peer review only

Additional File 8. ICBP M4 ethics, working and academic reference groups

Section 1 - Summary of the (ethical) approvals obtained in the ten jurisdictions for local data collection and data transfer to University College London (United Kingdom) and Aarhus University (Denmark).

	Date of Ethics Approval	Approvals obtained	Reference
Victoria	4 September 2012	Cancer Council Victoria Human Research Ethics Committee	HREC 1125
Manitoba	7 March 2013<br><br>15 April 2013	Health Research Ethics Board, University of Manitoba<br><br>Research Resource Ethics Committee, CancerCare Manitoba	HS15227 (H2012:105)<br><br>RRIC#28-2012
Ontario	7 November 2013<br><br>28 January 2014	University of Toronto Research Ethics Board	27881
Denmark	6 August 2013<br><br>19 June 2013	The Danish Data Protection Agency<br><br>According to Danish law and the Central Denmark Region Committees on Health Research Ethics, approval by the National Committee on Health Research Ethics was not required as no biomedical intervention was performed.	2013-41-2030<br><br>1-10-72-20-13
Sweden	23 October 2013	Ethics Review Board, Uppsala	2013/306
Norway	04 April 2013	Regional committees for medical and health research ethics	2013/136/REK nord
Wales	16 November 2012	NRES Committee East Midlands – Derby 2,<br><br>local R&D for each health board	11/EM/0420
Scotland	16 November 2012	NRES Committee East Midlands – Derby 2,<br><br>R&D for each health board,<br><br>Privacy Advisory Committee,<br><br>CHI Advisory Group	11/EM/0420
N Ireland	1 June 2012	ORECNI Ethical approval, local governance for each health Trust	12/NI/0053
England	16 November 2012	NRES Committee East Midlands – Derby 2<br><br>R&D for each Clinical Research Network	11/EM/0420

For peer review only

Section 2 – ICBP Module 4 Working Group

Alina Zalounina Falborg, Statistician, Research Unit for General Practice, Department of Public Health, Aarhus University, Bartholins Allé 2, 8000 Aarhus C, Denmark

Andriana Barisic, Research Associate, Department of Prevention and Cancer Control, Cancer Care Ontario, 620 University Avenue, Toronto, Ontario, M5G 2L7, Canada

Anna Gavin, Director, Northern Ireland Cancer Registry, Centre for Public Health, Queen's University Belfast, Mulhouse Building, Mulhouse Road, Belfast, BT12 6DP, United Kingdom

Anne Kari Knudsen, Administrative leader, Department of Cancer Research and Molecular Medicine, Norwegian University of Science and Technology, 7489 Trondheim, Norway

Breann Hawryluk, Project Planning Coordinator, Department of Patient Navigation, Cancer Care Manitoba, 675 McDermot Street, Winnipeg, Manitoba, Canada

Chantelle Anandan, Research Fellow, Centre for Population Health Sciences, University of Edinburgh, Doorway 1, Medical Quad Teviot Place, Edinburgh, EH8 9DX, United Kingdom

Conan Donnelly, Research Fellow, Centre for Public Health, Queen's University Belfast, Mulhouse Building, Mulhouse Road, Belfast, BT12 6DP, United Kingdom

David H Brewster, Scottish Cancer Registry, Information Services Division, NHS National Services Scotland, Gyle Square, 1 South Gyle Crescent, Edinburgh, EH12 9EB, United Kingdom

David Weller, James Mackenzie Professor of General Practice, Centre for Population Health Sciences, University of Edinburgh, Doorway 1, Medical Quad Teviot Place, Edinburgh, EH8 9DX, United Kingdom

Donna Turner, Epidemiologist/Provincial Director, Population Oncology, Cancer Care Manitoba, 675 McDermot Street, Winnipeg, Manitoba, Canada

Elizabeth Harland, Project Coordinator, Department of Epidemiology and Cancer Registry, CancerCare Manitoba, 675 McDermot Street, Winnipeg, Manitoba

Eva Grunfeld, Director, Knowledge Translation Research Network Health Services Research Program, Ontario Institute for Cancer Research; Professor and Vice Chair Research, Department of Family and Community Medicine, University of Toronto, 500 University Avenue, Toronto, Ontario, M5G 1V7, Canada

Evangelia Ourania Fourkala, Research Associate, Gynaecological Cancer Research Centre, Women's Cancer, Institute for Women's Health, University College London, United Kingdom

Henry Jensen, Research fellow, Research Unit for General Practice, Department of Public Health, Aarhus University, Bartholins Allé 2, 8000 Aarhus C, Denmark

Jackie Boylan, Research Fellow, Centre for Public Health, Queen's University Belfast, Mulhouse Building, Mulhouse Road, Belfast, BT12 6DP, United Kingdom

Jacqueline Kelly, Tumour Verification Officer, Northern Ireland Cancer Registry, Centre for Public Health, Queen's University Belfast, Mulhouse Building, Mulhouse Road, Belfast, BT12 6DP, United Kingdom

Kerry Moore, Research Fellow, Centre for Public Health, Queen's University Belfast, Mulhouse Building, Mulhouse Road, Belfast, BT12 6DP, United Kingdom

Maria Rejmyr Davis, Head, Southern Sweden Regional Cancer Center, Medicon Village, Scheelevägen 8, building 404, 223 81 Lund, Sweden

Martin Malmberg, MD PhD, Senior Consultant, Department of Oncology, Lund University Hospital, SE-221 85 Lund, Sweden

Mats Lambe, Professor of Medical Epidemiology, Regional Cancer Center Uppsala and Department of Medical Epidemiology and Biostatics, Karolinska Institutet, SE-171 77 Stockholm, Sweden

For peer review only

Oliver Bucher, Epidemiologist, Department of Epidemiology and Cancer Registry, CancerCare Manitoba, 675 McDermot Street, Winnipeg, Manitoba

Peter Vedsted, Professor, Research Unit for General Practice, Department of Public Health, Aarhus University, Bartholins Allé 2, 8000 Aarhus C, Denmark

Rebecca Bergin, Senior Research Officer/ PhD Candidate, Centre for Behavioural Research in Cancer, 615 St Kilda Rd, Melbourne, Victoria, 3004, Australia

Rebecca-Jane Law, Research Project Support Officer, North Wales Centre for Primary Care Research, Bangor University, Gwenfro Units 4-8, Wrexham Technology Park, Wrexham, LL13 7YP, United Kingdom

Richard D Neal, Professor of Primary Care Oncology, Academic Unit of Primary Care, Leeds Institute of Health Sciences, University of Leeds, Leeds LS2 9NL, United Kingdom

Sigrun Saur Almberg, Researcher, Department of Cancer Research and Molecular Medicine, Faculty of Medicine, Norwegian University of Science and Technology (NTNU), N-7491 Trondheim, Norway

Therese Kearney, Research fellow, Northern Ireland Cancer Registry, Centre for Public Health, Queen's University Belfast, Mulhouse Building, Mulhouse Road, Belfast, BT12 6DP, United Kingdom

Tindie Kalsi, Project Manager, Gynaecological Cancer Research Centre, Women's Cancer, Institute for Women's Health, University College London, United Kingdom

Victoria Cairnduff, Statistician, Northern Ireland Cancer Registry, Centre for Public Health, Queen's University Belfast, Mulhouse Building, Mulhouse Road, Belfast, BT12 6DP, United Kingdom

Victoria Hammersley, Researcher, Centre for Population Health Sciences, University of Edinburgh, Doorway 1, Medical Quad Teviot Place, Edinburgh, EH8 9DX, United Kingdom

Victoria White, Deputy Director, Centre for Behavioral Research in Cancer, Cancer Council Victoria, 615 St Kilda Road, Melbourne, Victoria, 3004, Australia

Usha Menon, Professor of Gynaecological Oncology and Head, Gynaecological Cancer Research Centre, Women's Cancer, Institute for Women's Health, University College London, United Kingdom

Yulan Lin, Postdoc, Department of Cancer Research and Molecular Medicine, Faculty of Medicine, Norwegian University of Science and Technology (NTNU), N-7491 Trondheim, Norway

Section 3 – ICBP Module 4 Academic Reference Group:

Prof Jan Willem Coebergh, Professor of Cancer Surveillance, Department of Public Health, Erasmus Universiteit Rotterdam, Rotterdam, the Netherlands

Jon Emery, Professor of Primary Care Cancer Research, University of Melbourne and Clinical Professor of General Practice, University of Western Australia, Australia

Dr Stefan Bergström, Senior consultant oncologist, Department of Oncology, Gävle, Sweden

Dr Monique E van Leerdam, Erasmus MC University Medical Centre, the Netherlands

Prof Marie-Louise Essink-Bot, Academic Medical Centre, Amsterdam University, the Netherlands

Prof Una MacLeod, Senior Lecturer in General Practice and Primary Care, Hull-York Medical School, United Kingdom

For peer review only
